# A Crystalline In(II) Hydride

**DOI:** 10.1021/jacs.5c22490

**Published:** 2026-01-30

**Authors:** Olympia Mouriki, Graham J. Tizzard, Simon J. Coles, Diego M. Andrada, Oriol Planas

**Affiliations:** † Department of Chemistry, Molecular Sciences Research Hub, 4615 Imperial College London, 82 Wood Lane, Shepherds Bush, London W12 0BZ, U.K.; ‡ EPSRC National Crystallography Service, School of Chemistry, 7423 University of Southampton, Southampton SO17 1BJ, U.K.; § General and Inorganic Chemistry Department, University of Saarland, Campus C4.1, Saarbrücken 66123, Germany

## Abstract

Low oxidation state hydride species of heavier main-group elements are notoriously elusive due to their intrinsic instability and rapid decomposition, generating hydrogen. Nevertheless, they offer significant potential for small-molecule activation and catalysis. Herein, we report the synthesis and characterization of the first stable, crystalline, low oxidation state indium hydride, supported by bis­(*N*-heterocyclic carbene)­borate ligands and featuring a covalent In–In bond. This compound has been comprehensively characterized by NMR spectroscopy, FT-IR, single-crystal X-ray diffraction, and computational calculations. Experimental and theoretical studies reveal key features that underpin its exceptional stability. Preliminary reactivity investigations demonstrate that this discrete In­(II) hydride acts as a nucleophile, opening new avenues for bond activation involving hydrides derived from the heaviest p-block elements.

## Graphical abstract

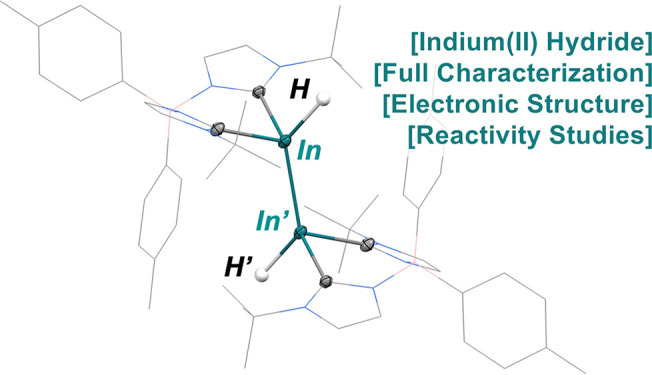

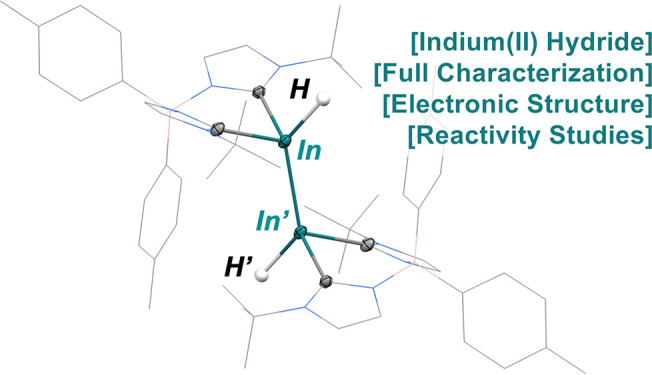

## 1 Introduction

Hydrides are central intermediates in chemistry, underpinning processes from hydrogenation and reduction to small-molecule activation and hydrogen storage.^1–4^ While transition-metal hydrides have long dominated this field,^5,6^ main group metal hydrides are gaining ground as powerful alternatives,^7,8^ as they are capable of emulating transition-metal behavior while avoiding reliance on scarce and expensive d-block elements.^9,10^ This is the case for groups 14 and 15 metal hydrides,^11–13^ several of which have been isolated or applied to organometallic fundamental processes.^14–18^ In parallel, group 13 metal hydrides have emerged as an equally rich class, with well-defined species of aluminum and gallium now extending into unusual oxidation states (Figure 1A, species **I**–**III**), including isolable Al­(I)–H,^19,20^ Al­(II)–H,^21–23^ and Ga­(II)–H complexes.^24–27^

**Figure 1:**
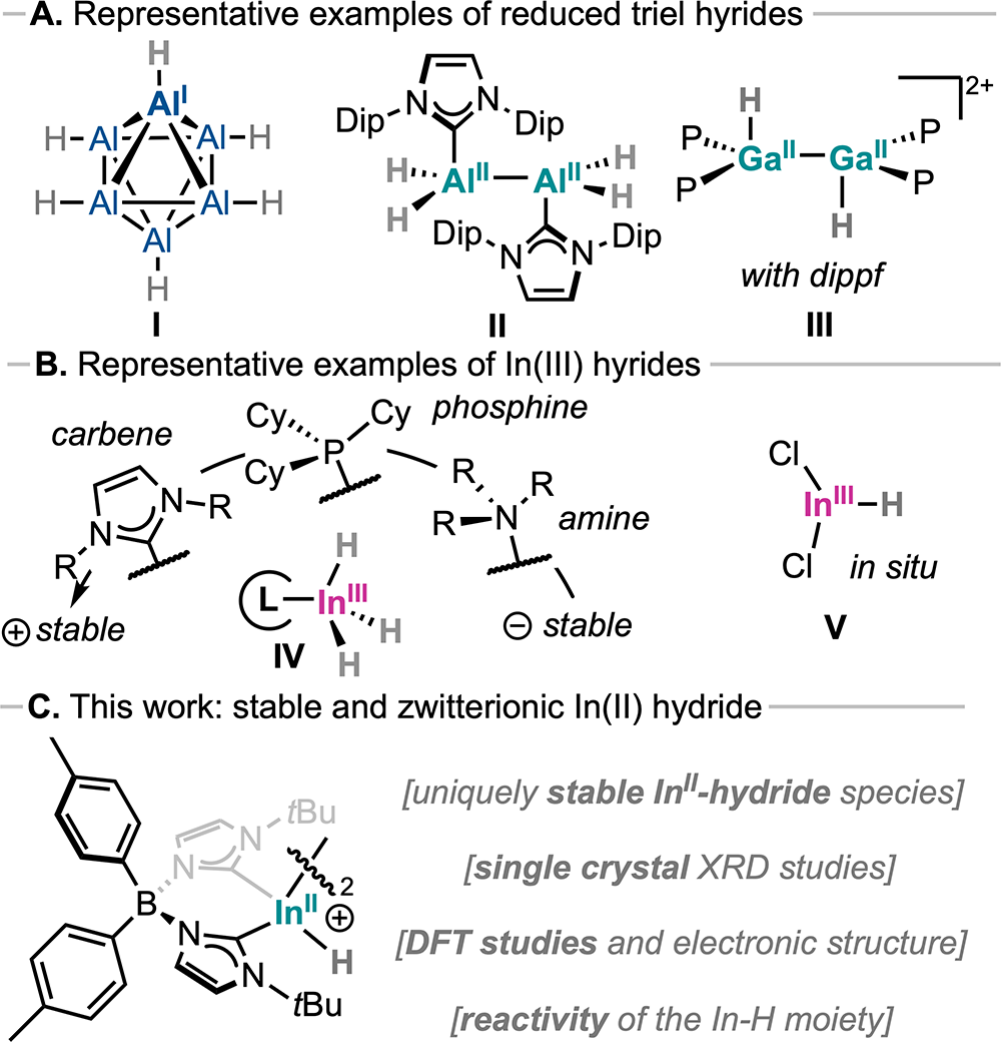
Representative examples of (A) low oxidation state triel hydrides and (B) In­(III) hydrides. (C) This work: use of monoanionic bis­(NHC)­borate ligands for the synthesis of an unprecedented zwitterionic In­(II) hydride species.

In striking contrast, the heavier hydride congeners, indium and thallium, remain underexplored. Molecular In­(III) hydrides stabilized by strong σ-donor ligands such as amines, phosphines, or *N*-heterocyclic carbenes (NHC) have been isolated and structurally characterized (Figure 1B, species **IV**),^28–38^ establishing the viability of In–H bonding in its highest oxidation state. In addition, in situ generated Cl_2_In–H intermediates (Figure 1B, species **V**) have also been proposed to participate in both polar and radical-based reduction reactions,^39–44^ providing applications in organic synthesis.^45–47^ Yet, low oxidation state In hydrides have remained entirely elusive.^48^ Previous efforts have been limited to transient observations in the gas phase or under cryogenic matrix-isolation conditions.^49,50^ Their inherent low stability leads to decomposition via low-energy pathways involving In–H–In bridges and facile H_2_ elimination, eventually producing In black.^51,52^ The lack of well-defined, discrete low-oxidation state heavier triel-hydride complexes has led to the long-standing view that such species are largely inaccessible, although some promising heterobimetallic species containing Tl­(I)···H interactions have been recently characterized.^53–55^ Low oxidation state heavy triel-hydrides are expected to display highly polarized E–H bonds and enhanced redox flexibility, potentially enabling reactivity modes not accessible to higher-valent species, but none of these possibilities can be assessed without first isolating well-defined molecular hydrides in oxidation states below +3.

Inspired by the recent emergence of carbene-based low-valent group 13 chemistry,^56–58^ we hypothesized that the bulky, anionic bis­(NHC)­borate ligand introduced by Hofmann and co-workers would be an excellent candidate to stabilize low oxidation state In hydride species,^59^ as it offers both strong donor properties and steric protection.^60–63^ Indeed, our group has recently demonstrated the ability of such platforms to stabilize low-valent main group species, reporting the synthesis of a family of heavier pnictogen complexes across a range of oxidation states, and their ability to engage in redox catalytic processes.^64^

Herein, we describe the synthesis and characterization of the first stable, crystalline In hydride in an oxidation state below +3. The stability and reactivity of this unprecedented species have been investigated through detailed spectroscopic, crystallographic, and computational studies, providing key insights of its electronic structure and bonding. These findings not only account for the rare stability of this compound and its reactivity with electrophiles but also offer design principles for isolating other highly unstable heavy main group hydrides, introducing a blueprint for extending hydride chemistry into previously inaccessible oxidation states.

## 2 Results

Initially, ligand **1-PF**_**6**_ was used to synthesize In­(III) complexes **2-X** (X = Br, I; Scheme 1A), which were isolated as white powders.^65^ While **2-Br** was obtained analytically pure in excellent yield, **2-I** required purification by several rounds of recrystallization, which led to an overall poor yield. The ^1^H NMR spectrum of **2-Br** in CDCl_3_ indicates a symmetric structure, displaying two pairs of aromatic doublets corresponding to the tolyl (δ_H_ = 7.02 and 6.59 ppm) and imidazole (δ_H_ = 7.18 and 6.93 ppm) fragments of the ligand scaffold.

**Scheme 1:**
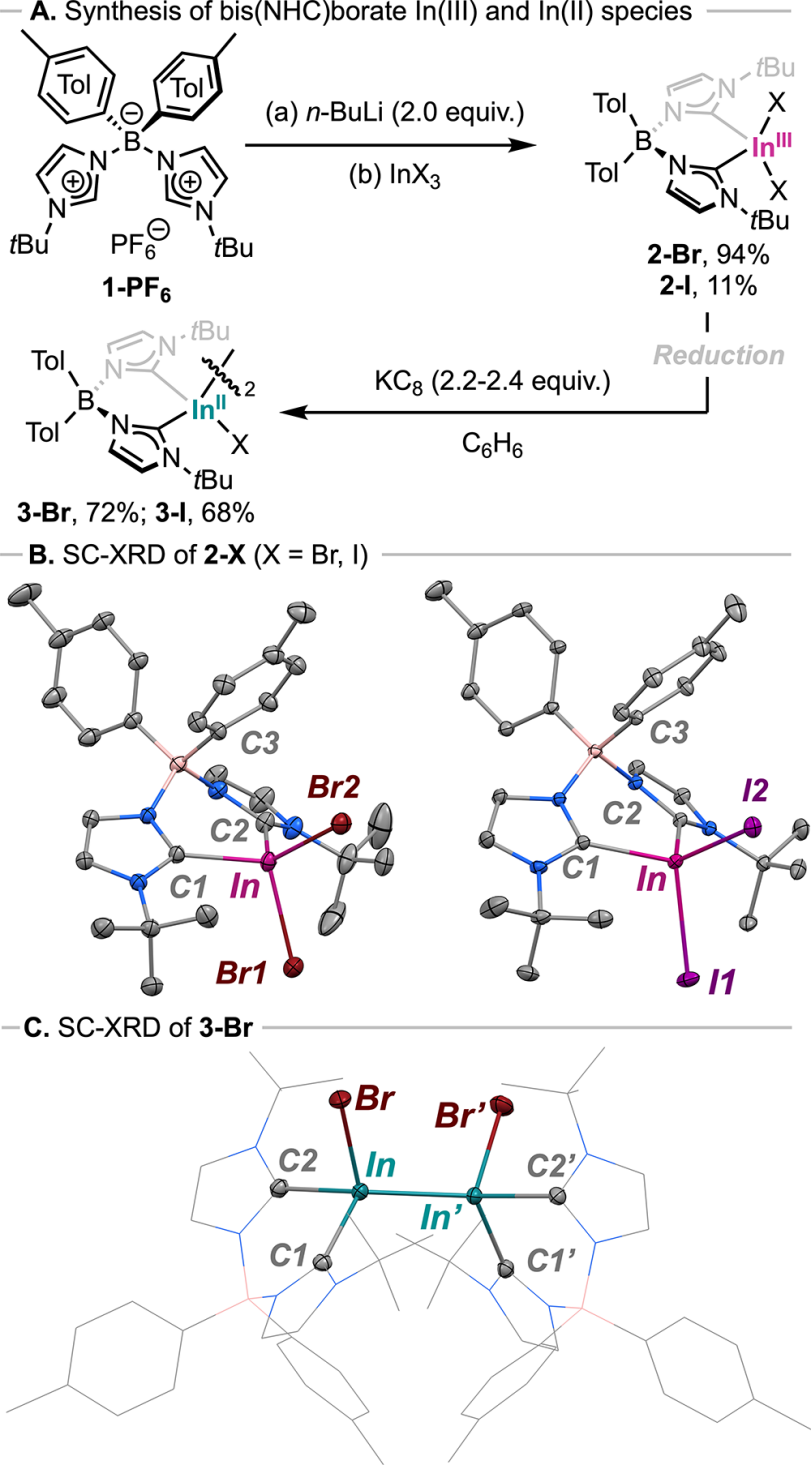
(A) Synthesis of In Species **2-X** and **3-X** (X = Br and I).^a^

a For Experimental Details, See Supporting Information. (B) Molecular structure of **2-Br** and **2-I**. (C) Molecular structure of **3-Br**. H-atoms and solvent molecules are omitted for clarity; ellipsoids displayed at 50% probability.

Analysis of ^13^C NMR spectrum of **2-Br** shows the expected resonances, with the exception of those corresponding to the carbon atoms connected to In and B. The In–C signal was identified in ^1^H–^13^C HMBC experiments, revealing a crosspeak at δ_C_ = 164.5 ppm. This chemical shift is consistent with previously reported bis­(NHC)­borate-supported main-group species^64^ and is slightly more deshielded than in analogous NHC-In­(III) halide compounds,^34,38,66–68^ likely reflecting the partial positive charge on the In center due to the zwitterionic electronic structure of compound **2-Br**. Crystallization of **2-Br** via slow evaporation of a hexane solution resulted in a crystal structure displaying an In center with a slightly distorted tetrahedral coordination (see Scheme 1B), with tetrahedral geometry indexes τ_4_ = 0.87 and τ_4_′ = 0.86.^69,70^ Interestingly, **2-Br** also displays a short distance between the In center and a tolyl ring [*d*_C3–In_ = 3.265(1) Å; sum of van der Waals radii = 3.6 Å], likely arising from a weak interaction between the nucleophilic tolylborane unit and the electrophilic In center. This is also reflected on the larger In–Br1 bond distance [2.5479(2) Å] compared to In–Br2 [2.4994(2) Å]. In addition, In–C_carbene_ bond lengths [2.189(1) Å and 2.192(1) Å] are slightly shorter than the typical range of C_carbene_–In bond distances reported (2.199 to 2.235 Å)^34,38,66–68^ probably due to the strong σ-donor ability of bis­(NHC)­borate ligands, as discussed in the case of pnictogen analogues.^64^ In addition, the In­(III)–C bond distance obtained is consistent with the sum of single-bond atomic radii for In and C (2.17 Å).^27^ Species **2-I** displays similar spectroscopic and structural features (Scheme 1B).^65^

Reduction of **2-Br** with 2.2 equiv of KC_8_ in benzene afforded diindane species **3-Br** as a white solid in 72% yield. The ^1^H NMR spectrum in C_6_D_6_ revealed the equatorial desymmetrization of the structure, showing two sets of doublets for each tolyl ring, consistent with a dimeric species (see Figure S50). Notably, the ^13^C NMR carbene resonance of **3-Br** appears at δ_C_ = 170.7 ppm in C_6_D_6_, indicating electronic properties closely resembling those of the parent In­(III) compound. In­(II) species **3-I** was obtained using a similar method and displayed equivalent spectroscopic features. Diindium complex **3-Br** was crystallized from ether/pentane, and its structure was unambiguously confirmed by Single-Crystal X-ray Diffraction (SC-XRD, Scheme 1C). SC-XRD analysis of **3-Br** displays In centers with a distorted tetrahedral geometry (τ_4_ = 0.81 and τ_4_′ = 0.80) and adopts a *gauche* conformation, with both bromide anions in *synclinal* position and a Br–In–In–Br′ dihedral angle of 61.2°. The In–In bond length [2.7752(4) Å] remains within the range of previously reported dimeric In­(II) species,^71–74^ as determined by a survey of the Cambridge Structural Database (63 crystal structures, from 2.65 to 2.97 Å).^75^ In addition, it displays longer In–Br [2.6242(7) Å], In–C1 [2.229(2) Å], and In–C2 [2.228(2) Å] distances compared to **2-Br**, consistent with more electron-rich reduced In center. Interestingly, the In­(II) dimer **3-Br** exhibits remarkable stability, remaining intact in the solid state and in solution under air for several weeks without noticeable decomposition, an unusual behavior for a low oxidation state In species. We speculated that this stability arises from the steric hindrance of the bis­(NHC)­borate ligand, which provides a high degree of axial and equatorial protection through its tolyl and *tert*-butyl substituents, respectively.

Motivated by this observation, we next investigated the reactivity of **3-Br** with hydride sources, aiming at the synthesis of the first stable low oxidation state In–H species. To this end, **3-Br** was treated with a range of inorganic and organic hydride reagents, including KH, LiAlH_4_, and K-Selectride. Whereas most hydride sources left the initial In complex unreacted or led to decomposition, the reaction of **3-Br** with 2.2 equiv of K-Selectride in dry ether resulted in the formation of a new species, consistent with the targeted In–H species **3-H** (Scheme 2A), which could be isolated in 30% yield after crystallization. Similarly to the dihalide analogues **3-X** (X = Br, I), dihydride **3-H** displays a diamagnetic NMR spectrum (see Scheme 2B) with an asymmetric equatorial plane in solution, showing two sets of doublets for each tolyl ring. Importantly, a broad singlet integrating for 2H was observed at δ_H_ = 6.27 ppm in C_6_D_6_ (δ_H_ = 5.65 ppm in THF-*d*_8_), which was assigned to the In–H moiety. This assignment is consistent with previously reported In­(III)–H species,^28–34,38^ which display similarly broad signals in the δ_H_ = 5 – 6 ppm range due to the quadrupolar nature of the In center (^115^In 95%, *I* = 9/2; ^113^In 5%, *I* = 9/2). Additionally, through-space ^1^H–^1^H NOESY correlations were observed between the broad resonance at δ_H_ = 6.27 ppm and the *tert-*butyl groups of the ligand (δ_H_ = 1.32 ppm in C_6_D_6_), providing further evidence for an In­(II)–H moiety located in the proximity of the ligand scaffold. Such interactions were further analyzed by noncovalent interaction plots (see Figure S28). ^1^H–^1^H NOESY correlations were also detected between the *tert-*butyl groups substituents and the tolyl groups of the ligand backbone (Figure S2), suggesting an intramolecular interaction consistent with a structure in which the In–In bond is retained in solution. This was further supported by DOSY NMR analysis of species **2-Br**, **3-Br**, and **3-H**, which revealed a diffusion coefficient for species **3-H** consistent with a diindium species.^65^ Furthermore, variable-temperature ^1^H NMR in THF-*d*_8_ and C_6_D_6_ showed no significant change over the temperature range investigated (−70 to 60 °C, see Figures S4 and S5). Taken together, this evidence excludes a dynamic monomer–dimer equilibrium and supports the formulation of **3-H** as a diindium species in solution.

**Scheme 2:**
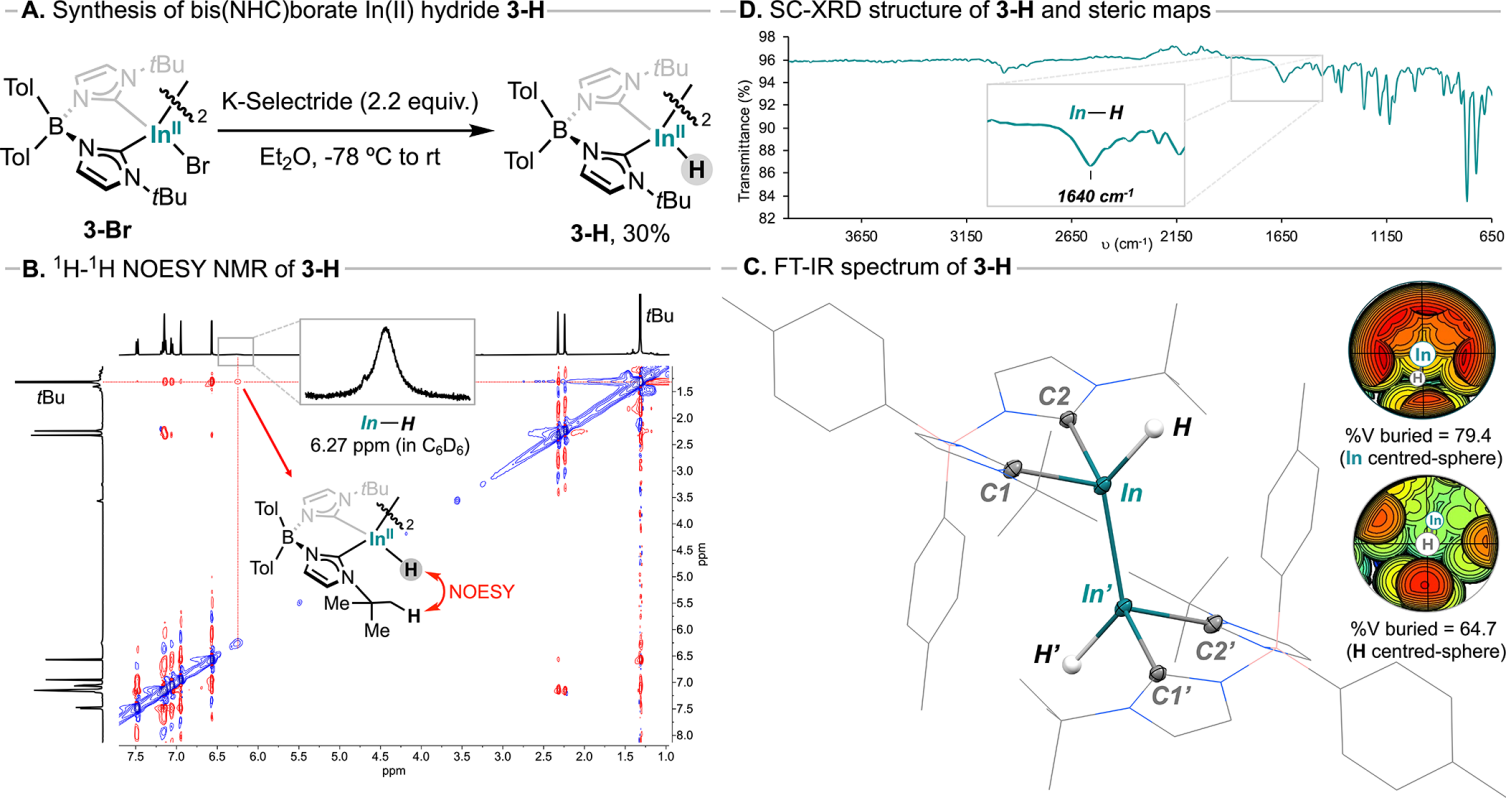
(A) Synthesis of In­(II) Hydride **3-H**,(B) ^1^H-^1^H NOESY NMR spectrum of 3-H in C_6_D_6_, Inset: broad signal at δ_H_ = 6.27 ppm assigned to the In–H moiety, (C) FT-IR spectrum of solid 3-H, Inset: broad vibrational signal at υ = 1640 cm^–1^ assigned to In–H stretching, (D) Molecular structure of 3-H; H-atoms and solvent molecules are omitted for clarity with exception of H-atoms directly attached to In; ellipsoids displayed at 50% probability, Inset: steric map and buried volume (%), sphere set at 5 Å.

Species **3-H** was also analyzed in the solid state. The infrared spectrum (FT-IR, Scheme 2D) of solid **3-H** displays a characteristic broad ν­(In–H) stretching band 1640 cm^–1^, which is similar to the previously reported In–H stretching bands for In­(III)–H species, particularly those bearing strong σ-donor carbene ligands.^32,38^ The band is also consistent with the simulated spectra, which predicts ν­(In–H) stretching at 1620 cm^–1^ (*syn* isomer*)* and 1730 cm^–1^ (*anti* isomer, see Figure S25). Compared to lighter low oxidation state hydride analogues, **3-H** exhibits a considerably lower wavenumber, consistent with a weaker In–H bond and the heavier mass of In. SC-XRD analysis of **3-H** revealed the presence of the expected In–In bond (Scheme 2B) in which each In center displays a distorted tetrahedral geometry (τ_4_ = 0.80 and τ_4_′ = 0.75), similarly to the bromide precursor **3-Br**. Hydride ligands were located in the difference maps, and their positional and isotropic thermal parameters were refined. Complex **3-H** displays an In­(II)–H bond length of 1.75(2) Å, which is slightly larger compared to previously reported structurally characterized In­(III)–H bonds,^30,33–38^ and consistent with the computationally optimized structure (*d*_In–H_ = 1.747 Å, vide infra). Importantly, no evidence of intra- and intermolecular interactions via bridging hydrides was observed, supporting the formulation of **3-H** as a terminal In­(II)–H dimer. Compared to **3-Br**, dihydride **3-H** presents the same In–In bond length [2.7734(3) Å] and slightly longer In–C1 [2.2695(9) Å] and In–C2 [2.265(1) Å] bond distances, likely due to an increase of electron density at the In center. In contrast to **3-Br**, dihydride **3-H** adopts a textbook staggered conformation in the solid state, with a H–In–In′–H′ dihedral angle of 180°. Interestingly, short intramonomer In­(II)–H···H–C contacts (*d*_H–H_ < 2.4 Å) involving the *t*Bu substituents are evident in the crystal structure, consistent with the ^1^H–^1^H NOESY correlations observed in solution. Likewise, intermonomer contacts between *tert-*Bu and tolyl groups are also present (*d*_H–H_ = 2.7 to 3.3 Å), in line with the corresponding NOESY crosspeaks and providing further support to the formulation of **3-H** as a dimeric species.

Remarkably, **3-H** also displays an unexpected degree of robustness. The compound shows good air stability in the solid and solution states, retaining its integrity upon exposure for several hours, eventually decomposing (Figures S23 and S24), and remaining stable for several hours at high temperatures (Figures S21 and S22). Indeed, prolonged heating of a sealed C_6_D_6_ solution of **3-H** resulted in a complex mixture of products, but the formation of hydrogen was not detected in solution. This outcome contrasts sharply with recent observations for heavier main group hydrides, where thermal decomposition is often accompanied by hydrogen evolution.^12^ Consistent with this result, computational studies indicate a substantial activation barrier of 48.6 kcal mol^–1^ for H_2_ elimination from **3-H**, suggesting that this event is inaccessible (Figure S31). Interestingly, **3-H** can be handled in solution under air for up to 1 h before noticeable decomposition occurs. Such resilience is exceptionally rare among low oxidation state main group hydrides, which are typically highly air- and moisture-sensitive, undergoing rapid oxidation, protonation, or decomposition upon air exposure. This unusual stability can be attributed to the axial and equatorial steric shielding provided by the bis­(NHC)­borate ligand framework, as evidenced by the calculated %V_Bur_ of 79.4% at the In center and 64.7% at the hydride moiety,^76^ which preserves the low oxidation state In­(II) center and reduces accessibility of the In–H bond, as visualized in the steric maps (Scheme 2D, inset). In addition, intramolecular noncovalent contacts (i.e., London Dispersion Forces)^77^ between the In–H bond and *t*Bu substituents observed in both solid state and solution likely provide an extra layer of stabilization and protection, contributing to the overall robustness of In–In and In–H bonds.

Given the intriguing properties and robustness of **3-H**, a computational study was carried out to rationalize its structure and behavior. The SC-XRD structure of **3-H** served as the starting point for the geometry optimization, performed at the PBE0-D4/def2-TZVPP level of theory (Table S2),^78–83^ which resulted in structural parameters in good agreement with experimental data. The Kohn–Sham molecular orbitals show the HOMO (ε = −5.28 eV) corresponding to the In–In bond (Figure 2A), with some delocalization onto the terminal hydride ligands, while the LUMO (ε = −0.16 eV, Figure S26) is delocalized onto the aryl substituents on the borane fragment. Additional In–H bond orbitals are computed lower in energy, corresponding to HOMO–12 (ε = −6.98 eV, Figure 2A) and HOMO–13 (ε = −7.03, Figure S26), for the out-of-phase and in-phase combination, respectively. Natural Bond Orbitals (NBOs) analysis revealed four highly polarized In (18.6%) – C_carbene_ (81.4%) bonds and two polarized In (31.9%) – H (68.1%) bonds, with the dimer stabilized through a covalent In–In bond (Figure 2B, Table S4). The Wiberg bond indexes (WBI) of In–H and In–In bonds are consistent with a single bond character, with values of 0.80 and 0.88, respectively, in agreement with previously predicted indium hydride species (Figure 2C).^51,52^ The In–C_carbene_ WBI value (0.56) is significantly lower compared to those reported for related pnictogen species bearing the same ligand scaffold, which show values of 0.91 (**Sb**) and 0.86 (**Bi**).^64^ This result is consistent with a higher degree of E–C bond polarization in In complexes compared to heavier pnictogens such as Sb or Bi. Furthermore, Natural Population Analysis (NPA) indicates that the In center carries substantial positive charge (+0.59*e* and +0.582*e*), while hydride ligands bear a negative charge (−0.34*e*), indicative of their nucleophilic nature. The overall In_2_H_2_ central unit is positively charged by +0.5*e*.

**Figure 2:**
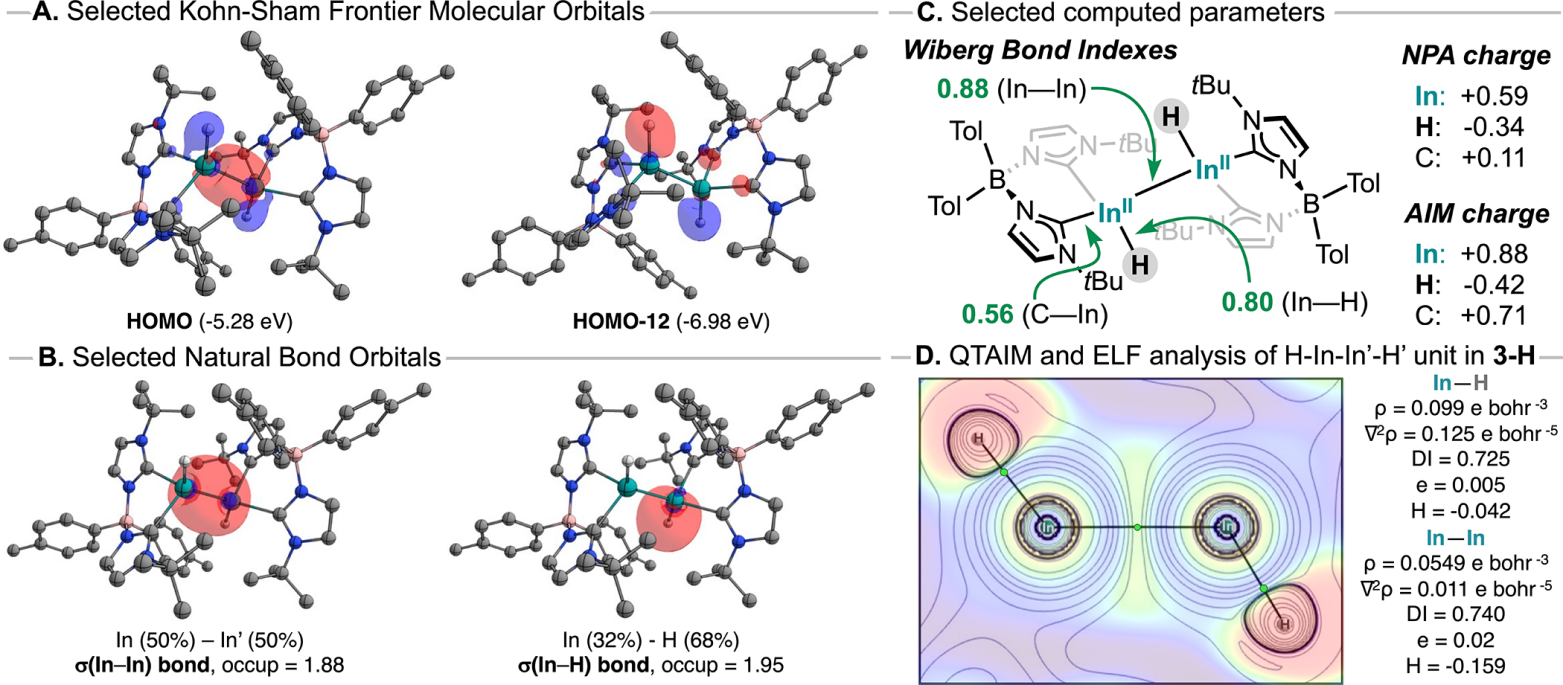
(A) Kohn–Sham Frontier Molecular Orbitals of **3-H**. (B) Selected NBOs of **3-H**. H-atoms and solvent molecules are omitted for clarity with the exception of H-atoms directly attached to In. (C) Wiberg Bond Indexes and partial charges (NPA and QT-AIM). (D) Laplacian distribution of the electron density overlaid with the 2D map of electron localization function for the molecular plane defined by the atoms H–In–In′-H′ (high electron localization areas are shown in red, whereas regions of low localization are shown in blue). Contour line diagrams of the Laplacian distribution ∇^2^ρ­(*r*): Black lines indicate areas of charge concentration (∇^2^ρ­(*r*)<0), while solid blue lines show areas of charge depletion (∇^2^ρ­(*r*)>0); the thick solid lines connecting the atomic nuclei are the bond paths and the small green dots are the Bond Critical Points (BCP).

The electron density (ρ­(*r*)) distribution on the H–In–In–H plane of **3-H** was analyzed within the Atoms in Molecules (QTAIM) framework.^84,85^ Figure 2D displays the resulting contour plot of the Laplacian of the electron density (∇^2^ρ­(*r*)) along the H–In–In–H plane. The In–H bond critical point (BCP) exhibits electron density and Laplacian values of ρ­(*r*) = 0.099e bohr^–3^ and ∇^2^ρ­(*r*) = 0.125e bohr^–5^, respectively, with a negative value of local electron density H.^86,87^ These parameters for the In–H unit indicate a 2c–2e polar covalent bond, ranging to the typical values computed for other indium hydrides.^88,89^ The In–In BCP bears a lower but significant ρ­(*r*) value of 0.055e bohr^–3^ and ∇^2^ρ­(*r*) = +0.011e bohr^–5^. Note the Laplacian shows negligible electron accumulation on the In–In bond; however, electron localization function analysis (ELF, Figure 2D overlay and Figure S27) is able to provide a disynaptic basin description V­(In,In) = 1.86. These values, together with a relatively large negative value of local electron density H (−0.159 au), suggest a metal–metal interaction.^88^ The Laplacian plot on the C_carbene_–In–C_carbene_ planes outlines the electron density accumulation around the carbene carbon atoms (Table S6). The Local density parameters at the In–C BCPs (ρ = 0.082e bohr^–3^, ∇^2^ρ = +0.194e bohr^–5^, H = – 0.231, ϵ = 0.07) indicate an appreciable covalent stabilization (H < 0) but modest electron sharing (Delocalization index = 0.56). This result is consistent with NBO (81% C*sp* ^2^ contribution) and WBI (0.56) analysis and points to a highly polarized C–In dative interaction.

To gain further insights into the distinctive stability of the In­(II)–H species **3-H**, energy decomposition analysis (EDA)^90–92^ in combination with the natural orbitals for chemical valence (NOCV)^93–95^ method was performed (Table 1).^96^ The In–In bonding interaction in **3-H** was examined first, and for comparison, a yet-unrealized diindane complex supported by a dimesityl β-diketiminate (NacNac) ligand was also computed (**NacNac**_**2**_ **In**_**2**_ **H**_**2**_ **)**, for which the corresponding bromide analogue has been reported.^73^ When the individual monomers are selected as distinct fragments in **3-H**, EDA results reveal a rather stable chemical bond (*D*_*e*_ = 73.3 kcal mol^–1^, see Table S9). The energy penalty caused by distortion represents only 4.5 kcal mol^–1^ per monomer, leading to an interaction energy of −82.4 kcal mol^–1^. This interaction energy value is ca. 30 kcal mol^–1^ stronger with respect to previously computed diindane species with formula [(PMe_3_)_2_(In_2_H_4_)].^51^ Further dissection of the energy terms reveals a bond with a strong electrostatic interaction (Δ*E*_elstat_, 52.8%) and smaller orbital contribution (Δ*E*_orb_, 29.3%), with dispersion (Δ*E*_disp_, 17.9%) providing additional stabilization. The orbital term is mainly the σ-type interaction (Δ*E*_orb‑σ_, 83.1%, see Figure S29), consistent with the formation of an In­(II)–In­(II) bond. Similar values are obtained for the NacNac_2_In_2_H_2_ analogue (see Table S9). These results, together with topological features of the electron density, suggest that the In–In bond in **3-H** is best described as a σ-type metallic interaction that is electrostatically dominated with a small but significant orbital interaction. Note that the term electrostatic interaction comes from electrostatic attraction between the charge distribution of the selected fragments, thereby distinguishing from the ionic bonding within the VB framework.^97^

**Table 1:** EDA-NOCV Results at the BP86-D3­(BJ)/TZ2P Level of Theory^a^

	**3-H**	**NacNac**_**2**_ **In**_**2**_ **H**_**2**_
fragmentation	[L_2_]^2–^ (S)^b^;[In_2_H_2_]^2+^(S)	[L_2_]^2–^ (S)^b^;[In_2_H_2_]^2+^(S)
Δ*E*_int_	–648.1	–582.2
Δ*E*_Pauli_	410.2	306.0
Δ*E*_disp_ ^c^	–45.9 (4.3%)	–36.9 (4.2%)
Δ*E*_elst_ ^c^	–670.4 (63.3%)	–563.7 (63.5%)
Δ*E*_orb_ ^c^	–342.0 (32.3%)	–287.6 (32.4%)
Δ*E*_orb‑σ(+,+,–,−)‑don_ ^d^	–87.7 (25.6%)	–69.2 (24.1%)
Δ*E*_orb‑σ(+,+,+,+)‑don_ ^d^	–57.4 (16.8%)	–48.2 (16.8%)
Δ*E*_orb‑σ(+,–,–,+)‑don_ ^d^	–37.0 (10.8%)	–31.8 (11.1%)
Δ*E*_orb‑σ(+,–,+,−)‑don_ ^d^	–27.9 (8.2%)	–29.3 (10.2%)
Δ*E*_orb‑rest_ ^d^	–159.9 (46.8%)	–138.4 (48.1%)

a All values are reported in kcal·mol^–1^. All calculations were performed on the PBE0-D3­(BJ)/def2-TZVP optimized structures.

b S stands for singlet electronic state.

c The value in parentheses gives the percentage contribution to the total attractive interactions Δ*E*_elst_^+Δ*E*^_orb_^+Δ*E*^_disp_·

d The value in parentheses gives the percentage contribution to the total orbital interaction term.

Next, the focus was directed toward the interaction between the H–In–In′–H′ core and the bis­(NHC)­borate ligands, with comparison to the NacNac analogue. The fragments were defined as [In_2_H_2_]^2+^ and [L_2_]^2–^, thereby treating the interaction as donor–acceptor, although alternative fragmentations are also feasible. The EDA results are summarized in Table 1. The interaction energy between the [In_2_H_2_]^2+^ moiety and the ligands is −648.1 kcal mol^–1^ for **3-H**, compared to −582.2 kcal mol^–1^ for **NacNac**_**2**_ **In**_**2**_ **H**_**2**_. Further partitioning of the interaction energy reveals similar relative contributions to the total stability for both systems, i.e., 4% dispersion, 63% electrostatic, 32% orbital interaction. However, in absolute terms, complex **3-H** exhibits a markedly stronger interaction, with increases of 9 kcal mol^–1^ in dispersion, 107 kcal mol^–1^ in electrostatics, and 54.4 kcal mol^–1^ in orbital interactions, despite the longer predicted donor–acceptor distance (In–C 2.24 Å in **3-H** vs In–N 2.20 Å in **NacNac**_**2**_ **In**_**2**_ **H**_**2**_). This enhanced stabilization is partially offset by the larger Pauli repulsion observed for **3-H**.

Although the bonding within this fragmentation scheme is predominantly governed by electrostatic contributions (63%), it is informative to analyze the origins of the covalent component through the NOCV method. Four principal orbital interaction channels were identified, with all remaining contributions grouped as “rest of orbital terms” (Table 1). Figures 3 and S30 illustrate the associated deformation densities and the pairwise orbitals involved in **3-H** and **NacNac**_**2**_ **In**_**2**_ **H**_**2**_, respectively. These dominant interactions correspond to σ-donation from the lone pairs on the carbene carbon atoms (or nitrogen atoms in the NacNac analogue) into the vacant orbitals of the [In_2_H_2_]^2+^ fragment. The first channel arises from donation from lone pairs of phase (+,+,–,−) into the LUMO of [In_2_H_2_]^2+^. The second involves in-phase lone pairs (+,+,+,+) interacting with LUMO+2, while the third and fourth channels correspond to combinations (+,–,–,+) and (+,–,+,−), respectively. Summing these four contributions provides an estimate of the overall donor strength (i.e., stabilizing ability) of the ligand framework: −210.0 kcal mol^–1^ for the bis­(NHC)­borate ligand, compared with −178.6 kcal mol^–1^ for NacNac. Overall, the stronger interactions delivered by the bis­(NHC)­borate framework account for the efficient stabilization of the [In_2_H_2_]^2+^ core in **3-H**, highlighting that an optimized donor ligand is crucial for accessing low oxidation state In­(II)–H species.

**Figure 3:**
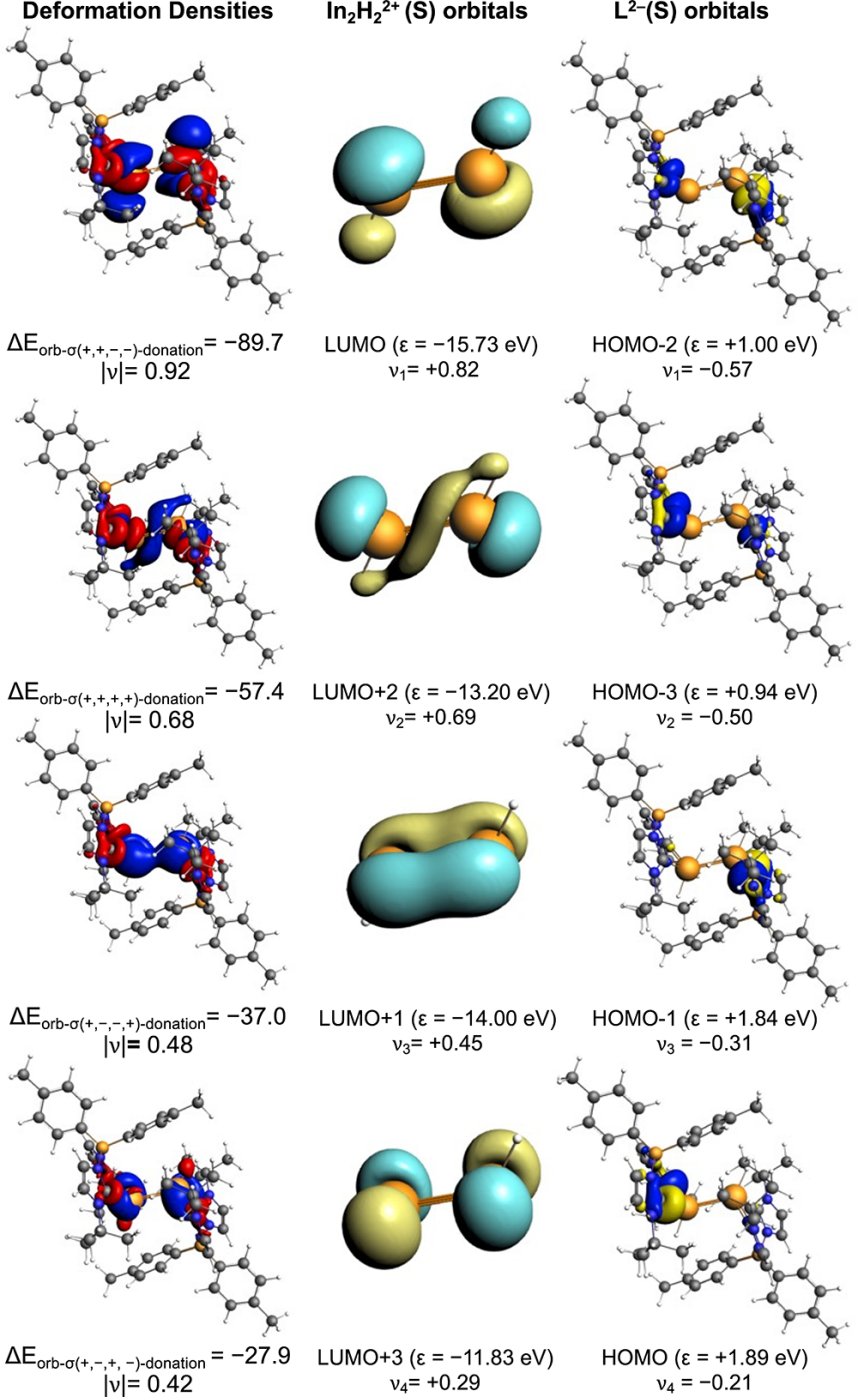
Plot of deformation densities Δρ (isocontour value = 0.001) of the pairwise orbital interactions (isocontour value = 0.05) between [L_2_]^2–^ and [In_2_H_2_]^2+^ in the singlet state of **3-H**, associated energies Δ*E* in kcal/mol, and eigenvalues ν in a.u. Red color shows charge outflow, whereas blue shows charge density accumulation.

Finally, the reactivity of **3-H** was investigated. Guided by structural and computational data suggesting a nucleophilic In­(II)–H moiety, its behavior toward a range of electrophiles was examined. It is important to note that these reactions were run at small scale due to the limitations regarding the synthesis of **3-H**. First, reaction with N-bromosuccinimide in THF-*d*_8_ at room temperature (Scheme 3A) resulted in dibromide dimer **3-Br** (88%) together with quantitative formation of succinimide (see Figures S6 and S7), which suggests direct hydride attack on the brominating agent. In a similar reaction, treatment with methyl iodide (Scheme 3B) afforded iodide derivative **3-I** (78%) along with methane (identified by ^1^H NMR at δ_H_ = 0.16 ppm in C_6_D_6_, see Figures S8 and S9), again consistent with nucleophilic substitution by the In–H bond. Notably, in both cases, the In–In bond remained intact even under excess methyl iodide, underscoring the robustness of the diindane and the In–In bond. Reaction of **3-H** with pentafluoropyridine (Scheme 3C, see Figures S10 and S11) produced 2,3,5,6-tetrafluoropyridine in quantitative yield together with the corresponding difluoro diindane species **3-F** (98% isolated yield), which displays a clear ^19^F NMR resonance at δ_F_ = 189.37 ppm in C_6_D_6_ and constitutes a rare example of an indium fluoride species in oxidation state lower than +3 (see the inset in Scheme 3 for the SC-XRD structure). Interestingly, catalytic hydrodefluorination was attempted using 3.6 mol % of **3-H** in the presence of PhSiH_3_ but afforded modest yields of 2,3,5,6-tetrafluoropyridine, likely due to catalyst degradation (see Figures S12–S15).^65^ Exposure of **3-H** to excess dimethyl disulfide (Scheme 3D) under analogous conditions yielded compound **3-SMe** (>95%). This species is unstable and decomposes within hours, which made in situ characterization necessary immediately after reaction completion (see Figure S81).^65^ In addition, **3-H** undergoes acid–base reactivity with pyridinium bromide in C_6_D_6_ (Scheme 3E), resulting in pyridine (>95%), **3-Br** (>95%), and H_2_, which can be clearly detected by ^1^H NMR (see Figures S18–S20). Finally, **3-H** acts as a reducing agent when mixed with Bi dibromide derivative **4** (Scheme 3F), resulting in the formation of bismuthinidene species **5** in 58% yield along with **3-Br** (55%) and H_2_, which was detected by ^1^H NMR (δ_H_ = 4.47 ppm in C_6_D_6_). Together, these results establish **3-H** as a *bona fide* nucleophilic In hydride capable of engaging a diverse set of reactions while preserving its unusual dimeric structure and the In–In bond. Additional reactivity with **3-H** was explored with other substrates; however, these examples yielded complex mixtures (see Table S1).

**Scheme 3:**
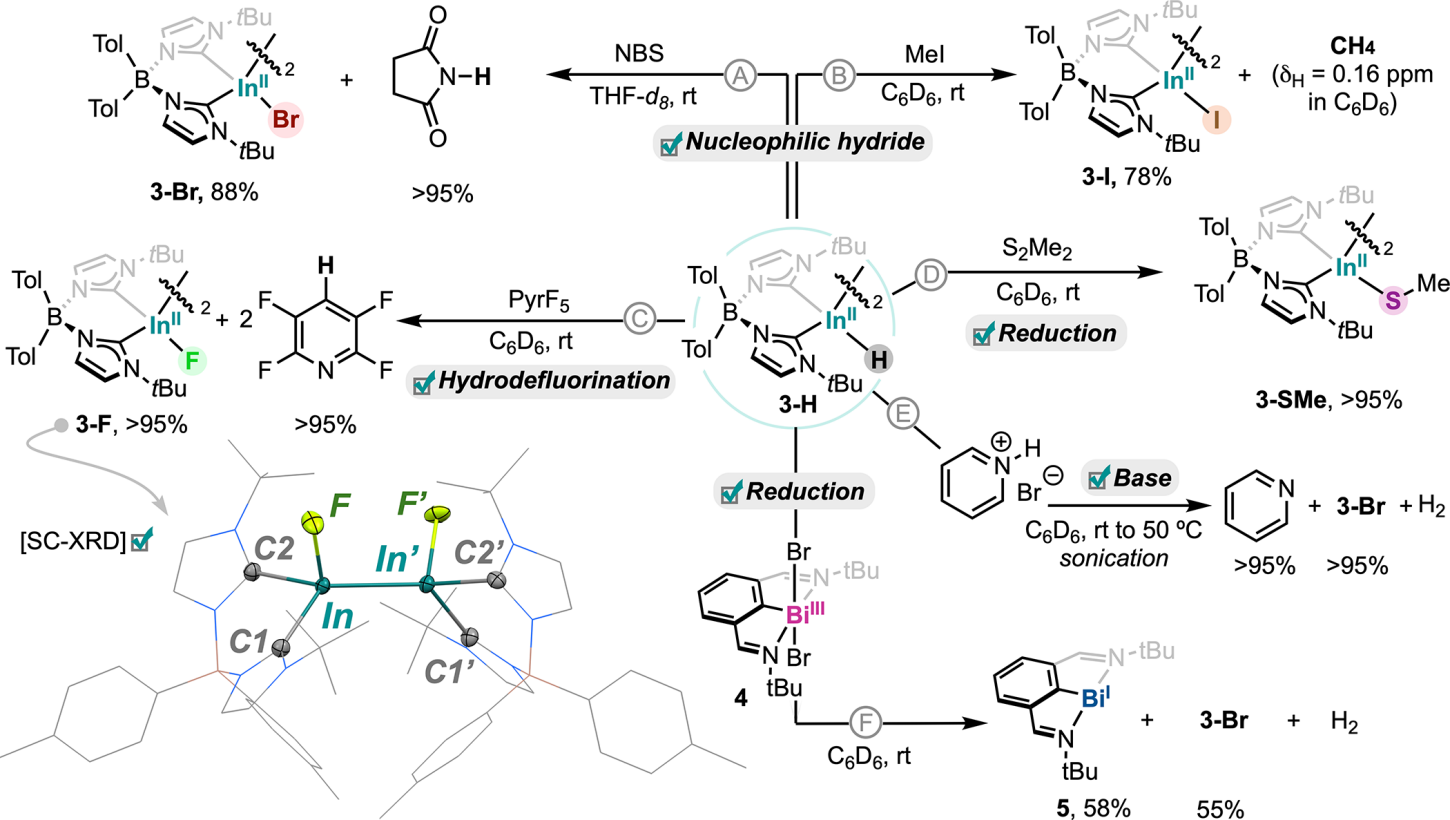
Stoichiometric reactivity of In­(II) Hydride **3-H** with electrophilic reagents (N-bromosuccinimide, MeI), pentafluoropyridine, dimethyl disulfide, and pyridinium bromide, as well as reduction of bismuth dibromide species **4.** ^a^

a See Supporting Information for detailed reaction conditions. Inset: molecular structure of **3-F**. H-atoms are omitted for clarity; ellipsoids displayed at 50% probability.

## 3 Conclusion

In summary, In­(II) hydride **3-H**, supported by a bis­(NHC)­borate ligand scaffold, demonstrates that the interplay of steric design, noncovalent interactions, and zwitterionic charge distribution can stabilize low-valent heavy main group hydride species. Beyond its robustness, **3-H** shows a nucleophilic hydride that engages in halogenation, alkylation, hydrodefluorination, chalcogenolysis, and reduction, all while preserving its In–In bond. Complementary computational analyses corroborate the experimental data, unveiling a strong covalent In–In interaction and a highly polarized In–H bond, both of them shielded by substantial steric protection and stabilized by noncovalent interactions. Together, these features rationalize the remarkable stability and nucleophilic behavior of this otherwise elusive low oxidation state hydride. By uncovering the design principles that govern both stability and reactivity in this rare class of compounds, our work not only delivers a blueprint for genuine In hydride chemistry below the +3 oxidation state but also redefines heavier group 13 hydrides as viable platforms for bond activation and small-molecule functionalization. In doing so, it establishes the conceptual and structural foundations for extending main group hydride chemistry into oxidation states that were once considered inaccessible.

## Supplementary Material



## Notes

Complete experimental details, NMR, computational details, and crystallographic data (PDF)
