# Supplementary material for: A Crystalline In(II) Hydride
Source: J Am Chem Soc. 2026 Jan 30;148(5):5783–92. doi: 10.1021/jacs.5c22490 (PMC12903871; doi:10.1021/jacs.5c22490)
Supplement: Supplementary file 1 [file ja5c22490_si_001.pdf]

Supporting Information

A Crystalline In(II) Hydride

Olympia Mouriki,[a] Graham J. Tizzard,[b] Simon J. Coles,[b] Diego M. Andrada, [c] Oriol Planas[a,*]

[a]Department of Chemistry, Molecular Sciences Research Hub, Imperial College London, 82 Wood Lane, Shepherds Bush, London, W12 0BZ, UK.

[b]EPSRC National Crystallography Service, School of Chemistry and Chemical Engineering, University of Southampton, Southampton SO17 1BJ, UK

[c]General and Inorganic Chemistry Department, University of Saarland, Campus C4.1 66123, Saarbrücken, Germany

*corresponding author email: o.planas@imperial.ac.uk

TABLE OF CONTENTS

1 General Methods S3
2 Synthesis of In(III) Complexes S4
2.1 Synthesis of In(III) Complex (**2-Br**) S4
2.2 Synthesis of In(III) Complex (**2-I**) S5
3 Synthesis of In(II) Complexes S6
3.1 Synthesis of In(II)-Br Complex (**3-Br**) S6
3.2 Synthesis of In(II)-I Complex (**3-I**) S7
3.3 Synthesis of In(II)-H Complex (**3-H**) S8
4 Reactivity studies of 3-H S12
4.1 Reactivity with N-Bromosuccinimide S12
4.2 Reactivity with Methyl Iodide S14
4.3 Reactivity with Pentafluoropyridine S16
4.4 Reactivity with Dimethyl Disulphide S21
4.5 Reactivity with Dibromo Bismuthine **4** S22
4.6 Reactivity with Pyridinium Bromide S24
4.7 Unsuccessful Reactivity Studies S26
5 Stability Studies of 3-H S27
5.1 Thermal Decomposition S27
5.2 Air Exposure S29
6 Computational Details S31
6.1 Benchmark S33
6.2 Computed FT-IR Spectra of **3-H** S34
6.3 Frontier Molecular Orbitals (FMO) S35
6.4 Natural Bond Orbitals (NBO) S36
6.5 Atoms in Molecules (AIM) S38
6.6 Topographical analysis of the Electron Localization Function S42
6.7 Non-Covalent Interaction Analysis S43
6.8 Energy Decomposition Analysis (EDA) S44
6.9 xyz Coordinates (in Å) and Energies (in Hartree) S48
7 Crystallographic Data S70
7.1 Single Crystal Structure Analysis of **2-Br** S70
7.2 Single Crystal Structure Analysis of **2-I** S81
7.3 Single Crystal Structure Analysis of **3-Br** S92
7.4 Single Crystal Structure Analysis of **3-H** S116
7.5 Single Crystal Structure Analysis of **3-F** S128
8 NMR Characterisation Data S154
8.1 NMR characterisation of In(III) complexes **2-X** S154
8.2 NMR characterisation of diindium complexes **3-X** S167
9 References S205

1 General Methods

Unless otherwise stated, all manipulations were performed using standard Schlenk techniques under dry nitrogen (N_2) in oven-dried glassware or in N_2-filled MBraun Unilab SP glovebox. Celite was dried at 150 °C under vacuum for 3 days and stored in the glovebox prior to use. Diethyl ether, toluene, tetrahydrofuran, hexane, pentane and dichloromethane were collected from an Inert PureSolv MD4 solvent purification system under N_2 and stored over activated 4Å MS. Anhydrous benzene was distilled from Na-benzophenone. Deuterated solvents were stored over activated 4Å MS under N_2 prior to use. Commercially available indium(III) bromide and indium(III) iodide were stored inside a N_2-filled glovebox and used as received. Pentafluoropyridine was stored over activated 4Å molecular sieves. N-bromosuccinimide, methyl disulfide, methyl iodide and K-selectride were obtained from commercial sources and utilized without further purification. Imidazolium salts **1-Cl** and **1-PF$_6$** were prepared following previously reported by our group.[1]

IR spectra were recorded using a Cary630 spectrometer (placed with in an MBraun glovebox) spectrometer under inert conditions with 16 scans on a diamond ATR unit. NMR spectra were recorded using 400 MHz AVANCE III HD nanobay console running at 296 K and 500 MHz AVANCE III HD single bay console running at 298 K, unless otherwise stated. The magnets have a field of 9.4 T and 11.75 T, respectively. The probes are BBO (broadband observe) and prodigy cryoprobe, respectively. All equipment is Bruker Biospin using Topspin version is 3.8.0. ^{1}H NMR spectra (400 MHz) were referenced to the residual protons of the corresponding deuterated solvent:[6] $CDCl_3$ (δ = 7.26 ppm), C_6D_6 (δ = 7.16 ppm) or THF-d_8 (δ = 3.58 ppm). $^{13}C\{^{1}H\}$ NMR spectra (101 MHz) were referenced internally to the D-coupled ^{13}C resonances of the corresponding deuterated solvent:[6] $CDCl_3$ (δ = 77.16 ppm) or C_6D_6 (δ = 128.06 ppm). ^{13}C and ^{11}B spectra are ^{1}H decoupled. Chemical shifts (δ) are given in ppm, relative to deuterated solvent residual peak, and coupling constants (*J*) provided in Hz. NMR data are represented according to the following structure: chemical shift, multiplicity (s = singlet, d = doublet, dd = doublet of doublets, t = triplet, q = quartet, m = multiplet, bs = broad singlet), coupling constants (Hz), integration and atom (labelled according to the depicted structure).

Atmospheric Solid Probe Analysis coupled with Capillary Heated Electrospray Ionisation (ASAP-HESI) was used to acquire mass spectra at the University College London Chemistry Mass Spectrometry Facility. The generated gas phase ions were measured using the Exactive Plus mass spectrometer. HESI parameters were the auxiliary gas to 5 and the sheath and sweep gases to 0 and the auxiliary gas heater to 400 °C and typical operating temperatures were between 100 °C and 450 °C. The discharge voltage was set to between 3.5 and 4.0 kV to give a stable background ion signal would depend on the structure of analyte. Elemental analysis was performed at the LMU using a ThermoFlash 2000 instrument.

2 Synthesis of In(III) Complexes

2.1 Synthesis of In(III) Complex (2-Br)

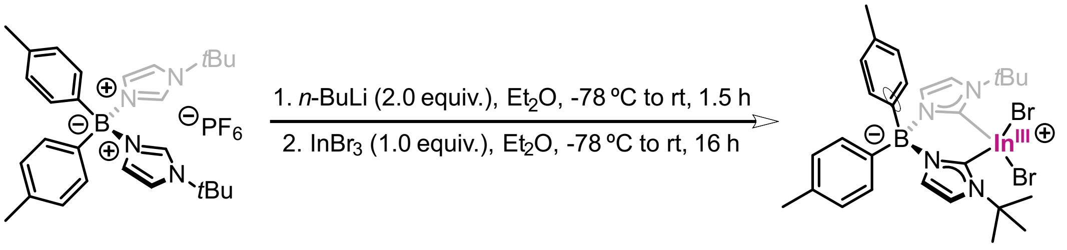

1-PF$_6$, 72% **2-Br**, 94%

Under inert Schlenk conditions, **1-PF$_6$** (3.00 g, 1.0 equiv., 5.12 mmol) was dissolved in dry diethyl ether (60 mL) to form a yellow solution. n-BuLi (4.09 ml, 2.5 M in hexane, 2.0 equiv., 10.2 mmol) was then added dropwise at -78 ºC, allowed to warm to room temperature and stirred for 1.5 hours. In(III)Br$_3$ (1.81 g, 1.0 equiv., 5.12 mmol) was then dissolved in dry diethyl ether (15 mL) and added dropwise at -78 ºC to the stirring solution and then allowed to warm to room temperature. The solution initially turned more yellow and then gradually became colourless as it warmed to room temperature. After stirring the solution at room temperature overnight, the solvent was removed *in vacuo*. The product was extracted away from salts through a frit and a celite plug using toluene, producing a clear colourless solution. The solvent was then removed *in vacuo* from the air stable product, washed with hexanes and then dried to produce **2-Br** as a white solid (3.42 g, 94 %). Crystals suitable for XRD were obtained by dissolving the sparingly soluble product in hexane and the slow evaporation of this solution.

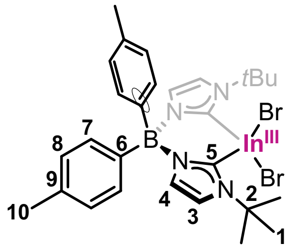

^{1}H NMR: δ$_H$ (400 MHz, CDCl$_3$) 7.18 (d, $^{3}J_{H-H}$ = 1.8 Hz, 2H, H-3), 7.02 (d, $^{3}J_{H-H}$ = 7.6 Hz, 4H, H-7), 6.93 (d, $^{3}J_{H-H}$ = 1.8 Hz, 2H, H-4), 6.59 (d, $^{3}J_{H-H}$ = 7.6 Hz, 4H, H-8), 2.31 (s, 6H, H-10), 1.82 (s, 18H, H-1).

^{13}C NMR: δ$_C$ (101 MHz, CDCl$_3$) 164.5 (C-5), 146.8 (C-6), 136.2 (s, C-9), 133.4 (s, C-8), 128.8 (s, C-4), 128.4 (s, C-7), 117.6 (s, C-3), 59.3 (s, C-2), 31.8 (s, C-1), 21.4 (s, C-10).

^{11}B NMR: δ$_B$ (128 MHz, C$_6$D$_6$) 2.47 (bs).

HRMS (ASAP-pos, m/z): calc'd for $C_{28}H_{36}BBrInN_4$ [M-Br]$^+$ 633.1250; found 633.1247.

CHN: $C_{28}H_{36}BBr_2InN_4$ calcd. C 47.10, H 5.08, N 7.85 %; exp. C 47.37, H 5.11, N 7.89 %.

2.2 Synthesis of In(III) Complex (2-I)

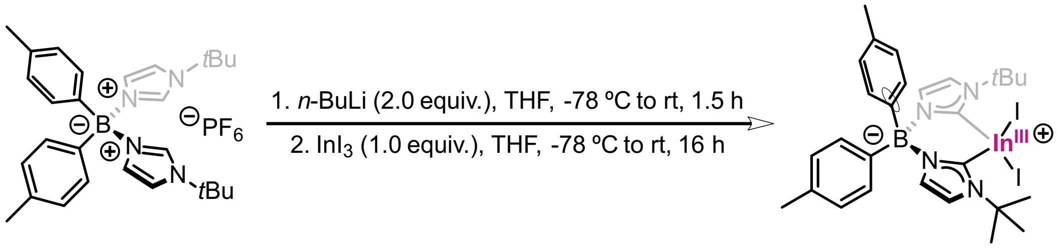

1-PF$_6$, 72% **2-I,** 11%

Under inert Schlenk conditions **1-PF$_6$** (0.74 g, 1.0 equiv., 1.26 mmol) was suspended in dry tetrahydrofuran (THF) (22 mL) to form a bright yellow solution. n-BuLi in hexane (1.01 ml, 2.5 M in hexane, 2.0 equiv., 2.51 mmol) was then added dropwise at -78 ºC, allowed to warm to room temperature and stirred for 1.5 hours. In(III)I$_3$ (0.62 g, 1.26 mmol) was then dissolved in dry THF (15 mL) and added dropwise at to the stirring solution at -78 ºC and then allowed to warm to room temperature. The solution initially turned more colourless and then gradually became very bright yellow. This solution gradually turned a slightly turbid orange. After stirring the solution at room temperature overnight, the solvent was removed *in vacuo*. The product was extracted away from salts through a frit and a pad of celite using toluene, producing a clear orange solution. On exposure to air this solution turned cloudy* and was therefore allowed to sit and refiltered twice through celite to produce a clear orange solution. The solvent was then removed *in vacuo.* This was washed with hexane and the partially ether soluble product was dissolved in ether and allowed to slow evaporate. This yielded colourless crystals which were washed with cold dry ether and then dried to afford the air stable product **2-I** (0.11 mg, 11 %).

Note*: Repeating the whole procedure under inert conditions did not improve results.

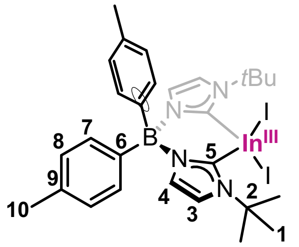

^{1}H NMR: δ_H (400 MHz, C_6D_6) 7.15 (d, $^{3}J_{H-H}$ = 7.6 Hz, 4H, H-7), 6.93 (d, $^{3}J_{H-H}$ = 1.8 Hz, 2H, H-4), 6.89 (d, $^{3}J_{H-H}$ = 7.6 Hz, 4H, H-8), 6.45 (d, $^{3}J_{H-H}$ = 1.8 Hz, 2H, H-3), 2.24 (s, 6H, H-10), 1.49 (s, 18H, H-1).

^{13}C NMR: δ_C (101 MHz, C_6D_6) 163.7 (C-5), 147.8 (C-6), 136.4 (s, C-9), 133.9 (s, C-8), 128.9 (s, C-7), 128.8 (s, C-4), 117.6 (s, C-3), 59.6 (s, C-2), 31.9 (s, C-1), 21.4 (s, C-10).

^{11}B NMR: δ_B (128 MHz, C_6D_6): 2.88 (s).

HRMS (ASAP-pos, m/z): calc'd for $C_{28}H_{36}BI_2InN_4$ $[M+H]^+$ 809.0234; found 809.0245.

CHN: $C_{28}H_{36}BBr_2InN_4$ calcd. C 41.62, H 4.49, N 6.93 %; exp. C 41.67, H 4.23, N 6.85 %.

3 Synthesis of In(II) Complexes

3.1 Synthesis of In(II)-Br Complex (3-Br)

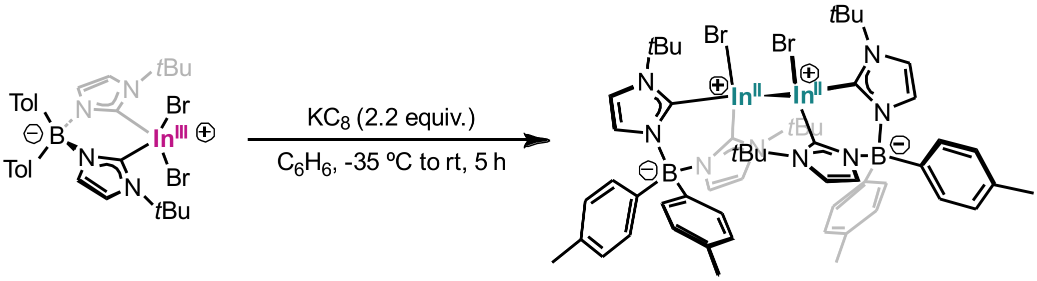

2-Br **3-Br,** 72 %

A Schlenk was charged with KC_8 (0.42 g, 2.2 equiv., 1.40 mmol) suspended in C_6H_6 (5 mL) and frozen at -35 ºC. A solution of **2-Br** (1.00 g, 1.0 equiv., 3.11 mmol) in C_6H_6 (5 mL) was then prepared and suspended over the frozen KC_8 solution. This was manually stirred and allowed to warm to room temperature, then stirring for a further 5 hours. The solution was filtered using a filter cannula to afford a clear, golden solution. The solvent was removed *in vacuo* to produce a pale brown solid. This solid was sonicated and vigorously stirred in hexane for a minimum of 2 hours. This suspension was then filtered through a frit, washing with hexane. The solid was dried to produce **3-Br** as a white powder (0.64 g, 72 %). Crystals suitable for XRD were obtained by ether and pentane bilayer.

<u>Note*</u>: It is advised that aliquots are obtained to assess reaction completion as it has been observed that different synthesised batches of KC_8 can afford varying reaction times (observed completion range = 2 - 8 h).

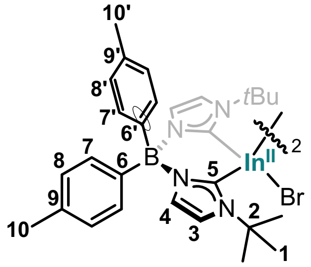

^{1}H NMR: δ_H (400 MHz, C_6D_6) 7.29 (d, $^3J_{H\text{-}H}$ = 7.7 Hz, 4H, H-7’), 7.12 (d, $^3J_{H\text{-}H}$ = 7.7 Hz, 4H, H-8’), 7.04 (d, $^3J_{H\text{-}H}$ = 7.7 Hz, 4H, H-8), 6.86 (d, $^3J_{H\text{-}H}$ = 1.8 Hz, 4H, H-4), 6.84 (d, $^3J_{H\text{-}H}$ = 7.7 Hz, 4H, H-7), 6.54 (d, $^3J_{H\text{-}H}$ = 1.8 Hz, 4H, H-3), 2.23 (s, 6H, H-10’), 2.14 (s, 6H, H-10), 1.51 (s, 36H, H-1).

^{13}C NMR: δ_C (101 MHz, C_6D_6) 170.7 (s, C-5), 149.4 (bs, C-6/6’), 136.0 (s, C-9), 135.9 (s, C-7’), 134.7 (s, C-9’), 132.2 (s, C-7), 128.6 (s, C-8’), 128.5 (s, C-8), 128.4 (s, C-4), 117.5 (s, C-3), 58.7 (s, C-2), 31.8 (s, C-1), 21.6 (s, C-10), 21.4 (s, C-10’).

^{11}B NMR: δ_B (128 MHz, C_6D_6) 1.45 (bs).

HRMS (ESI-pos, m/z): calc’d for $C_{56}H_{72}B_2BrIn_2N_8$+CH_4O [M-Br+CH_3OH]$^+$ 1219.3984; found 1219.4096.

3.2 Synthesis of In(II)-I Complex (3-I)

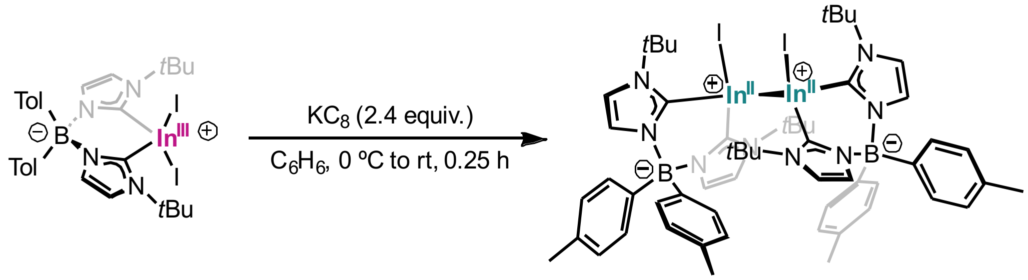

2-I **3-I,** 68 %

A Schlenk was charged with KC_8 (7.6 mg, 2.4 equiv., 5.6 µmol) suspended in C_6D_6 (0.3 mL) and frozen at -35 ºC. A solution of **2-I** (18.9 mg, 1.0 equiv., 2.3 µmol) in C_6D_6 (0.3 mL) was then prepared and suspended over the frozen KC_8 solution. The solution was manually stirred and allowed to melt and warm to room temperature, stirring for 0.25 hours*. The solution was then filtered using a PTFE syringe filter and the solvent was removed *in vacuo* to produce **3-I** as a white solid (3.6 mg, 68 %). The sample obtained contains trace amounts of ligand (see Figure S58).

<u>Note*</u>: It is advised that aliquots are obtained to assess reaction completion as it has been observed that different batches of KC_8 can afford varying times (observed completion range = 2 - 8 h).

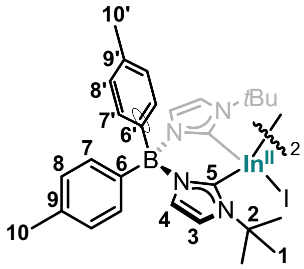

^{1}H NMR: δ_H (400 MHz, C_6D_6, 298 K) 7.38 (d, $^3J_{H-H}$ = 7.9 Hz, 4H, H-7), 7.14 (d, $^3J_{H-H}$ = 7.9 Hz, 4H, H-8), 7.05 (d, $^3J_{H-H}$ = 7.9 Hz, 4H, H-8'), 6.94 (bs, 4H, H-4), 6.88 (d, $^3J_{H-H}$ = 7.9 Hz, 4H, H-7'), 6.52 (bs, 4H, H-3), 2.22 (s, 6H, H-10), 2.18 (s, 6H, H-10'), 1.49 (bs, 36H, H-1).

^{13}C NMR: δ_C (101 MHz, C_6D_6, 298 K) 169.2 (s, C-5), 149.8 (bs, C-6/6'), 136.0 (s, C-9), 135.4 (s, C-7), 134.9 (s, C-9'), 132.5 (s, C-7'), 129.0 (s, C-8'), 128.8 (s, C-4), 128.6 (s, C-8), 117.7 (s, C-3), 58.8 (s, C-2), 32.1 (s, C-1), 21.4 (s, C-10/10').

^{11}B NMR: δ_B (128 MHz, C_6D_6, 298 K) -2.74 (bs).

HRMS (ESI-pos, m/z): calc'd for $C_{56}H_{72}B_2I_2In_2N_{8+}CH_4ONa$ $[M+CH_3OH+Na]^+$ 1417.2388; found 1417.2365.

3.3 Synthesis of In(II)-H Complex (3-H)

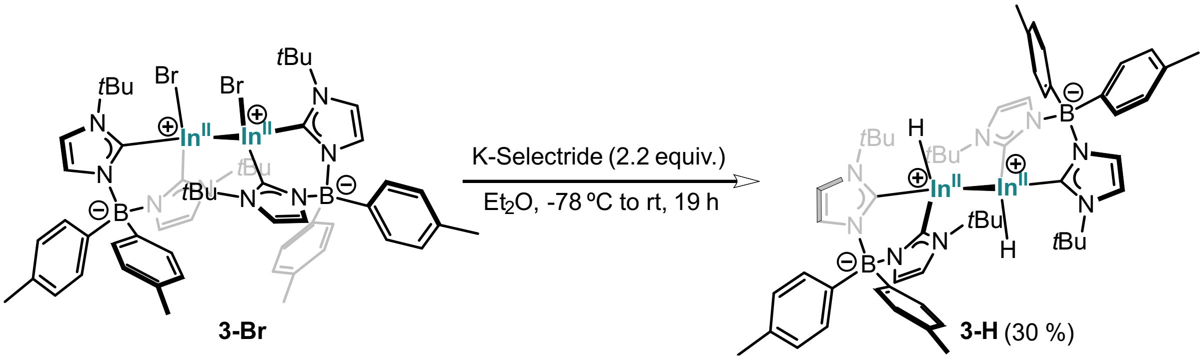

3-Br **3-H** (30 %)

In a reaction vial, **3-Br** (74.0 mg, 1.0 equiv., 58.3 mmol) was suspended in dry diethyl ether (1.2 mL). This was cooled to -78 ºC and K-Selectride (129.0 µL, 1M in THF, 2.2 equiv, 129.0 µmol) was added dropwise. The solution was allowed to warm to room temperature and stirred for 19 hours to afford a brown-grey suspension. The solvent was removed *in vacuo* and the solid dissolved in THF. Grey precipitates present were removed using a PTFE syringe filter to produce a brown-orange solution. The solvent was reduced *in vacuo* at 0 ºC to induce incipient crystallisation. After storing the saturated solution at -35 ºC overnight, clear crystals were produced. These were washed with THF (*ca.* 4 x 0.2 mL) at -95 ºC and dried* in *vacuo* to yield **3-H** as a white solid (30.9 mg, 30 %).

Note*: It is advised that crystals are not fully dried due to a high level of subsequent crystal packing affecting the extent of solubility when attempting to redissolve for reactivity.

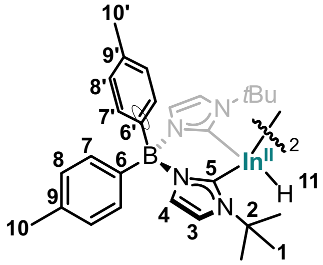

^{1}H NMR: δ_H (400 MHz, C_6D_6) 7.50 (d, $^{3}J_{H\text{-}H}$ = 7.9 Hz, 4H, H-7), 7.17 (d, $^{3}J_{H\text{-}H}$ = 7.9 Hz, 4H, H-8), 7.15 (d, $^{3}J_{H\text{-}H}$ = 7.9 Hz, 4H, H-8'), 7.07 (d, $^{3}J_{H\text{-}H}$ = 7.8 Hz, 4H, H-7'), 6.96 (d, $^{3}J_{H\text{-}H}$ = 1.8 Hz, 4H, H-4), 6.57 (d, $^{3}J_{H\text{-}H}$ = 1.7 Hz, 4H, H-3), 6.27 (bs, 2H, H-11), 2.33 (s, 6H, H-10'), 2.25 (s, 6H, H-10), 1.32 (s, 36H, H-1).

^{13}C NMR: δ_C (101 MHz, C_6D_6, 298 K) 176.1 (s, C-5), 149.1 (C-6/6'), 136.2 (s, C-7), 135.5 (s, C-9), 134.3 (s, C-9'), 133.1 (s, C-7'), 128.6 (s, C-8), 128.4 (s, C-8), 127.4 (s, C-4), 116.1 (s, C-3), 57.4 (s, C-2), 31.1 (s, C-1), 21.7 (s, C-10'), 21.4 (s, C-10).

^{11}B NMR: δ_B (128 MHz, C_6D_6) -12.87 (bs).

HRMS (ESI-pos, m/z): calc'd for $C_{56}H_{74}B_2In_2N_8$+H_3O $[M+H_2O+H]^+$ 1129.4479; found 1129.4297, $[M+1/2M]^+$ 555.2145; found 555.2141.
CHN: $C_{56}H_{74}B_2In_2N_8$ calcd. C 60.57, H 6.72, N 10.09 %; exp. C 60.91, H 6.82, N 9.56 %.

FT-IR (ATR): υ 1640 cm^{-1} (br, In-H).

3.3.1 Selected Spectroscopic Data of 3-H

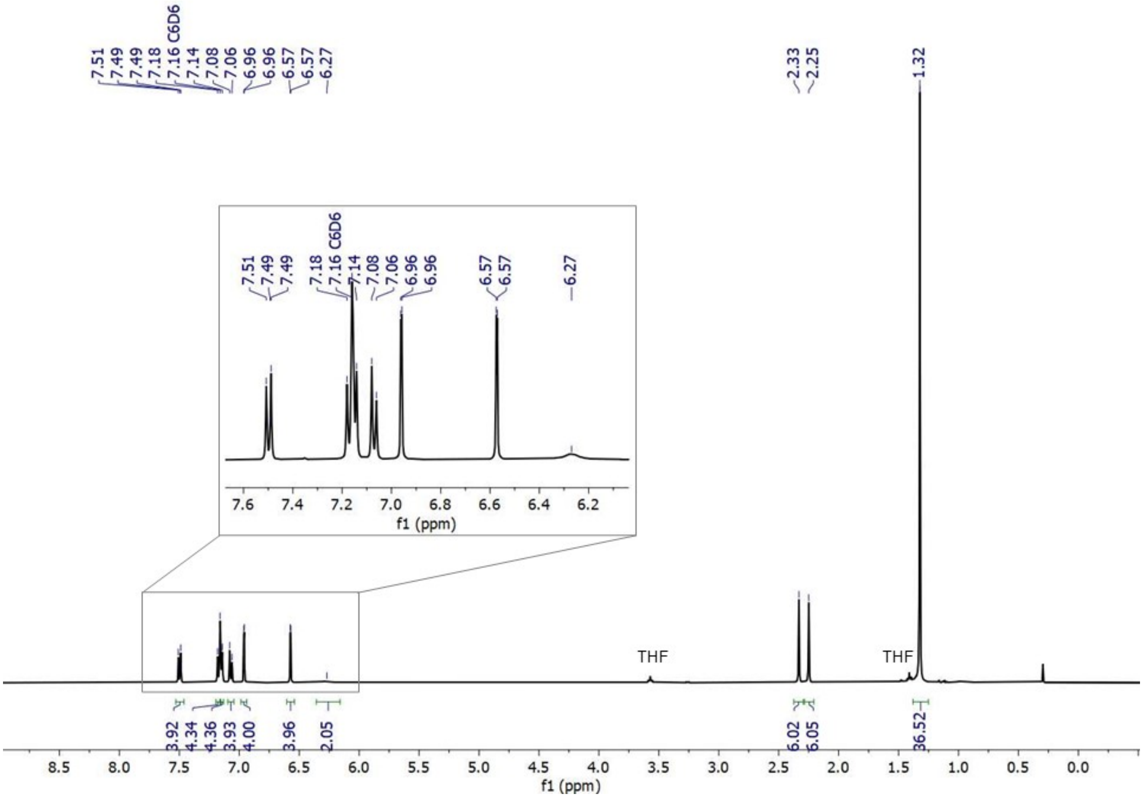

Figure S1. ^{1}H NMR (C_6D_6, 400 MHz) spectrum of **3-H**.

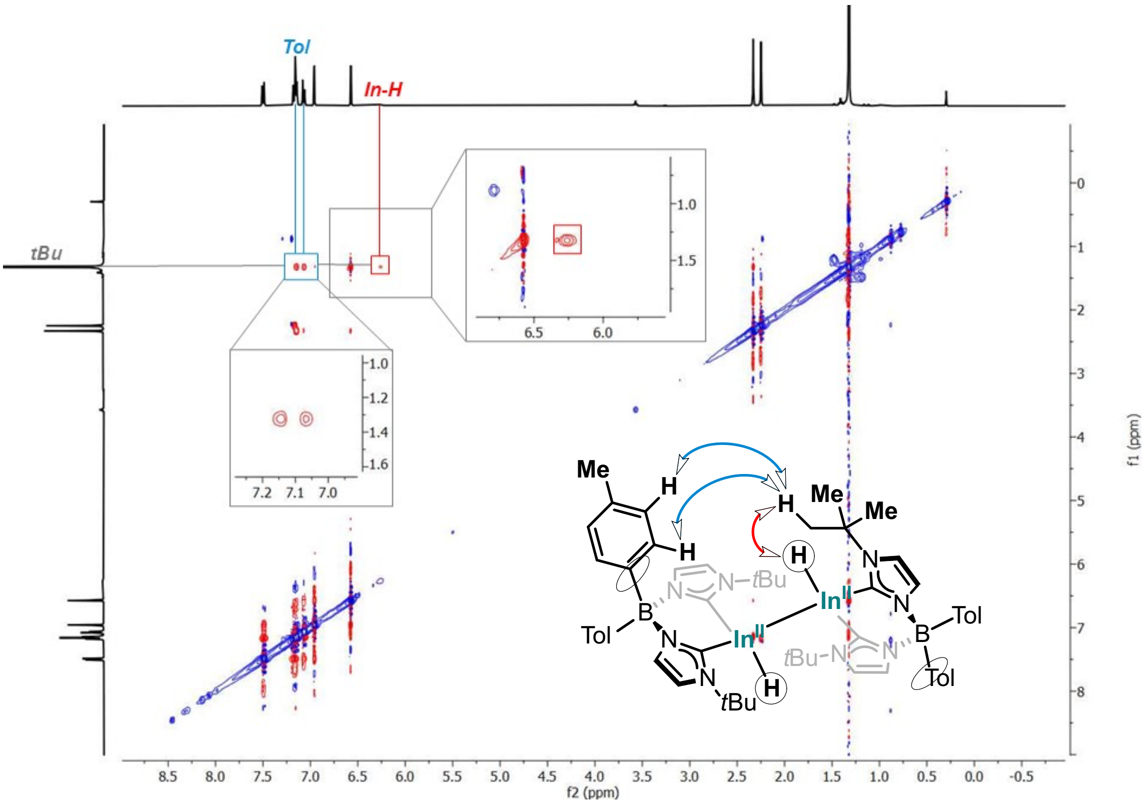

Figure S2. ^{1}H-^{1}H NOESY (C_6D_6, 400 MHz) spectrum of **3-H**.

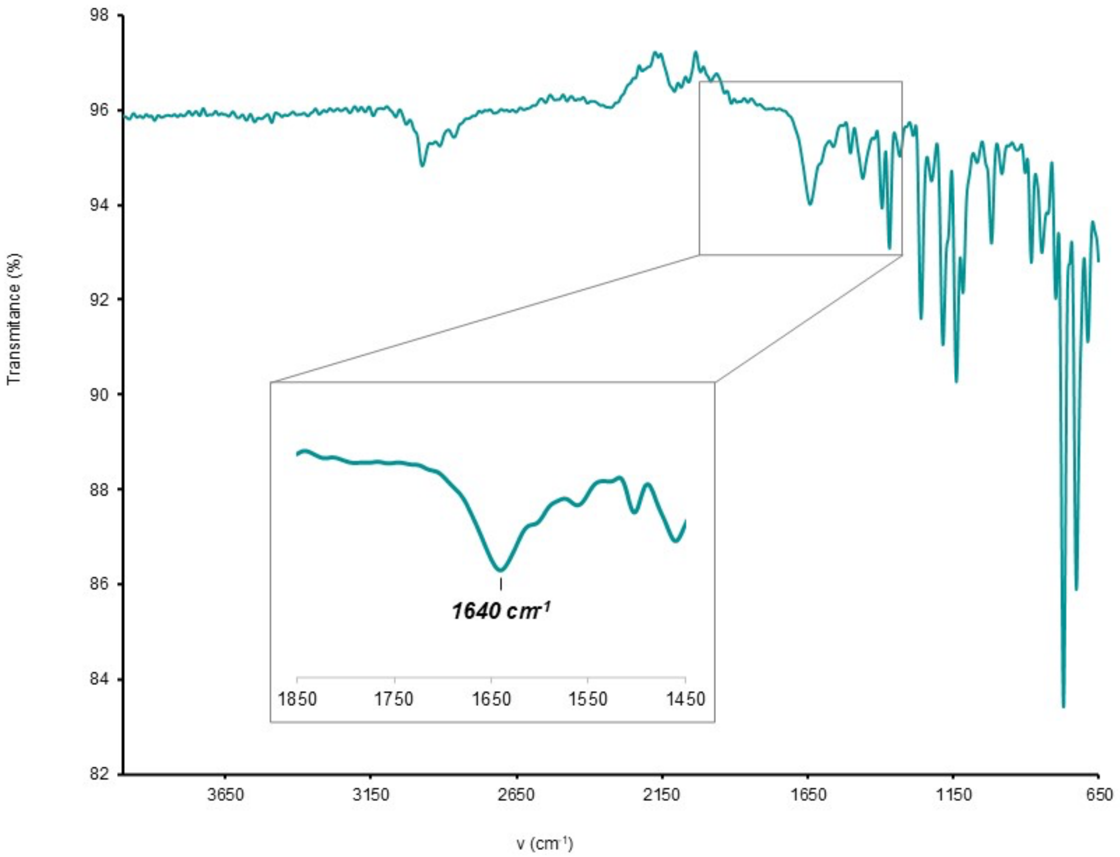

Figure S3. FT-IR spectrum of **3-H**.

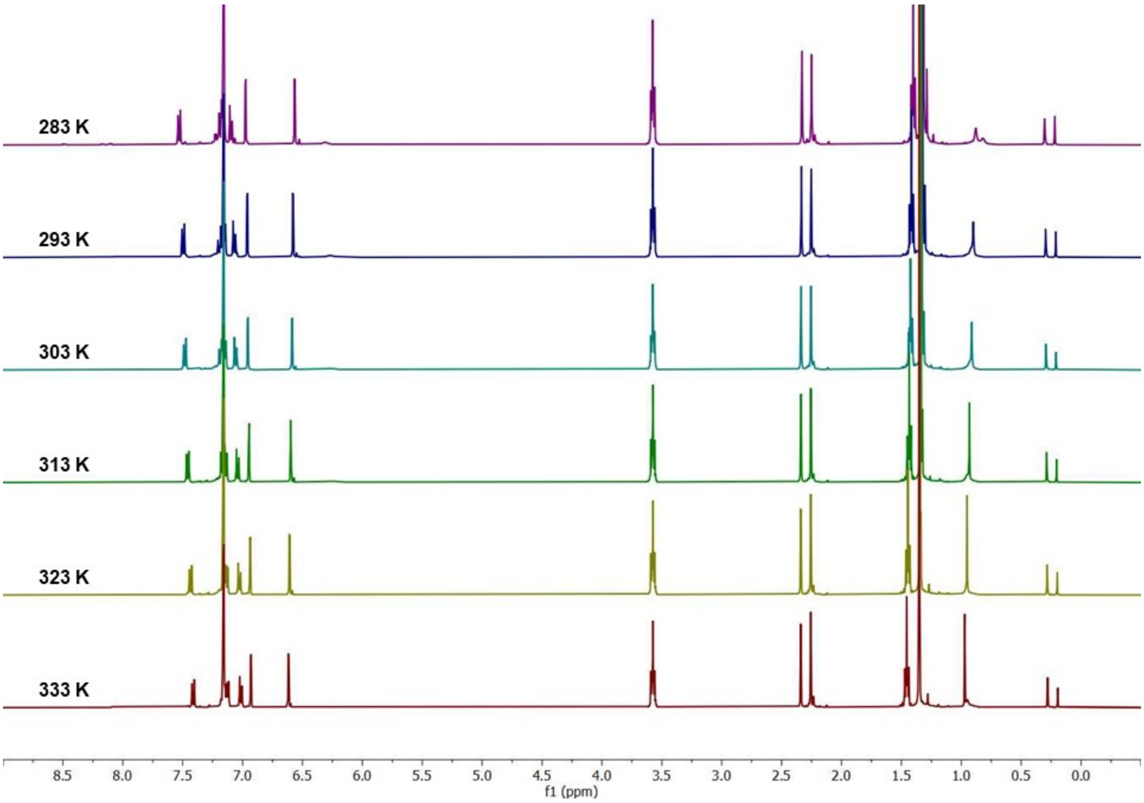

Figure S4. ^{1}H VT NMR (C_6D_6, 500 MHz, 283 – 333 K) spectrum of **3-H**.

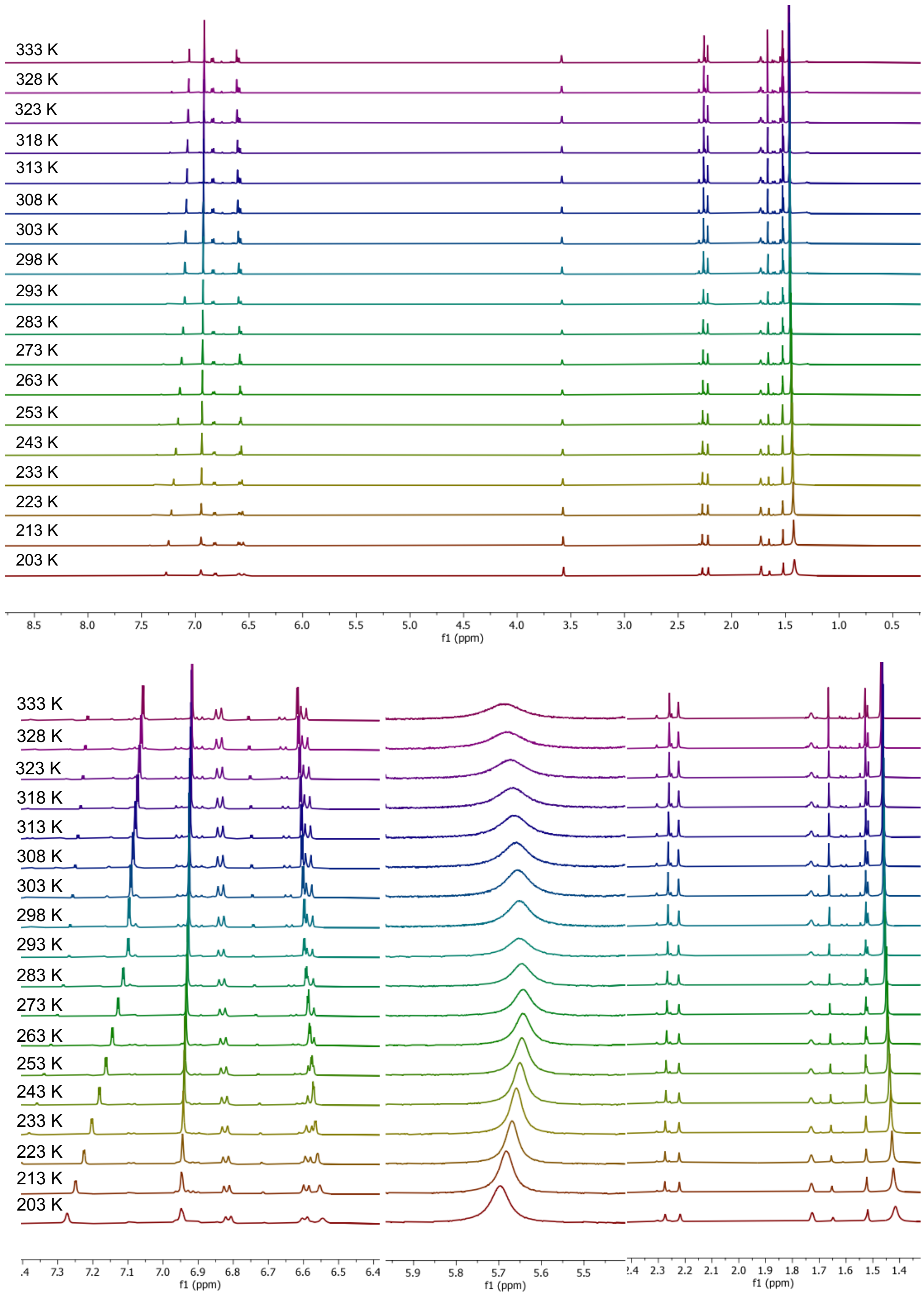

Figure S5. ^{1}H VT NMR (THF-d_8, 500 MHz, 203 – 333 K) spectrum of **3-H**. Top: full spectrum, bottom: selected zoomed-in areas.

4 Reactivity studies of 3-H

4.1 Reactivity with N-Bromosuccinimide

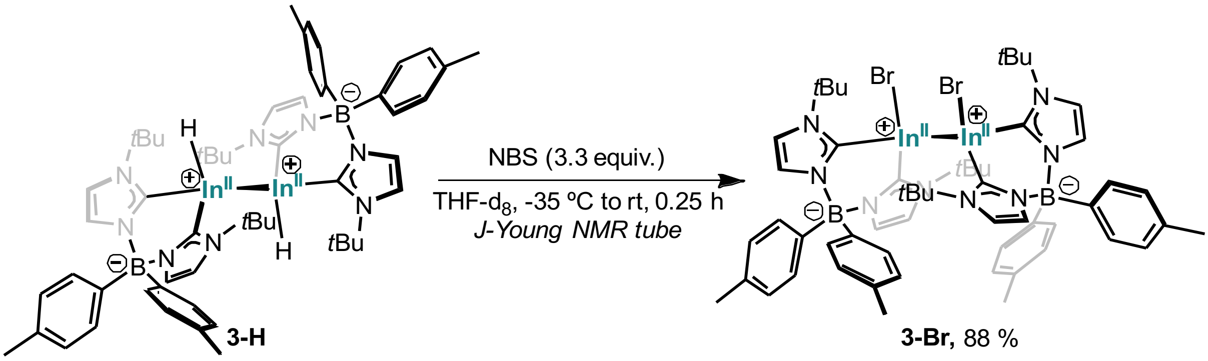

A vial was prepared with a solution of In(II) complex **3-H** (6.8 mg, 1.0 equiv., 6.2 µmol) in THF-d^8 (0.30 mL) and cooled to -35 ºC. NBS (3.6 mg, 3.3 equiv., 20.0 µmol) was dissolved in THF-d^8 (0.20 mL) precooled to -35 ºC and the two cold solutions were quickly mixed and transferred into a J-Young NMR tube. Hexane (2.0 µL, 2.5 equiv., 15 µmol) internal standard was added. The ^{1}H NMR was obtained 0.25 h later, showing the formation of **3-Br** (88 %) and succinimide (>95%). The crude ^{1}H NMR spectrum is shown in Figure S6 and stacked spectra are compared in Figure S7.

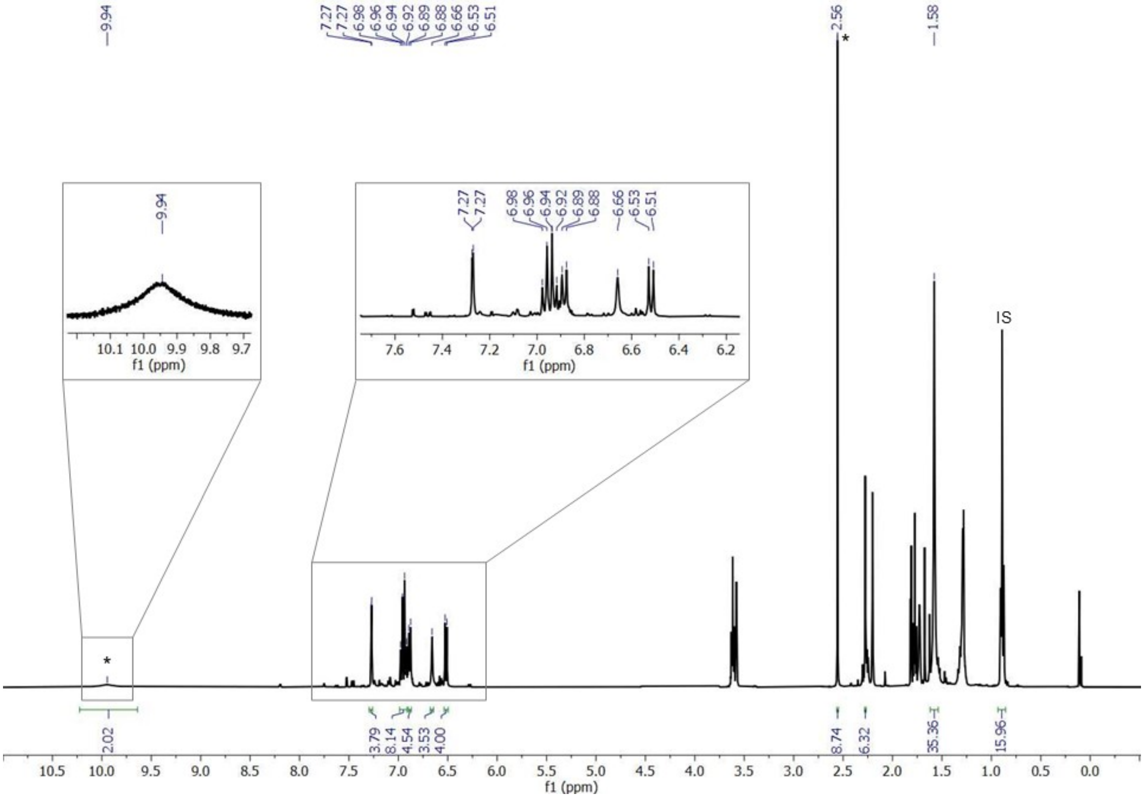

Figure S6. ^{1}H NMR (THF-d_8, 400 MHz) spectrum of **3-H** reactivity with N-bromosuccinimide to afford **3-Br** and succinimide (*) (IS = hexane internal standard).

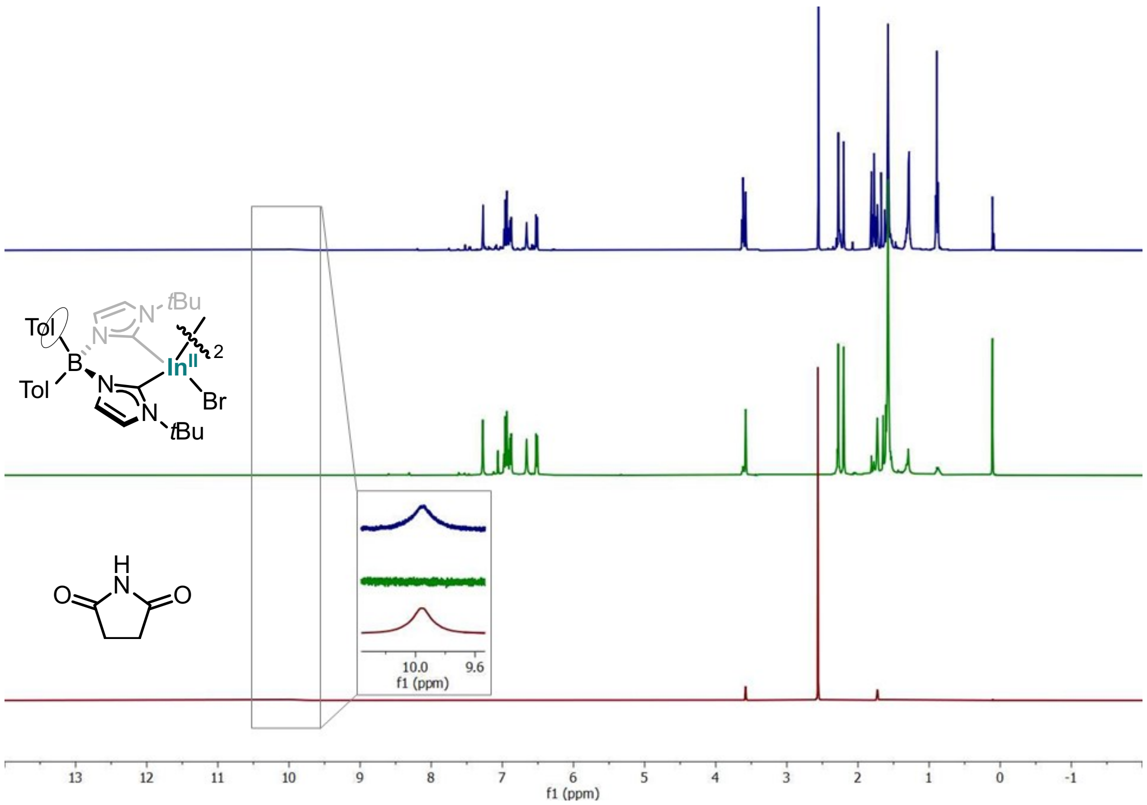

Figure S7. ^{1}H NMR stacked spectra (THF-d_8, 400 MHz) of the crude reaction of **3-H** with NBS (top, blue), isolated **3-Br** (middle, green) and commercial succinimide (bottom, red).

4.2 Reactivity with Methyl Iodide

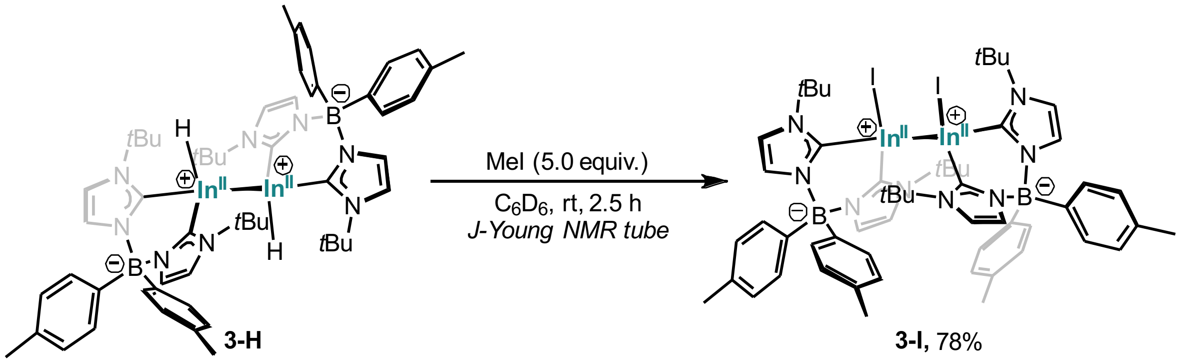

A J-Young NMR tube was prepared with a solution of In(II) complex **3-H** (8.1 mg, 1.0 equiv., 7.3 µmol) and pentane (4.0 µL, 4.8 equiv., 35 µmol) internal standard in C_6D_6 (0.50 mL). Following this, methyl iodide (2.4 µL, 5.0 equiv., 36 µmol) was added to the solution at room temperature. The ^{1}H NMR showed the reaction was complete after 2.5 h to afford **3-I** (78 %) and methane (detected at δ_H = 0.16 ppm, see Figure S8 and Figure S9).

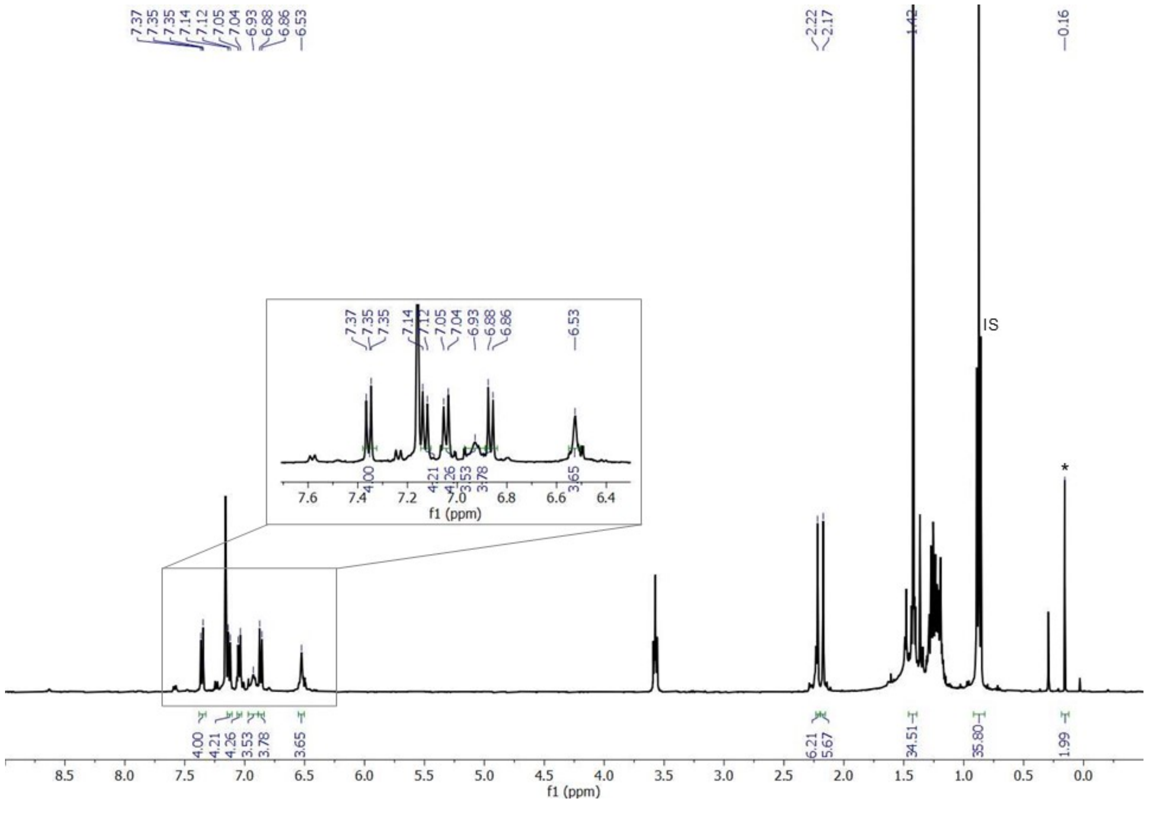

Figure S8. ^{1}H NMR spectrum (C_6D_6, 400 MHz) of **3-H** reactivity with methyl iodide to afford **3-I** and methane (*) (IS = pentane internal standard).

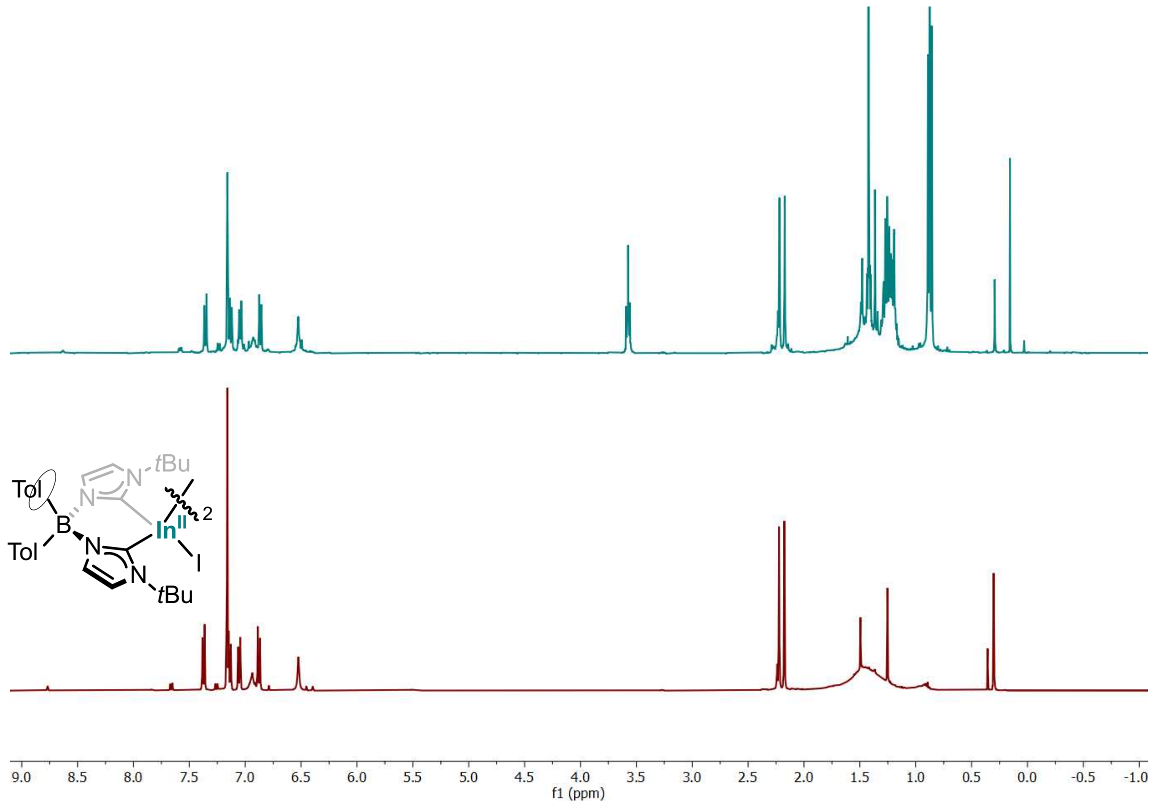

Figure S9. ^{1}H NMR stacked spectra (C_6D_6, 400 MHz) of the crude reaction (top, green) and **3-I** obtained from reduction of **2-I** with KC_8 (bottom, red, see Section 3.2).

4.3 Reactivity with Pentafluoropyridine

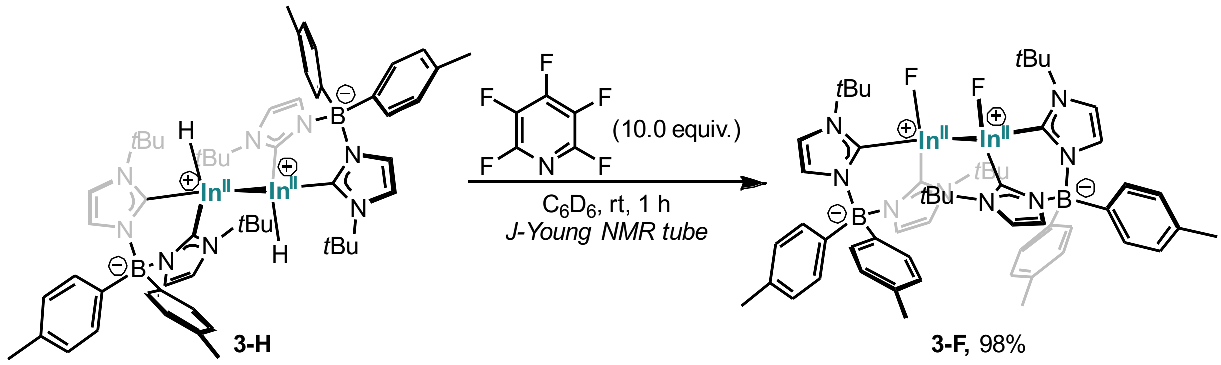

A -Young NMR tube was prepared with a solution of In(II) complex **3-H** (3.8 mg, 1.0 equiv., 3.4 µmol) and hexane (3.0 µL, 6.7 equiv., 23 µmol) internal standard in C_6D_6 (0.50 mL). Pentafluoropyridine (5.8 µL, 10.0 equiv., 34 µmol) was added to the solution and the reaction was left to sit at room temperature for 1 hour. A ^{1}H NMR was obtained and the resonances for **3-F** and 2,3,5,6-tetrafluoropyridine were identified to be present in a 1:1 ratio (Figure S10 and Figure S11). The volatiles were then removed *in vacuo* and the solid was washed with hexane and subsequently dried to afford pure **3-F** as a white solid (3.8 mg, 98%). Crystals were obtained *via* slow diffusion of pentane over a concentrated solution of the product in C_6D_6.

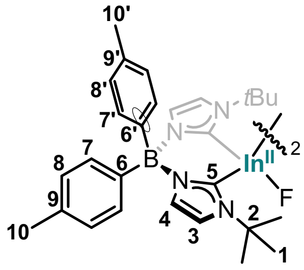

^{1}H NMR: δ_H (400 MHz, C_6D_6) 7.38 (d, $^3J_{H\text{-}H}$ = 7.9 Hz, 4H, H-7), 7.18 (d, $^3J_{H\text{-}H}$ = 7.6 Hz, 4H, H-8), 7.09 (d, $^3J_{H\text{-}H}$ = 7.6 Hz, 4H, H-8'), 6.92 (d, $^3J_{H\text{-}H}$ = 7.8 Hz, 4H, H-7'), 6.87 (d, $^3J_{H\text{-}H}$ = 1.8 Hz, 4H, H-4), 6.53 (d, $^3J_{H\text{-}H}$ = 1.8 Hz, 4H, H-3), 2.26 (s, 6H, H-10), 2.15 (s, 6H, H-10'), 1.52 (s, 36H, H-1).

^{13}C NMR: δ_C (101 MHz, C_6D_6) 174.1 (dd, $^2J_{C\text{-}F}$ = 10.6 Hz, $^3J_{C\text{-}F}$ = 2.9 Hz , C-5), 149.6 (bs, 6'), 148.8 (bs, 6), 136.4 (s, C-7'), 136.0 (s, C-9), 134.4 (s, C-9'), 132.5 (s, C-7'), 128.5 (s, C-8/8'), 127.6 (s, C-4), 116.7 (s, C-3), 58.4 (s, C-2), 31.4 (d, $^{TS}J_{C\text{-}F}$ = 2.9 Hz ,C-1), 21.7 (s, C-10'), 21.4 (s, C-10).

^{11}B NMR: δ_B (128 MHz, C_6D_6) -3.10 (bs).

^{19}F NMR: δ_F (471 MHz, C_6D_6) -189.37 (bs).

HRMS (ESI-pos, m/z): calc'd for $C_{56}H_{72}B_2I_2In_2N_8Li$ [M+Li]+1153.4267; found 1153.4140.

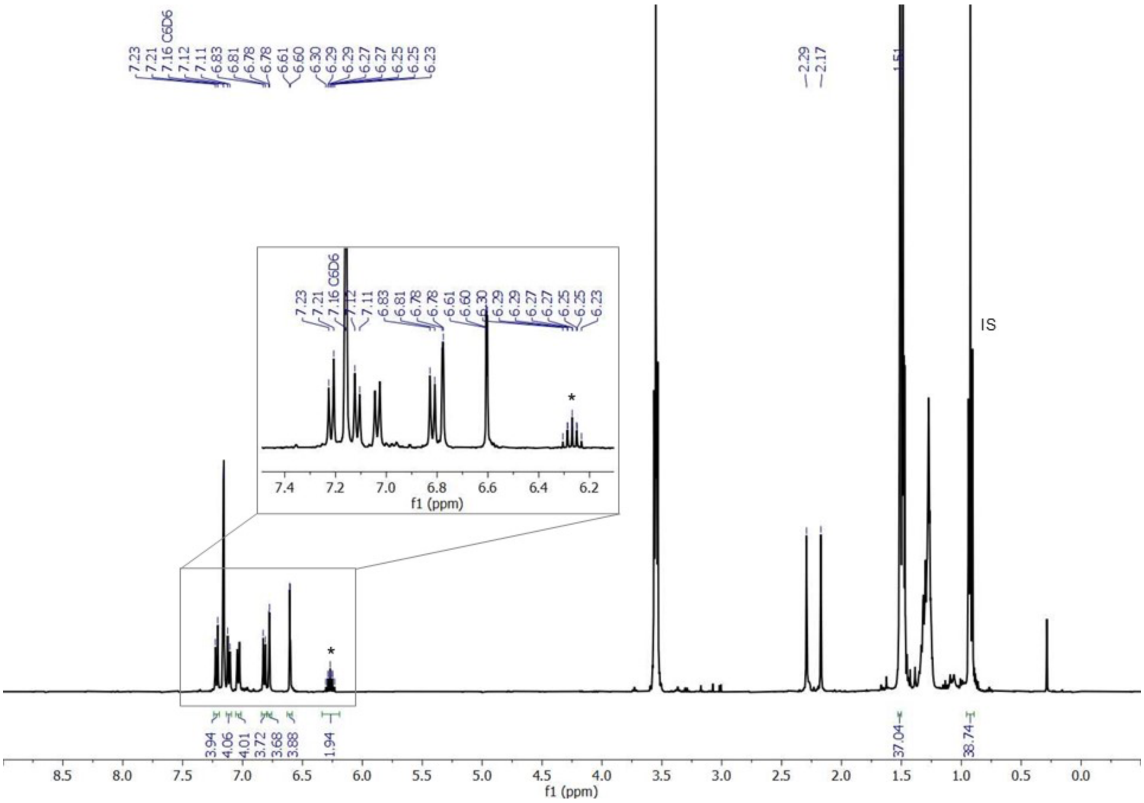

Figure S10. ^{1}H NMR spectrum (C_6D_6, 400 MHz) after reaction of **3-H** pentafluoropyridine to afford **3-F** and 2,3,5,6-tetrafluoropyridine (*) (IS = pentane).

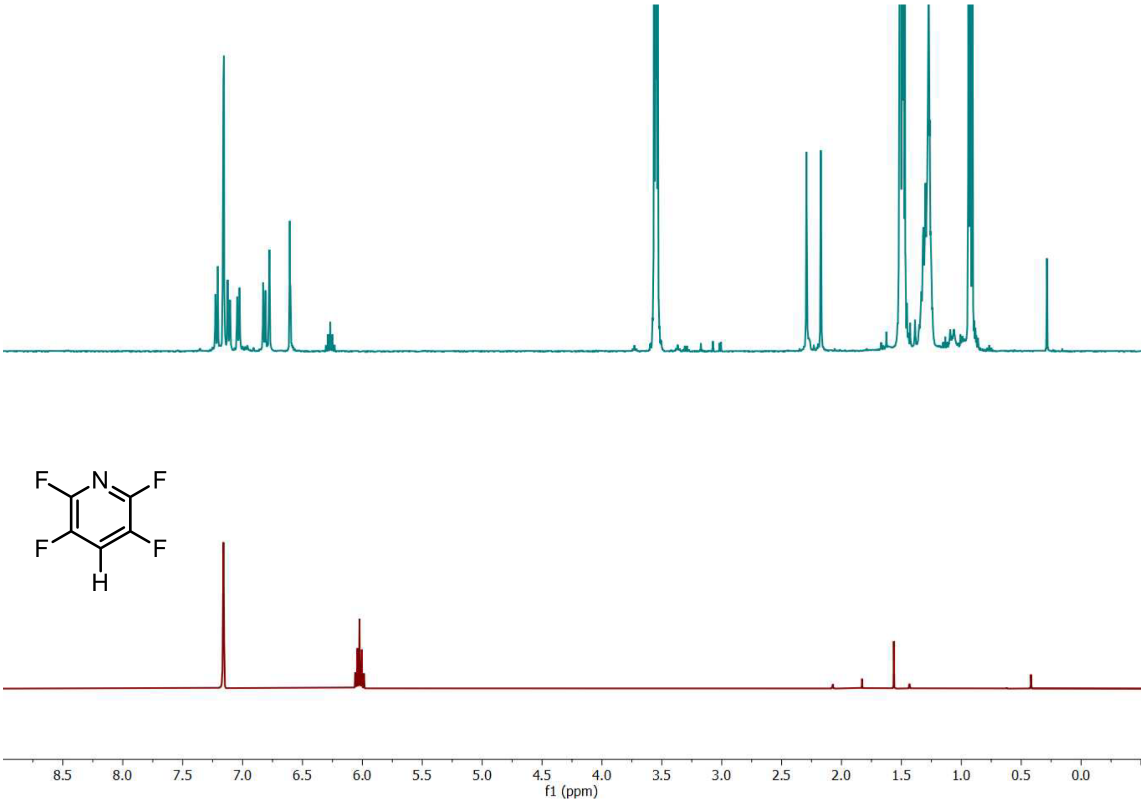

Figure S11. ^{1}H NMR stacked spectra (C_6D_6, 400 MHz) of reaction of 3-H pentafluoropyridine (top, green) and commercially available 2,3,5,6-tetrafluoropyridine (bottom, red).

4.3.1 Catalytic Hydrodefluorination of Pentafluoropyridine

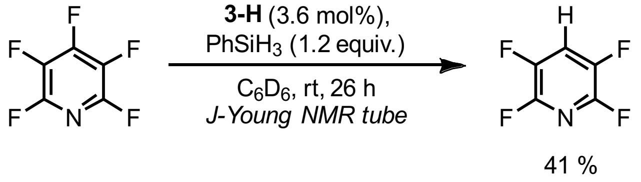

A stock solution of pentafluoropyridine (70.0 µL, 644 µmol) and phenylsilane (100.0 µL, 807 µmol) in C_6D_6 (0.6 mL) was prepared. A solution of In(II) complex **3-H** (2.0 mg, 0.036 equiv., 1.8 µmol, 3.6 mol%) and hexane (3.0 µL, 0.45 equiv., 23 µmol) internal standard (used to determine the quantity of **3-H**) in C_6D_6 (0.50 mL) were added into a J-Young NMR tube. The internal standards 1,3,5-trimethoxybenzene (2.4 mg, 0.28 equiv., 14.0 µmol) and trifluorotoluene (3 µL, 0.48 equiv., 24.0 µmol) were then added. Following this, pentafluoropyridine (5.5 µL, 1.0 equiv., 51 µmol) and phenylsilane (5.8 µL, 1.20 equiv., 63 µmol) were added to the solution at room temperature *via* the addition of stock solution (60 µL). A ^{1}H NMR was obtained 26 h later 2,3,5,6-tetrafluoropyridine (41 % NMR yield, see Figure S12-15) was identified.

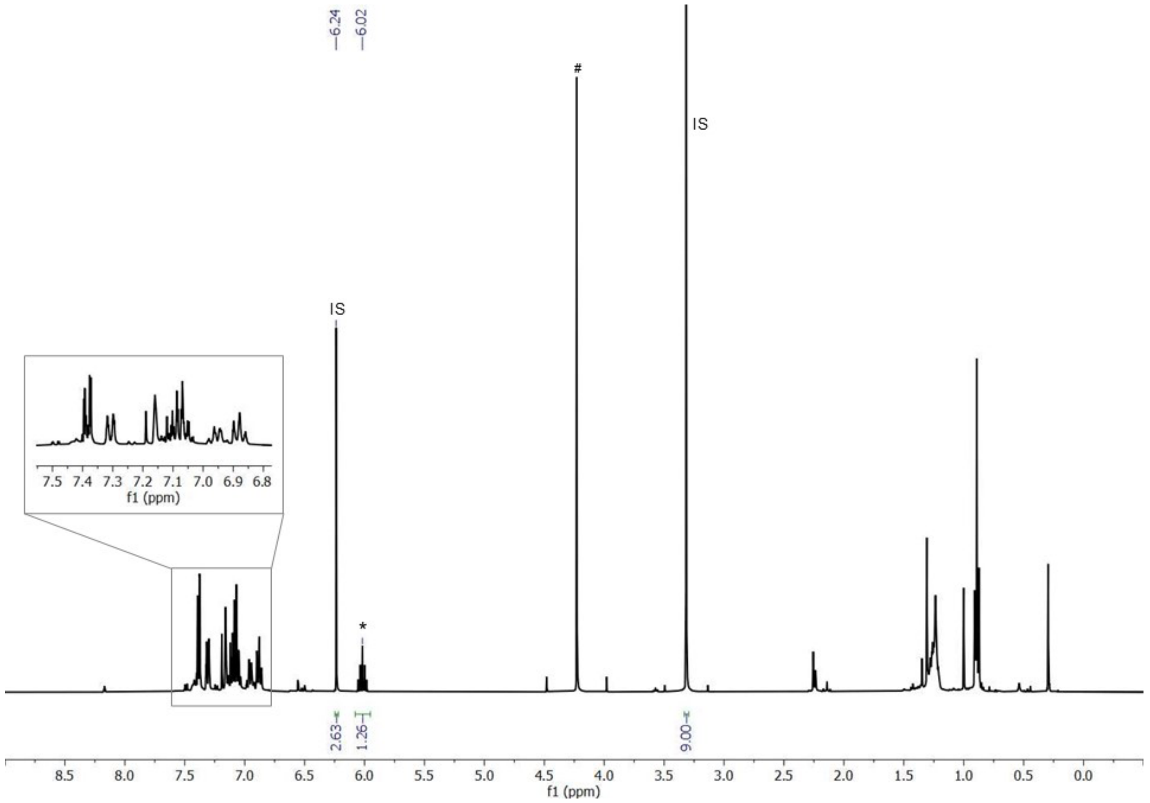

Figure S12. ^{1}H NMR spectrum (C_6D_6, 400 MHz) of the **3-H** catalysed reaction of pentafluoropyridine and phenylsilane to afford 2,3,5,6-tetrafluoropyridine (*) (IS = TMB; TFT present in aromatic region) (# = phenylsilane).

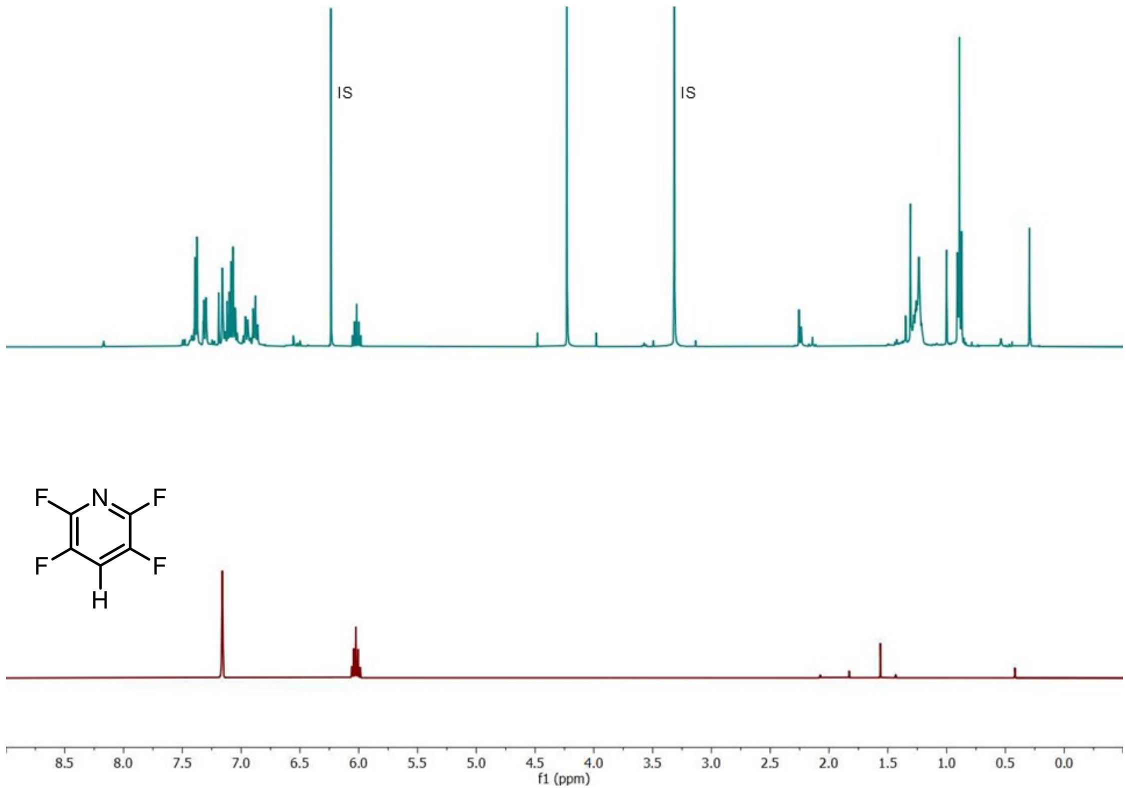

Figure S13. ^{1}H NMR stacked spectra (C_6D_6, 400 MHz) of the **3-H** catalysed reaction of pentafluoropyridine and phenyl silane (top, green) and 2,3,5,6-tetrafluoropyridine (bottom, red) (IS = TMB; TFT present in aromatic region).

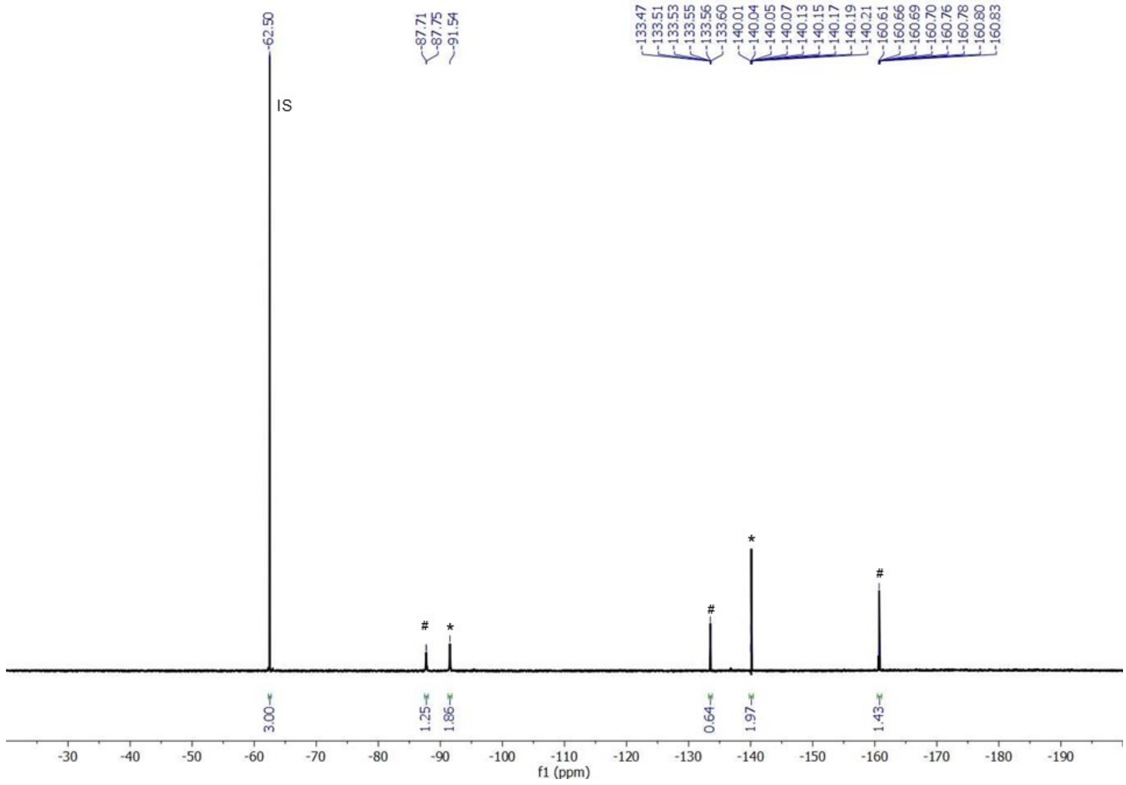

Figure S14. ^{19}F NMR spectra (C_6D_6, 471 MHz) of the **3-H** catalysed reaction of pentafluoropyridine (#) and phenyl silane to afford 2,3,5,6-tetrafluoropyridine (*) (IS = TFT).

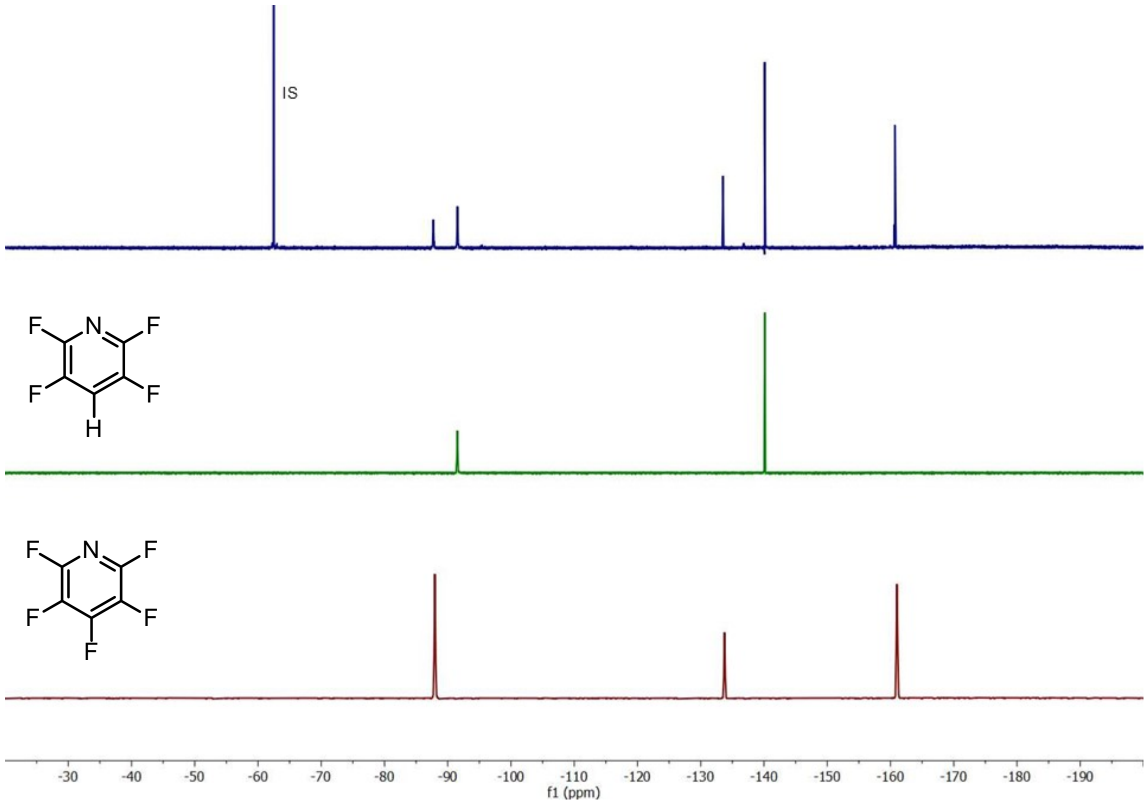

Figure S15. ^{19}F NMR stacked spectra (C_6D_6, 471 MHz) of the **3-H** catalysed reaction of pentafluoropyridine and phenyl silane (top, blue); 2,3,5,6-tetrafluoropyridine (middle, green); and pentafluoropyridine starting material (bottom, red) (IS = TFT).

4.4 Reactivity with Dimethyl Disulphide

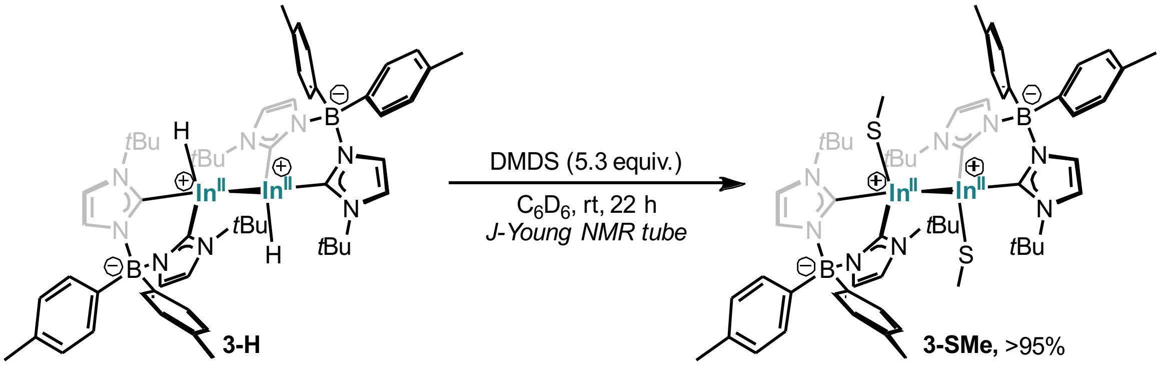

A J-Young NMR tube was prepared with a solution of In(II) complex 4 (2.4 mg, 1.0 equiv., 1.12 µmol) and hexane (2.0 µL, 7.2 equiv., 15.3 µmol) internal standard in C_6D_6 (0.50 mL). Following this, dimethyl disulphide (1.0 µL, 5.3 equiv., 11.2 µmol) was added to the solution at room temperature. The ^{1}H NMR was obtained 22 h later and showed quantitative conversion of **3-H** to **3-SMe** (>95% NMR yield, see Figure S81).

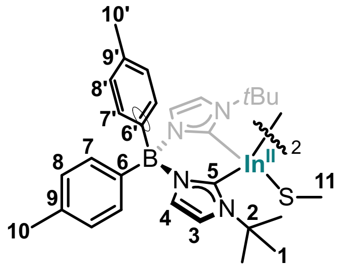

^{1}H NMR: δ_H (500 MHz, C_6D_6, 298 K) 7.44 (d, $^{3}J_{H\text{-}H}$ = 7.9 Hz, 4H, H-7), 7.17 (d, 4H, H-8), 7.10 (d, $^{3}J_{H\text{-}H}$ = 7.5 Hz, 4H, H-8'), 6.94 (d, $^{3}J_{H\text{-}H}$ = 7.9 Hz, 4H, H-7'), 6.91 (bs, 4H, H-4), 6.58 (d, $^{3}J_{H\text{-}H}$ = 1.7 Hz, 4H, H-3), 2.24 (s, 6H, H-10), 2.10 (s, 6H, H-10'), 1.77 (s, S-Me, 6H, H-11) 1.48 (bs, 36H, H-1).

^{13}C NMR: δ_C (101 MHz, C_6D_6, 298 K) 173.0 (s, C-5), 150.6 (bs, 6), 149.6 (bs, 6'), 135.8 (s, C-9), 135.6 (s, C-7), 134.6 (s, C-9'), 132.7 (s, C-7'), 128.7 (s, C-8), 128.4 (s, C-8'), 128.4 (s, C-4), 117.3 (s, C-3), 58.7 (s, C-2), 31.5 (s, C-1), 21.5 (s, C-10'), 21.4 (s, C-10), 11.4 (s, C-11)

^{11}B NMR: δ_B (128 MHz, C_6D_6, 298 K) -4.23 (bs).

HRMS (ESI-pos, m/z): calc'd for $C_{57}H_{85}B_2In_2N_8S$ $[M\text{-}SMe]^+$ 1155.4094 found 1155.4087.

4.5 Reactivity with Dibromo Bismuthine 4

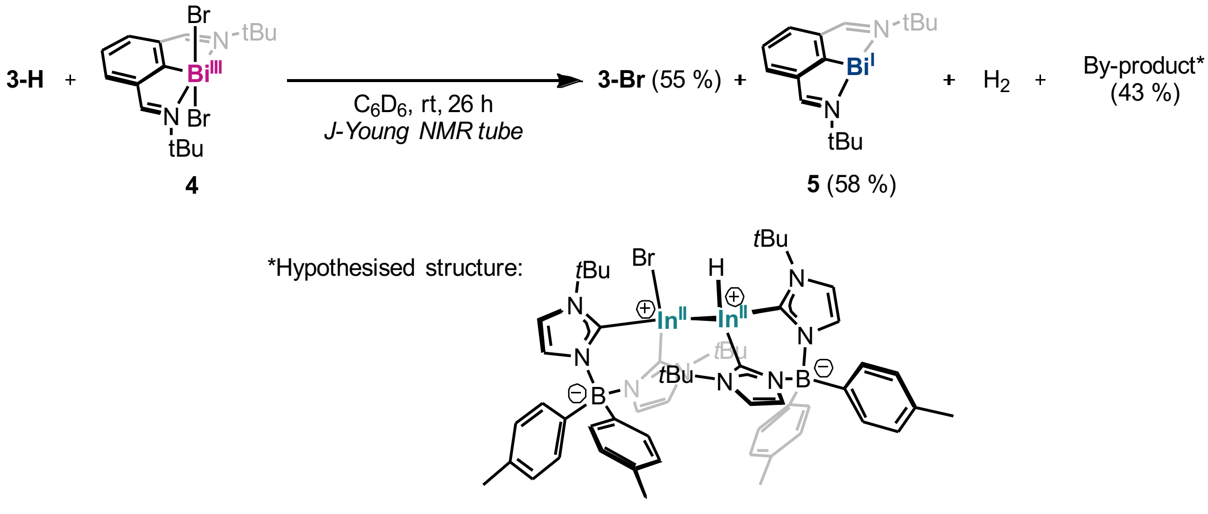

A J-Young NMR tube was prepared with a solution of complex **3-H** (3.8 mg, 1.0 equiv., 3.4 μmol) and hexane (3.0 μL, 6.77 equiv., 23 μmol) internal standard in C_6D_6 (0.50 mL). Following this, a solution of bismuthine dibromide (**4**) (2.1 mg, 1.0 equiv., 3.4 μmol) in C_6D_6 (0.20 mL) was added at room temperature. The solution gradually turned into a vibrant, dark green, indicative of the formation of bismuthinidene (**5**).[2] The ^{1}H NMR was obtained 26 hours later and showed conversion of **4** to **5** (58 % NMR yield), as well as the production of **3-Br** (55 % NMR yield) and hydrogen gas (see Figure S16 and Figure S17). An additional species was observed, similar in structure to **3-Br**, which is hypothesized to be structure **6** (43%). A broad hydride peak corresponding to a single proton present at 5.51 ppm could be attributed to this complex (see reaction scheme above and Figure S16).

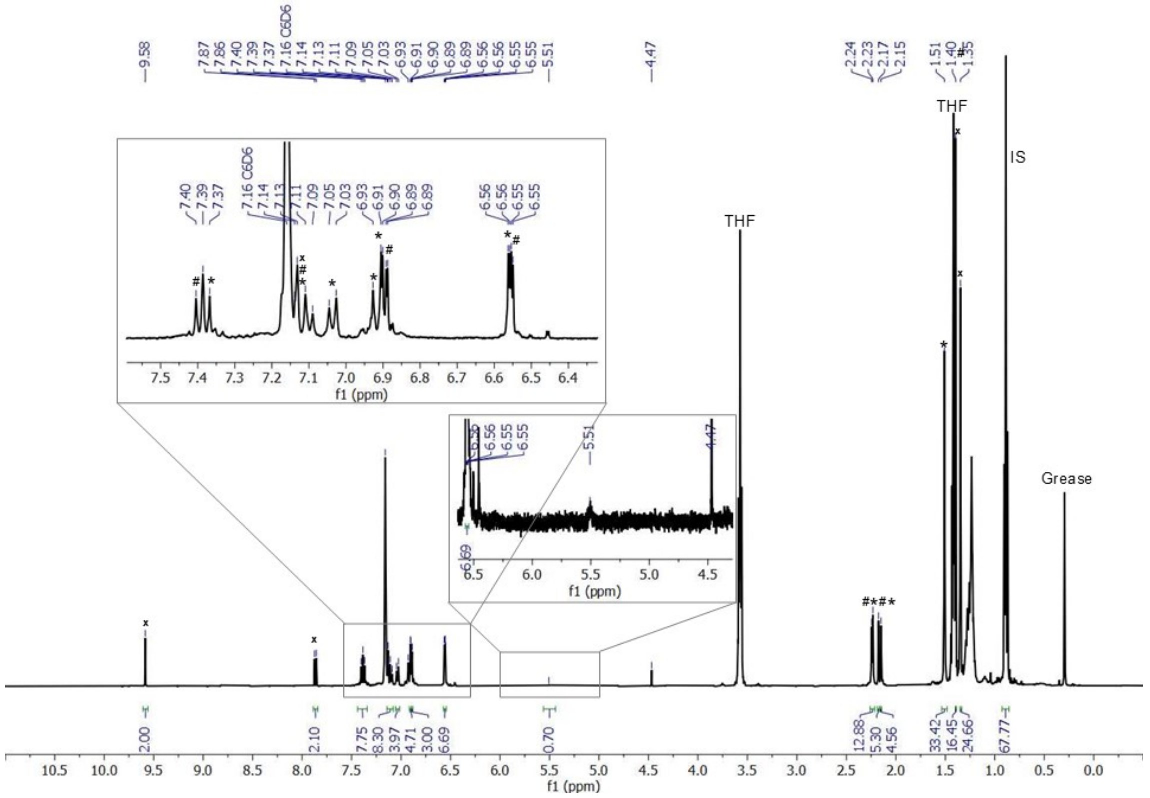

Figure S16. ^{1}H NMR spectrum (C_6D_6, 400 MHz) after reaction of **3-H** with dibromobismuthine **4** to afford bismuthinidene **5** (**x**) and **3-Br** (*) (IS = hexane). Hydrogen peak is observed at 4.47 ppm, another species (**#**) was observe, hypothesised to be **6**.

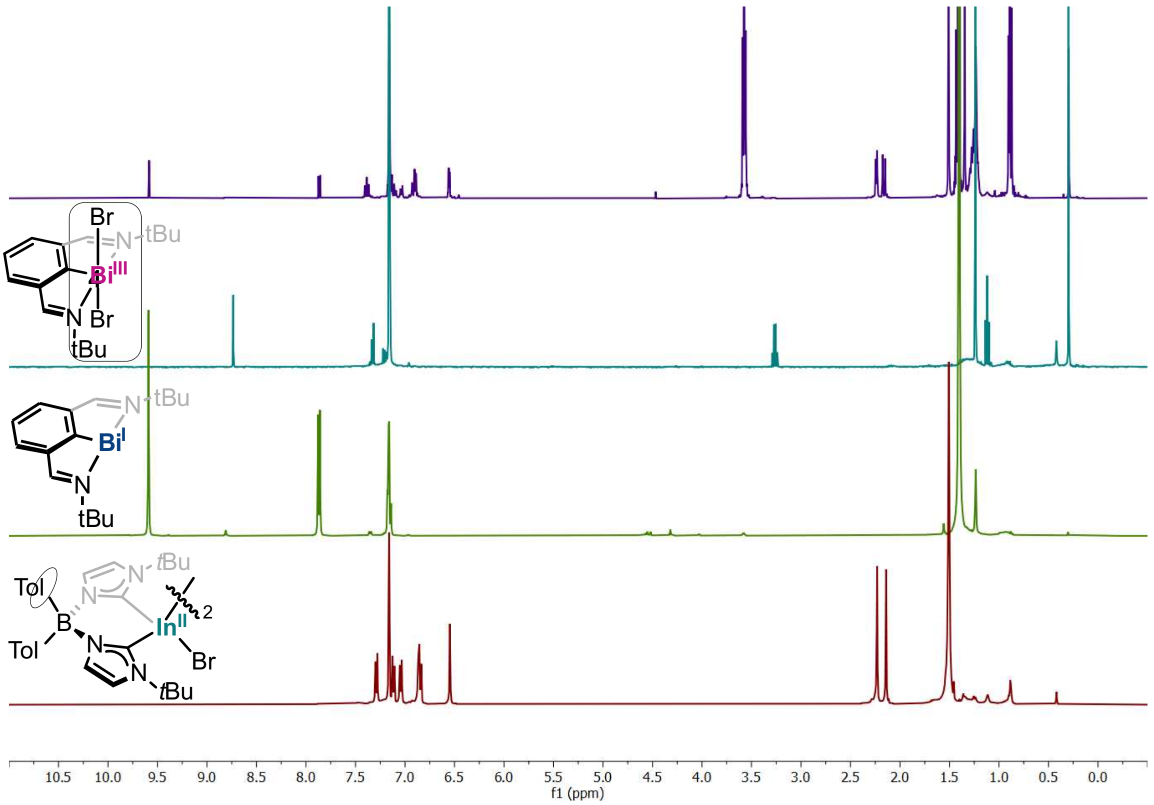

Figure S17. ^{1}H NMR stacked spectra (C_6D_6, 400 MHz) after reaction of **3-H** with dibromobismuthine **4** (top, purple), pure dibromobismuthine **4** (second from top, blue), pure bismuthinidene **5** (third from top, green), and pure **3-Br** (bottom, red).

4.6 Reactivity with Pyridinium Bromide

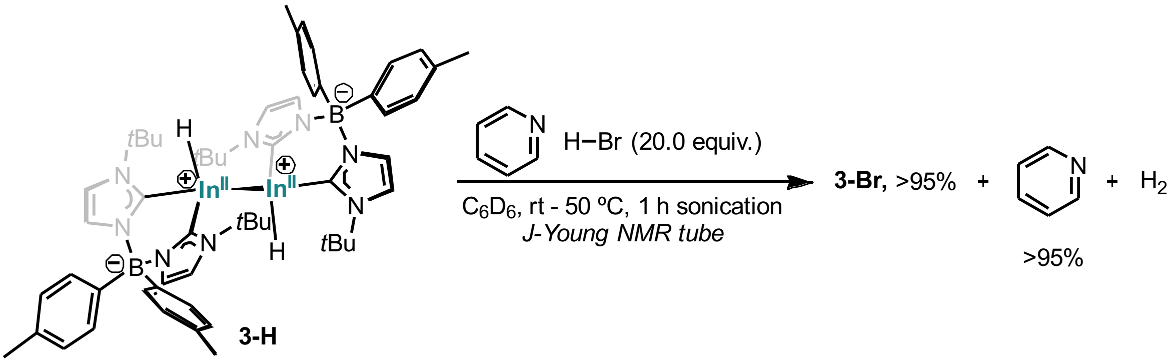

A J-Young NMR tube was prepared with a solution of In(II) complex **4** (1 mg, 1.0 equiv., 0.9 µmol) and hexane (3.0 µL, 26.0 equiv., 23 µmol) internal standard in C_6D_6 (0.50 mL). Following this, pyridinium bromide (2.9 mg, 20.0 equiv., 18 µmol) was added to the solution at room temperature. Due to the insolubility of pyridinium bromide, the solution was sonicated at room temperature and reactivity was observed via NMR within 1 minute. The solution was further sonicated for 1 hour, the bath temperature beginning room temperature and gradually warming to 50 ºC. The ^{1}H NMR was obtained and showed full conversion of **3-H** to **3-Br** (> 95% NMR yield) as well as the production of pyridine (>95 % NMR yield) and hydrogen gas (see Figure S18, Figure S19 and Figure S20).

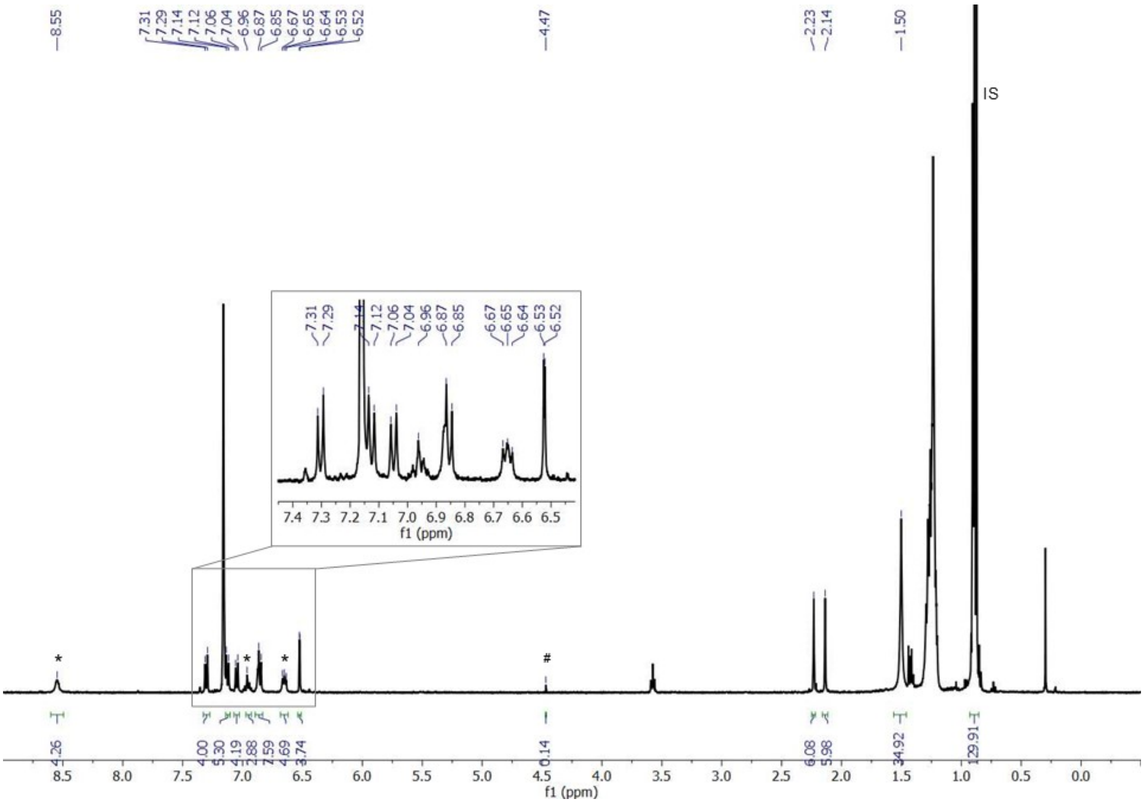

Figure S18. ^{1}H NMR spectrum (C_6D_6, 400 MHz) after reaction of **3-H** with pyridinium bromide to afford **3-Br**, pyridine (*) and H_2 (**#**) (IS = hexane).

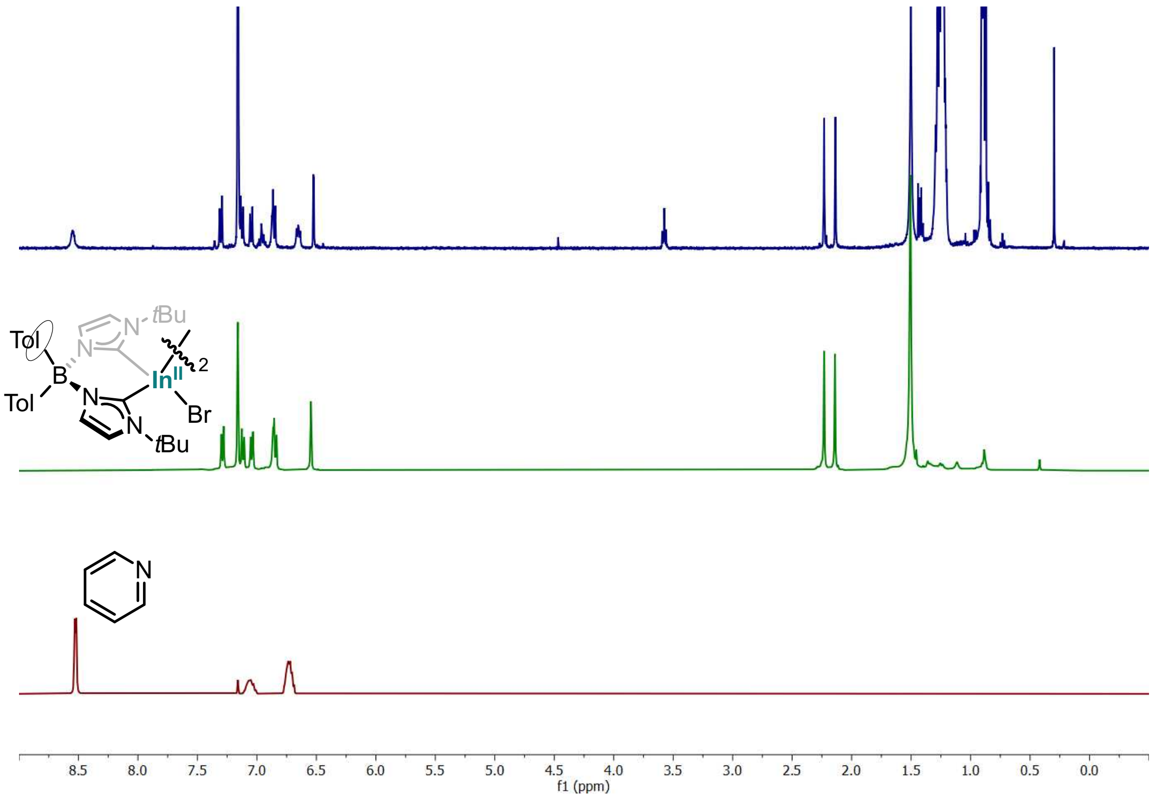

Figure S19. ^{1}H NMR stacked spectra (C_6D_6, 400 MHz) of reaction of **3-H** with pyridinium bromide (top, blue) to afford **3-Br** (middle, green) and pyridine (bottom, red).

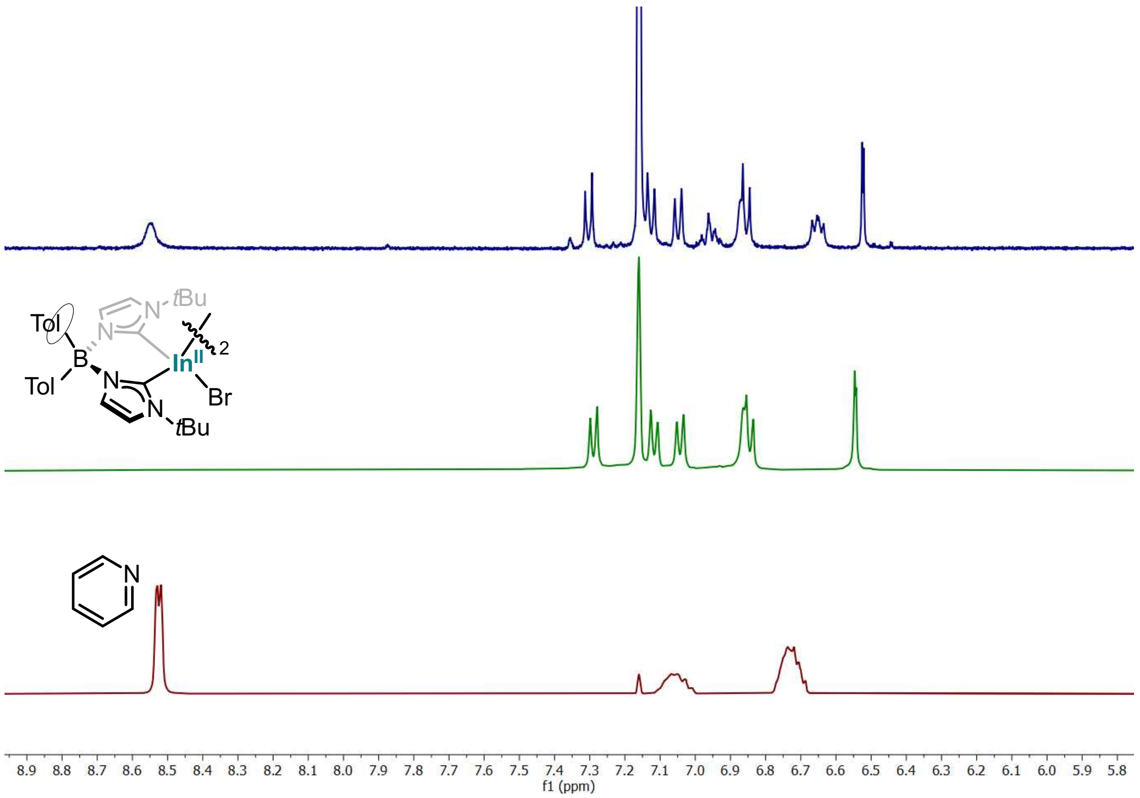

Figure S20. Expanded ^{1}H NMR stacked spectra (C_6D_6, 400 MHz) of reaction of **3-H** with pyridinium bromide (top, blue) to afford **3-Br** (middle, green) and pyridine (bottom, red).

4.7 Unsuccessful Reactivity Studies

The reactivity of **3-H** towards a range of other substrates was also examined, affording complex mixtures in which products rapidly decomposed or which did not contain any identifiable products from the intended transformations (see Table S1).

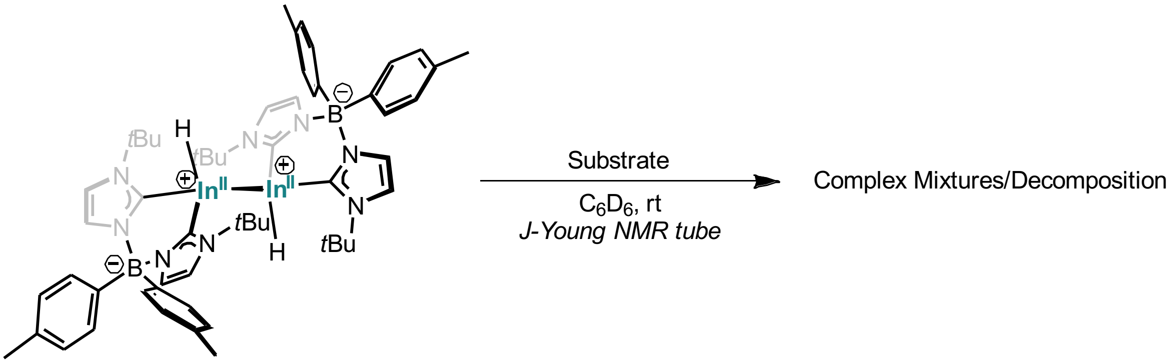

3-H

Table S1. Substrates and results of unsuccessful reactivities with **3-H**.

**Substrate**	**Result**
Acetaldehyde	Decomposition to complex mixture.
Bi(III)Br_2 bis(NHC)borate Complex 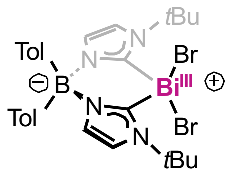	Identifiable products, such as **3-Br** and reduced Bi species. However, these species were accompanied by several impurities, attributed to rapid decomposition to a complex mixture of products.
CO_2 (1 barg)	Mixture of multiple unidentifiable products.
Dibromomethane	Decomposition to complex mixture.
Ethylene (1 barg)	No reactivity at rt; multiple species after heating to 50 ºC for 76 hours in a complex and unresolvable mixture.
p-tolyl disulphide	Successful observation of the product in NMR however a complex mixture proving challenging to elucidate due to multiple aromatic peaks (DMDS used to reduce complexity, see Section 4.4).

5 Stability Studies of 3-H

5.1 Thermal Decomposition

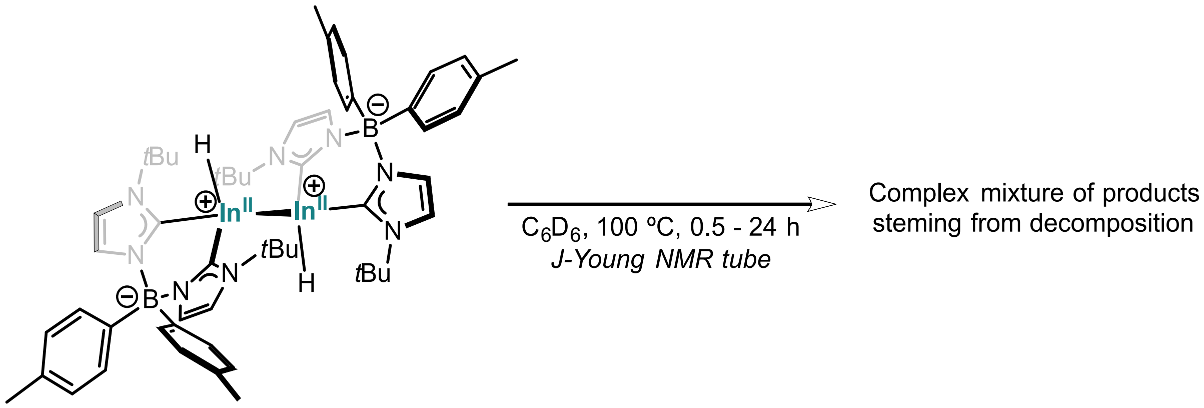

A J-Young tube was charged with a solution of **3-H** (3.2 mg, 2.5 mmol) in C_6D_6 (0.5 mL) and heated at 100ºC. Periodic monitoring by ^{1}H NMR spectroscopy revealed progressive decomposition of **3-H** into multiple unidentifiable species (see Figure S21 and Figure S22), including In black, noting that H_2 was not observed in solution (expected ^{1}H NMR peak in C_6D_6 δ_H = 4.47 ppm).

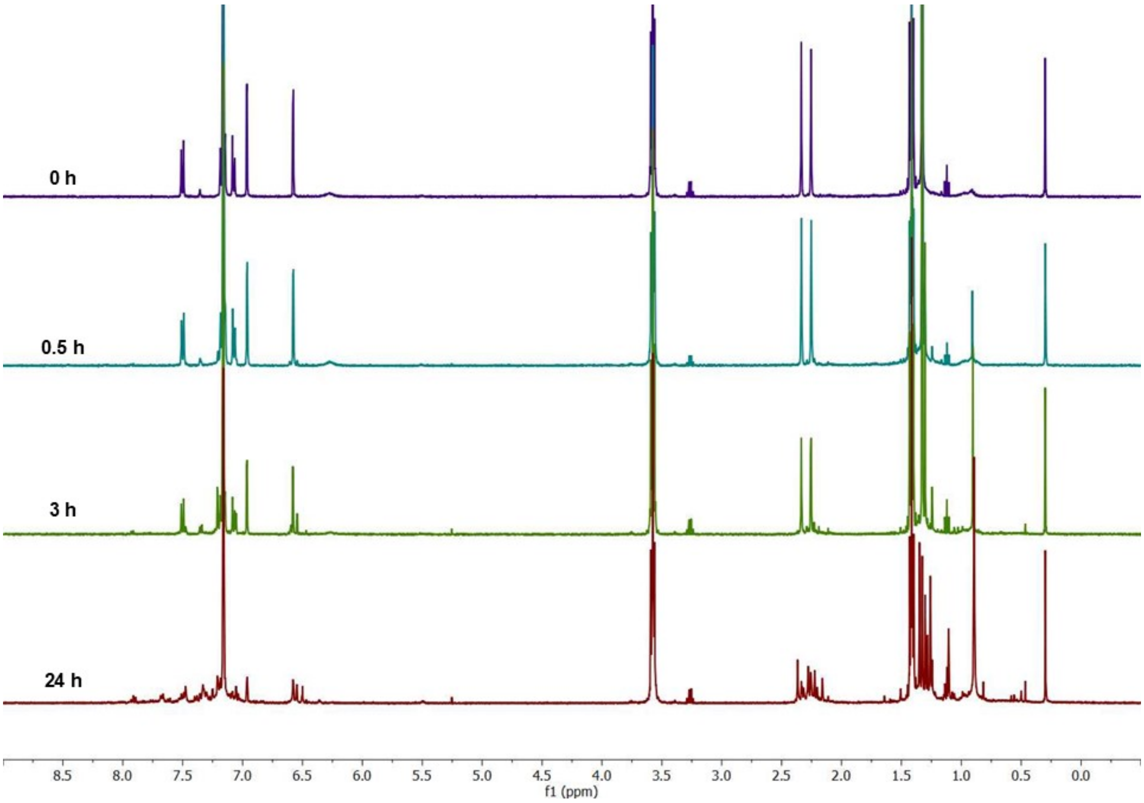

Figure S21. ^{1}H NMR spectrum (C_6D_6, 400 MHz) of **4** decomposition at 100ºC (0 - 24 h).

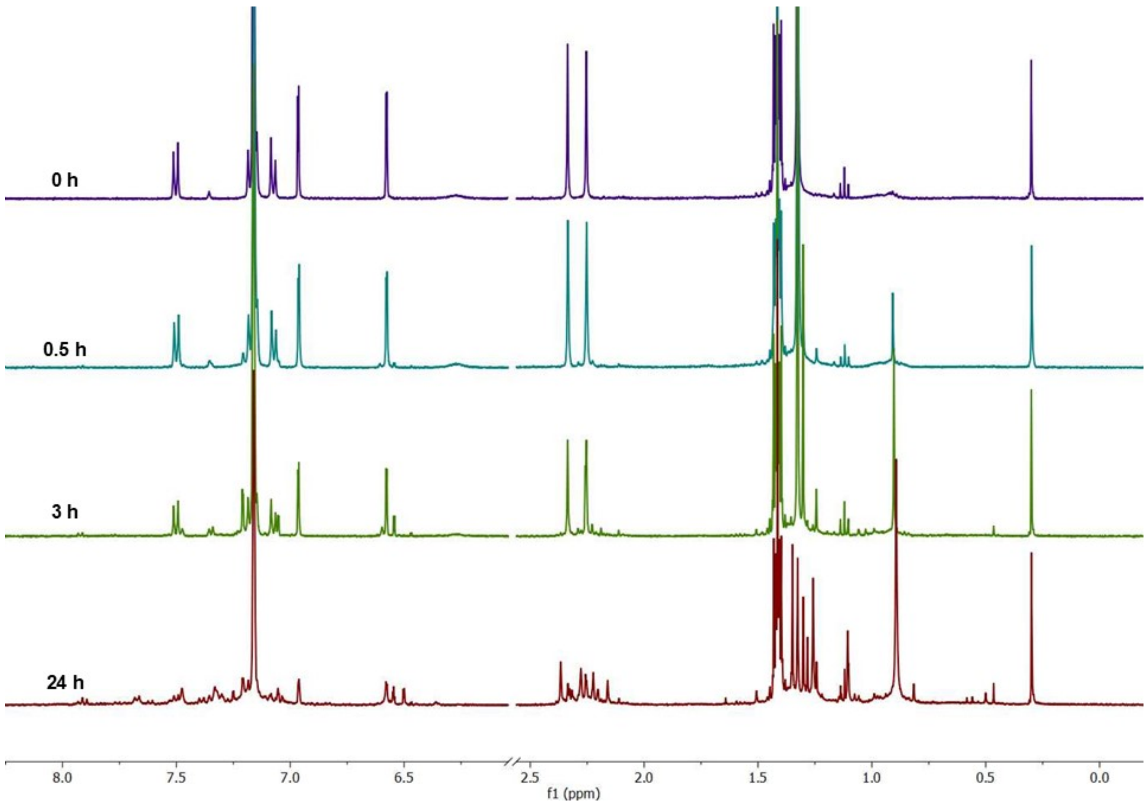

Figure S22. ^{1}H NMR expanded spectrum (C_6D_6, 400 MHz) of **4** decomposition at 100ºC (0 - 24 h).

5.2 Air Exposure

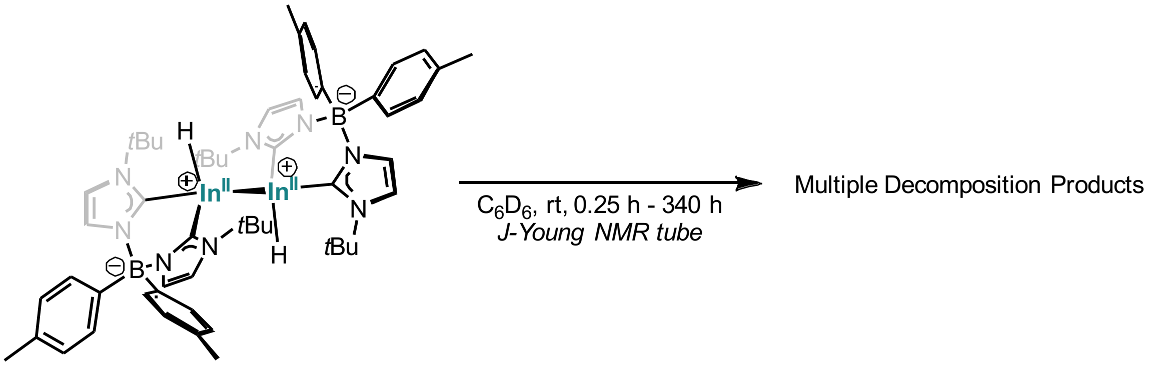

3-H

A J-Young tube was charged with a solution of **3-H** (5.7 mg, 4.5 mmol) in C_6D_6 (0.5 mL) and exposed to air. Periodic monitoring by ^{1}H NMR spectroscopy revealed progressive decomposition of **3-H** into multiple broad bands and unidentifiable species (see Figure S23 and Figure S24).

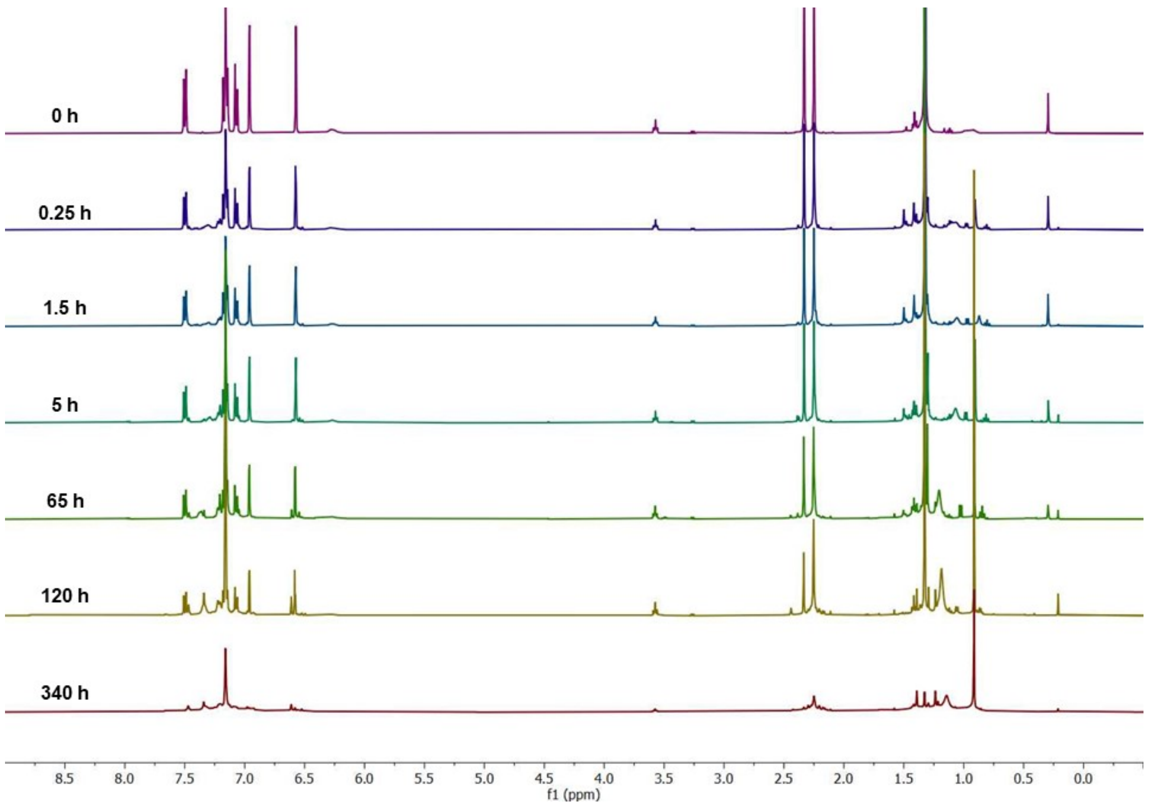

Figure S23. ^{1}H NMR spectrum (C_6D_6, 400 MHz) of **4** decomposition post air exposure (0 - 340 h).

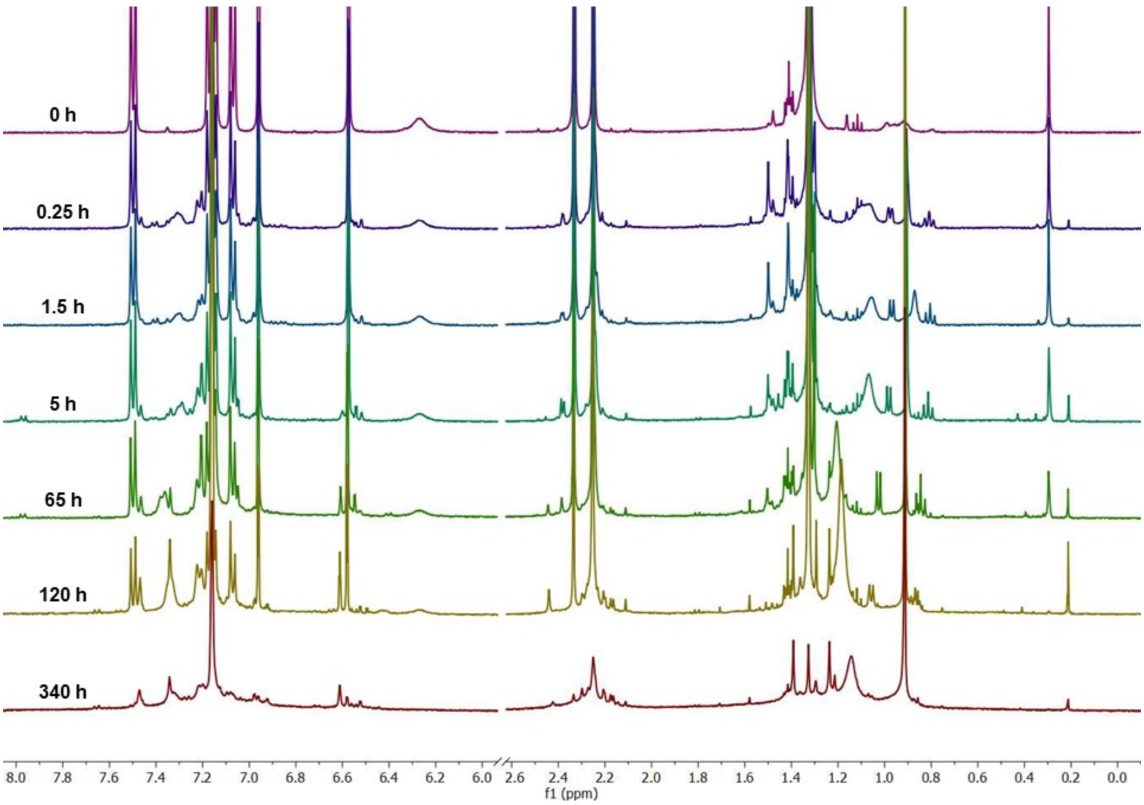

Figure S24. ^{1}H NMR expanded spectrum (C_6D_6, 400 MHz) of **4** decomposition post air exposure (0 - 340 h).

6 Computational Details

Geometry optimizations were performed using the ORCA 6.0 software.[3] Geometry optimizations were computed using the functional PBE0[4,5] functional with Grimme dispersion corrections D3-BJ[6,7] or D4[8,9] in combination with def2-SVP, def2-TZVP and def2-TZVPP basis sets.[10] Analytical Hessians were computed to determine the nature of stationary points (one and zero imaginary frequencies for transition states and minima, respectively)[11] and to calculate unscaled zero-point energies (ZPEs) as well as thermal corrections and entropy effects using the standard statistical-mechanics relationships for an ideal gas. The atomic partial charges were computed with the natural bond orbital (NBO) method.[12,13] Natural Resonance Theory has been employed as implemented in NBO 7.0.[14] The topological quantum theory of atoms in molecules (QTAIM),[15] and Laplacian of the electron density analyses were carried out with AIMAII.[16] All these analysis were performed at the PBE0-D4/def2-TZVPP level of theory.

The nature of the chemical bonds were investigated by means of the Energy Decomposition Analysis (EDA) method, which was developed by Morokuma[17] and by Ziegler and Rauk.[18,19] The bonding analysis focuses on the interaction energy ΔE_{int} of a bond A–B between two (or more) fragments A and B in the particular electronic reference state and in the frozen geometry AB. This energy is divided into three main components, and one more related to the dispersion according to the level of theory used (Eq. S1).

$$\Delta E_{\text{int}} = \Delta E_{elst} + \Delta E_{Pauli} + \Delta E_{orb} + (\Delta E_{disp}) \quad \text{(Eq. S1)}$$

The term ΔE_{elst} corresponds to the quasiclassical electrostatic interaction between the unperturbed charge distributions of the prepared fragments, and it is usually attractive. The Pauli repulsion ΔE_{Pauli} is the energy change associated with the transformation from the superposition of the unperturbed wave functions (Slater determinant of the Kohn-Sham orbitals) of the isolated fragments to the wave function $\Psi_0 = N\hat{A}[\Psi^A\Psi^B]$, which properly obeys the Pauli principle through explicit antisymmetrization ($\hat{A}$ operator) and renormalization (N = constant) of the product wave function. It comprises the destabilizing interactions between electrons of the same spin on either fragment. The orbital interaction ΔE_{orb} accounts for charge transfer and polarization effects.[20] In the case that the Grimme dispersion corrections[6–8] are computed, the term ΔE_{disp} is added to equation S1. Further details on the EDA method can be found in the literature.[21] The relaxation of the fragments to their equilibrium geometries at the electronic ground state is termed ΔE_{prep}, because it may be considered as preparation energy for chemical bonding. The addition of ΔE_{prep} to the intrinsic interaction energy ΔE_{int} gives the total energy ΔE, which is, by definition, the opposite sign of the bond dissociation energy D_e:

$$\Delta E(-D_e) = \Delta E_{\text{int}} + \Delta E_{prep} \quad \text{(Eq. S2)}$$

The EDA–NOCV method combines the EDA with the natural orbitals for chemical valence (NOCV) to decompose the orbital interaction term ΔE_{orb} into pairwise contributions.[22–24] The NOCVs Ψ_i are defined as the eigenvector of the valence operator, $\hat{V}$, given by Eq. S3.

$$\hat{V}\Psi_i = v_i\Psi_i \qquad \text{(Eq. S3).}$$

In the EDA–NOCV scheme the orbital interaction term, ΔE_{orb}, is given by Eq. S4,

$$\Delta E_{orb} = \sum_k \Delta E_k = \sum_{k=1}^{N/2} v_k \left[-F_{-k,k}^{TS} + F_{k,k}^{TS} \right] \qquad \text{(Eq. S4)}$$

in which $F_{-k,-k}^{TS}$ and $F_{k,k}^{TS}$ are diagonal transition state Kohn–Sham matrix elements corresponding to NOCVs with the eigenvalues $-v_k$ and v_k, respectively. The ΔE_k^{orb} term for a particular type of bond is assigned by visual inspection of the shape of the deformation density $\Delta\rho_k$. The latter term is a measure of the size of the charge deformation, and it provides a visual notion of the charge flow that is associated with the pairwise orbital interaction. The EDA–NOCV scheme thus provides both qualitative and quantitative information about the strength of orbital interactions in chemical bonds. The EDA-NOCV calculations were carried out with ADF2019.[25] The basis sets for all elements have triple-ζ quality augmented by two sets of polarizations functions and one set of diffuse functions. Core electrons were treated by the frozen-core approximation. This level of theory is denoted BP86-D3(BJ)/TZ2P.[6,7,26,27] Scalar relativistic effects have been incorporated by applying the zeroth-order regular approximation (ZORA).[28] The geometries for this analysis were optimized in Gaussian 16 C.01 at the PBE0-D3(BJ)/def2-TZVPP level of theory.

6.1 Benchmark

Table S2. Benchmarking of compound **3-H** with different functionals (DFT/def2-TZVPP) and their respective computed relevant bond lengths.

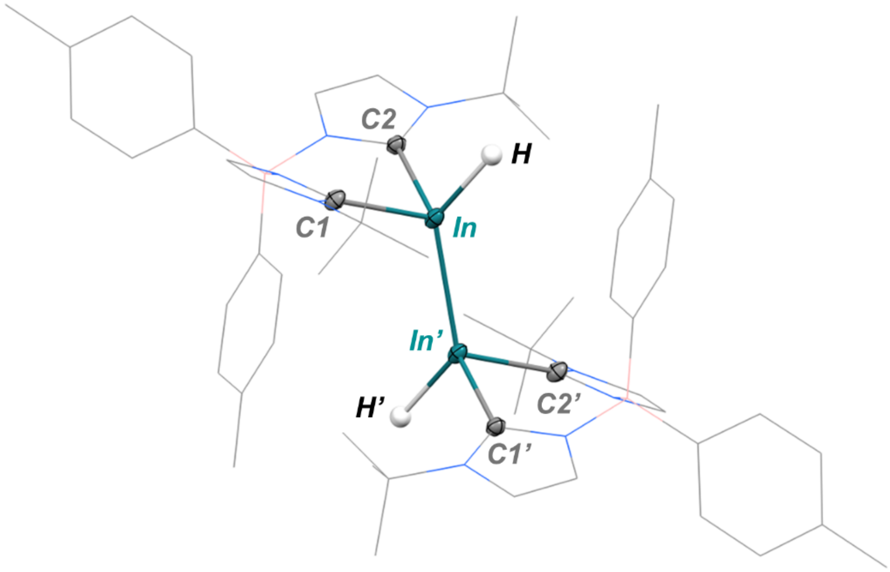

Parameter	XRD	PBE-D3BJ	PBE0-D3BJ	PBE0-D4	wB97X3c-D3BJ	M062X
In-H	1.75(2)	1.755	1.746	1.747	1.745	1.773
In-In'	2.773	2.768	2.753	2.755	2.758	2.818
C1-In	2.269	2.249	2.236	2.237	2.251	2.288
C2-In	2.265	2.246	2.234	2.235	2.252	2.291
C1-In-H	96.7	105.4	105.4	105.4	104.7	104.6
RMSD	-	1.164	1.158	1.155	1.105	1.311

Based on the benchmarking results (Table S2), the PBE0-D4 functional was selected for all subsequent geometry optimisations. This functional reproduces the experimental bond metrics of compound **3-H** with accuracy, showing excellent agreement for both In–H and In–In bond lengths, while maintaining a reasonable computational cost. The PBE0 hybrid functional also provided a geometry close to the experimental, as reflected by its RMSD value when compared to the XRD structure of **3-H**. Together, these features make PBE0-D4 a well-balanced choice for accurately modelling both the geometry and electronic structure of low-valent In species reported in this work.

6.2 Computed FT-IR Spectra of 3-H

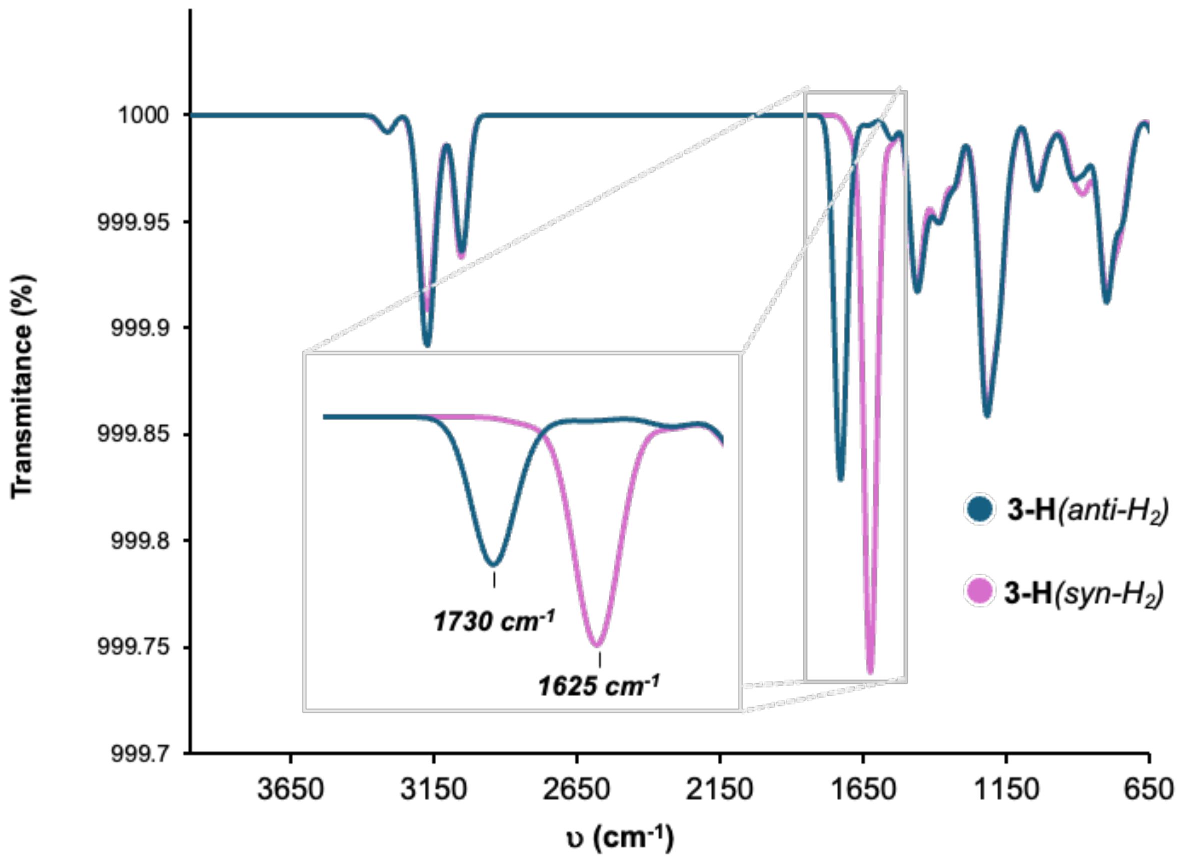

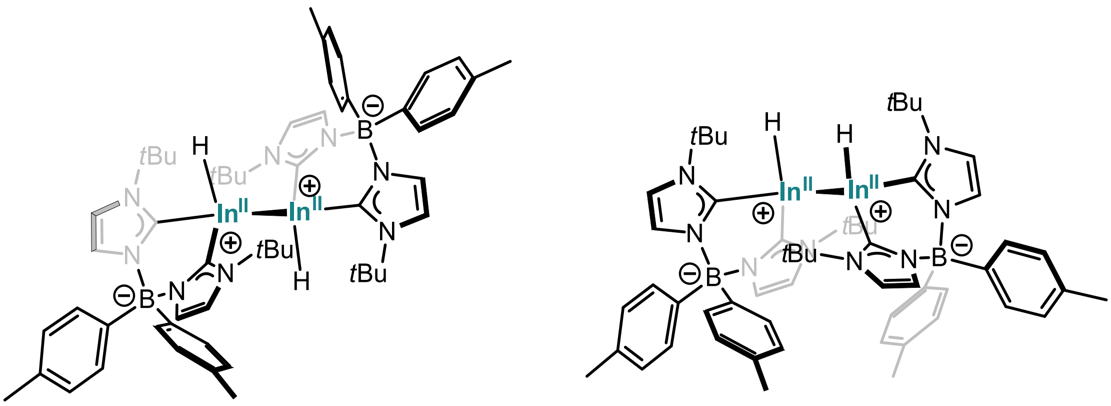

4-(*anti*-H_2)

4-(*syn*-H_2)

Asymmetric stretching: 1730.6 cm^{-1} 1617.7 cm^{-1}

Symmetric stretching: 1740.8 cm^{-1} [illegible]

Figure S25. Computed FT-IR spectra for **3-H** at the PBE0-D4/def2-TZVPP level of theory. In blue, FT-IR spectrum corresponding to **3-H***(anti-H₂)*. In pink, FT-IR spectrum corresponding to **3-H***(anti-H₂)*. Bottom, main In-H stretching modes and their computed frequencies.

6.3 Frontier Molecular Orbitals (FMO)

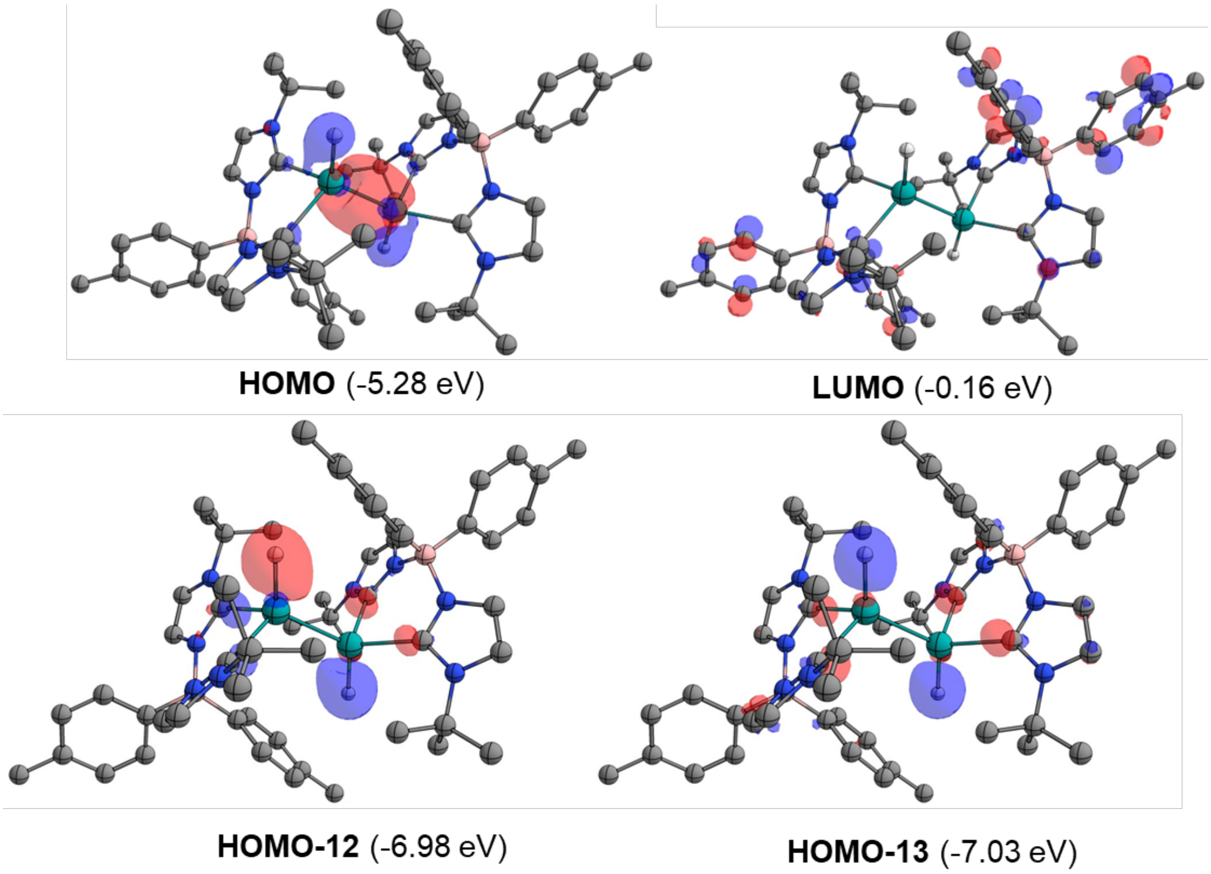

Figure S26. Shape of the frontier molecular orbitals (isocontour = 0.05 au) for **3-H** and eigenvalues (in eV) at the PBE0-D4/def2-TZVPP level of theory. Hydrogen atoms were omitted for clarity.

6.4 Natural Bond Orbitals (NBO)

Table S3. NBO results calculated at PBE0-D4/def2-TZVPP level of theory: partial charges, Q (in e), Wiberg bond order, WBI (in a.u.).

	**3-H**
**Q(In)**	0.59
**Q(C_{carb})**	0.11
**Q(H)**	-0.34
**WBI(In−C)**	0.56
**WBI(In−H)**	0.8
**WBI(In−In‘)**	0.88

Table S4. NBO results at the PBE0-D4/def2-TZVPP of **3-H**. Hydrogen atoms were omitted for clarity except those bound to In atoms.

Orbital	Occ.	Contribution from atoms to the orbitals	Atomic orbitals
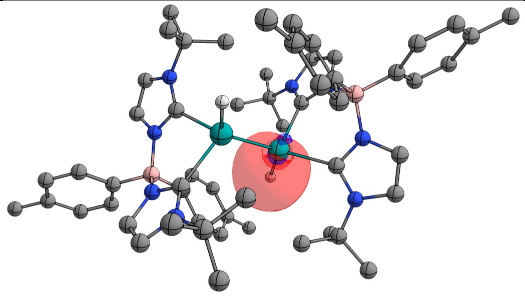	1.95	In (31.9%) - H (68.1%)	In: s(28.89%) p 2.45(70.91%) d 0.01(0.17%) f 0.00(0.04%)<br>H: s(99.76%)p 0.00(0.24%)d 0.00(0.00%)
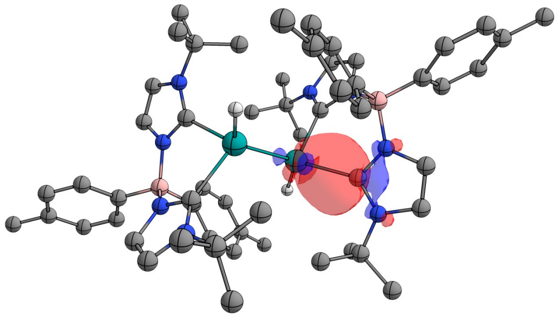	1.93	In (18.6%) – C1 (81.4%)	In: s(17.21%)p 4.80(82.67%)d 0.00(0.06%) f 0.00(0.06%)<br>C: s(40.12%)p 1.49(59.86%)d 0.00(0.01%) f 0.00(0.01%)
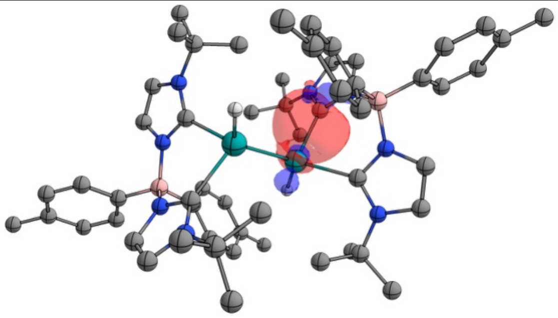	1.93	In (18.6%) – C2 (81.4%)	In: s(16.23%)p 5.15(83.65%)d 0.00(0.05%) f 0.00(0.06%)<br>C: s(40.12%)p 1.49(59.86%)d 0.00(0.01% f 0.00(0.01%)
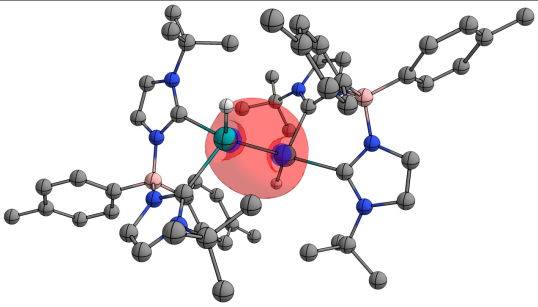	1.88	In (49.6%) – In' (50.4%)	In: s(37.60%)p 1.66(62.27%)d 0.00(0.10%) f 0.00(0.03%)<br>In':s(37.90%)p 1.63(61.95%)d 0.00(0.11%) f 0.00(0.03%)

6.5 Atoms in Molecules (AIM)

Table S5. QTAIM Local topological parameters of the charge density at the main Bond Critical Points of the H-In-In'-H' unit calculated at PBE0-D4/def2-TZVPP level of theory: Local kinetic energy density (G), Local potential energy density (V), local electronic energy density (H) and ellipticity (ϵ).

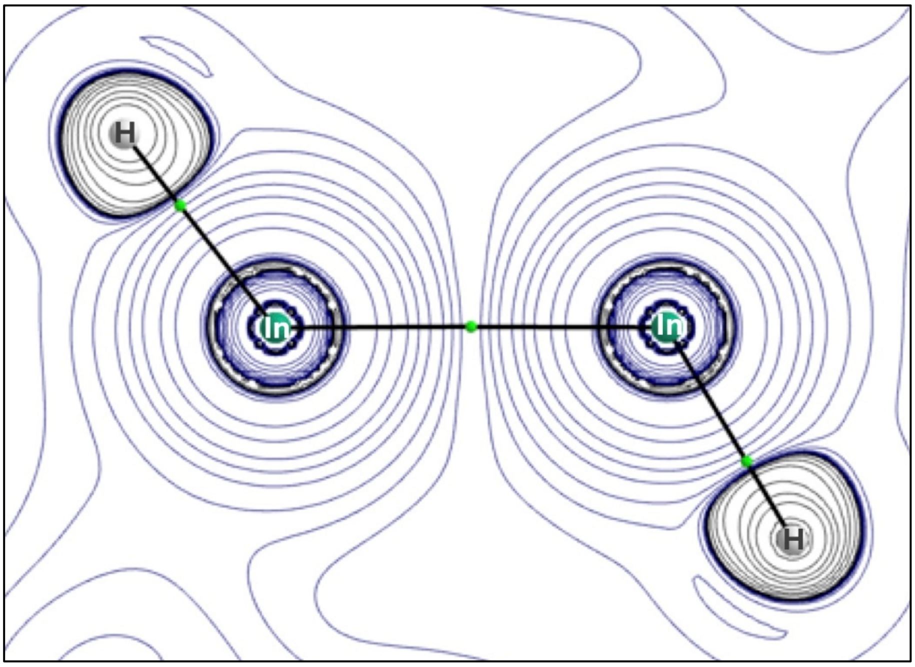

BCP	ρ(r) [e/bohr^3]	∇^2ρ(r) [e/bohr^5]	V	G	H	ϵ	Delocalization Index
In-H	0.099	0.125	-0.1165	0.0739	-0.0426	0.005	0.725
In-In'	0.0549	0.011	-0.0347	0.0188	-0.0159	0.015	0.741

Table S6. QTAIM Local topological parameters of the charge density at the main Bond Critical Points of the C1-In-C2 unit calculated at PBE0-D4/def2-TZVPP level of theory: Local kinetic energy density (G), Local potential energy density (V), local electronic energy density (H) and ellipticity (ϵ).

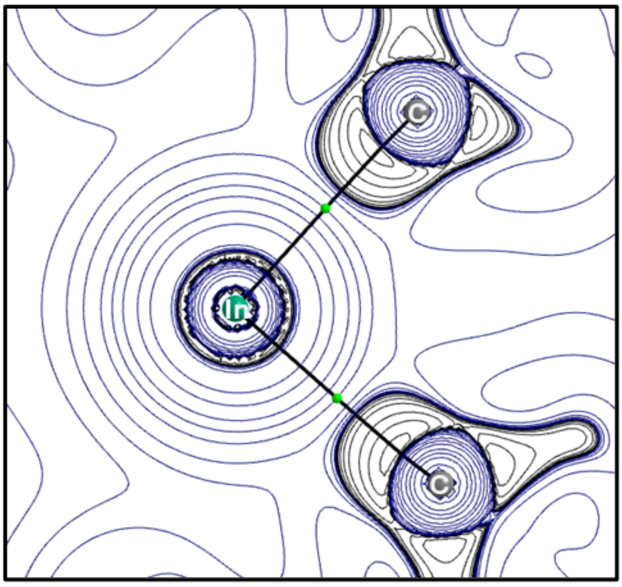

BCP	ρ(r) [e/bohr^3]	∇^2ρ(r) [e/bohr^5]	V	G	H	ϵ	Delocalization Index
In-C1	0.082	0.193	-0.0946	0.0715	-0.0231	0.07	0.563
In-C2	0.082	0.195	-0.0952	0.0719	-0.0232	0.07	0.563

Table S7. QTAIM Local topological parameters of the charge density at the main Bond Critical Points of the In-H----H-C unit calculated at PBE0-D4/def2-TZVPP level of theory: Local kinetic energy density (G), Local potential energy density (V), local electronic energy density (H) and ellipticity (ϵ).

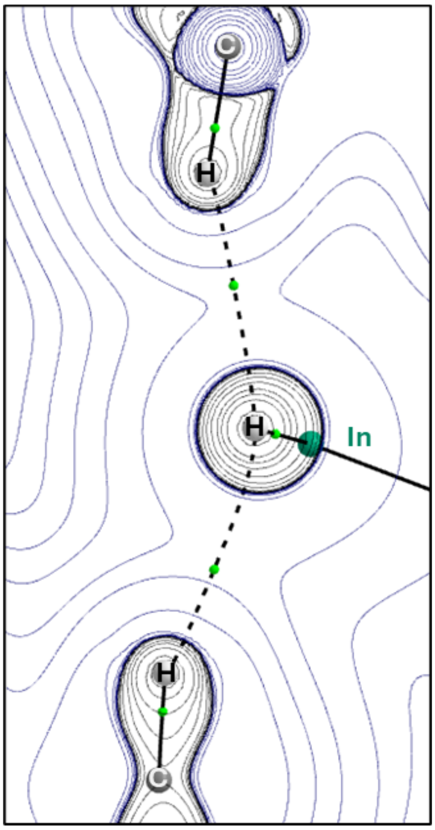

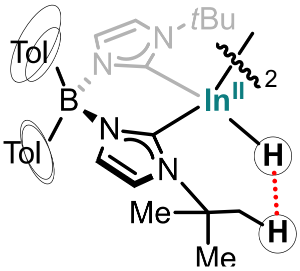

BCP	ρ(r) [e/Bohr^3]	∇^2ρ(r) [e/Bohr^5]	V	G	H	ϵ	Delocalization Index
In-H---H_a	0.011	0.025	-0.0049	0.0056	-0.0006	0.21	0.015
In-H---H_b	0.009	0.021	-0.0041	0.0047	-0.0007	0.29	0.038

Table S8. QTAIM Local topological parameters of the charge density at the main Bond Critical Points of the In-H---C unit calculated at PBE0-D4/def2-TZVPP level of theory: Local kinetic energy density (G), Local potential energy density (V), local electronic energy density (H) and ellipticity (ϵ).

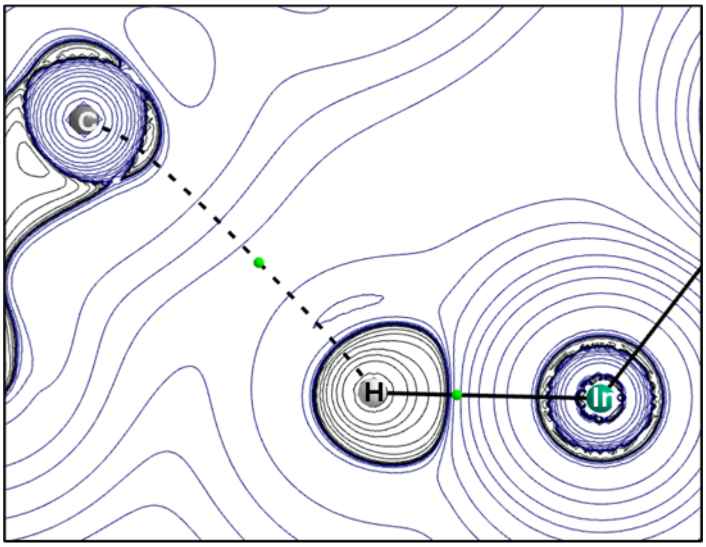

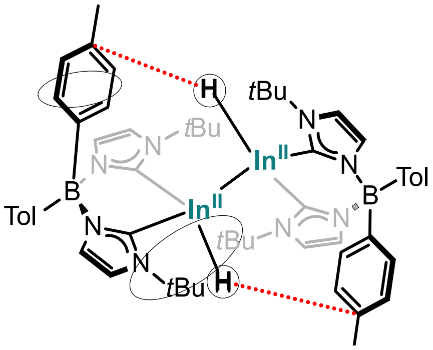

BCP	ρ(r) [e/Bohr³]	∇²ρ(r) [e/Bohr⁵]	V	G	H	ϵ	Delocalization Index
In-H---C	0.006	0.017	-0.0029	0.0036	-0.0007	2.28	0.023

6.6 Topographical analysis of the Electron Localization Function

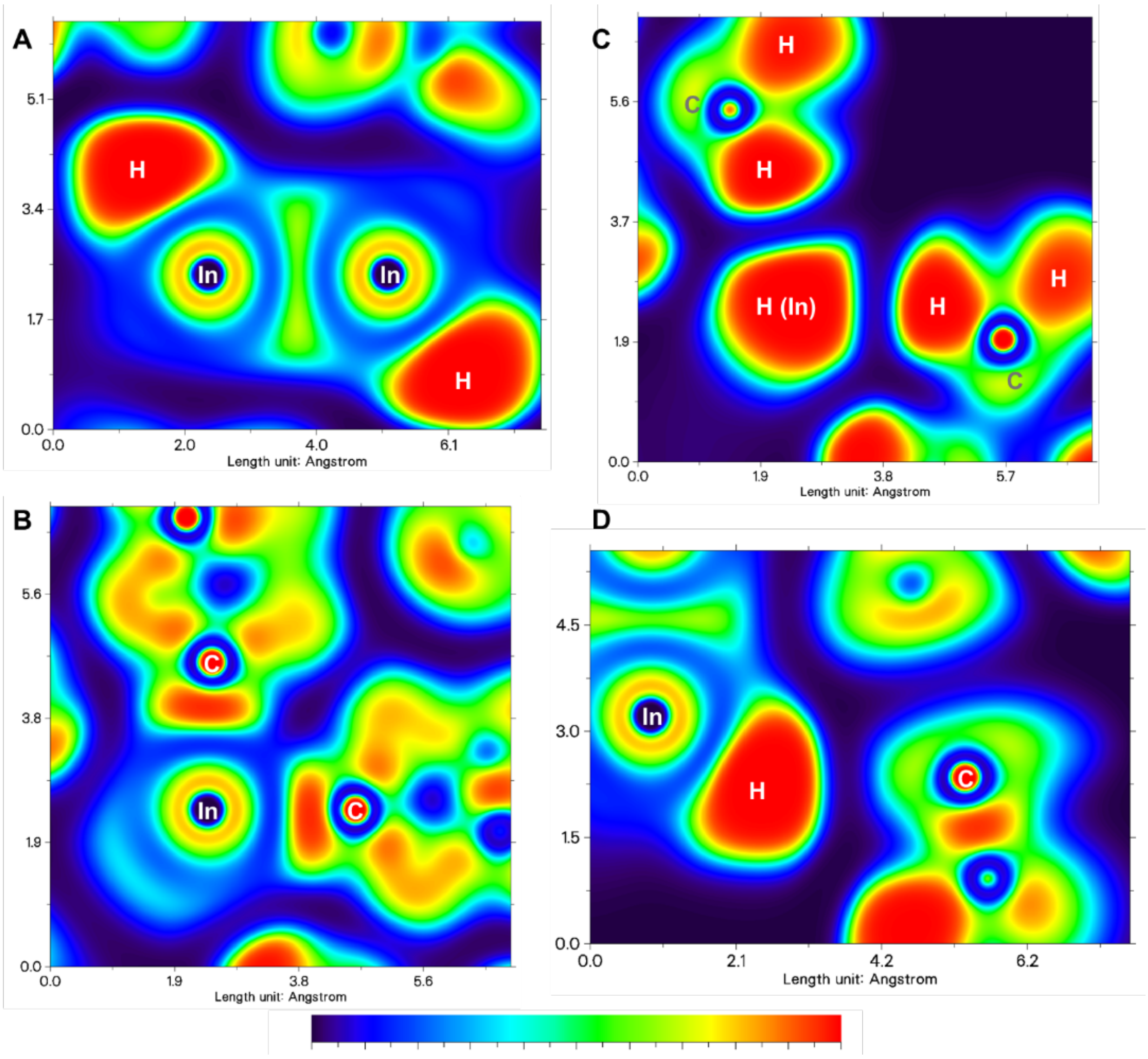

Figure S27. The 2D map of η(r) function for the molecular plane defined by the atoms: (A) H-In-In'-H', (B) C1-In-C2, (C) C_{tBu}-H_a---H_{In}---H_b-C_{tBu} and (D) In-H---CTol. The ELF has been calculated using the PBE0-D4/def2-TZVPP data and topological maps were obtained using Multiwfn software package.[29]

The two-dimensional ELF maps (Figure S27) provide a detailed representation of electron localisation across key molecular planes involving In, C, and H atoms. Regions of high electron localisation (η ≈ 1, shown in red) correspond to domains typical of shared-electron, covalent interactions, whereas regions of low localisation (η ≈ 0, shown in blue) indicate delocalised or ionic character. Covalent bonds, such as the In–In interaction (Figure S27A), are characterised by well-defined localisation domains located between the two atoms, consistent with the presence of a shared electron density [V(In,In) = 1.86]. Conversely, more diffuse or asymmetric localisation domains suggest polarised or closed-shell interactions, as observed for the In–H, In–C bonds and weak In–H···H–C contacts (Figure S27B-D).

6.7 Non-Covalent Interaction Analysis

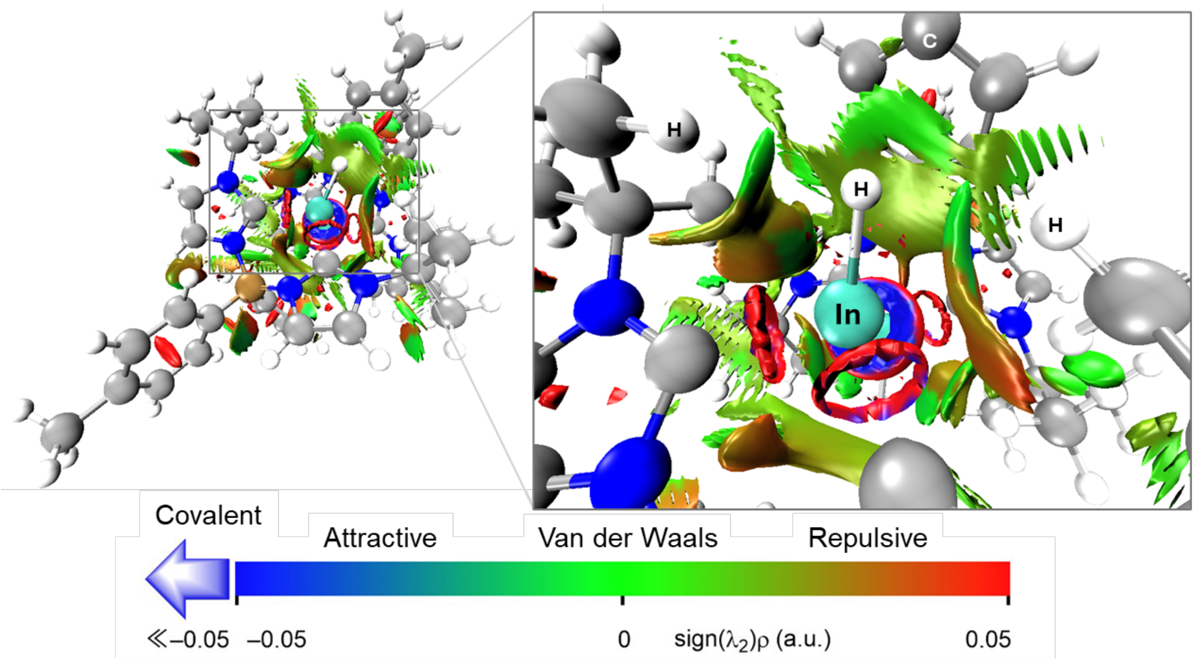

Figure S28. NCIPLOT analysis for **3-H**. Colour key to the qualitative description of NCIPLOT analyses (isovalue = 0.005) performed with Multiwfn software package.[29]

The NCI plot reveals distinct regions of weak attractive interactions surrounding the In–H bond in **3-H**, visualised as green isosurfaces in Figure S28. This qualitative analysis indicates that the In–H bonds are accompanied by subtle attractive Van der Waals contributions (dispersion and electrostatic) from the surrounding ligand environment. The absence of extended red regions along the In–H vector suggests minimal steric repulsion in this region. Instead, the spatial distribution of green van der Waals surfaces between the In–H and adjacent C–H groups of the tBu substituents supports the presence of weak, stabilising In–H···H–C contacts, consistent with QTAIM and ELF analyses as well as experimental observations from 2D ^{1}H NMR and SC-XRD. Collectively, these findings indicate that the stability of the In(II)–H bond in diindane **3-H** arises from the combined effects of steric protection provided by the bis(NHC)borate ligand framework and additional weak non-covalent stabilisation at the ligand periphery.

6.8 Energy Decomposition Analysis (EDA)

Table S9. EDA-NOCV results (in kcal mol^{-1}) at the BP86-D3(BJ)/TZ2P level of theory along the In-In bond.[a]

	**3-H**<br>**LInH (D); LInH (D)**	**NacNac$_2$(In$_2$H$_2$)**<br>**NacNacInH (D); NacNacInH (D)**
ΔE_{int}	-82.4	-85.2
ΔE_{Pauli}	137.1	144.6
ΔE_{disp}[b]	-39.3 (17.9 %)	-37.8 (16.5 %)
ΔE_{elstat}[b]	-115.9 (52.8 %)	-129.5 (56.4 %)
ΔE_{orb}[b]	-64.2 (29.3 %)	-62.4 (27.2 %)
$\Delta E_{orb-\sigma}$[c]	-53.4 (83.1 %)	-62.4 (84.8 %)
$\Delta E_{orb-rest}$[c]	-10.8 (16.9 %)	-9.5 (15.2 %)
ΔE_{prep}	9.1	11.6
D_e	73.3	73.6

[a] Geometries optimized at the PBE0-D3(BJ)/def2-TZVP level of theory. [b] The value in parenthesis gives the percentage contribution to the total attractive interactions ΔE_{elstat} + ΔE_{orb} + ΔE_{disp}. [c] The values in parenthesis gives the percentage contribution to the total orbital interaction.

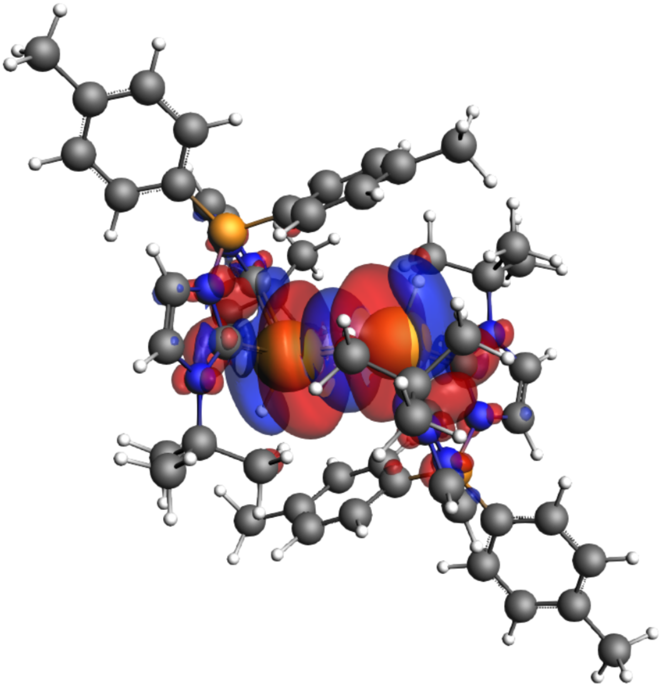

3-H

$\Delta E_{orb\text{-}\sigma}$= -53.4; $|v|_{\alpha}$= 0.48; $|v|_{\beta}$= 0.48

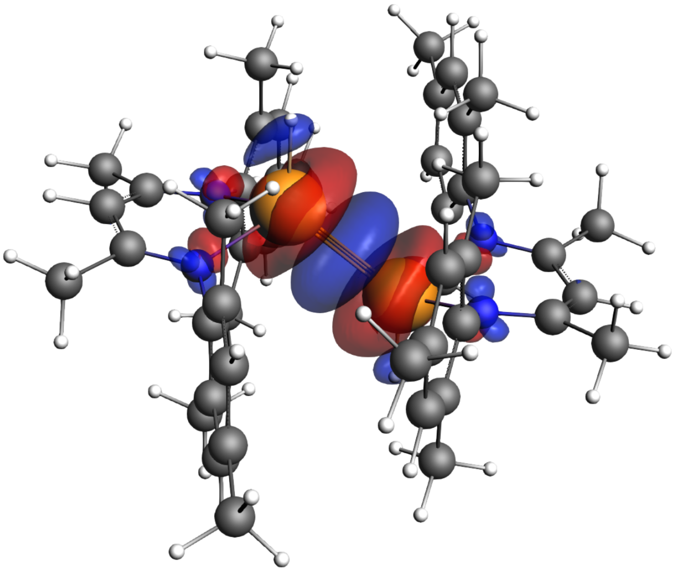

NacNac₂In₂H₂

$\Delta E_{orb\text{-}\sigma}$= -52.9; $|v|_{\alpha}$= 0.46; $|v|_{\beta}$= 0.46

Figure S29. Plot of deformation densities $\Delta\rho$ of the pairwise orbital interactions of **3-H** and **$NacNac_2In_2H_2$** associated energies ΔE in kcal/mol and eigenvalues v in a.u. The red colour shows the charge outflow, whereas blue shows charge density accumulation.

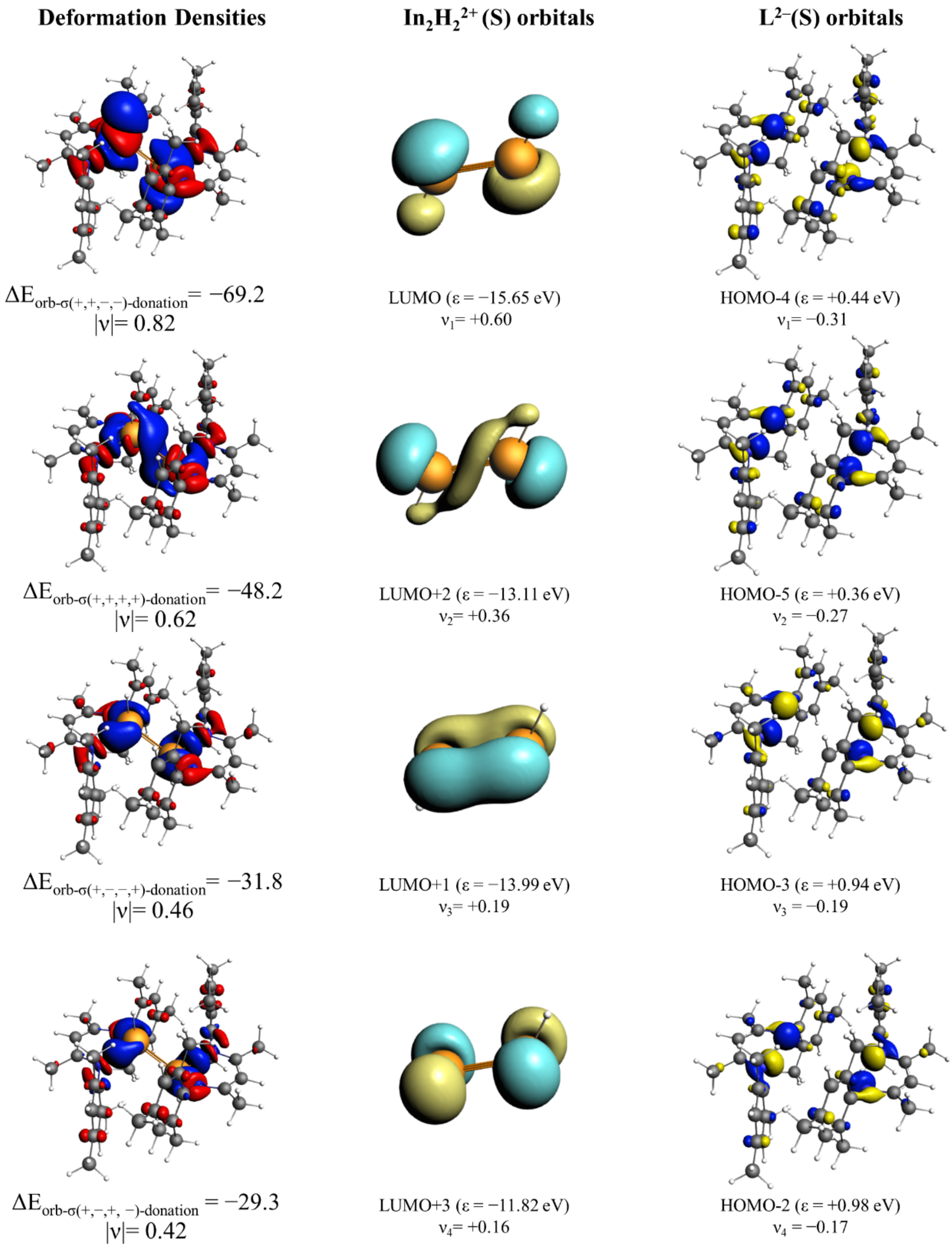

Figure S30. Plot of the first four deformation densities Δρ (isocontour value = 0.001) of the pairwise orbital interactions (isocontour value = 0.03) for **NacNac$_2$In$_2$H$_2$** between [NacNac$_2$]$^{2-}$ and [In$_2$H$_2$]$^{2+}$ in the singlet state, associated energies ΔE in kcal/mol and eigenvalues ν in a.u. The red colour shows the charge outflow, whereas blue shows charge density accumulation.

6.9 Reductive Elimination of Hydrogen from 3-H

The exploration of the potential energy surface (PES) was performed at the PBE0-D4/def2-SVP level of theory, using CPCM with benzene as implicit solvation. All optimized structure energies were later refined at the PBE0-D4/CPCM(C_6H_6)/def2-TZVPP level of theory. The nature of the stationary points was characterized with the normal mode analysis (no imaginary frequencies for local minima and one imaginary frequency for transition states). All transition states were confirmed either by IRC or by optimizing the geometries following the negative eigenvalue of the Hessian. The pathway for hydrogen elimination from In(II) dihydride **3-H** is presented in Figure S31.

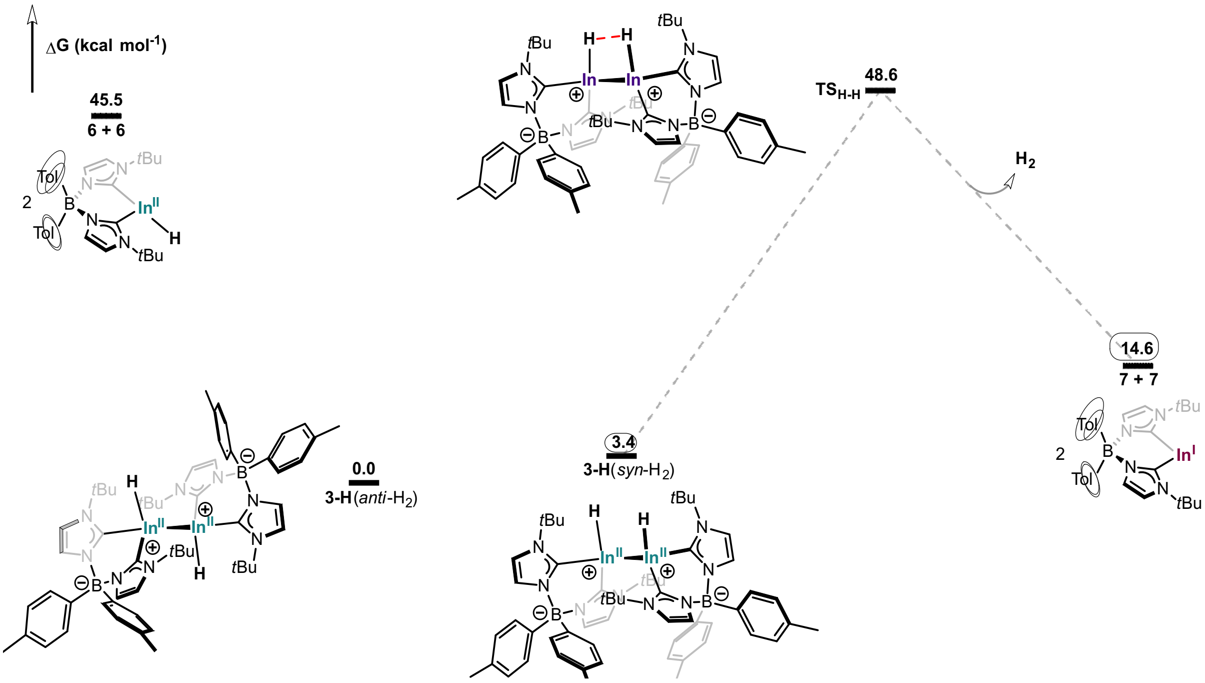

Figure S31. Gibbs energy profile for the elimination of H_2 from **3-H**. Thermodynamic energy differences between **3-H** in *syn* and *anti* isomers, as well as monomeric species 8 after In-In bond dissociation. Thermal free in energy in kcal/mol. Calculated at the PBE0-D4-CPCM(C_6H_6)/def2-TZVPP//PBE0-D4-CPCM(C_6H_6)/def2-SVP level of theory.

6.10 xyz Coordinates (in Å) and Energies (in Hartree)

6.10.1 3-H(-anti-H_2)

Energy(PBE0-D4/def2-SVP) = -3041.922434942444

49	9.045888	7.420836	5.783289
1	7.574988	6.479945	5.905773
7	11.724112	5.974481	5.010691
7	10.763547	7.531258	3.157812
7	10.922465	4.822999	6.669903
7	8.7363	7.99455	2.521799
6	10.708399	5.915267	5.894393
6	12.586969	4.929727	5.243289
1	13.492801	4.785174	4.663728
6	12.088462	4.200745	6.276035
1	12.478696	3.305611	6.745554
6	9.491849	7.634144	3.590371
6	10.8136	7.858966	1.822986
1	11.740433	7.85417	1.258517
6	9.548247	8.14359	1.416543
1	9.182309	8.429859	0.437603
6	9.997323	4.302952	7.711012
6	9.592251	5.437398	8.648154
1	10.481983	5.910186	9.086886
1	8.997585	6.210624	8.143159
1	8.971332	5.033787	9.46099
6	10.709258	3.22931	8.530827
1	10.963449	2.345852	7.927708
1	11.62581	3.618758	8.998787
1	10.034459	2.896742	9.331636
6	8.77683	3.697308	7.018073
1	8.231711	4.464894	6.450295
1	9.084148	2.895	6.330352
1	8.092787	3.271001	7.767086
6	7.247974	8.028956	2.49227
6	6.741062	6.601314	2.702072
1	7.154168	5.929553	1.934342
1	7.03182	6.229477	3.694827
1	5.643346	6.577508	2.633085
6	6.778461	8.545343	1.134838
1	7.169948	9.552527	0.926656
1	7.063927	7.872588	0.313156
1	5.681566	8.606731	1.147541
6	6.706795	8.95663	3.57582
1	7.015931	8.65891	4.585665
1	7.020515	9.995653	3.411255
1	5.608402	8.924309	3.557028

6	13.168716	6.591405	2.95296
6	12.876978	5.500371	2.112639
1	11.884799	5.039006	2.160644
6	13.810349	4.983461	1.221101
1	13.541951	4.132721	0.58651
6	15.096498	5.536194	1.122643
6	15.399301	6.61751	1.952827
1	16.39516	7.06855	1.901597
6	14.454615	7.130291	2.846873
1	14.731926	7.976637	3.481553
6	16.100512	4.980581	0.154131
1	16.256473	3.901689	0.313727
1	15.760894	5.103361	-0.887323
1	17.073625	5.482507	0.251552
6	12.592598	8.428818	4.832027
6	13.12891	8.316449	6.121983
1	13.108597	7.347507	6.630372
6	13.69435	9.405315	6.784915
1	14.094061	9.274949	7.795654
6	13.745334	10.667687	6.186472
6	13.226384	10.79041	4.892205
1	13.254338	11.764922	4.394028
6	12.664278	9.697107	4.237573
1	12.271107	9.837588	3.225746
6	14.279123	11.859874	6.924711
1	14.998719	11.566115	7.703204
1	14.774375	12.571092	6.246592
1	13.458851	12.403688	7.424674
5	12.055125	7.149204	4.000702
49	9.412601	9.877993	6.984718
1	10.917764	10.768276	6.924725
7	6.603843	11.278497	6.884658
7	7.344119	10.51836	9.261095
7	7.696131	12.093472	5.190016
7	9.232791	10.200649	10.287758
6	7.800978	11.273655	6.267047
6	5.734495	12.074861	6.176193
1	4.704034	12.225013	6.48171
6	6.412441	12.5931	5.118118
1	6.074537	13.27086	4.343088
6	8.637424	10.192193	9.068961
6	7.121981	10.712747	10.603803
1	6.143934	10.965567	10.999894
6	8.301121	10.522741	11.25228
1	8.531555	10.591122	12.308893
6	8.831848	12.538288	4.33566

6	9.533857	11.33851	3.705478
1	8.874662	10.811293	3.003857
1	9.890913	10.616995	4.451122
1	10.414133	11.690098	3.149593
6	8.30736	13.434736	3.217684
1	7.856782	14.360235	3.604808
1	7.570081	12.911869	2.589952
1	9.152433	13.719201	2.575651
6	9.801744	13.329648	5.214262
1	10.229027	12.685616	5.995775
1	9.28666	14.177081	5.691619
1	10.627259	13.720723	4.601333
6	10.683191	9.999499	10.545186
6	11.441884	11.229465	10.04726
1	11.08652	12.134746	10.562515
1	11.305551	11.357764	8.964027
1	12.517444	11.113772	10.248068
6	10.913414	9.826918	12.044841
1	10.332994	8.984585	12.450164
1	10.666382	10.736781	12.610762
1	11.978755	9.615731	12.210881
6	11.157079	8.733504	9.835847
1	11.091713	8.80958	8.741957
1	10.565874	7.866509	10.162644
1	12.212913	8.551309	10.08272
6	4.899184	11.260924	8.835709
6	4.990361	12.622027	9.183014
1	5.928384	13.158108	9.002902
6	3.926082	13.313056	9.751416
1	4.04053	14.371598	10.006137
6	2.703235	12.672564	10.00442
6	2.600579	11.321639	9.666066
1	1.660228	10.793576	9.852428
6	3.676217	10.6348	9.095357
1	3.555764	9.576949	8.845672
6	1.555109	13.421041	10.617701
1	1.254783	14.276751	9.991493
1	1.825222	13.828117	11.605549
1	0.676681	12.773149	10.748043
6	5.829789	8.944056	7.832266
6	5.075589	8.622166	6.694901
1	4.79268	9.417144	5.997824
6	4.670607	7.317802	6.420499
1	4.092015	7.108959	5.514883
6	5.005537	6.263074	7.276961
6	5.743956	6.572594	8.423411

1	6.01981	5.770878	9.115973
6	6.14591	7.880861	8.689489
1	6.723808	8.075578	9.598207
6	4.640504	4.846067	6.945256
1	3.705499	4.791105	6.36807
1	4.524377	4.233728	7.851901
1	5.430449	4.377101	6.333143
5	6.175198	10.482544	8.192053

6.10.2 3-H(-syn-H_2)

Energy(PBE0-D4/def2-SVP) = -3041.923275500356

49	13.042402	5.77353	4.600858
49	12.163517	8.222301	5.559852
7	12.518716	5.412505	1.34406
7	14.622756	5.372024	1.889228
7	15.686873	4.555372	6.145164
7	16.070068	5.061414	4.065206
7	11.093386	8.048082	8.697535
7	12.824553	9.338195	8.444753
7	13.776469	10.824403	4.342408
7	14.090304	10.544264	6.475894
6	13.380586	5.528904	2.389685
6	13.228593	5.187097	0.185369
1	12.765481	5.054779	-0.78494
6	14.540523	5.164987	0.53217
1	15.419159	5.006796	-0.084594
6	11.032978	5.390057	1.423438
6	10.604364	4.072421	2.068539
1	10.973431	3.219535	1.478869
1	9.506573	4.015681	2.112119
1	10.99713	3.987908	3.091946
6	10.54006	6.588109	2.227496
1	10.88655	6.570142	3.268473
1	9.441017	6.585577	2.250321
1	10.876956	7.531497	1.774124
6	10.445064	5.48268	0.016715
1	10.786644	6.387859	-0.507095
1	9.351059	5.534488	0.10308
1	10.685154	4.601509	-0.595466
6	15.112295	5.037961	5.012713
6	17.018305	4.297809	5.909153
1	17.694647	3.909228	6.660823
6	17.252977	4.618733	4.610931
1	18.169738	4.55742	4.033652
6	14.975881	4.222177	7.410072

6	14.20174	5.441757	7.898147
1	13.737217	5.216797	8.869157
1	13.396128	5.728968	7.210283
1	14.868013	6.305388	8.024614
6	14.0482	3.037896	7.139959
1	13.527837	2.753101	8.066586
1	14.625343	2.170503	6.784926
1	13.292836	3.291858	6.382901
6	15.992017	3.833916	8.479931
1	16.7035	4.647974	8.674602
1	16.545908	2.921156	8.216266
1	15.449117	3.632384	9.413526
6	16.441741	7.14039	2.501436
6	16.770335	7.888056	3.63789
1	16.707675	7.413845	4.619389
6	17.173289	9.221812	3.560287
1	17.419212	9.767473	4.476212
6	17.273673	9.874411	2.327924
6	16.927969	9.145611	1.182878
1	16.979413	9.630948	0.203206
6	16.522401	7.815559	1.272346
1	16.265064	7.289999	0.347951
6	17.759629	11.292508	2.239223
1	18.861945	11.334033	2.241519
1	17.413605	11.88855	3.097081
1	17.417354	11.781709	1.315342
6	17.090836	4.618438	1.757442
6	18.294256	5.071452	1.207423
1	18.554649	6.130547	1.286485
6	19.184132	4.20576	0.564651
1	20.117487	4.60023	0.150853
6	18.90592	2.842365	0.443562
6	17.705317	2.374152	0.998484
1	17.461786	1.309165	0.92883
6	16.82704	3.242075	1.63776
1	15.902439	2.837362	2.063349
6	19.842949	1.908924	-0.267257
1	19.457612	1.644722	-1.266487
1	19.968575	0.966077	0.287
1	20.835275	2.361907	-0.40492
6	11.988025	8.512389	7.784375
6	11.378371	8.583078	9.934315
1	10.803874	8.368096	10.827165
6	12.460481	9.38555	9.770008
1	12.990324	9.994163	10.495523
6	9.902161	7.202846	8.412612

6	9.281737	6.734473	9.727052
1	8.442663	6.064658	9.493493
1	8.880804	7.569717	10.319182
1	10.002578	6.172741	10.339744
6	8.886448	8.04638	7.642892
1	7.980702	7.454318	7.444454
1	9.300941	8.379886	6.680905
1	8.601281	8.932782	8.229774
6	10.321126	5.973031	7.614735
1	11.067578	5.382897	8.16566
1	10.747396	6.229009	6.636205
1	9.441937	5.339039	7.431921
6	13.44138	10.047393	5.40481
6	14.658189	11.80015	4.749062
1	15.075785	12.54491	4.082407
6	14.853307	11.618601	6.080364
1	15.471356	12.176869	6.775975
6	13.200067	10.725285	2.973485
6	11.723248	11.11048	3.043624
1	11.1657	10.424221	3.696676
1	11.279353	11.067069	2.037837
1	11.609409	12.135186	3.429096
6	13.931013	11.688177	2.042303
1	13.792544	12.738106	2.338693
1	13.515975	11.572127	1.031761
1	15.004605	11.461081	1.992547
6	13.385756	9.307427	2.444745
1	14.444326	9.017197	2.464474
1	13.029604	9.251917	1.405832
1	12.819711	8.565922	3.023339
6	15.394916	9.003303	8.156631
6	15.696804	8.455608	9.414507
1	15.0663	8.697476	10.275181
6	16.786927	7.611094	9.612601
1	16.987168	7.211059	10.611536
6	17.635467	7.266358	8.552832
6	17.329372	7.78144	7.289766
1	17.961824	7.516365	6.437405
6	16.237717	8.630869	7.103307
1	16.035789	9.015553	6.101386
6	18.846712	6.404951	8.766582
1	19.744775	7.022631	8.936562
1	19.051249	5.774556	7.888284
1	18.731409	5.751753	9.644236
6	14.333773	11.389601	8.880804
6	15.490117	11.710281	9.598365

1	16.333544	11.014272	9.59471
6	15.604787	12.903449	10.318731
1	16.527694	13.116923	10.866873
6	14.5616	13.831025	10.35144
6	13.397375	13.523019	9.630616
1	12.562455	14.231088	9.63227
6	13.293085	12.335779	8.915392
1	12.370221	12.133184	8.361399
6	14.671978	15.114959	11.122118
1	13.884212	15.190355	11.889034
1	14.557762	15.988982	10.460643
1	15.644679	15.198611	11.627393
5	16.04347	5.570564	2.566599
5	14.165765	10.044147	7.975655
1	11.955821	4.441855	5.041045
1	10.573115	8.670812	4.915728

6.10.3 TS$_{H-H}$

Energy(PBE0-D4/def2-SVP) = --3041.846381883584

49	13.133882	5.853211	4.496735
49	12.182581	8.289165	5.619245
7	12.647419	5.293611	1.217359
7	14.727259	5.118032	1.814694
7	15.604694	4.240857	6.045214
7	16.105544	4.80055	4.007961
7	11.136199	8.177537	8.802756
7	12.777754	9.567335	8.504053
7	13.561707	11.07175	4.42183
7	14.004584	10.757488	6.524032
6	13.495966	5.430165	2.270114
6	13.351651	4.900402	0.101613
1	12.894941	4.724042	-0.864868
6	14.652245	4.797291	0.481177
1	15.526556	4.52175	-0.099585
6	11.171729	5.474331	1.257115
6	10.56526	4.332895	2.071457
1	10.828859	3.361567	1.626203
1	9.469161	4.424376	2.0884
1	10.924049	4.361654	3.110659
6	10.850167	6.8372	1.864243
1	11.196688	6.929367	2.90199
1	9.760824	6.98669	1.87336
1	11.308446	7.640262	1.268965
6	10.611564	5.435122	-0.162743
1	11.061456	6.211575	-0.799261

1	9.530851	5.626064	-0.111277
1	10.750952	4.454351	-0.640346
6	15.090684	4.738939	4.891653
6	16.953679	4.012728	5.889774
1	17.588584	3.610131	6.670329
6	17.261325	4.366469	4.613501
1	18.210834	4.324448	4.089009
6	14.831206	3.99725	7.291216
6	14.43841	5.33881	7.90386
1	13.903742	5.175718	8.85082
1	13.779352	5.910301	7.235339
1	15.326503	5.950883	8.107118
6	13.598146	3.161459	6.951785
1	13.034862	2.948034	7.871931
1	13.887008	2.207365	6.485911
1	12.923389	3.694075	6.264443
6	15.69868	3.228404	8.283993
1	16.569976	3.817407	8.605951
1	16.044503	2.269459	7.869807
1	15.097322	3.013892	9.178173
6	16.435538	6.939305	2.500478
6	16.760837	7.682008	3.641103
1	16.746226	7.19157	4.617592
6	17.103338	9.034044	3.572271
1	17.351458	9.578833	4.487855
6	17.135469	9.710421	2.349335
6	16.792772	8.984239	1.201453
1	16.790571	9.487675	0.22956
6	16.448378	7.637002	1.280852
1	16.187584	7.110992	0.356935
6	17.533548	11.156227	2.273112
1	18.630004	11.265118	2.21906
1	17.197373	11.709681	3.162569
1	17.113688	11.644674	1.381205
6	17.241428	4.46948	1.734234
6	18.410735	5.006394	1.186857
1	18.591332	6.082311	1.260247
6	19.36405	4.205236	0.551023
1	20.267359	4.665229	0.138314
6	19.186644	2.825063	0.433903
6	18.020771	2.272416	0.985797
1	17.854558	1.192513	0.917876
6	17.080194	3.076198	1.619822
1	16.186592	2.605969	2.044251
6	20.195521	1.95775	-0.262026
1	19.787479	1.546887	-1.200375

1	20.486226	1.098537	0.362854
1	21.105714	2.521202	-0.512163
6	12.017276	8.644278	7.878622
6	11.350494	8.80727	10.007974
1	10.771253	8.607288	10.901359
6	12.379917	9.672353	9.814911
1	12.855272	10.354392	10.512457
6	10.064028	7.175691	8.557969
6	9.403135	6.798563	9.881641
1	8.647005	6.026872	9.681466
1	8.889138	7.652291	10.346843
1	10.128087	6.382255	10.596878
6	9.015964	7.79567	7.63557
1	8.191249	7.085244	7.476727
1	9.449383	8.036455	6.653841
1	8.605629	8.713926	8.082635
6	10.684712	5.920366	7.951101
1	11.42877	5.491193	8.637598
1	11.173574	6.11452	6.987114
1	9.900041	5.171109	7.771902
6	13.254136	10.305725	5.500543
6	14.526396	11.991473	4.767374
1	14.93487	12.721841	4.078975
6	14.800273	11.788869	6.083096
1	15.487899	12.312198	6.740179
6	12.951034	10.937973	3.073049
6	11.430442	10.982443	3.216206
1	11.054584	10.131464	3.804439
1	10.964031	10.933747	2.221323
1	11.112179	11.913464	3.708723
6	13.404362	12.100542	2.193897
1	13.127602	13.073978	2.625574
1	12.909968	12.011451	1.216818
1	14.489753	12.079673	2.018094
6	13.410423	9.626145	2.442327
1	14.506538	9.574767	2.398164
1	13.017077	9.546068	1.418239
1	13.052798	8.756414	3.011109
6	15.314447	9.158216	8.160773
6	15.590045	8.573525	9.408403
1	14.961994	8.821325	10.269795
6	16.647303	7.686136	9.594662
1	16.823139	7.253041	10.584379
6	17.485098	7.328748	8.530478
6	17.210235	7.888691	7.279355
1	17.8395	7.618011	6.426542

6	16.149194	8.779252	7.103192
1	15.96594	9.188844	6.106769
6	18.638017	6.386096	8.723049
1	19.574073	6.935316	8.921046
1	18.806542	5.773043	7.825056
1	18.469388	5.712083	9.57604
6	14.362882	11.580055	8.92034
6	15.53886	11.838606	9.631335
1	16.344505	11.099344	9.621217
6	15.72116	13.023381	10.35125
1	16.658503	13.188253	10.891584
6	14.728416	14.004582	10.392182
6	13.545426	13.759768	9.677966
1	12.749926	14.511836	9.683903
6	13.374566	12.580792	8.961442
1	12.4402	12.430028	8.410238
6	14.908405	15.275235	11.171631
1	14.206317	15.326028	12.020207
1	14.716094	16.16061	10.545098
1	15.92755	15.358507	11.575233
5	16.122354	5.348757	2.525906
5	14.121299	10.247195	8.014738
1	11.101495	5.934482	4.845028
1	10.742549	6.965636	4.99559

6.10.4 H_2

Energy(PBE0-D4/def2-SVP) = -1.160979389728

1	-1.34964	-0.00558	-4.749208
1	-0.699946	0.294435	-4.46881

6.10.5 6

Energy(PBE0-D4/def2-SVP) = -1520.905792546152

49	9.33562	7.511637	5.734019
1	7.723308	7.007751	6.258801
7	11.833706	5.822197	4.966675
7	10.787828	7.147465	3.00553
7	11.077693	4.841079	6.752072
7	8.77423	7.73821	2.442001
6	10.843252	5.852688	5.880288
6	12.697564	4.799346	5.270426
1	13.583639	4.59169	4.679155
6	12.228128	4.176428	6.384997
1	12.628585	3.327557	6.926537
6	9.57317	7.456196	3.500526
6	10.756808	7.246625	1.636544

1	11.629455	7.056997	1.019814
6	9.497011	7.611742	1.275252
1	9.079542	7.785705	0.290427
6	10.211129	4.486841	7.906144
6	10.081175	5.703865	8.820891
1	11.072383	6.039728	9.160169
1	9.585925	6.547089	8.318883
1	9.478988	5.441418	9.702791
6	10.848016	3.344421	8.691949
1	10.940224	2.430913	8.086412
1	11.839887	3.618736	9.080716
1	10.20363	3.110344	9.550573
6	8.850606	4.038975	7.374021
1	8.353961	4.84855	6.820933
1	8.967283	3.174334	6.703449
1	8.199171	3.746024	8.210618
6	7.334507	8.094637	2.526886
6	6.57037	6.8997	3.095677
1	6.708937	6.01555	2.455386
1	6.913698	6.656541	4.110902
1	5.496144	7.131331	3.143376
6	6.80298	8.41894	1.134009
1	7.337285	9.26555	0.67787
1	6.861038	7.552687	0.458719
1	5.744431	8.6997	1.22339
6	7.180319	9.328673	3.414716
1	7.514905	9.139088	4.444375
1	7.763607	10.170342	3.012457
1	6.121985	9.624159	3.456682
6	13.274582	6.393848	2.873758
6	13.127807	5.194735	2.150871
1	12.207529	4.611276	2.261898
6	14.117195	4.721154	1.295347
1	13.962429	3.782749	0.753426
6	15.314637	5.429886	1.116197
6	15.472895	6.620789	1.829104
1	16.397447	7.194584	1.712525
6	14.47388	7.088986	2.686716
1	14.635954	8.02556	3.227725
6	16.376103	4.924617	0.182068
1	16.631836	3.874787	0.395341
1	16.035749	4.964227	-0.865867
1	17.295748	5.522059	0.257654
6	12.480228	8.314947	4.602106
6	13.167369	8.335976	5.82486
1	13.411002	7.390927	6.321007

6	13.545729	9.52881	6.438239
1	14.076635	9.500861	7.395019
6	13.255099	10.766502	5.852099
6	12.583905	10.758155	4.623767
1	12.349116	11.709205	4.135499
6	12.208598	9.560558	4.017325
1	11.680776	9.60075	3.058975
6	13.623448	12.054557	6.530475
1	14.533806	11.944394	7.138214
1	13.790369	12.861258	5.801452
1	12.819006	12.388537	7.207706
5	12.103018	6.924563	3.861101

6.10.6 7

Energy(PBE0-D4/def2-SVP) = -1520.345649653606

6	-3.207202	-0.288069	-2.19255
7	-2.510194	-0.236449	-1.010191
6	-3.01324	0.736454	-0.226137
6	-4.155872	0.68799	-2.152003
1	-4.895689	0.972596	-2.891874
1	-2.984454	-1.008834	-2.97357
7	-4.01597	1.313701	-0.928692
6	-4.856929	2.417678	-0.410004
49	-2.181545	0.835344	2.012189
7	-0.104141	-0.262025	-0.197009
6	-0.164903	0.705634	0.73756
6	1.911989	0.628187	-0.097037
7	1.067551	1.262311	0.793153
1	2.954027	0.895558	-0.233168
1	1.45333	-1.053024	-1.471821
6	1.16711	-0.331533	-0.712374
6	1.436319	2.354496	1.723344
5	-1.344534	-1.183248	-0.522234
6	-1.81933	-1.887699	0.86289
6	-0.967352	-2.252613	-1.67819
6	-1.056637	-3.636109	-1.471997
6	-0.731415	-4.556106	-2.468734
6	-0.299869	-4.134034	-3.730676
6	-0.201874	-2.754651	-3.951713
6	-0.529058	-1.842734	-2.94996
6	0.020269	-5.123221	-4.813965
1	0.692859	-4.691635	-5.569463
1	-0.895613	-5.446932	-5.336997
1	0.494813	-6.02807	-4.405642
1	-0.812525	-5.627738	-2.261127
1	-1.388862	-4.008378	-0.49864

1	0.138956	-2.390608	-4.926066
1	-0.435767	-0.77388	-3.169344
6	-0.89775	-2.265524	1.855727
6	-1.291674	-2.929895	3.015086
6	-2.635702	-3.25158	3.241852
6	-3.56312	-2.893514	2.257307
6	-3.161995	-2.22831	1.099379
1	-3.918978	-1.963223	0.354964
1	0.161917	-2.029475	1.717839
1	-0.541613	-3.202438	3.763745
6	-3.066284	-3.927599	4.510665
1	-4.619932	-3.136739	2.402939
1	-2.32164	-4.664451	4.847074
1	-4.030633	-4.441071	4.386736
1	-3.186555	-3.193262	5.324935
6	1.419593	1.803423	3.149075
1	0.415691	1.441633	3.417781
1	2.127504	0.966568	3.247285
1	1.705975	2.589075	3.86389
6	0.427688	3.49199	1.567516
1	0.398648	3.84628	0.526352
1	-0.585805	3.17287	1.853649
1	0.70996	4.332739	2.217953
6	-3.943205	3.536005	0.089637
1	-4.548866	4.386619	0.434893
1	-3.32464	3.200557	0.935471
1	-3.275499	3.881005	-0.71362
6	-5.723176	1.875726	0.72672
1	-6.36708	1.059162	0.366649
1	-5.098417	1.489316	1.545921
1	-6.363929	2.674283	1.128895
6	-5.746759	2.956289	-1.525875
1	-5.152669	3.321897	-2.376715
1	-6.45629	2.197397	-1.886524
1	-6.333377	3.799161	-1.134791
6	2.831232	2.871008	1.385117
1	3.60129	2.09889	1.528716
1	2.885724	3.241294	0.350478
1	3.071331	3.706508	2.057347

6.10.7 3-H(*anti-H*$_2$)

Energy(PBE0-D3(BJ)/def2-TZVPP) = -3044.70820589

49	-0.717814	0.441517	-0.985994
1	0.037685	0.950766	-2.468697
7	-3.651177	1.355486	-0.366013
7	-3.417771	-1.149088	-0.999014
7	-2.593982	3.181271	-0.849973

7	-2.137381	-2.21533	-2.382876
6	-2.444231	1.84309	-0.70499
6	-4.552484	2.388106	-0.279654
1	-5.579315	2.230443	-0.00451
6	-3.900657	3.531386	-0.586611
1	-4.262636	4.5407	-0.630035
6	-2.199575	-1.134427	-1.56811
6	-4.109969	-2.253635	-1.433061
1	-5.106962	-2.473071	-1.096786
6	-3.31905	-2.920533	-2.302704
1	-3.508474	-3.821157	-2.854758
6	-1.550533	4.117639	-1.330059
6	-0.2868	3.949174	-0.496962
1	-0.502513	4.123577	0.557832
1	0.159109	2.961488	-0.608314
1	0.458726	4.676319	-0.822948
6	-2.04297	5.550004	-1.172253
1	-2.905325	5.758354	-1.807019
1	-2.300132	5.77736	-0.136
1	-1.242234	6.22409	-1.476284
6	-1.275164	3.834296	-2.802622
1	-0.887981	2.824068	-2.936227
1	-2.189375	3.946259	-3.388596
1	-0.532336	4.538333	-3.182491
6	-1.061989	-2.484709	-3.36944
6	-1.094097	-1.381147	-4.422616
1	-2.079272	-1.329537	-4.890078
1	-0.860839	-0.415076	-3.974577
1	-0.352674	-1.587091	-5.196842
6	-1.311149	-3.830441	-4.036337
1	-1.355353	-4.640904	-3.306156
1	-2.228382	-3.832579	-4.627036
1	-0.482579	-4.035623	-4.714118
6	0.296653	-2.529767	-2.686006
1	0.540527	-1.601707	-2.173042
1	0.353172	-3.349431	-1.971044
1	1.067711	-2.68579	-3.441496
6	-5.605145	-0.279563	0.073265
6	-6.347843	-0.087021	-1.098489
1	-5.828637	0.157996	-2.020666
6	-7.726773	-0.195548	-1.121744
1	-8.264464	-0.036385	-2.051526
6	-8.439267	-0.505874	0.036584
6	-7.715892	-0.696661	1.205642
1	-8.243996	-0.935274	2.123413
6	-6.329511	-0.585582	1.220884

1	-5.799352	-0.739834	2.153876
6	-9.931738	-0.637187	0.008734
1	-10.398901	0.232234	-0.459607
1	-10.240483	-1.515511	-0.564847
1	-10.338934	-0.738282	1.015285
6	-3.350096	-0.419786	1.517172
6	-3.120129	0.59565	2.445086
1	-3.290262	1.628531	2.158139
6	-2.682804	0.32647	3.732878
1	-2.511995	1.145332	4.425006
6	-2.444336	-0.97698	4.151969
6	-2.68162	-1.999593	3.240788
1	-2.505381	-3.02807	3.541221
6	-3.122974	-1.723847	1.956559
1	-3.296812	-2.551132	1.275329
6	-1.8867	-1.265055	5.51056
1	-2.181921	-0.504289	6.235222
1	-2.21576	-2.237084	5.882167
1	-0.792444	-1.28036	5.475437
5	-3.991674	-0.128143	0.069283
49	0.732079	-0.246876	1.242916
1	0.113843	-0.730485	2.799326
7	3.377361	-1.54213	0.140259
7	3.657545	0.866694	1.08489
7	2.117376	-3.216002	0.685468
7	2.654769	2.19868	2.46386
6	2.193326	-1.862327	0.689986
6	4.033656	-2.690122	-0.233312
1	5.001255	-2.670445	-0.700947
6	3.254781	-3.739631	0.109684
1	3.425713	-4.792251	-0.010392
6	2.464105	1.128716	1.656394
6	4.588532	1.771554	1.534036
1	5.608732	1.757507	1.19663
6	3.971462	2.601861	2.399904
1	4.359892	3.429814	2.963613
6	1.078768	-4.020721	1.374548
6	-0.312007	-3.620245	0.905838
1	-0.44031	-3.797128	-0.160907
1	-0.541218	-2.577743	1.114859
1	-1.050897	-4.218684	1.440452
6	1.288133	-5.495656	1.060114
1	2.232313	-5.871006	1.457429
1	1.25165	-5.688391	-0.013956
1	0.484812	-6.063449	1.529394
6	1.21943	-3.794087	2.876674

1	1.026836	-2.750605	3.126784
1	2.224076	-4.06082	3.210326
1	0.499188	-4.414136	3.413769
6	1.684561	2.881244	3.347024
6	2.062098	2.577996	4.793919
1	3.070925	2.923928	5.024814
1	2.014471	1.503728	4.977422
1	1.3671	3.075461	5.472326
6	1.761194	4.38161	3.071797
1	1.548025	4.588705	2.022093
1	2.738318	4.800418	3.312697
1	1.020563	4.8983	3.683362
6	0.266843	2.408992	3.086768
1	0.115591	1.37275	3.38316
1	-0.010355	2.511378	2.037377
1	-0.415219	3.024616	3.675071
6	5.562916	-0.235851	-0.269531
6	6.318419	-0.795014	0.768561
1	5.811322	-1.145739	1.662886
6	7.694778	-0.913348	0.695194
1	8.243297	-1.350841	1.523838
6	8.391122	-0.475743	-0.431435
6	7.654688	0.083269	-1.466678
1	8.170309	0.436725	-2.354028
6	6.271003	0.199455	-1.385069
1	5.730306	0.643786	-2.213112
6	9.880137	-0.619684	-0.518699
1	10.169679	-1.668644	-0.627474
1	10.369836	-0.243393	0.382306
1	10.28103	-0.075142	-1.374281
6	3.288086	0.529328	-1.467705
6	3.064151	-0.255832	-2.59887
1	3.258232	-1.322856	-2.55078
6	2.604482	0.283715	-3.789291
1	2.434566	-0.363811	-4.644333
6	2.342427	1.644322	-3.905646
6	2.574139	2.441107	-2.79121
1	2.381552	3.507975	-2.852971
6	3.03243	1.893841	-1.602398
1	3.199659	2.554352	-0.757514
6	1.769	2.216785	-5.164199
1	2.130142	1.687163	-6.047618
1	2.018777	3.273454	-5.273846
1	0.677118	2.136286	-5.158199
5	3.952996	-0.09523	-0.137953

6.10.8 7

Energy(PBE0-D3(BJ)/def2-TZVPP) = -1522.30047964

49	-2.139933	0.000095	0.425033
1	-3.756181	0.000081	-0.276637
7	0.484392	1.289667	-0.6166
7	0.484329	-1.290077	-0.616056
7	-1.002354	2.830649	-0.932072
7	-1.002515	-2.831045	-0.931031
6	-0.827624	1.561498	-0.495723
6	1.13365	2.38877	-1.117147
1	2.195659	2.400118	-1.282452
6	0.209843	3.355531	-1.321551
1	0.326628	4.352003	-1.702632
6	-0.827712	-1.561707	-0.49522
6	1.13354	-2.389443	-1.116093
1	2.195568	-2.400958	-1.281284
6	0.209659	-3.356198	-1.3202
1	0.326399	-4.352885	-1.700738
6	-2.303722	3.531226	-1.017573
6	-2.957203	3.5442	0.359186
1	-2.300876	4.01549	1.092334
1	-3.194047	2.5394	0.707687
1	-3.890701	4.107252	0.314015
6	-2.081203	4.967909	-1.468075
1	-1.647092	5.018721	-2.467835
1	-1.442588	5.516295	-0.773425
1	-3.047465	5.470985	-1.501876
6	-3.181394	2.813537	-2.036438
1	-3.3798	1.786377	-1.730427
1	-2.697145	2.800921	-3.014571
1	-4.137595	3.331267	-2.130252
6	-2.303953	-3.531486	-1.016421
6	-3.181593	-2.813801	-2.035301
1	-2.697353	-2.801235	-3.013441
1	-3.380023	-1.786625	-1.729368
1	-4.137778	-3.331569	-2.129099
6	-2.081616	-4.968228	-1.466836
1	-1.443036	-5.516664	-0.772188
1	-1.64755	-5.019151	-2.46661
1	-3.047934	-5.471204	-1.500523
6	-2.957325	-3.544236	0.360391
1	-3.193461	-2.539322	0.709053
1	-2.301201	-4.016024	1.093406
1	-3.89117	-4.106707	0.315253
6	2.736596	-0.000271	-0.585848
6	3.031134	-0.000461	-1.955283

1	2.216176	-0.000521	-2.67375
6	4.331554	-0.000999	-2.428108
1	4.515995	-0.001514	-3.498092
6	5.412548	-0.001202	-1.546806
6	5.134706	-0.001349	-0.186803
1	5.956331	-0.002108	0.52233
6	3.825154	-0.0008	0.280696
1	3.647114	-0.001104	1.350165
6	6.820774	0.000676	-2.058901
1	7.02429	0.893883	-2.655183
1	7.010696	-0.863402	-2.700518
1	7.539593	-0.025385	-1.239433
6	1.091512	0.000292	1.551223
6	1.079228	1.186486	2.286012
1	1.095196	2.137638	1.76304
6	1.042371	1.190915	3.671635
1	1.030759	2.135778	4.206067
6	1.016551	0.001145	4.391952
6	1.042003	-1.188995	3.672359
1	1.030075	-2.133505	4.207393
6	1.078867	-1.18542	2.286671
1	1.094551	-2.136894	1.764286
6	0.934804	0.001745	5.88806
1	1.42067	0.881779	6.312801
1	1.406302	-0.886095	6.312641
1	-0.106362	0.010311	6.224056
5	1.210041	-0.000091	-0.057925

6.10.9 $(NacNac)_2In_2H_2$

Energy(PBE0-D3(BJ)/def2-TZVPP) = -2386.91454623

49	-0.001654	1.050143	0.883005
7	-1.512929	2.570111	0.386442
7	1.507555	2.572132	0.386792
6	-1.268524	3.855603	0.569194
6	-0.003892	4.413049	0.800914
1	-0.004631	5.476412	0.989087
6	1.261534	3.857298	0.569533
6	-2.408014	4.832537	0.460014
1	-3.258819	4.520436	1.066689
1	-2.764576	4.880294	-0.571671
1	-2.092895	5.828388	0.762628
6	2.39985	4.835632	0.460796
1	2.083546	5.830972	0.763866
1	2.756352	4.884303	-0.570866
1	3.251014	4.524243	1.067334
6	-2.802585	2.197452	-0.073006
6	-3.054656	2.172749	-1.449406

6	-4.341463	1.894165	-1.888477
1	-4.53766	1.882527	-2.956312
6	-5.380391	1.647191	-0.999889
6	-5.089894	1.632132	0.35607
1	-5.879983	1.411006	1.065376
6	-1.956533	2.454895	-2.425847
1	-1.496838	3.430793	-2.252917
1	-2.33103	2.427341	-3.44887
1	-1.155729	1.716382	-2.338341
6	-3.811429	1.882245	0.839276
6	-3.525101	1.835856	2.307511
1	-4.425913	1.585101	2.867285
1	-3.140344	2.788191	2.680508
1	-2.764492	1.086356	2.538706
6	2.797563	2.200914	-0.072767
6	5.375719	1.652754	-0.999837
6	5.085203	1.636847	0.356118
1	5.875395	1.415789	1.065321
6	3.806587	1.885938	0.839406
6	1.951397	2.459132	-2.425453
1	1.15048	1.720817	-2.337526
1	2.325679	2.431231	-3.448544
1	1.491923	3.435186	-2.252737
6	4.336686	1.899553	-1.888334
1	4.532925	1.888493	-2.956165
6	3.049683	2.177121	-1.449161
6	3.520254	1.838792	2.307617
1	3.136032	2.791138	2.681165
1	4.420951	1.58725	2.867217
1	2.759239	1.089601	2.538462
49	0.001577	-1.050544	-0.882963
7	1.512847	-2.570469	-0.38638
7	-1.507555	-2.572559	-0.386648
6	1.268504	-3.856009	-0.568881
6	0.003883	-4.413551	-0.80041
1	0.004633	-5.47695	-0.988383
6	-1.26154	-3.857757	-0.569119
6	2.408053	-4.832855	-0.459585
1	3.258863	-4.520758	-1.066253
1	2.764573	-4.880506	0.572121
1	2.093002	-5.828752	-0.762121
6	-2.399883	-4.836037	-0.460217
1	-2.083638	-5.831422	-0.763204
1	-2.756338	-4.884592	0.571467
1	-3.251056	-4.524661	-1.066752
6	2.802516	-2.197604	0.072871

6	3.054728	-2.172553	1.449217
6	4.341514	-1.893459	1.888086
1	4.537815	-1.881405	2.955894
6	5.380252	-1.646381	0.999334
6	5.089606	-1.631741	-0.35661
1	5.879536	-1.410439	-1.066033
6	1.956792	-2.454767	2.425845
1	1.497428	-3.430885	2.253266
1	2.33137	-2.426764	3.448826
1	1.155716	-1.716571	2.338179
6	3.811182	-1.882333	-0.83961
6	3.524661	-1.836295	-2.30782
1	4.425268	-1.585091	-2.867721
1	3.140444	-2.788918	-2.680644
1	2.763571	-1.087298	-2.539057
6	-2.79751	-2.201155	0.072884
6	-5.375431	-1.651824	0.999919
6	-5.084959	-1.636385	-0.35607
1	-5.875091	-1.415113	-1.06527
6	-3.80648	-1.886054	-0.83934
6	-1.951354	-2.459001	2.425624
1	-1.150556	-1.720519	2.337931
1	-2.325754	-2.431333	3.448679
1	-1.491688	-3.434936	2.252791
6	-4.336478	-1.898794	1.888422
1	-4.532646	-1.887296	2.95626
6	-3.04958	-2.176981	1.449261
6	-3.520124	-1.839202	-2.307557
1	-3.135376	-2.791446	-2.680802
1	-4.420922	-1.588279	-2.867274
1	-2.759491	-1.089668	-2.538577
6	-6.771182	1.400049	-1.499124
1	-7.426198	1.061481	-0.695934
1	-7.206998	2.309849	-1.920608
1	-6.783191	0.644375	-2.28818
6	6.766756	1.407019	-1.499064
1	7.203841	2.31875	-1.915069
1	7.420668	1.063504	-0.697074
1	6.778804	0.655698	-2.292234
6	6.771134	-1.399058	1.498215
1	7.209868	-2.31062	1.912868
1	7.424138	-1.053554	0.696334
1	6.782381	-0.648708	2.292309
6	-6.766462	-1.405759	1.49899
1	-7.418541	-1.055665	0.698349
1	-7.206332	-2.319055	1.908632

1	-6.777616	-0.659457	2.296862
1	-0.002222	0.978797	2.630125
1	0.001922	-0.979113	-2.630081

6.10.10 NacNacInH

Energy(PBE0-D3(BJ)/def2-TZVPP) = -1193.40369384

49	-0.000005	0.740946	0.722356
7	1.510942	-0.596757	-0.162146
7	-1.510943	-0.596805	-0.162082
6	1.271568	-1.785977	-0.681642
6	0.000012	-2.341949	-0.875281
1	0.000019	-3.335041	-1.29948
6	-1.271555	-1.786024	-0.681578
6	2.441644	-2.615104	-1.13515
1	2.981839	-2.104077	-1.935022
1	3.156294	-2.757144	-0.322779
1	2.11591	-3.587021	-1.497926
6	-2.441627	-2.615203	-1.135001
1	-2.115875	-3.587095	-1.497825
1	-3.156195	-2.757295	-0.322566
1	-2.981924	-2.104191	-1.934814
6	2.841493	-0.11249	-0.080229
6	3.597352	-0.33781	1.074185
6	4.878049	0.196773	1.147235
1	5.4653	0.023152	2.043473
6	5.420884	0.948232	0.113286
6	4.639554	1.170136	-1.01403
1	5.037807	1.767873	-1.827936
6	3.033461	-1.133818	2.209769
1	2.769077	-2.14904	1.903433
1	3.74931	-1.200741	3.028635
1	2.114812	-0.680294	2.593224
6	3.354466	0.656671	-1.13009
6	2.524738	0.926222	-2.346544
1	3.073864	1.539104	-3.060915
1	2.226588	0.0021	-2.847908
1	1.601663	1.448717	-2.082258
6	-2.841503	-0.112575	-0.080099
6	-5.420913	0.948102	0.113395
6	-4.639572	1.169965	-1.014037
1	-5.037851	1.767626	-1.827999
6	-3.354559	0.656558	-1.130067
6	-3.033445	-1.133833	2.20989
1	-2.114731	-0.680388	2.593281
1	-3.749272	-1.200608	3.028789
1	-2.769197	-2.149117	1.903634
6	-4.878081	0.196738	1.147289

1	-5.465349	0.023105	2.043501
6	-3.597285	-0.337852	1.074261
6	-2.524811	0.926002	-2.346531
1	-2.226594	0.001834	-2.84777
1	-3.073949	1.538772	-3.060987
1	-1.601771	1.448573	-2.082274
6	6.815391	1.489517	0.202166
1	7.534574	0.795872	-0.243125
1	6.90775	2.438398	-0.328329
1	7.114395	1.648645	1.239028
6	-6.815272	1.489815	0.202017
1	-6.902382	2.448523	-0.311547
1	-7.532126	0.805809	-0.261538
1	-7.121593	1.630301	1.239385
1	-0.000069	2.239684	-0.208513

7 Crystallographic Data

7.1 Single crystal structure analysis of 2-Br

CCDC Deposition number 2503900

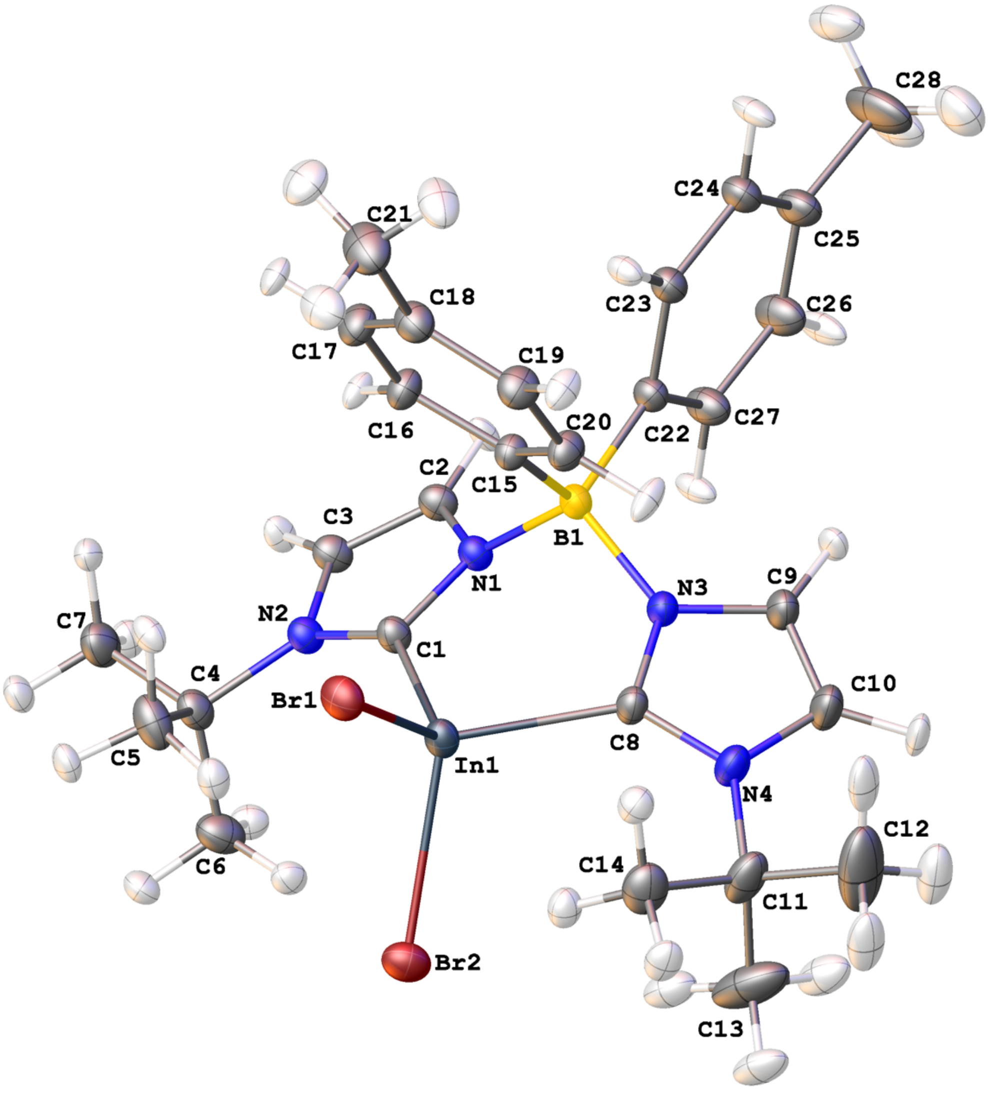

Figure S32. Olex-2 representation of the molecular structure of ligand **2-Br**.

7.1.1 Experimental

Single colourless block-shaped crystals of **2-Br** were recrystallised from hexane by slow evaporation. A suitable crystal with dimensions 0.10 × 0.06 × 0.05 mm^3 was selected and mounted on a MITIGEN holder in oil on a Rigaku 007HF diffractometer with HF Varimax confocal mirrors, an UG2 goniometer and HyPix Arc-100 detector diffractometer. The crystal was kept at a steady T = 100(2) K during data collection. The structure was solved with the ShelXT 2018/2[30] solution program using dual methods and by using Olex2 1.5-dev[31] as the graphical interface. The model was refined with olex2.refine 1.5-dev[32] using full matrix least squares minimisation on $\boldsymbol{F}^2$.

7.1.2 Crystal Data

$C_{28}H_{36}BN_4Br_2In$, M_r = 714.074, monoclinic, $P2_1/c$ (No. 14), a = 11.5167(1) Å, b = 14.2721(1) Å, c = 19.3948(2) Å, b = 107.253(1)°, $\alpha = \gamma$ = 90°, V = 3044.43(5) Å^3, T = 100(2) K, Z = 4, Z' = 1, μ(Cu K_α) = 9.443, 59002 reflections measured, 5737 unique (R_{int} = 0.0253) which were used in all calculations. The final wR_2 was 0.0331 (all data) and R_1 was 0.0144 (I≥2 σ(I)).

**Compound**	**2-Br**
Formula	$C_{28}H_{36}BN_4Br_2In$
$D_{calc.}$/ g cm^{-3}	1.558
m/mm^{-1}	9.445
Formula Weight	714.074
Colour	colourless
Shape	block-shaped
Size/mm	0.10×0.06×0.05
T/K	100(2)
Crystal System	monoclinic
Space Group	$P2_1/c$
a/Å	11.5167(1)
b/Å	14.2721(1)
c/Å	19.3948(2)
a/°	90
b/°	107.253(1)
g/°	90
V/Å^3	3044.43(5)
Z	4
Z'	1
Wavelength/Å	1.54184
Radiation type	Cu K_a
Q_{min}/°	3.91
Q_{max}/°	71.68
Index range h	-10 ≥ h ≥ 13
Index range k	-17 ≥ k ≥ 17
Index range l	-23 ≥ l ≥ 23
Measured Refl's.	59002
Indep't Refl's	5737
Refl's I≥2*s*(I)	5601
R_{int}	0.0253
Parameters	369
Restraints	318
Largest Peak/eÅ^3	0.3235
Deepest Hole/eÅ^3	-0.4050
GooF	1.0583
R_1 (I≥2*s*(I) / all)	0.0144 / 0.0150
wR_2 (I≥2*s*(I) / all)	0.0332 / 0.0334

7.1.3 Structure Quality Indicators

Reflections:	d min (CuKα) 2Θ=143.4° 0.81	I/σ(I) 75.8	Rint m=10.54 2.53%	Full 135.4° 96% to 143.4° 99.1
Refinement:	Shift 0.001	Max Peak 0.3	Min Peak -0.4	GooF 1.058

A colourless block-shaped crystal with dimensions 0.10 × 0.06 × 0.05 mm was mounted on a MITIGEN holder in oil. Data were collected using a Rigaku 007HF diffractometer with HF Varimax confocal mirrors, an UG2 goniometer and HyPix Arc-100 detector diffractometer equipped with an Oxford Cryosystems low-temperature device operating at T = 100(2) K.

Data were measured using profile data from ω-scans with Cu K_{α} radiation. The diffraction pattern was indexed and the total number of runs and images was based on the strategy calculation from the program CrysAlis^{Pro} system (CCD 43.136a 64-bit (release 23-08-2024)). The maximum resolution achieved was Θ = 71.68° (0.81 Å).

The unit cell was refined using CrysAlis^{Pro} on 44951 reflections, 76% of the observed reflections.

Data reduction, scaling and absorption corrections were performed using CrysAlis^{Pro}. The final completeness is 99.11 % out to 71.68° in Θ. A gaussian absorption correction was performed using CrysAlis^{Pro} 1.171.43.135a (Rigaku Oxford Diffraction, 2024). Numerical absorption correction based on gaussian integration over a multifaceted crystal model. Empirical absorption correction using spherical harmonics, implemented in SCALE3 ABSPACK scaling algorithm. The absorption coefficient μ of this material is 9.445 mm^{-1} at this wavelength (λ = 1.54184Å) and the minimum and maximum transmissions are 0.513 and 0.768.

The structure was solved in the space group $P2_1/c$ (# 14) by ShelXT 2018/2 using dual methods.[30] It was refined by full matrix least squares minimisation on $|F|^2$ using version of olex2.refine 1.5-dev.[32] All non-hydrogen atoms were refined anisotropically.

Hydrogen atom positions were calculated geometrically and refined using the riding model.

_*olex2_refine_details*: Refinement using NoSpherA2, an implementation of NOn-SPHERical Atom-form-factors in Olex2.[33] 2021 NoSpherA2 implementation of HAR makes use of tailor-made aspherical atomic form factors calculated on-the-fly from a Hirshfeld-partitioned electron density (ED) - not from spherical-atom form factors. The ED is calculated from a gaussian basis set single determinant SCF wavefunction - either Hartree-Fock or DFT using selected functionals- for a fragment of the crystal. This fragment can be embedded in an electrostatic crystal field by employing cluster charges or modelled using implicit solvation models, depending on the software used. The following options were used: SOFTWARE: ORCA 6.0 ::: PARTITIONING: NoSpherA2 ::: INT ACCURACY: Normal ::: METHOD: R2SCAN ::: BASIS SET: x2c-TZVP ::: CHARGE: 0 ::: MULTIPLICITY: 1 ::: RELATIVISTIC: ZORA ::: DATE: 2024-12-29_01-32-48

_*exptl_absorpt_process_details*: CrysAlis^{Pro} 1.171.43.135a (Rigaku Oxford Diffraction, 2024). Numerical absorption correction based on gaussian integration over a multifaceted crystal model. Empirical absorption correction using spherical harmonics, implemented in SCALE3 ABSPACK scaling algorithm.

There is a single formula unit in the asymmetric unit, which is represented by the reported sum formula. In other words: Z is 4 and Z' is 1. The moiety formula is C28 H36 B Br2 In N4.

7.1.4 Data Plots: Diffraction Data

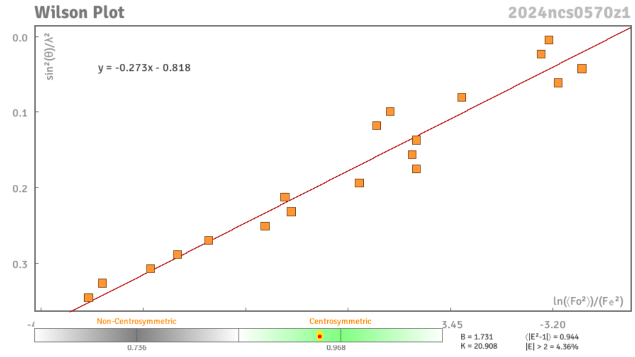

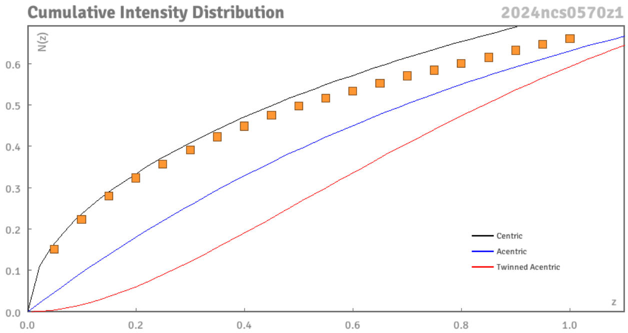

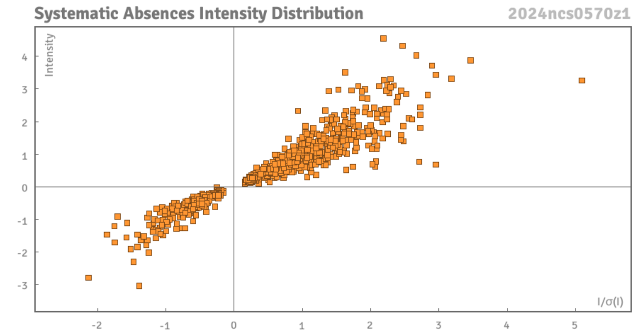

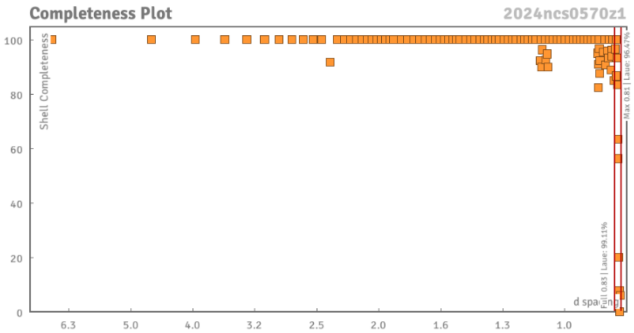

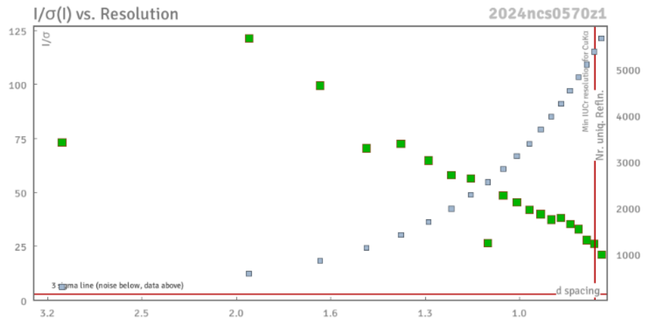

7.1.5 Data Plots: Refinement and Data

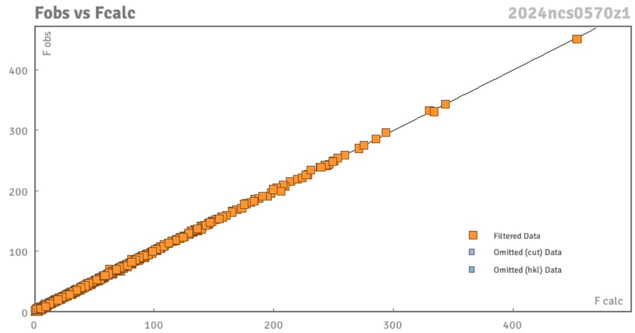

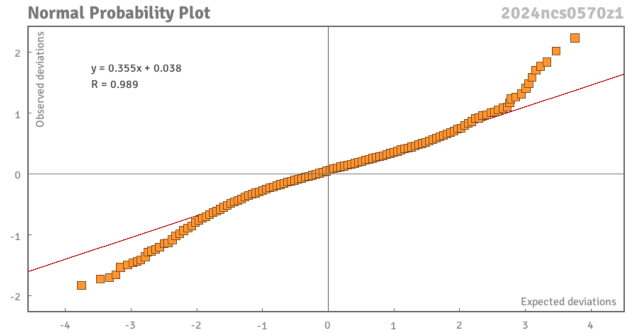

7.1.6 Reflection Statistics

Total reflections (after filtering)	60462	Unique reflections	5737
Completeness	0.965	Mean I/*s*	52.98
hkl_{max} collected	(13, 17, 23)	hkl_{min} collected	(-10, -17, -23)
hkl_{max} used	(13, 17, 23)	hkl_{min} used	(-13, 0, 0)
Lim d_{max} collected	100.0	Lim d_{min} collected	0.77
d_{max} used	18.52	d_{min} used	0.81
Friedel pairs	3765	Friedel pairs merged	1
Inconsistent equivalents	1	R_{int}	0.0253
R_{sigma}	0.0132	Intensity transformed	0
Omitted reflections	0	Omitted by user (OMIT hkl)	0
Multiplicity	(3811, 3092, 2298, 1297, 891, 667, 621, 468, 393, 329, 273, 191, 140, 90, 87, 65, 32, 28, 18, 21, 30, 26, 20, 17, 9, 5, 1, 1)	Maximum multiplicity	54
Removed systematic absences	1460	Filtered off (Shel/OMIT)	0

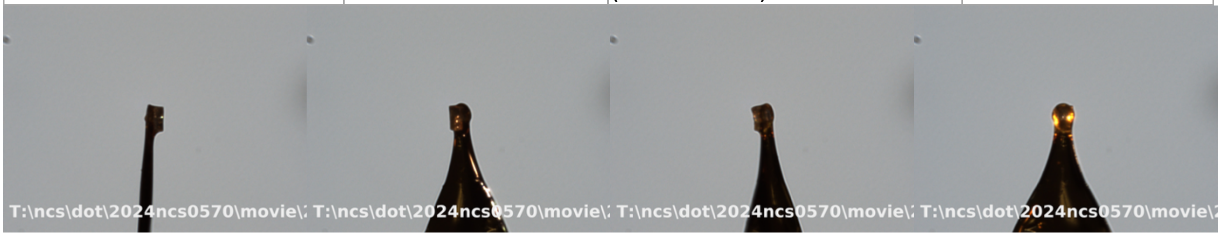

7.1.7 Fractional Atomic Coordinates and Equivalent Isotropic Displacement Parameters

Table S10. Fractional Atomic Coordinates (×10^4) and Equivalent Isotropic Displacement Parameters (Å^2×10^3) for **2-Br**. U_{eq} is defined as 1/3 of the trace of the orthogonalised U_{ij}.

Atom	x	y	z	U_{eq}
In1	6417.95(8)	7362.99(6)	3559.86(5)	18.54(3)
Br1	7872.02(13)	7824.15(10)	2885.57(8)	25.68(4)
Br2	5103.77(13)	8808.05(10)	3527.34(9)	26.84(4)
N1	5528.5(9)	5387.2(7)	3656.9(6)	16.4(2)
N2	4179.9(9)	5947.2(8)	2699.6(6)	18.3(2)
N3	7089.8(10)	6020.9(8)	4813.6(6)	17.8(2)
N4	7612.2(11)	7373.6(8)	5341.9(7)	23.3(3)
C1	5231.8(11)	6144.1(9)	3219.8(7)	17.3(3)
C2	4652.3(11)	4709.0(9)	3410.8(7)	18.8(3)
C3	3809.3(12)	5056.9(9)	2816.9(8)	19.9(3)
C4	3505.1(12)	6566.6(10)	2087.6(8)	21.4(3)
C5	4411.1(13)	7124.8(12)	1814.6(9)	29.6(3)
C6	2672.6(13)	7221.2(10)	2349.0(9)	29.1(3)
C7	2747.4(13)	5949.9(11)	1473.8(8)	28.9(3)
C8	7202.9(12)	6945.4(9)	4690.4(7)	19.2(3)
C9	7414.7(13)	5872.7(10)	5546.1(8)	25.3(3)
C10	7732.5(15)	6713.1(11)	5877.7(8)	29.8(3)
C11	7892.2(16)	8386.6(11)	5499.6(9)	33.6(4)
C12	8979(2)	8464.3(17)	6175.4(11)	65.4(7)
C13	6780(2)	8846.0(13)	5627.9(14)	57.8(6)
C14	8229.1(15)	8847.1(11)	4877.3(9)	31.7(3)
C15	7814.8(11)	5345.7(9)	3754.3(7)	16.9(3)
C16	7543.8(12)	4962.6(9)	3057.7(7)	20.4(3)
C17	8422.0(12)	4876.6(10)	2701.8(8)	23.5(3)
C18	9610.4(12)	5180.3(10)	3024.4(8)	23.2(3)
C19	9885.6(12)	5591.9(10)	3706.7(8)	24.1(3)
C20	9004.0(12)	5666.2(10)	4064.4(8)	20.8(3)
C21	10584.5(14)	5054.8(13)	2658.5(9)	34.7(4)
C22	6986.7(12)	4211.9(9)	4593.2(7)	18.5(3)
C23	7984.3(12)	3640.4(9)	4605.7(7)	20.5(3)
C24	8209.6(13)	2796.1(10)	4984.5(8)	24.2(3)
C25	7437.2(15)	2476.3(10)	5362.8(9)	28.9(3)
C26	6423.0(15)	3025.6(11)	5348.0(9)	31.1(3)
C27	6206.5(13)	3873.2(10)	4971.8(8)	25.4(3)
C28	7706(2)	1575.9(13)	5782.1(12)	52.3(5)
B1	6847.1(13)	5232.5(10)	4208.4(8)	16.8(3)

7.1.8 Anisotropic Displacement and Structural Parameters

Table S11. Anisotropic Displacement Parameters (×10^4) for **2-Br**. The anisotropic displacement factor exponent takes the form: $-2p^2[h^2a^{*2} \times U_{11} + \ldots +2hka^* \times b^* \times U_{12}]$

Atom	U_{11}	U_{22}	U_{33}	U_{23}	U_{13}	U_{12}
In1	21.19(5)	16.59(5)	17.20(5)	-2.86(3)	4.79(4)	1.98(3)
Br1	28.17(8)	24.68(8)	27.19(8)	0.26(6)	12.87(6)	6.58(6)
Br2	27.04(8)	19.18(7)	34.09(9)	0.14(5)	8.85(6)	0.76(6)
N1	16.9(5)	14.6(5)	17.3(6)	-0.4(4)	4.6(4)	1.9(4)
N2	16.9(5)	17.2(5)	19.5(6)	-0.5(4)	3.6(4)	0.8(4)
N3	22.7(5)	16.4(5)	13.9(5)	1.5(4)	4.8(4)	0.6(4)
N4	32.3(7)	20.6(6)	16.6(6)	-2.4(5)	6.8(5)	-3.3(5)
C1	17.6(6)	15.8(6)	17.2(6)	-1.6(5)	3.2(5)	1.5(5)
C2	18.9(6)	15.5(6)	22.1(7)	-1.4(5)	6.1(5)	1.5(5)
C3	17.2(6)	17.8(6)	23.7(7)	-1.3(5)	4.6(5)	0.7(5)
C4	19.6(7)	20.5(7)	20.6(7)	0.4(5)	1.5(5)	2.2(5)
C5	26.1(8)	35.5(8)	24.8(8)	-1.6(6)	3.7(6)	11.0(7)
C6	24.9(7)	23.4(7)	35.5(9)	3.9(6)	3.6(6)	0.9(6)
C7	29.5(8)	26.8(8)	23.8(8)	2.1(6)	-2.1(6)	0.3(6)
C8	25.4(7)	17.3(6)	14.4(7)	-1.5(5)	5.4(5)	-0.1(5)
C9	39.1(8)	20.4(7)	15.5(7)	3.3(6)	6.4(6)	1.7(5)
C10	48.9(9)	24.6(8)	14.4(7)	0.0(6)	7.1(6)	-1.3(6)
C11	51.9(10)	25.0(8)	23.2(8)	-12.1(7)	12.1(7)	-7.6(6)
C12	96.9(17)	57.1(14)	28.7(10)	-41.6(13)	-4.0(10)	-3.4(9)
C13	97.5(16)	23.5(9)	76.4(16)	-5.8(9)	59.8(14)	-12.2(9)
C14	39.0(9)	25.0(8)	29.6(9)	-10.8(6)	7.5(7)	-2.5(6)
C15	16.4(6)	18.3(6)	15.7(6)	-0.5(5)	4.3(5)	-0.7(5)
C16	21.0(7)	23.8(7)	16.4(7)	-2.2(5)	5.8(5)	-3.5(5)
C17	25.9(7)	27.3(7)	19.1(7)	1.3(5)	9.1(6)	-2.1(6)
C18	22.6(7)	26.1(7)	23.1(7)	3.4(5)	10.4(6)	3.6(6)
C19	18.5(7)	27.7(7)	25.9(8)	-0.4(5)	6.1(6)	-0.8(6)
C20	18.5(6)	24.4(7)	18.6(7)	-0.7(5)	4.2(5)	-3.0(6)
C21	31.2(8)	46.1(10)	32.5(9)	9.2(7)	17.6(7)	8.6(8)
C22	22.4(7)	15.6(6)	17.6(7)	1.6(5)	6.3(5)	2.0(5)
C23	23.0(7)	17.8(6)	19.7(7)	3.0(5)	4.9(5)	1.9(5)
C24	29.7(8)	18.2(7)	23.8(7)	5.1(5)	7.0(6)	3.6(6)
C25	41.5(9)	18.1(7)	29.7(8)	7.7(6)	14.9(7)	7.2(6)
C26	43.1(9)	22.0(7)	36.1(9)	6.1(6)	23.3(7)	10.1(7)
C27	31.5(8)	20.4(7)	28.4(8)	5.9(6)	15.2(6)	6.9(6)
C28	76.8(14)	29.7(9)	65.3(14)	21.9(9)	41.6(12)	26.8(9)
B1	19.4(7)	15.5(7)	15.1(7)	0.8(5)	4.4(5)	1.9(5)

Table S12. Bond Lengths in Å for **2-Br**.

Atom	Atom	Length/Å
In1	Br1	2.49939(17)
In1	Br2	2.54787(17)
In1	C1	2.1893(13)
In1	C8	2.1916(14)
N1	C1	1.3533(17)
N1	C2	1.3772(16)
N1	B1	1.5920(18)
N2	C1	1.3559(16)
N2	C3	1.3801(17)
N2	C4	1.4998(17)
N3	C8	1.3543(17)
N3	C9	1.3732(17)
N3	B1	1.5902(18)

Atom	Atom	Length/Å
N4	C8	1.3562(18)
N4	C10	1.3781(19)
N4	C11	1.4931(18)
C2	C3	1.3594(19)
C4	C5	1.528(2)
C4	C6	1.529(2)
C4	C7	1.528(2)
C9	C10	1.358(2)
C11	C12	1.527(3)
C11	C13	1.525(3)
C11	C14	1.521(2)
C15	C16	1.4044(18)
C15	C20	1.3984(18)

Atom	Atom	Length/Å
C15	B1	1.620(2)
C16	C17	1.388(2)
C17	C18	1.394(2)
C18	C19	1.395(2)
C18	C21	1.507(2)
C19	C20	1.392(2)
C22	C23	1.4037(18)

Atom	Atom	Length/Å
C22	C27	1.4034(19)
C22	B1	1.6223(19)
C23	C24	1.3943(19)
C24	C25	1.387(2)
C25	C26	1.399(2)
C25	C28	1.506(2)
C26	C27	1.396(2)

Table S13. Bond Angles in ° for **2-Br**.

Atom	Atom	Atom	Angle/°
Br2	In1	Br1	105.021(6)
C1	In1	Br1	120.43(4)
C1	In1	Br2	108.83(3)
C8	In1	Br1	116.97(3)
C8	In1	Br2	108.09(4)
C8	In1	C1	96.94(5)
C2	N1	C1	108.66(11)
B1	N1	C1	123.03(11)
B1	N1	C2	126.20(11)
C3	N2	C1	108.64(11)
C4	N2	C1	127.28(11)
C4	N2	C3	124.08(11)
C9	N3	C8	108.75(12)
B1	N3	C8	124.35(11)
B1	N3	C9	126.09(11)
C10	N4	C8	108.86(12)
C11	N4	C8	128.38(12)
C11	N4	C10	122.75(12)
N1	C1	In1	115.23(9)
N2	C1	In1	136.80(10)
N2	C1	N1	107.72(11)
C3	C2	N1	107.66(12)
C2	C3	N2	107.32(12)
C5	C4	N2	109.50(11)
C6	C4	N2	109.05(12)
C6	C4	C5	110.90(12)
C7	C4	N2	108.58(11)
C7	C4	C5	108.80(13)
C7	C4	C6	109.98(12)
N3	C8	In1	114.13(9)
N4	C8	In1	137.40(10)
N4	C8	N3	107.41(12)
C10	C9	N3	107.82(13)

Atom	Atom	Atom	Angle/°
C9	C10	N4	107.14(13)
C12	C11	N4	108.38(15)
C13	C11	N4	107.86(13)
C13	C11	C12	110.46(18)
C14	C11	N4	110.23(12)
C14	C11	C12	108.26(15)
C14	C11	C13	111.60(15)
C20	C15	C16	116.73(12)
B1	C15	C16	119.44(11)
B1	C15	C20	122.90(12)
C17	C16	C15	121.71(13)
C18	C17	C16	120.88(13)
C19	C18	C17	118.10(13)
C21	C18	C17	121.36(14)
C21	C18	C19	120.53(13)
C20	C19	C18	120.74(13)
C19	C20	C15	121.78(13)
C27	C22	C23	115.93(13)
B1	C22	C23	119.93(12)
B1	C22	C27	123.98(12)
C24	C23	C22	122.30(13)
C25	C24	C23	121.05(13)
C26	C25	C24	117.73(14)
C28	C25	C24	120.34(15)
C28	C25	C26	121.92(15)
C27	C26	C25	121.01(14)
C26	C27	C22	121.96(13)
N3	B1	N1	108.75(10)
C15	B1	N1	107.11(11)
C15	B1	N3	109.37(11)
C22	B1	N1	112.23(11)
C22	B1	N3	108.88(11)
C22	B1	C15	110.45(11)

Table S14. Torsion Angles in ° for **2-Br**.

Atom	Atom	Atom	Atom	Angle/°
In1	C1	N1	C2	175.06(9)
In1	C1	N1	B1	-20.56(12)
In1	C1	N2	C3	-173.26(14)
In1	C1	N2	C4	7.11(17)
In1	C8	N3	C9	-169.57(10)
In1	C8	N3	B1	20.23(12)
In1	C8	N4	C10	165.70(15)
In1	C8	N4	C11	-13.51(18)
N1	C1	N2	C3	0.45(12)
N1	C1	N2	C4	-179.17(10)
N1	C2	C3	N2	0.42(12)
N1	B1	N3	C8	-66.42(13)
N1	B1	N3	C9	125.08(11)
N1	B1	C15	C16	-40.57(13)
N1	B1	C15	C20	150.77(10)
N1	B1	C22	C23	130.05(11)
N1	B1	C22	C27	-54.77(14)
N2	C1	N1	C2	-0.19(12)
N2	C1	N1	B1	164.19(10)
N3	C8	N4	C10	-1.26(13)
N3	C8	N4	C11	179.53(11)
N3	C9	C10	N4	-0.74(13)
N3	B1	N1	C1	66.15(13)
N3	B1	N1	C2	-132.28(11)
N3	B1	C15	C16	-158.26(10)
N3	B1	C15	C20	33.07(13)
N3	B1	C22	C23	-109.50(11)
N3	B1	C22	C27	65.68(13)
N4	C8	N3	C9	0.80(13)
N4	C8	N3	B1	-169.41(10)
C1	N1	C2	C3	-0.15(12)
C1	N1	B1	C15	-51.95(13)
C1	N1	B1	C22	-173.33(13)
C1	N2	C3	C2	-0.55(12)
C1	N2	C4	C5	36.24(15)
C1	N2	C4	C6	-85.27(14)
C1	N2	C4	C7	154.90(15)
C2	N1	B1	C15	109.63(13)
C2	N1	B1	C22	-11.75(13)
C2	C3	N2	C4	179.09(10)
C3	N2	C4	C5	-143.34(14)
C3	N2	C4	C6	95.16(14)
C3	N2	C4	C7	-24.67(14)
C3	C2	N1	B1	-163.91(10)
C8	N3	C9	C10	-0.03(13)
C8	N3	B1	C15	50.24(13)
C8	N3	B1	C22	171.01(13)
C8	N4	C10	C9	1.25(13)
C8	N4	C11	C12	-144.58(19)
C8	N4	C11	C13	95.81(18)
C8	N4	C11	C14	-26.26(16)
C9	N3	B1	C15	-118.26(14)
C9	N3	B1	C22	2.51(13)
C9	C10	N4	C11	-179.50(12)
C10	N4	C11	C12	36.31(18)
C10	N4	C11	C13	-83.29(17)
C10	N4	C11	C14	154.64(16)
C10	C9	N3	B1	169.96(11)

Atom	Atom	Atom	Atom	Angle/˚
C15	C16	C17	C18	-0.74(16)
C15	C20	C19	C18	-0.80(16)
C15	B1	C22	C23	10.61(13)
C15	B1	C22	C27	-174.21(11)
C16	C15	C20	C19	-1.33(15)
C16	C15	B1	C22	81.92(13)
C16	C17	C18	C19	-1.43(17)
C16	C17	C18	C21	177.40(14)
C17	C16	C15	C20	2.09(16)
C17	C16	C15	B1	-167.26(13)
C17	C18	C19	C20	2.18(16)
C19	C20	C15	B1	167.62(13)
C20	C15	B1	C22	-86.74(13)
C20	C19	C18	C21	-176.66(14)
C22	C23	C24	C25	0.86(17)
C22	C27	C26	C25	0.00(18)
C23	C22	C27	C26	1.09(16)
C23	C24	C25	C26	0.31(18)
C23	C24	C25	C28	-178.38(17)
C24	C23	C22	C27	-1.52(16)
C24	C23	C22	B1	174.04(13)
C24	C25	C26	C27	-0.72(19)
C26	C27	C22	B1	-174.26(14)
C27	C26	C25	C28	177.94(18)

Table S15. Hydrogen Fractional Atomic Coordinates (×10^4) and Equivalent Isotropic Displacement Parameters (Å^2×10^3) for **2-Br**. U_{eq} is defined as 1/3 of the trace of the orthogonalised U_{ij}.

Atom	x	y	z	U_{eq}
H2	4636.8(11)	4023.6(9)	3646.1(8)	34(4)
H3	2998.8(12)	4701.3(9)	2496.3(8)	39(5)
H5a	4865(8)	7630(6)	2217(3)	54(6)
H5b	5074(6)	6654.4(15)	1713(6)	44(5)
H5c	3937(2)	7482(7)	1323(4)	51(6)
H6a	2031(7)	6812.5(11)	2528(6)	55(6)
H6b	3211.8(18)	7640(6)	2790(4)	49(6)
H6c	2190(8)	7673(6)	1913(2)	60(6)
H7a	3332(2)	5454(5)	1322(4)	44(5)
H7b	2074(7)	5578(6)	1652(2)	55(6)
H7c	2302(8)	6381.4(14)	1017(2)	55(6)
H9	7418.3(13)	5205.4(10)	5811.7(8)	46(5)
H10	8023.9(15)	6840.3(11)	6453.5(8)	56(6)
H12a	8726(5)	8223(11)	6636.8(17)	113(10)
H12b	9713(6)	8032(9)	6112(4)	69(8)
H12c	9277(9)	9179(2)	6255(5)	96(9)
H13a	6011(4)	8770(10)	5149(3)	92(9)
H13b	6576(9)	8510(8)	6075(6)	91(9)
H13c	6954(6)	9577(3)	5743(9)	98(9)
H14a	8922(8)	8438(5)	4747(4)	54(6)
H14b	7437(3)	8883(8)	4412(2)	58(6)
H14c	8567(10)	9543(3)	5032(3)	62(6)
H16	6626.3(12)	4728.4(10)	2790.7(7)	36(4)
H17	8181.3(13)	4570.3(10)	2167.7(8)	42(5)
H19	10792.4(12)	5856.0(10)	3960.8(8)	41(5)
H20	9245.4(12)	5980.2(10)	4595.8(8)	42(5)
H21a	10295(7)	4536(9)	2237(7)	111(10)
H21b	10741(12)	5710(3)	2426(8)	87(8)
H21c	11413(5)	4825(12)	3047(2)	108(10)
H23	8600.7(12)	3861.7(10)	4310.7(8)	37(5)
H24	8998.0(13)	2384.8(10)	4983.6(8)	48(5)
H26	5798.4(15)	2790.7(11)	5632.7(9)	53(6)
H27	5414.0(14)	4280.4(10)	4971.8(8)	39(5)
H28a	6932(8)	1371(9)	5948(11)	100(9)
H28b	7920(20)	1035(4)	5453(4)	166(16)
H28c	8479(13)	1678(5)	6257(7)	220(20)

7.2 Single crystal structure analysis of 2-I

CCDC Deposition number 2503901

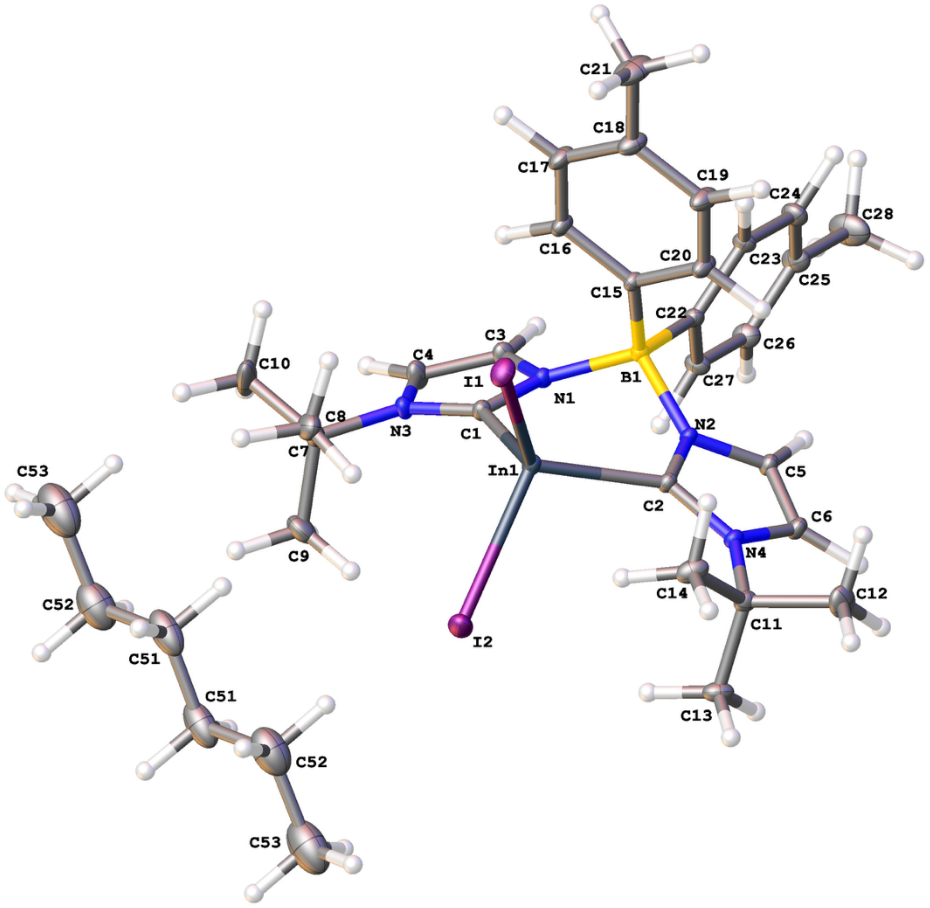

Figure S33. Olex-2 representation of the molecular structure of ligand **2-I**.

7.2.1 Experimental

Single colourless lath-shaped crystals of **2-I** were recrystallised from hexane by slow evaporation. A suitable crystal with dimensions 0.20 × 0.03 × 0.01 mm was selected and mounted on a MITIGEN holder in oil on a Rigaku FRE+ diffractometer with HF Varimax confocal mirrors, a UG2 goniometer and HyPix 6000HE detector. The crystal was kept at a steady T = 100(2) K during data collection. The structure was solved with the ShelXT 2018/2[30] solution program using dual methods and by using Olex2 1.5-dev[31] as the graphical interface. The model was refined with olex2.refine 1.5-dev[32] using full matrix least squares minimisation on $|F|^2$.

7.2.2 Crystal Data

$C_{31}H_{43}BI_2InN_4$, M_r = 851.165, monoclinic, $P2_1/c$ (No. 14), a = 12.9114(2) Å, b = 25.2464(4) Å, c = 10.5301(2) Å, β = 96.525(1)°, $\alpha = \gamma$ = 90°, V = 3410.22(10) Å^3, T = 100(2) K, Z = 4, Z' = 1, m(Mo K_a) = 2.529, 74812 reflections measured, 17291 unique (R_{int} = 0.0403) which were used in all calculations. The final wR_2 was 0.0576 (all data) and R_1 was 0.0291 (I≥2σ(I)).

**Compound**	**2-I**
Formula	$C_{31}H_{43}BI_2InN_4$
$D_{calc.}$/ g cm^{-3}	1.658
m/mm^{-1}	2.529
Formula Weight	851.165
Colour	colourless
Shape	lath-shaped
Size/mm	0.20×0.03×0.01
T/K	100(2)
Crystal System	monoclinic
Space Group	$P2_1/c$
a/Å	12.9114(2)
b/Å	25.2464(4)
c/Å	10.5301(2)
α/°	90
β/°	96.525(1)
γ/°	90
V/Å^3	3410.22(10)
Z	4
Z'	1
Wavelength/Å	0.71073
Radiation type	Mo K_a
Θ_{min}/°	1.78
Θ_{max}/°	38.06
Index range h	-21 ≥ h ≥ 21
Index range k	-42 ≥ k ≥ 42
Index range l	-16 ≥ l ≥ 17
Measured Refl's.	74812
Indep't Refl's	17291
Refl's I≥2σ(I)	14042
R_{int}	0.0403
Parameters	391
Restraints	0
Largest Peak/eÅ^3	1.2961
Deepest Hole/eÅ^3	-1.0597
GooF	1.0158
R_1 (I≥2σ(I) / all)	0.0291 / 0.0440
wR_2 (I≥2σ(I) / all)	0.0538 / 0.0576

7.2.3 Structure Quality Indicators

**Reflections:**	d min (MoKα) 2Θ=76.1° 0.58	I/σ(I) 27.2	Rint m=4.38 4.03%	Full 50.5° 93% to 76.1° 99.7
**Refinement:**	Shift 0.001	Max Peak 1.3	Min Peak -1.1	GooF 1.016

A colourless lath-shaped crystal with dimensions 0.20 × 0.03 × 0.01 mm was mounted on a MITIGEN holder in oil. Data were collected using a Rigaku FRE+ diffractometer with HF Varimax confocal mirrors, a UG2 goniometer and HyPix 6000HE detector equipped with an Oxford Cryosystems low-temperature device operating at T = 100(2) K.

Data were measured using profile data from ω-scans with Mo K_α radiation. The diffraction pattern was indexed and the total number of runs and images was based on the strategy calculation from the program CrysAlis^{Pro} system (CCD 44.126a 64-bit (release 23-10-2025)). The maximum resolution achieved was Θ = 38.06° (0.58 Å).

The unit cell was refined using CrysAlis^{Pro} on 28375 reflections, 38% of the observed reflections.

Data reduction, scaling and absorption corrections were performed using CrysAlis^{Pro}. The final completeness is 99.68 % out to 38.06° in Θ. A multi-scan absorption correction was performed using CrysAlis^{Pro} 1.171.44.126a (Rigaku Oxford Diffraction, 2025). Empirical absorption correction using spherical harmonics was implemented in SCALE3 ABSPACK scaling algorithm. The absorption coefficient μ of this material is 2.529 mm^{-1} at this wavelength (λ = 0.71073Å) and the minimum and maximum transmissions are 0.692 and 1.000.

The structure was solved in the space group $P2_1/c$ (# 14) by ShelXT 2018/2[30] using dual methods. It was refined by full matrix least squares minimisation on $|F|^2$ using version of olex2.refine 1.5-dev.[32] All non-hydrogen atoms were refined anisotropically.

Hydrogen atom positions were calculated geometrically and refined using the riding model.

_refine_special_details: In and I atoms were refined anisotropically with 3rd order anharmonic components.

_olex2_refine_details: Refinement using NoSpherA2, an implementation of NOn-SPHERical Atom-form-factors in Olex2.[33] 2021 NoSpherA2 implementation of HAR makes use of tailor-made aspherical atomic form factors calculated on-the-fly from a Hirshfeld-partitioned electron density (ED) - not from spherical-atom form factors. The ED is calculated from a gaussian basis set single determinant SCF wavefunction - either Hartree-Fock or DFT using selected functionals- for a fragment of the crystal. This fragment can be embedded in an electrostatic crystal field by employing cluster charges or modelled using implicit solvation models, depending on the software used. The following options were used: SOFTWARE: ORCA 6.1 ::: PARTITIONING: NoSpherA2 ::: INT ACCURACY: Normal ::: METHOD: B3LYP ::: BASIS SET: def2-SVP ::: CHARGE: 0 ::: MULTIPLICITY: 2 ::: DATE: 2025-10-28_18-20-38

_exptl_absorpt_process_details: CrysAlis^{Pro} 1.171.44.126a (Rigaku Oxford Diffraction, 2025) using spherical harmonics, implemented in SCALE3 ABSPACK scaling algorithm.

There is a single formula unit in the asymmetric unit, which is represented by the reported sum formula. In other words: Z is 4 and Z' is 1. The moiety formula is C28 H36 B I2 In N4, 0.5(C6 H14).

7.2.4 Data Plots: Diffraction Data

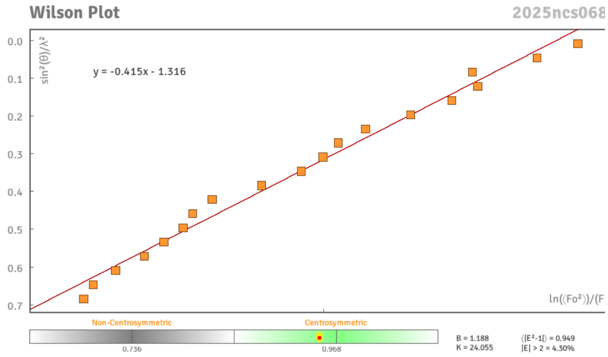

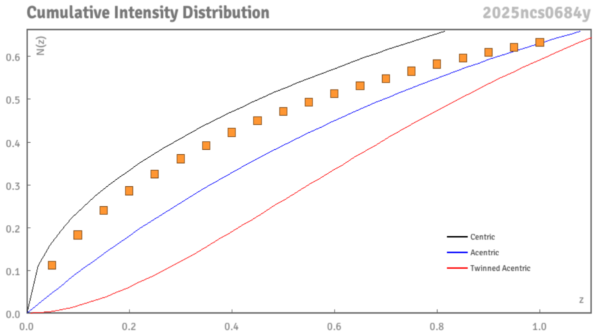

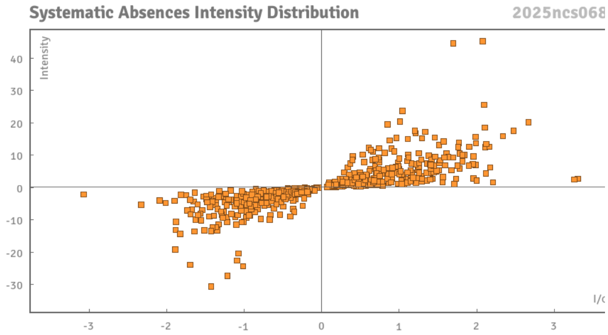

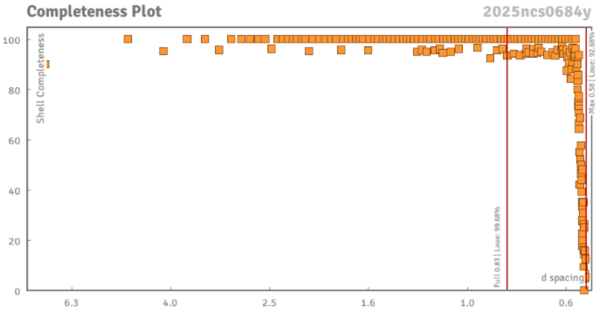

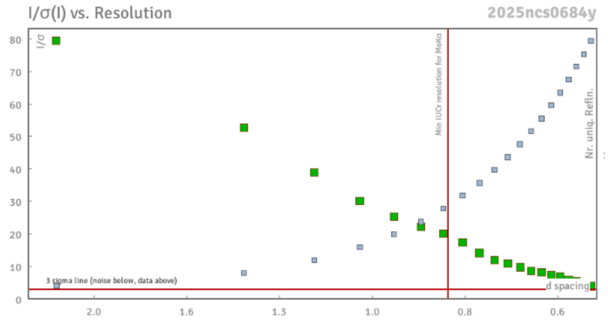

7.2.5 Data Plots: Refinement and Data

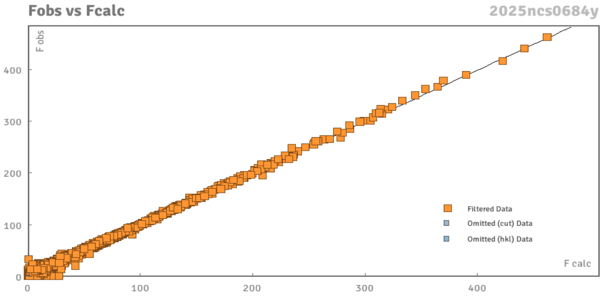

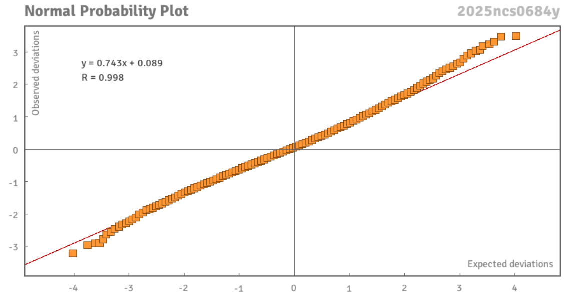

7.2.6 Reflection Statistics

Total reflections (after filtering)	75765	Unique reflections	17291
Completeness	0.927	Mean I/*s*	20.25
hkl_{max} collected	(21, 42, 17)	hkl_{min} collected	(-21, -42, -16)
hkl_{max} used	(21, 42, 17)	hkl_{min} used	(-21, 0, 0)
Lim d_{max} collected	100.0	Lim d_{min} collected	0.36
d_{max} used	11.44	d_{min} used	0.58
Friedel pairs	14226	Friedel pairs merged	1
Inconsistent equivalents	10	R_{int}	0.0403
R_{sigma}	0.0368	Intensity transformed	0
Omitted reflections	0	Omitted by user (OMIT hkl)	0
Multiplicity	(23612, 16498, 4310, 1143, 227, 71, 10, 3)	Maximum multiplicity	22
Removed systematic absences	953	Filtered off (Shel/OMIT)	0

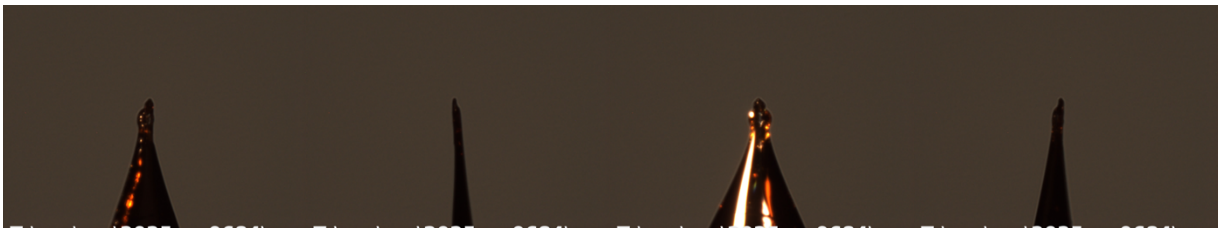

7.2.7 Fractional Atomic Coordinates and Equivalent Isotropic Displacement Parameters

Table S16. Fractional Atomic Coordinates (×10^4) and Equivalent Isotropic Displacement Parameters (Å^2×10^3) for **2-I**. U_{eq} is defined as 1/3 of the trace of the orthogonalised U_{ij}.

Atom	x	y	z	U_{eq}
I1	2484.56(17)	4163.55(8)	9418.17(18)	18.00(2)
I2	284.90(16)	3668.70(8)	6129.8(2)	21.22(2)
In1	2352.24(16)	3744.51(8)	7054.66(18)	11.50(2)
N1	4055.5(9)	3928.0(5)	5324.3(10)	11.38(19)
N2	3941.4(9)	2979.0(4)	6249.1(10)	10.27(18)
N3	2990.0(9)	4589.5(5)	4847.3(11)	14.5(2)
N4	2774.1(9)	2446.1(4)	6901.4(10)	11.30(19)
C1	3176.8(10)	4165.8(5)	5633.8(12)	12.0(2)
C2	3059.9(10)	2963.4(5)	6833.1(12)	10.8(2)
C3	4417.6(11)	4203.3(6)	4334.5(13)	16.2(3)
C4	3750.0(12)	4611.3(6)	4023.0(14)	18.6(3)
C5	4206.6(11)	2469.7(5)	5945.9(12)	12.4(2)
C6	3483.1(11)	2137.3(5)	6344.5(12)	13.5(2)
C7	2120.0(12)	4984.4(6)	4782.7(15)	17.9(3)
C8	1746.7(13)	5048.3(6)	6095.5(15)	22.5(3)
C9	1247.4(13)	4792.6(7)	3792.7(15)	24.7(3)
C10	2527.7(14)	5520.9(7)	4371(2)	35.1(5)
C11	1887.8(11)	2209.4(6)	7514.6(12)	13.4(2)
C12	2267.9(13)	1690.3(6)	8158.8(16)	23.5(3)
C13	966.2(12)	2097.2(6)	6512.9(13)	18.0(3)
C14	1573.1(12)	2580.9(6)	8539.3(13)	19.2(3)
C15	5015.9(10)	3718.4(5)	7563.9(12)	11.2(2)
C16	5169.2(11)	4261.6(5)	7790.9(13)	14.5(2)
C17	5570.5(12)	4459.3(6)	8981.9(13)	17.9(3)
C18	5839.2(13)	4117.8(6)	10005.4(13)	18.8(3)
C19	5689.1(13)	3575.5(6)	9798.0(13)	19.8(3)
C20	5282.5(12)	3383.5(6)	8611.3(12)	16.1(3)
C21	6287.1(17)	4323.9(8)	11294.2(15)	31.6(4)
C22	5674.6(10)	3287.9(5)	5449.4(12)	11.6(2)
C23	6668.4(10)	3231.6(6)	6118.6(12)	13.3(2)
C24	7513.3(11)	3040.4(6)	5536.6(14)	17.4(3)
C25	7408.7(12)	2902.2(6)	4250.1(14)	18.8(3)
C26	6422.8(12)	2952.6(6)	3564.7(13)	17.3(3)
C27	5579.4(11)	3136.4(6)	4151.2(13)	14.9(2)
C28	8321.8(14)	2700.0(9)	3620.6(18)	33.1(4)
B1	4683.7(12)	3481.0(6)	6149.1(13)	10.7(2)
C51	335.0(17)	5222.8(9)	324.1(17)	38.2(5)
C52	525.2(19)	5674.4(10)	-569(2)	45.7(6)
C53	1211(2)	6147.7(12)	106(2)	56.5(7)

7.2.8 Anisotropic Displacement and Structural Parameters

Table S17. Anisotropic Displacement Parameters (×10^4) for **2-I**. The anisotropic displacement factor exponent takes the form: $-2p^2[h^2a^{*2} \times U_{11}+ ... +2hka^* \times b^* \times U_{12}]$

Atom	U_{11}	U_{22}	U_{33}	U_{23}	U_{13}	U_{12}
I1	23.72(5)	14.35(4)	16.44(4)	1.33(3)	4.55(3)	-2.41(3)
I2	11.57(4)	16.54(5)	34.99(5)	-0.42(3)	0.19(3)	2.93(4)
In1	11.02(4)	9.04(4)	14.79(4)	0.28(3)	3.03(3)	-0.28(3)
N1	10.1(5)	9.7(5)	14.4(4)	-0.5(4)	1.2(4)	2.1(4)
N2	9.6(5)	7.4(4)	13.8(4)	-0.8(4)	1.3(3)	0.2(4)
N3	10.8(5)	11.0(5)	21.3(5)	1.3(4)	0.1(4)	4.8(4)
N4	11.1(5)	8.9(5)	13.8(4)	-1.9(4)	0.7(4)	0.6(4)
C1	10.5(5)	9.6(5)	15.9(5)	0.0(4)	1.5(4)	2.0(4)
C2	11.0(5)	8.2(5)	13.3(5)	-0.6(4)	1.9(4)	0.1(4)
C3	13.8(6)	15.4(6)	20.0(6)	0.2(5)	4.4(5)	6.8(5)
C4	15.9(6)	16.0(7)	24.3(6)	1.8(5)	3.8(5)	9.9(5)
C5	12.3(6)	8.6(5)	16.6(5)	0.1(4)	2.3(4)	-0.2(4)
C6	13.7(6)	8.8(5)	18.0(5)	-0.9(5)	2.6(4)	-0.2(4)
C7	12.7(6)	12.0(6)	28.5(7)	1.1(5)	0.0(5)	5.4(5)
C8	19.7(7)	15.2(7)	31.9(8)	3.4(6)	-0.1(6)	-4.0(6)
C9	16.6(7)	30.0(9)	25.7(7)	3.3(6)	-4.8(5)	4.4(6)
C10	21.0(8)	14.5(7)	70.5(14)	2.4(6)	7.7(8)	16.6(8)
C11	14.4(6)	12.2(6)	13.6(5)	-2.5(5)	1.6(4)	1.6(4)
C12	24.1(8)	19.1(7)	27.9(7)	-0.8(6)	5.8(6)	11.9(6)
C13	15.4(6)	22.0(7)	16.1(6)	-6.5(5)	0.4(5)	-1.8(5)
C14	21.3(7)	20.8(7)	16.4(6)	-7.3(6)	6.4(5)	-3.6(5)
C15	12.0(5)	8.6(5)	12.7(5)	-1.5(4)	0.7(4)	0.4(4)
C16	17.9(6)	9.2(5)	16.1(5)	-2.8(5)	0.3(5)	0.3(5)
C17	23.5(7)	11.0(6)	18.6(6)	-4.5(5)	-0.1(5)	-2.3(5)
C18	24.4(7)	16.4(7)	15.0(6)	-6.8(6)	-0.1(5)	-1.9(5)
C19	27.5(8)	15.4(6)	15.0(6)	-6.4(6)	-4.5(5)	1.9(5)
C20	22.6(7)	10.8(6)	13.8(5)	-4.0(5)	-2.6(5)	1.4(5)
C21	49.0(12)	26.3(9)	17.6(6)	-13.6(8)	-3.9(7)	-4.3(6)
C22	9.8(5)	10.4(5)	14.5(5)	-1.0(4)	1.6(4)	0.3(4)
C23	9.5(5)	14.9(6)	15.4(5)	-0.5(5)	0.8(4)	-0.1(5)
C24	9.7(6)	19.9(7)	22.8(6)	1.1(5)	2.3(5)	-1.6(5)
C25	13.3(6)	20.6(7)	23.2(7)	0.1(5)	5.4(5)	-4.8(5)
C26	15.6(6)	19.7(7)	17.3(6)	-0.4(5)	4.9(5)	-3.6(5)
C27	12.0(6)	18.1(6)	14.6(5)	0.1(5)	1.2(4)	-1.3(5)
C28	18.4(8)	45.9(12)	36.3(9)	3.1(8)	9.6(7)	-14.8(8)
B1	9.7(6)	9.8(6)	12.6(5)	-0.5(5)	0.6(4)	0.9(5)
C51	37.9(11)	51.8(13)	24.7(8)	23.8(10)	3.3(7)	-1.3(8)
C52	42.4(13)	59.8(15)	35.4(10)	18.0(11)	7.3(9)	4.4(10)
C53	53.3(16)	72.6(19)	43.7(12)	29.4(14)	5.4(11)	-2.6(12)

Table S18. Bond Lengths in Å for **2-I**.

Atom	Atom	Length/Å
I1	In1	2.6918(3)
I2	In1	2.7421(3)
In1	C1	2.2057(14)
In1	C2	2.1970(13)
N1	C1	1.3556(18)
N1	C3	1.3780(18)
N1	B1	1.5892(18)
N2	C2	1.3543(17)
N2	C5	1.3774(17)
N2	B1	1.5997(18)
N3	C1	1.3576(17)
N3	C4	1.383(2)
N3	C7	1.4977(18)
N4	C2	1.3612(17)
N4	C6	1.3826(18)
N4	C11	1.5005(18)
C3	C4	1.359(2)
C5	C6	1.3571(19)
C7	C8	1.523(2)
C7	C9	1.524(2)
C7	C10	1.534(2)
C11	C12	1.530(2)
C11	C13	1.5243(18)

Atom	Atom	Length/Å
C11	C14	1.519(2)
C15	C16	1.4022(18)
C15	C20	1.4014(18)
C15	B1	1.6179(19)
C16	C17	1.3938(18)
C17	C18	1.393(2)
C18	C19	1.397(2)
C18	C21	1.506(2)
C19	C20	1.3868(18)
C22	C23	1.3992(18)
C22	C27	1.4114(18)
C22	B1	1.622(2)
C23	C24	1.397(2)
C24	C25	1.391(2)
C25	C26	1.396(2)
C25	C28	1.506(2)
C26	C27	1.391(2)
C51	C51[1]	1.532(4)
C51	C52	1.516(3)
C52	C53	1.604(4)

[1]-x,1-y,-z

Table S19. Bond Angles in ° for **2-I**.

Atom	Atom	Atom	Angle/°
I2	In1	I1	108.225(9)
C1	In1	I1	116.92(3)
C1	In1	I2	108.55(3)
C2	In1	I1	117.87(3)
C2	In1	I2	107.42(3)
C2	In1	C1	97.02(5)
C3	N1	C1	108.88(11)
B1	N1	C1	124.90(11)
B1	N1	C3	125.15(11)
C5	N2	C2	108.78(11)
B1	N2	C2	126.42(11)
B1	N2	C5	123.90(11)
C4	N3	C1	108.95(12)
C7	N3	C1	129.23(13)
C7	N3	C4	121.81(12)
C6	N4	C2	108.78(11)
C11	N4	C2	129.01(12)
C11	N4	C6	122.14(11)
N1	C1	In1	115.54(9)
N3	C1	In1	136.70(10)
N3	C1	N1	107.32(12)
N2	C2	In1	113.78(9)
N4	C2	In1	137.48(10)

Atom	Atom	Atom	Angle/°
N4	C2	N2	107.38(12)
C4	C3	N1	107.73(13)
C3	C4	N3	107.11(12)
C6	C5	N2	107.90(12)
C5	C6	N4	107.15(12)
C8	C7	N3	110.02(12)
C9	C7	N3	108.17(12)
C9	C7	C8	111.64(13)
C10	C7	N3	108.72(13)
C10	C7	C8	108.49(14)
C10	C7	C9	109.75(14)
C12	C11	N4	108.06(12)
C13	C11	N4	110.47(10)
C13	C11	C12	109.48(12)
C14	C11	N4	109.77(11)
C14	C11	C12	108.17(12)
C14	C11	C13	110.81(12)
C20	C15	C16	116.02(12)
B1	C15	C16	122.47(11)
B1	C15	C20	121.12(12)
C17	C16	C15	122.30(12)
C18	C17	C16	120.61(13)
C19	C18	C17	117.89(13)

Atom	Atom	Atom	Angle/°
C21	C18	C17	121.32(14)
C21	C18	C19	120.79(14)
C20	C19	C18	121.02(13)
C19	C20	C15	122.15(13)
C27	C22	C23	115.74(13)
B1	C22	C23	121.82(11)
B1	C22	C27	122.34(11)
C24	C23	C22	122.10(12)
C25	C24	C23	121.33(13)
C26	C25	C24	117.56(14)
C28	C25	C24	121.31(14)

Atom	Atom	Atom	Angle/°
C28	C25	C26	121.12(14)
C27	C26	C25	120.98(13)
C26	C27	C22	122.27(13)
N2	B1	N1	109.04(10)
C15	B1	N1	107.79(11)
C15	B1	N2	109.33(10)
C22	B1	N1	110.19(11)
C22	B1	N2	107.53(11)
C22	B1	C15	112.91(11)
C53	C52	C51	113.92(18)

Table S20. Torsion Angles in ° for **2-I**.

Atom	Atom	Atom	Atom	Angle/°
In1	C1	N1	C3	173.46(9)
In1	C1	N1	B1	-17.91(12)
In1	C1	N3	C4	-170.70(14)
In1	C1	N3	C7	8.05(17)
In1	C2	N2	C5	-168.86(8)
In1	C2	N2	B1	21.73(11)
In1	C2	N4	C6	164.72(13)
In1	C2	N4	C11	-18.22(16)
N1	C1	N3	C4	1.03(12)
N1	C1	N3	C7	179.78(10)
N1	C3	C4	N3	1.22(13)
N1	B1	N2	C2	-64.46(13)
N1	B1	N2	C5	127.63(10)
N1	B1	C15	C16	-27.59(14)
N1	B1	C15	C20	159.84(11)
N1	B1	C22	C23	133.33(11)
N1	B1	C22	C27	-50.51(13)
N2	C2	N4	C6	-0.43(12)
N2	C2	N4	C11	176.63(9)
N2	C5	C6	N4	-0.31(11)
N2	B1	N1	C1	61.02(13)
N2	B1	N1	C3	-132.17(11)
N2	B1	C15	C16	-145.99(11)
N2	B1	C15	C20	41.43(14)
N2	B1	C22	C23	-107.94(11)
N2	B1	C22	C27	68.22(12)
N3	C1	N1	C3	-0.27(12)
N3	C1	N1	B1	168.36(10)
N4	C2	N2	C5	0.24(11)
N4	C2	N2	B1	-169.18(9)
C1	N1	C3	C4	-0.61(12)
C1	N1	B1	C15	-57.58(13)
C1	N1	B1	C22	178.81(13)
C1	N3	C4	C3	-1.41(12)
C1	N3	C7	C8	29.39(15)
C1	N3	C7	C9	-92.80(15)
C1	N3	C7	C10	148.07(17)
C2	N2	C5	C6	0.05(11)
C2	N2	B1	C15	53.16(13)

Atom	Atom	Atom	Atom	Angle/°
C2	N2	B1	C22	176.08(13)
C2	N4	C6	C5	0.46(11)
C2	N4	C11	C12	-141.05(14)
C2	N4	C11	C13	99.21(14)
C2	N4	C11	C14	-23.28(14)
C3	N1	B1	C15	109.23(14)
C3	N1	B1	C22	-14.38(13)
C3	C4	N3	C7	179.73(11)
C4	N3	C7	C8	-152.00(14)
C4	N3	C7	C9	85.81(15)
C4	N3	C7	C10	-33.32(16)
C4	C3	N1	B1	-169.20(11)
C5	N2	B1	C15	-114.74(12)
C5	N2	B1	C22	8.17(12)
C5	C6	N4	C11	-176.84(9)
C6	N4	C11	C12	35.66(13)
C6	N4	C11	C13	-84.08(13)
C6	N4	C11	C14	153.42(13)
C6	C5	N2	B1	169.79(9)
C15	C16	C17	C18	0.10(18)
C15	C20	C19	C18	0.83(19)
C15	B1	C22	C23	12.75(14)
C15	B1	C22	C27	-171.09(11)
C16	C15	C20	C19	-0.97(16)
C16	C15	B1	C22	94.36(14)
C16	C17	C18	C19	-0.27(19)
C16	C17	C18	C21	179.18(16)
C17	C16	C15	C20	0.51(18)
C17	C16	C15	B1	-172.42(14)
C17	C18	C19	C20	-0.18(19)
C19	C20	C15	B1	172.07(14)
C20	C15	B1	C22	-78.22(14)
C20	C19	C18	C21	-179.63(17)
C22	C23	C24	C25	0.82(17)
C22	C27	C26	C25	0.98(17)
C23	C22	C27	C26	-1.23(16)
C23	C24	C25	C26	-1.09(18)
C23	C24	C25	C28	179.53(16)
C24	C23	C22	C27	0.35(16)
C24	C23	C22	B1	176.75(13)
C24	C25	C26	C27	0.21(18)
C26	C27	C22	B1	-177.61(13)
C27	C26	C25	C28	179.60(16)
C51[1]	C51	C52	C53	179.1(2)
C52	C51	C51[1]	C52[1]	180.0(3)

[1]-x,1-y,

Table S21. Hydrogen Fractional Atomic Coordinates (×10^4) and Equivalent Isotropic Displacement Parameters (Å^2×10^3) for **2-I**. U_{eq} is defined as 1/3 of the trace of the orthogonalised U_{ij}.

Atom	x	y	z	U_{eq}
H3	5122.7(11)	4109.5(6)	3877.1(13)	19.5(3)
H4	3804.1(12)	4904.6(6)	3256.9(14)	22.3(3)
H5	4886.6(11)	2352.7(5)	5464.3(12)	14.9(3)
H6	3464.3(11)	1702.5(5)	6243.3(12)	16.2(3)
H8a	1360(9)	4682.9(19)	6351(5)	33.8(5)
H8b	2416.6(18)	5128(5)	6807(2)	33.8(5)
H8c	1195(8)	5379(3)	6074(4)	33.8(5)
H9a	1551(3)	4742(5)	2869(3)	37.0(5)
H9b	948(7)	4413(3)	4101(6)	37.0(5)
H9c	616(4)	5085(2)	3696(9)	37.0(5)
H10a	3231(7)	5628(3)	5003(9)	52.7(7)
H10b	2715(11)	5494.0(17)	3383(5)	52.7(7)
H10c	1928(4)	5824.7(13)	4433(13)	52.7(7)
H12a	2451(9)	1406.0(18)	7428.7(19)	35.2(5)
H12b	2970(6)	1766.2(11)	8823(9)	35.2(5)
H12c	1656(4)	1527(3)	8686(10)	35.2(5)
H13a	671(6)	2471.2(7)	6082(8)	26.9(4)
H13b	1218(2)	1839(4)	5769(6)	26.9(4)
H13c	346(4)	1901(4)	6967(2)	26.9(4)
H14a	2266.5(18)	2694(4)	9182(7)	28.8(4)
H14b	1210(9)	2937(2)	8087.5(15)	28.8(4)
H14c	1015(7)	2380.2(18)	9088(8)	28.8(4)
H16	4965.1(11)	4542.3(5)	6999.3(13)	17.4(3)
H17	5676.1(12)	4890.0(6)	9115.1(13)	21.4(3)
H19	5897.1(13)	3295.6(6)	10590.3(13)	23.8(4)
H20	5165.1(12)	2953.0(6)	8487.8(12)	19.3(3)
H21a	6804(9)	4660(4)	11166.5(15)	47.4(6)
H21b	5651.8(18)	4454(5)	11830(6)	47.4(6)
H21c	6732(10)	4008.4(19)	11825(6)	47.4(6)
H23	6788.4(10)	3341.7(6)	7138.4(12)	16.0(3)
H24	8279.0(11)	2998.3(6)	6108.9(14)	20.9(3)
H26	6310.8(12)	2845.1(6)	2543.0(13)	20.8(3)
H27	4809.7(11)	3164.7(6)	3581.9(13)	17.9(3)
H28a	9047.0(16)	2861(4)	4118(8)	49.6(6)
H28b	8341(6)	2266.0(9)	3668(11)	49.6(6)
H28c	8242(6)	2826(5)	2617(4)	49.6(6)
H51a	-55.5(17)	5381.5(9)	1131.3(17)	45.8(6)
H51b	1098.4(17)	5055.9(9)	727.5(17)	45.8(6)
H52a	926.5(19)	5516.1(10)	-1368(2)	54.8(7)
H52b	-239.1(19)	5836.0(10)	-985(2)	54.8(7)
H53a	1389(13)	6435(4)	-620(3)	84.8(11)
H53b	773(6)	6344(5)	806(13)	84.8(11)
H53c	1940(7)	5987.3(16)	593(15)	84.8(11)

7.3 Single crystal structure analysis of 3-Br

CCDC Deposition number 2503902

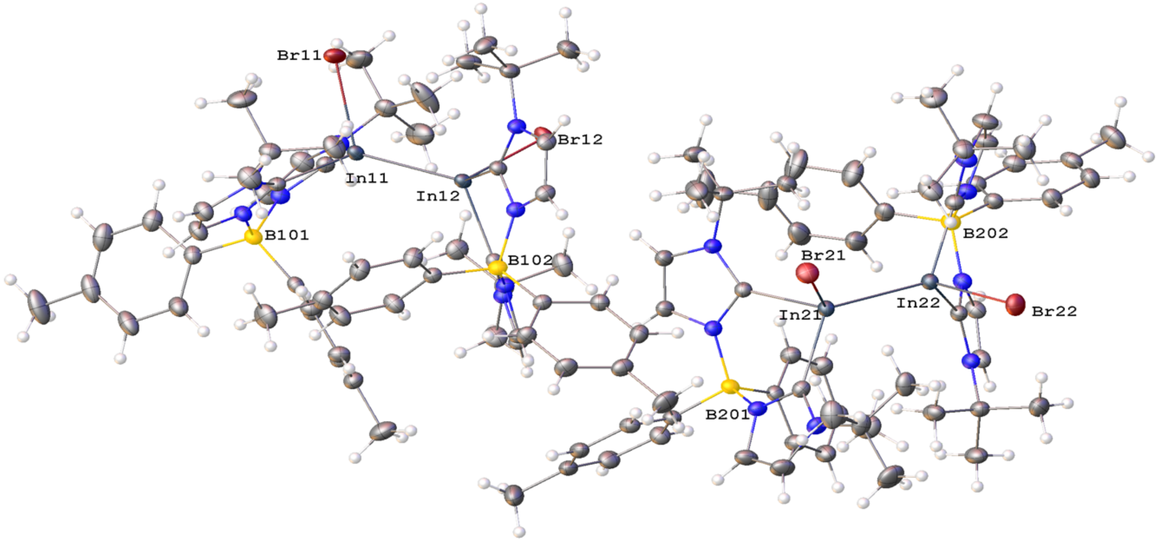

Figure S34. Olex-2 representation of the molecular structure of ligand **3-Br**.

7.3.1 Experimental

Single colourless chip-shaped crystals of **3-Br** were recrystallised by solvent layering of ether and pentane. A suitable crystal with dimensions 0.16 × 0.14 × 0.09 mm was selected and mounted on a MITIGEN holder in oil on a Rigaku FRE+ diffractometer with Arc)Sec VHF Varimax confocal mirrors, a UG2 goniometer and HyPix 6000HE detector. The crystal was kept at a steady T = 100(2) K during data collection. The structure was solved with the ShelXT 2018/2[30] solution program using dual methods and by using Olex2 1.5-dev[31] as the graphical interface. The model was refined with olex2.refine 1.5-dev[32] using full matrix least squares minimisation on $|F|^2$.

7.3.2 Crystal Data

$C_{60}H_{82}B_2Br_2In_2N_8O$, M_r = 1342.465, triclinic, P-1 (No. 2), a = 14.6908(1) Å, b = 18.3799(1) Å, c = 24.1277(2) Å, α = 75.112(1)°, β = 86.573(1)°, γ = 82.740(1)°, V = 6243.14(8) Å^3, T = 100(2) K, Z = 4, Z' = 2, μ(Mo K_α) = 2.067, 605311 reflections measured, 65838 unique (R_{int} = 0.0664) which were used in all calculations. The final wR_2 was 0.0837 (all data) and R_1 was 0.0370 (I≥2σ(I)).

**Compound**	**3-Br**
Formula	$C_{60}H_{82}B_2Br_2In_2N_8O$
$D_{calc.}$/ g cm^{-3}	1.428
μ/mm^{-1}	2.067
Formula Weight	1342.465
Colour	colourless
Shape	chip-shaped
Size/mm	0.16×0.14×0.09
T/K	100(2)
Crystal System	triclinic
Space Group	P-1
a/Å	14.6908(1)
b/Å	18.3799(1)
c/Å	24.1277(2)
α/°	75.112(1)
β/°	86.573(1)
γ/°	82.740(1)
V/Å^3	6243.14(8)
Z	4
Z'	2
Wavelength/Å	0.71073
Radiation type	Mo K_α
Θ_{min}/°	1.67
Θ_{max}/°	38.21
Index range h	-25 ≥ h ≥ 25
Index range k	-31 ≥ k ≥ 31
Index range l	-41 ≥ l ≥ 41
Measured Refl's.	605311
Indep't Refl's	65838
Refl's I≥2σ(I)	47088
R_{int}	0.0664
Parameters	1293
Restraints	0
Largest Peak/eÅ^3	2.1091
Deepest Hole/eÅ^3	-1.6380
GooF	0.9945
R_1 (I≥2σ(I) / all)	0.0370 / 0.0666
wR_2 (I≥2σ(I) / all)	0.0745 / 0.0837

7.3.3 Structure Quality Indicators

Reflections:	d min (MoKα) 2Θ=76.4° 0.57	I/σ(I) 23.7	Rint m=9.19 6.64%	Full 50.5° 95% to 76.4° 100
Refinement:	Shift 0.001	Max Peak 2.1	Min Peak -1.6	GooF 0.995

A colourless chip-shaped crystal with dimensions 0.16 × 0.14 × 0.09 mm was mounted on a MITIGEN holder in oil. Data were collected using a Rigaku FRE+ diffractometer with Arc)Sec VHF Varimax confocal mirrors, a UG2 goniometer and HyPix 6000HE detector equipped with an Oxford Cryosystems low-temperature device operating at T = 100(2) K.

Data were measured using profile data from ω-scans with Mo K_α radiation. The diffraction pattern was indexed and the total number of runs and images was based on the strategy calculation from the program CrysAlis^{Pro} system (CCD 43.125a 64-bit (release 04-06-2024)). The maximum resolution achieved was Θ = 38.21° (0.57 Å).

The unit cell was refined using CrysAlis^{Pro} on 161059 reflections, 27% of the observed reflections.

Data reduction, scaling and absorption corrections were performed using CrysAlis^{Pro}. The final completeness is 99.97 % out to 38.21° in Θ. A gaussian absorption correction was performed using CrysAlis^{Pro} 1.171.43.125a (Rigaku Oxford Diffraction, 2024). Numerical absorption correction based on gaussian integration over a multifaceted crystal model Empirical absorption correction using spherical harmonics, implemented in SCALE3 ABSPACK scaling algorithm. The absorption coefficient μ of this material is 2.067 mm^{-1} at this wavelength (λ = 0.71073Å) and the minimum and maximum transmissions are 0.530 and 1.000.

The structure was solved in the space group *P*-1 (# 2) by ShelXT 2018/2 using dual methods.[30] It was refined by full matrix least squares minimisation on $|F|^2$ using version of olex2.refine 1.5-dev.[32] All non-hydrogen atoms were refined anisotropically.

Hydrogen atom positions were calculated geometrically and refined using the riding model.

_refine_special_details: Solvent masking was used to omit the electronic contribution equivalent to two molecules of ether per ASU

_exptl_absorpt_process_details: CrysAlis^{Pro} 1.171.43.125a (Rigaku Oxford Diffraction, 2024). Numerical absorption correction based on gaussian integration over a multifaceted crystal model. Empirical absorption correction using spherical harmonics, implemented in SCALE3 ABSPACK scaling algorithm.

_smtbx_masks_special_details: A solvent mask was calculated and 170 electrons were found in a volume of 1039Å^3 in 4 voids per unit cell. This is consistent with the presence of 1[C4H10O] per formula unit which account for 168 electrons per unit cell.

The value of Z' is 2. This means that there are two independent molecules in the asymmetric unit. The moiety formula is C56 H72 B2 Br2 In2 N8, 1[C4H10O].

_olex2_refine_details: Refinement using NoSpherA2, an implementation of NOn-SPHERical Atom-form-factors in Olex2.[33] 2021 NoSpherA2 implementation of HAR makes use of tailor-made aspherical atomic form factors calculated on-the-fly from a Hirshfeld-partitioned electron density (ED) - not from spherical-atom form factors. The ED is calculated from a gaussian basis set single determinant SCF wavefunction - either Hartree-Fock or DFT using selected functionals- for a fragment of the crystal. This fragment can be embedded in an electrostatic crystal field by employing cluster charges or modelled using implicit solvation models, depending on the software used. The following options were used: SOFTWARE: ORCA 6.1 ::: PARTITIONING: NoSpherA2 ::: INT ACCURACY: Normal ::: METHOD: r2SCAN ::: BASIS SET: jorge-DZP-DKH ::: CHARGE: 0 ::: MULTIPLICITY: 1 ::: RELATIVISTIC: ZORA ::: DATE: 2025-11-17_13-56-07

7.3.4 Data Plots: Diffraction Data

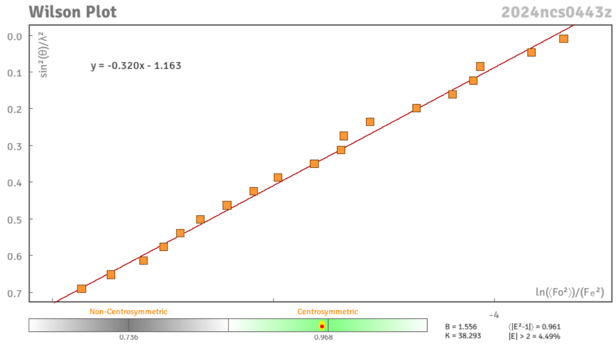

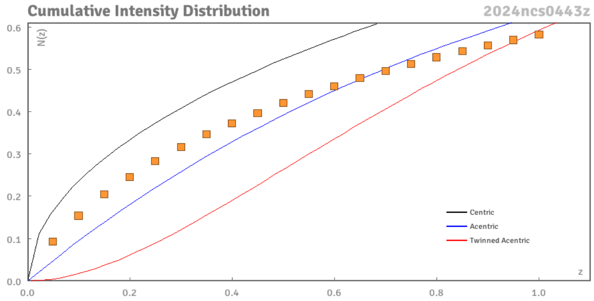

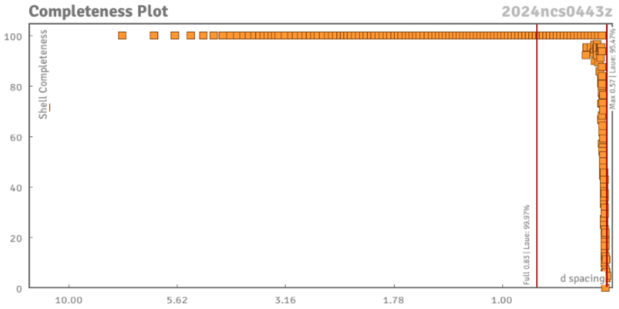

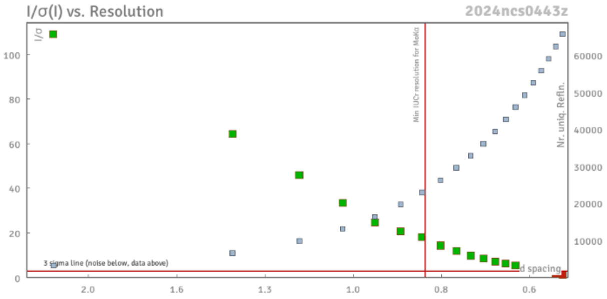

7.3.5 Data Plots: Refinement and Data

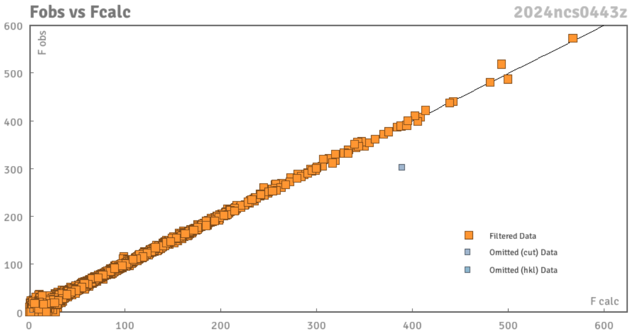

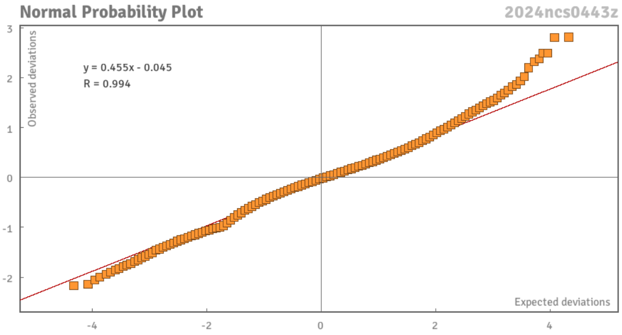

7.3.6 Reflection Statistics

Total reflections (after filtering)	605310	Unique reflections	65838
Completeness	0.955	Mean I/σ	20.75
hkl_{max} collected	(25, 31, 41)	hkl_{min} collected	(-25, -31, -41)
hkl_{max} used	(25, 31, 41)	hkl_{min} used	(-25, -30, 0)
Lim d_{max} collected	100.0	Lim d_{min} collected	0.36
d_{max} used	12.2	d_{min} used	0.57
Friedel pairs	62522	Friedel pairs merged	1
Inconsistent equivalents	6	R_{int}	0.0664
R_{sigma}	0.0422	Intensity transformed	0
Omitted reflections	0	Omitted by user (OMIT hkl)	1
Multiplicity	(8360, 19155, 25483, 22261, 14194, 9811, 7959, 6872, 4977, 3554, 2429, 1287, 678, 516, 289, 150, 132, 80, 53, 36, 36, 13, 8, 9, 7, 5, 4, 1, 0, 2)	Maximum multiplicity	41
Removed systematic absences	0	Filtered off (Shel/OMIT)	0

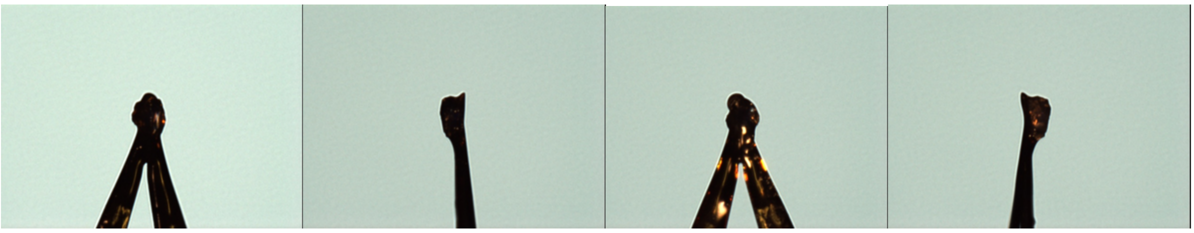

7.3.7 Fractional Atomic Coordinates

Table S22. Fractional Atomic Coordinates (×10^4) and Equivalent Isotropic Displacement Parameters (Å^2×10^3) for **3-Br**. U_{eq} is defined as 1/3 of the trace of the orthogonalised U_{ij}.

Atom	x	y	z	U_{eq}
In11	8336.22(7)	2469.72(6)	1983.42(4)	17.54(2)
In12	7539.34(7)	3645.07(6)	2433.83(4)	16.81(2)
Br11	7434.76(12)	1288.40(11)	2241.76(9)	30.10(4)
Br12	5895.86(11)	4194.63(12)	2070.74(8)	30.50(4)
N101	8008.3(9)	2705.5(8)	589.8(6)	23.0(3)
N102	9442.9(9)	2499.5(8)	835.3(5)	20.9(2)
N103	10155.8(9)	1463.0(8)	2685.3(5)	19.6(2)
N104	10398.2(9)	1965.2(7)	1772.9(5)	19.2(2)
N105	6589.9(9)	3234.6(8)	3746.1(6)	20.0(2)
N106	7619.2(9)	4027.1(7)	3609.0(5)	18.8(2)
N107	8330.9(9)	5299.7(7)	1824.1(5)	19.4(2)
N108	8455.2(9)	4908.6(7)	2753.3(5)	18.3(2)
C101	8568.6(10)	2599.5(9)	1040.3(6)	20.5(3)
C102	8533.3(12)	2670.9(11)	101.0(7)	28.9(3)
C103	9424.8(12)	2547.4(11)	254.7(7)	28.4(3)
C104	6988.3(11)	2861.6(10)	555.0(7)	25.7(3)
C105	6634.9(15)	2209.9(13)	390.5(11)	43.2(5)
C106	6544.3(14)	2964.8(18)	1114.1(9)	52.3(7)
C107	6765.5(15)	3601.8(13)	95.4(11)	47.4(6)
C108	9743.4(10)	1892.6(8)	2195.4(6)	18.4(2)
C109	11076.5(11)	1275.1(10)	2563.7(7)	25.5(3)
C110	11220.9(11)	1593.1(10)	1998.3(7)	25.4(3)
C111	9724.6(11)	1153.3(10)	3260.8(7)	23.8(3)
C112	8932.6(12)	1717.3(12)	3382.7(8)	31.8(4)
C113	9397.6(16)	396.1(12)	3265.0(9)	39.1(5)
C114	10443.3(13)	1025.9(12)	3720.8(7)	31.4(4)
C115	10487.5(10)	3353.5(9)	1167.3(6)	19.7(3)
C116	10742.5(11)	3542.2(9)	1656.6(7)	22.7(3)
C117	10985.9(11)	4258.0(10)	1635.2(7)	24.7(3)
C118	10994.1(12)	4814.8(9)	1120.6(7)	24.7(3)
C119	10715.8(12)	4643.2(10)	630.7(7)	26.1(3)
C120	10460.6(12)	3932.3(9)	656.4(7)	24.0(3)
C121	11274.7(16)	5580.5(11)	1097.8(10)	38.3(4)
C122	11227.0(11)	2187.4(9)	760.9(7)	21.9(3)
C123	12008.2(11)	2568.2(10)	607.9(8)	27.8(3)
C124	12713.4(13)	2339.9(12)	254.7(8)	34.3(4)
C125	12661.7(13)	1722.6(13)	28.1(7)	35.1(4)
C126	11908.4(15)	1321.0(12)	191.5(8)	35.3(4)
C127	11207.6(13)	1540.6(10)	557.1(7)	28.4(3)
C128	13390.9(16)	1515.0(17)	-389.5(9)	51.6(7)
C129	7229.1(10)	3560.9(9)	3361.9(6)	19.0(3)
C130	6573.0(11)	3503.2(9)	4232.3(7)	22.2(3)
C131	7216.2(11)	3990.3(9)	4147.5(6)	21.9(3)
C132	5956.5(11)	2672.7(10)	3709.3(7)	24.3(3)
C133	6085.3(15)	2008.3(10)	4245.8(8)	33.8(4)
C134	4977.6(12)	3071.0(12)	3690.3(10)	36.8(4)
C135	6181.3(13)	2364.2(12)	3183.4(8)	32.1(4)
C136	8137.6(10)	4735.3(8)	2293.4(6)	17.6(2)
C137	8780.1(11)	5817.3(9)	1994.2(7)	22.1(3)
C138	8864.3(11)	5569.3(9)	2569.7(7)	21.7(3)
C139	8016.1(11)	5437.4(10)	1223.0(6)	22.3(3)
C140	7037.9(12)	5838.0(12)	1189.0(8)	31.1(4)
C141	8641.0(13)	5950.5(11)	810.8(7)	30.4(4)
C142	8069.7(13)	4686.3(11)	1057.1(7)	28.0(3)
C143	9413.6(10)	3832.7(9)	3491.7(6)	19.0(3)

Atom	x	y	z	U_{eq}
C144	9580.1(12)	3294.7(10)	4015.8(7)	25.2(3)
C145	10405.7(13)	2830.9(10)	4123.9(7)	27.2(3)
C146	11115.2(12)	2877.1(10)	3708.6(7)	26.0(3)
C147	10942.6(12)	3380.6(11)	3176.1(8)	29.7(4)
C148	10111.9(11)	3845.4(10)	3071.6(7)	25.5(3)
C149	12035.7(13)	2414.2(13)	3828.4(9)	37.7(4)
C150	8523.3(10)	5080.6(9)	3771.4(6)	19.3(3)
C151	9237.3(11)	5101.0(10)	4122.4(7)	24.0(3)
C152	9248.1(13)	5697.4(10)	4386.4(8)	28.2(3)
C153	8544.8(12)	6291.7(10)	4309.4(7)	26.1(3)
C154	7816.5(12)	6274.4(10)	3966.1(7)	24.8(3)
C155	7812.9(11)	5681.7(9)	3707.7(7)	22.2(3)
C156	8550.7(17)	6939.8(12)	4584.6(9)	39.7(5)
B101	10379.1(11)	2498.2(10)	1142.0(7)	19.0(3)
B102	8508.7(12)	4445.7(10)	3408.2(7)	18.4(3)
In21	5107.34(7)	8083.91(6)	3489.94(4)	17.77(2)
In22	3442.88(7)	8972.57(6)	3222.46(5)	18.52(2)
Br21	5193.81(13)	7208.63(11)	4536.60(7)	28.45(4)
Br22	2926.28(13)	9785.35(11)	3959.39(8)	30.59(4)
N201	5516.5(9)	6564.7(8)	2947.7(6)	20.8(2)
N202	6470.9(9)	7415.9(8)	2669.1(5)	19.5(2)
N203	7082.8(9)	8445.9(8)	3084.5(6)	21.2(2)
N204	6801.4(10)	8943.9(8)	3816.6(6)	24.7(3)
N205	3632.8(9)	10486.7(8)	2128.3(6)	21.3(2)
N206	2685.2(9)	9720.4(8)	2004.3(6)	21.2(2)
N207	1692.4(9)	8717.3(8)	2658.6(6)	23.7(3)
N208	1551.5(10)	8105.7(9)	3557.6(6)	26.6(3)
C201	5723.9(10)	7245.9(9)	3010.9(6)	19.2(3)
C202	6135.5(11)	6321.0(9)	2560.5(7)	23.1(3)
C203	6719.9(11)	6853.4(9)	2385.9(7)	22.4(3)
C204	4826.2(11)	6081.1(10)	3282.0(8)	25.4(3)
C205	3973.3(11)	6580.5(10)	3412.7(8)	27.0(3)
C206	4556.3(14)	5566.7(12)	2922.1(10)	38.9(5)
C207	5266.5(14)	5591.5(11)	3832.9(9)	36.5(4)
C208	6426.3(11)	8571.3(9)	3480.4(6)	20.6(3)
C209	7692.3(13)	9047.0(11)	3626.5(8)	32.2(4)
C210	7862.6(12)	8745.4(11)	3169.4(8)	29.1(3)
C211	6411.0(13)	9202.2(10)	4330.1(7)	27.7(3)
C212	5369.4(13)	9270.0(12)	4351.6(8)	32.5(4)
C213	6793.2(15)	8628.4(13)	4865.2(8)	39.9(5)
C214	6705.2(18)	9982.7(13)	4293.8(11)	45.2(5)
C215	6372.4(10)	8839.1(9)	2068.8(7)	20.6(3)
C216	6707.8(12)	9550.5(10)	1889.4(8)	26.5(3)
C217	6341.5(12)	10112.4(10)	1431.3(8)	27.5(3)
C218	5611.3(12)	9995.5(10)	1129.6(7)	26.9(3)
C219	5246.4(13)	9313.0(10)	1320.5(8)	29.3(3)
C220	5618.9(11)	8744.8(10)	1779.1(7)	24.9(3)
C221	5246.2(15)	10579.9(12)	606.1(9)	38.5(4)
C222	7972.6(10)	7963.7(9)	2244.1(7)	20.8(3)
C223	8675.0(11)	7494.1(9)	2575.9(7)	23.5(3)
C224	9528.6(11)	7298.5(10)	2334.2(8)	26.6(3)
C225	9709.2(11)	7550.7(10)	1746.0(8)	27.8(3)
C226	9012.7(12)	8002.5(11)	1410.7(8)	28.7(3)
C227	8167.4(12)	8209.2(10)	1657.0(7)	25.8(3)
C228	10631.1(14)	7344.1(12)	1484.9(11)	42.9(5)
C229	3246.4(10)	9840.8(9)	2389.5(7)	20.0(3)
C230	3319.8(11)	10759.0(10)	1577.4(7)	24.8(3)
C231	2734.4(11)	10278.4(10)	1502.3(7)	24.8(3)
C232	4207.9(11)	10921.0(9)	2392.5(8)	24.4(3)
C233	4702.4(13)	11472.0(10)	1920.5(9)	31.6(4)

Atom	x	y	z	U_{eq}
C234	4920.2(12)	10366.0(10)	2769.5(8)	28.6(3)
C235	3575.4(13)	11375.7(11)	2741.8(9)	33.6(4)
C236	2121.2(11)	8517.8(9)	3167.5(7)	22.3(3)
C237	764.1(13)	8052.0(12)	3287.5(8)	32.7(4)
C238	859.3(12)	8427.5(11)	2730.7(8)	31.1(4)
C239	1642.0(12)	7771.9(11)	4186.5(7)	28.1(3)
C240	1391.0(16)	6956.5(12)	4322.6(9)	39.7(5)
C241	2614.4(13)	7744.1(12)	4373.5(8)	33.6(4)
C242	984.1(14)	8259.8(13)	4498.4(9)	38.5(4)
C243	2685.1(11)	8376.6(10)	1807.8(8)	26.2(3)
C244	3243.9(14)	8530.5(13)	1306.3(10)	37.3(4)
C245	3776.0(14)	7959.3(14)	1111.4(10)	41.8(5)
C246	3756.2(15)	7197.1(13)	1395.3(10)	41.6(5)
C247	3163.8(19)	7036.5(13)	1868.2(11)	51.0(6)
C248	2655.8(16)	7612.8(12)	2076.0(9)	39.4(5)
C249	4366(2)	6576.2(17)	1205.8(12)	64.1(9)
C250	1183.9(11)	9430.9(10)	1636.6(7)	23.2(3)
C251	1020.2(11)	9278.9(10)	1115.3(7)	23.6(3)
C252	216.4(11)	9583.0(10)	814.6(7)	26.1(3)
C253	-454.8(11)	10057.7(10)	1021.9(8)	27.0(3)
C254	-298.3(12)	10228.8(11)	1536.0(8)	30.0(3)
C255	504.3(12)	9925.9(11)	1832.0(8)	28.1(3)
C256	-1332.3(13)	10370.7(14)	708.3(9)	40.7(5)
B201	6960.3(12)	8168.1(10)	2520.1(7)	19.5(3)
B202	2074.8(12)	9055.8(11)	2025.3(8)	22.4(3)

7.3.8 Anisotropic Displacement and Structural Parameters

Table S23. Anisotropic Displacement Parameters (×10^4) for **3-Br**. The anisotropic displacement factor exponent takes the form: $-2\pi^2[h^2a^{*2} \times U_{11} + ... +2hka^* \times b^* \times U_{12}]$

Atom	U_{11}	U_{22}	U_{33}	U_{23}	U_{13}	U_{12}
In11	16.24(4)	21.81(5)	16.31(4)	-3.32(3)	-0.34(3)	-7.44(4)
In12	16.20(4)	20.51(5)	15.20(4)	-4.05(3)	0.82(3)	-6.55(3)
Br11	26.14(8)	31.57(9)	39.35(10)	-13.04(7)	5.48(7)	-17.92(8)
Br12	17.88(7)	42.85(10)	31.32(9)	-0.50(6)	-2.40(6)	-11.37(8)
N101	24.1(6)	27.5(7)	17.8(6)	-4.1(5)	-3.4(5)	-5.5(5)
N102	22.3(6)	25.2(6)	14.7(5)	-3.9(5)	-0.2(4)	-3.8(5)
N103	20.3(5)	21.7(6)	16.0(5)	-4.2(4)	0.7(4)	-2.6(5)
N104	18.7(5)	20.9(6)	16.3(5)	-2.3(4)	0.9(4)	-1.8(4)
N105	21.7(6)	21.0(6)	18.0(5)	-5.8(5)	3.3(4)	-5.4(5)
N106	21.8(6)	20.7(6)	14.3(5)	-5.3(4)	0.3(4)	-4.0(4)
N107	22.1(6)	20.3(6)	14.8(5)	-3.5(4)	-1.0(4)	-1.8(4)
N108	21.0(5)	18.9(6)	14.7(5)	-4.7(4)	-1.3(4)	-2.4(4)
C101	20.9(6)	24.6(7)	16.8(6)	-3.6(5)	-1.6(5)	-6.2(5)
C102	28.5(8)	40.5(10)	16.2(7)	-4.7(7)	-3.3(6)	-3.9(6)
C103	26.6(8)	40.8(10)	15.8(7)	-4.1(7)	0.0(6)	-3.8(6)
C104	25.0(7)	31.4(8)	21.7(7)	-5.1(6)	-6.1(6)	-6.3(6)
C105	35.0(10)	38.1(11)	62.0(15)	-11.5(8)	-12.9(10)	-16.4(10)
C106	23.1(8)	106(2)	32.4(10)	4.1(11)	-6.5(7)	-29.5(13)
C107	34.6(10)	38.7(12)	60.2(15)	-3.4(9)	-17.1(10)	6.3(10)
C108	18.1(6)	19.0(6)	17.3(6)	-3.6(5)	0.1(5)	-2.7(5)
C109	22.9(7)	29.8(8)	19.6(7)	0.8(6)	-2.6(5)	-0.2(6)
C110	20.4(7)	32.4(8)	19.1(7)	0.7(6)	1.7(5)	-1.0(6)
C111	25.5(7)	26.8(8)	18.0(6)	-6.6(6)	0.6(5)	-2.4(6)
C112	29.0(8)	44.3(11)	19.7(7)	-0.8(7)	3.1(6)	-6.4(7)
C113	51.7(12)	33.4(10)	32.2(10)	-21.3(9)	4.3(9)	-1.5(8)
C114	33.7(9)	39.9(10)	17.8(7)	-3.3(7)	-2.4(6)	-2.3(7)
C115	19.5(6)	21.5(7)	16.3(6)	-1.5(5)	0.5(5)	-2.3(5)
C116	27.0(7)	24.1(7)	16.8(6)	-7.8(6)	-1.1(5)	-2.4(5)
C117	28.8(7)	25.2(8)	20.8(7)	-9.1(6)	-1.0(6)	-4.1(6)
C118	28.3(7)	21.0(7)	24.2(7)	-4.1(6)	-1.1(6)	-3.7(6)
C119	33.7(8)	21.3(7)	20.9(7)	-2.0(6)	-1.9(6)	-1.4(6)
C120	31.2(8)	22.2(7)	17.0(6)	-2.3(6)	-2.1(6)	-2.3(6)
C121	49.8(12)	25.0(9)	40.4(11)	-11.4(8)	-6.2(9)	-4.4(8)
C122	23.3(7)	22.5(7)	17.5(6)	0.1(5)	2.9(5)	-2.9(5)
C123	23.7(7)	29.1(8)	26.7(8)	-0.9(6)	5.2(6)	-2.4(6)
C124	25.9(8)	42.3(11)	26.2(8)	5.8(7)	5.7(6)	0.3(8)
C125	30.7(8)	48.9(12)	18.7(7)	13.7(8)	0.6(6)	-4.8(7)
C126	45.6(11)	38.0(10)	21.2(8)	11.0(8)	-4.9(7)	-11.9(7)
C127	36.4(9)	27.9(8)	20.3(7)	1.4(7)	-0.7(6)	-7.7(6)
C128	39.3(11)	82.0(19)	26.7(9)	22.3(11)	0.8(8)	-15.9(11)
C129	19.2(6)	21.9(7)	16.8(6)	-5.2(5)	1.9(5)	-5.9(5)
C130	27.4(7)	23.1(7)	16.1(6)	-6.2(6)	4.4(5)	-4.5(5)
C131	28.8(7)	22.0(7)	15.4(6)	-6.7(6)	1.7(5)	-4.1(5)
C132	24.0(7)	25.8(8)	25.3(7)	-8.5(6)	6.7(6)	-9.1(6)
C133	45.9(11)	22.6(8)	33.4(9)	-11.3(7)	9.9(8)	-6.9(7)
C134	22.9(8)	40.8(11)	49.0(12)	-8.1(7)	4.6(7)	-14.6(9)
C135	33.4(9)	39.4(10)	31.3(9)	-20.2(8)	9.4(7)	-17.8(8)
C136	18.3(6)	19.8(6)	14.8(6)	-2.6(5)	-0.9(5)	-4.3(5)
C137	25.3(7)	21.9(7)	17.7(6)	-6.1(5)	-0.5(5)	-1.1(5)
C138	26.9(7)	21.8(7)	17.1(6)	-8.2(5)	-1.2(5)	-3.3(5)
C139	24.6(7)	26.7(8)	14.0(6)	-1.0(6)	-1.8(5)	-3.0(5)
C140	28.3(8)	39.4(10)	22.5(8)	5.7(7)	-6.6(6)	-5.5(7)
C141	36.8(9)	33.8(9)	17.0(7)	-4.9(7)	1.4(6)	0.2(6)
C142	35.9(9)	31.9(9)	17.0(7)	-4.8(7)	-0.1(6)	-7.4(6)
C143	21.1(6)	19.9(7)	15.1(6)	-2.2(5)	-2.1(5)	-2.7(5)

Atom	U_{11}	U_{22}	U_{33}	U_{23}	U_{13}	U_{12}
C144	32.0(8)	24.9(8)	15.7(6)	1.1(6)	-0.3(6)	-2.1(6)
C145	34.9(9)	26.1(8)	18.1(7)	3.2(6)	-5.2(6)	-3.1(6)
C146	25.8(7)	28.4(8)	22.3(7)	0.4(6)	-7.0(6)	-4.2(6)
C147	22.3(7)	39.0(10)	22.4(8)	0.8(7)	-1.0(6)	-0.2(7)
C148	21.4(7)	32.5(9)	18.1(7)	0.3(6)	-0.8(5)	0.3(6)
C149	30.2(9)	43.1(11)	34.8(10)	6.4(8)	-11.4(7)	-3.7(8)
C150	22.5(6)	20.0(7)	16.1(6)	-3.5(5)	-2.6(5)	-4.8(5)
C151	26.9(7)	24.9(8)	22.4(7)	-2.6(6)	-7.3(6)	-8.6(6)
C152	33.0(8)	29.0(8)	26.7(8)	-5.0(7)	-8.4(6)	-11.9(7)
C153	33.3(8)	24.7(8)	22.8(7)	-6.5(6)	-0.4(6)	-9.3(6)
C154	29.2(8)	22.1(7)	23.3(7)	-1.4(6)	-0.4(6)	-7.2(6)
C155	23.1(7)	22.2(7)	21.6(7)	-2.1(5)	-2.8(5)	-5.7(6)
C156	56.2(13)	33.0(10)	37.1(10)	-8.7(9)	-2.2(9)	-19.7(9)
B101	18.7(7)	21.7(8)	15.7(7)	-3.4(6)	1.4(5)	-3.1(6)
B102	21.4(7)	19.4(7)	14.4(6)	-4.6(6)	-1.2(5)	-3.0(5)
In21	18.85(4)	18.51(5)	15.13(4)	-0.96(3)	0.11(3)	-3.46(3)
In22	18.55(4)	18.75(5)	17.27(4)	-1.24(3)	-1.52(3)	-2.97(4)
Br21	33.55(8)	32.14(9)	16.50(7)	-3.87(7)	1.16(6)	-0.76(6)
Br22	31.95(8)	33.55(9)	28.01(8)	0.77(7)	0.98(7)	-13.49(7)
N201	20.3(6)	19.5(6)	23.3(6)	-4.9(4)	1.4(5)	-5.9(5)
N202	20.4(5)	20.5(6)	18.1(5)	-3.8(4)	1.0(4)	-5.5(5)
N203	23.4(6)	21.2(6)	20.6(6)	-6.3(5)	1.2(5)	-6.8(5)
N204	29.1(7)	24.6(7)	23.3(6)	-5.3(5)	-1.6(5)	-9.8(5)
N205	19.9(6)	19.4(6)	22.8(6)	-2.3(4)	-1.2(5)	-2.2(5)
N206	21.3(6)	22.3(6)	18.8(6)	-3.3(5)	-1.6(4)	-2.5(5)
N207	22.6(6)	26.0(7)	22.6(6)	-7.0(5)	-1.5(5)	-4.5(5)
N208	24.7(6)	29.6(7)	23.5(7)	-7.5(5)	1.7(5)	-1.5(5)
C201	20.4(6)	17.9(6)	18.9(6)	-2.9(5)	1.6(5)	-3.9(5)
C202	23.0(7)	22.3(7)	26.4(7)	-5.5(5)	2.4(6)	-9.9(6)
C203	22.8(7)	23.7(7)	23.0(7)	-5.3(5)	3.4(5)	-9.7(6)
C204	22.9(7)	22.5(7)	31.4(8)	-6.9(6)	3.7(6)	-6.9(6)
C205	22.6(7)	28.2(8)	31.8(9)	-5.4(6)	2.3(6)	-9.7(7)
C206	33.9(9)	35.8(10)	55.4(13)	-16.7(8)	10.8(9)	-23.4(10)
C207	32.5(9)	28.4(9)	39.4(10)	-2.5(7)	4.3(8)	6.4(8)
C208	23.8(7)	20.7(7)	17.8(6)	-3.1(5)	-1.1(5)	-5.3(5)
C209	33.9(9)	36.8(10)	34.1(9)	-14.7(7)	1.3(7)	-19.1(8)
C210	27.6(8)	34.3(9)	31.2(9)	-13.6(7)	4.4(6)	-15.1(7)
C211	33.5(8)	27.8(8)	24.5(8)	1.0(7)	-5.2(6)	-12.4(7)
C212	35.5(9)	37.0(10)	27.3(8)	4.6(7)	-3.9(7)	-15.6(8)
C213	44.9(11)	48.4(12)	23.8(8)	9.9(9)	-8.7(8)	-10.0(8)
C214	59.6(14)	36.5(11)	49.4(13)	-10.7(10)	0.0(11)	-26.5(10)
C215	19.6(6)	21.3(7)	20.4(7)	-3.0(5)	0.3(5)	-4.5(5)
C216	25.0(7)	22.9(8)	30.3(8)	-6.2(6)	-3.4(6)	-2.3(6)
C217	25.8(7)	23.3(8)	30.3(8)	-4.3(6)	-1.7(6)	-0.2(6)
C218	29.1(8)	24.7(8)	24.8(8)	-0.7(6)	-3.1(6)	-3.1(6)
C219	31.3(8)	26.7(8)	29.1(8)	-4.8(6)	-8.6(7)	-3.5(7)
C220	26.4(7)	24.3(8)	23.8(7)	-5.4(6)	-4.1(6)	-3.9(6)
C221	44.5(11)	35.2(10)	31.0(9)	-1.2(8)	-10.7(8)	0.6(8)
C222	19.4(6)	22.7(7)	20.2(7)	-4.1(5)	1.4(5)	-4.8(6)
C223	21.3(7)	24.3(7)	24.5(7)	-5.3(5)	-2.4(5)	-3.9(6)
C224	20.3(7)	23.3(7)	36.9(9)	-4.3(6)	-1.6(6)	-8.0(7)
C225	22.4(7)	24.8(8)	38.6(10)	-6.5(6)	5.6(6)	-11.7(7)
C226	28.6(8)	31.2(9)	26.3(8)	-6.3(7)	8.1(6)	-7.7(7)
C227	25.3(7)	30.2(8)	20.1(7)	-3.6(6)	2.8(6)	-3.5(6)
C228	33.6(10)	31.0(10)	62.4(15)	-2.4(8)	17.4(9)	-13.2(10)
C229	20.2(6)	19.7(7)	19.1(6)	-2.6(5)	-1.3(5)	-3.1(5)
C230	22.1(7)	25.8(8)	23.0(7)	-2.6(6)	-1.1(5)	0.5(6)
C231	23.2(7)	29.5(8)	19.7(7)	-4.9(6)	-1.3(5)	-1.3(6)
C232	22.8(7)	21.7(7)	29.0(8)	-4.5(5)	-0.4(6)	-6.0(6)
C233	28.0(8)	25.2(8)	39.5(10)	-8.0(6)	-0.6(7)	-2.0(7)

Atom	U_{11}	U_{22}	U_{33}	U_{23}	U_{13}	U_{12}
C234	23.5(7)	27.1(8)	34.3(9)	-5.2(6)	-6.1(6)	-3.5(7)
C235	31.7(9)	29.6(9)	43.8(11)	-4.0(7)	1.7(8)	-17.4(8)
C236	22.2(7)	23.0(7)	21.4(7)	-5.5(5)	-0.1(5)	-3.9(6)
C237	28.0(8)	38.3(10)	31.1(9)	-14.3(7)	0.4(7)	-2.6(8)
C238	28.4(8)	36.2(10)	29.5(9)	-14.1(7)	-2.8(7)	-4.0(7)
C239	27.9(8)	29.9(9)	23.7(7)	-4.8(6)	5.4(6)	-2.4(6)
C240	47.4(12)	32.9(10)	33.9(10)	-11.6(9)	3.5(9)	2.7(8)
C241	31.4(9)	41.5(11)	21.5(8)	-3.8(7)	0.3(6)	3.0(7)
C242	36.8(10)	43.3(12)	31.4(10)	1.0(8)	7.2(8)	-6.7(8)
C243	24.0(7)	27.4(8)	29.2(8)	-0.8(6)	-5.7(6)	-10.4(7)
C244	33.3(9)	37.3(11)	46.7(12)	-7.4(8)	10.5(8)	-21.5(9)
C245	33.5(10)	49.6(13)	51.0(13)	-3.7(9)	3.0(9)	-30.0(11)
C246	44.1(11)	45.9(12)	38.6(11)	16.5(9)	-17.0(9)	-24.0(10)
C247	74.4(17)	30.1(11)	43.1(12)	15.6(11)	-7.6(12)	-8.3(9)
C248	54.6(12)	26.9(9)	31.5(10)	8.3(8)	-3.8(9)	-3.4(8)
C249	72.8(18)	68.1(18)	54.1(15)	37.4(15)	-24.5(13)	-37.1(14)
C250	20.3(6)	26.0(8)	24.3(7)	-2.9(5)	-2.9(5)	-7.2(6)
C251	22.6(7)	27.2(8)	21.4(7)	-4.4(6)	-1.5(5)	-5.8(6)
C252	25.0(7)	31.7(8)	21.2(7)	-5.6(6)	-3.6(6)	-4.1(6)
C253	19.3(7)	32.4(9)	27.2(8)	-4.7(6)	-2.5(6)	-2.2(7)
C254	22.8(7)	34.2(9)	33.0(9)	0.9(6)	-1.0(6)	-10.1(7)
C255	24.6(7)	33.9(9)	27.7(8)	-0.4(6)	-2.3(6)	-12.6(7)
C256	23.1(8)	55.5(13)	38.4(11)	-0.2(8)	-5.5(7)	-3.7(10)
B201	18.8(7)	21.9(8)	18.4(7)	-4.5(6)	1.5(5)	-5.3(6)
B202	21.4(7)	26.0(8)	20.8(8)	-3.7(6)	-1.8(6)	-6.8(6)

Table S24. Bond Lengths in Å for **3-Br.**

Atom	Atom	Length/Å
In11	In12	2.76287(15)
In11	Br11	2.6056(2)
In11	C101	2.2362(15)
In11	C108	2.2257(15)
In12	Br12	2.6075(2)
In12	C129	2.2278(15)
In12	C136	2.2277(15)
N101	C101	1.3618(19)
N101	C102	1.381(2)
N101	C104	1.493(2)
N102	C101	1.356(2)
N102	C103	1.382(2)
N102	B101	1.600(2)
N103	C108	1.3684(19)
N103	C109	1.386(2)
N103	C111	1.494(2)
N104	C108	1.3520(19)
N104	C110	1.379(2)
N104	B101	1.584(2)
N105	C129	1.3614(19)
N105	C130	1.382(2)
N105	C132	1.497(2)
N106	C129	1.3585(19)
N106	C131	1.3843(19)
N106	B102	1.588(2)
N107	C136	1.3680(19)
N107	C137	1.379(2)
N107	C139	1.4978(19)
N108	C136	1.3534(18)
N108	C138	1.3814(19)
N108	B102	1.592(2)
C102	C103	1.358(2)
C104	C105	1.513(3)
C104	C106	1.509(3)
C104	C107	1.531(3)
C109	C110	1.355(2)
C111	C112	1.525(3)
C111	C113	1.526(3)
C111	C114	1.532(2)
C115	C116	1.396(2)
C115	C120	1.405(2)
C115	B101	1.617(2)
C116	C117	1.394(2)
C117	C118	1.392(2)
C118	C119	1.395(2)
C118	C121	1.503(3)
C119	C120	1.390(2)
C122	C123	1.401(2)
C122	C127	1.402(2)
C122	B101	1.636(2)
C123	C124	1.394(2)
C124	C125	1.392(3)
C125	C126	1.389(3)
C125	C128	1.508(3)
C126	C127	1.404(3)
C130	C131	1.354(2)
C132	C133	1.537(3)
C132	C134	1.527(3)

Atom	Atom	Length/Å
C132	C135	1.521(2)
C137	C138	1.354(2)
C139	C140	1.526(2)
C139	C141	1.533(2)
C139	C142	1.525(2)
C143	C144	1.403(2)
C143	C148	1.396(2)
C143	B102	1.617(2)
C144	C145	1.389(2)
C145	C146	1.397(2)
C146	C147	1.393(2)
C146	C149	1.505(2)
C147	C148	1.395(2)
C150	C151	1.397(2)
C150	C155	1.405(2)
C150	B102	1.632(2)
C151	C152	1.404(2)
C152	C153	1.389(3)
C153	C154	1.400(2)
C153	C156	1.507(2)
C154	C155	1.388(2)
In21	In22	2.77507(15)
In21	Br21	2.6231(2)
In21	C201	2.2285(15)
In21	C208	2.2316(16)
In22	Br22	2.6241(2)
In22	C229	2.2302(15)
In22	C236	2.2327(16)
N201	C201	1.372(2)
N201	C202	1.382(2)
N201	C204	1.494(2)
N202	C201	1.3520(19)
N202	C203	1.381(2)
N202	B201	1.588(2)
N203	C208	1.357(2)
N203	C210	1.379(2)
N203	B201	1.599(2)
N204	C208	1.366(2)
N204	C209	1.380(2)
N204	C211	1.496(2)
N205	C229	1.368(2)
N205	C230	1.379(2)
N205	C232	1.501(2)
N206	C229	1.3549(19)
N206	C231	1.376(2)
N206	B202	1.593(2)
N207	C236	1.357(2)
N207	C238	1.380(2)
N207	B202	1.592(2)
N208	C236	1.364(2)

Atom	Atom	Length/Å
N208	C237	1.387(2)
N208	C239	1.491(2)
C202	C203	1.353(2)
C204	C205	1.524(2)
C204	C206	1.537(3)
C204	C207	1.528(3)
C209	C210	1.354(2)
C211	C212	1.518(3)
C211	C213	1.527(3)
C211	C214	1.530(3)
C215	C216	1.409(2)
C215	C220	1.395(2)
C215	B201	1.614(2)
C216	C217	1.386(2)
C217	C218	1.400(2)
C218	C219	1.385(3)
C218	C221	1.508(3)
C219	C220	1.396(2)
C222	C223	1.403(2)
C222	C227	1.396(2)
C222	B201	1.632(2)
C223	C224	1.396(2)
C224	C225	1.395(3)
C225	C226	1.389(3)
C225	C228	1.505(3)
C226	C227	1.396(2)
C230	C231	1.358(2)
C232	C233	1.531(2)
C232	C234	1.526(2)
C232	C235	1.531(3)
C237	C238	1.351(3)
C239	C240	1.537(3)
C239	C241	1.515(3)
C239	C242	1.532(3)
C243	C244	1.408(3)
C243	C248	1.392(3)
C243	B202	1.623(3)
C244	C245	1.392(3)
C245	C246	1.396(3)
C246	C247	1.385(4)
C246	C249	1.508(3)
C247	C248	1.400(3)
C250	C251	1.397(2)
C250	C255	1.408(2)
C250	B202	1.629(2)
C251	C252	1.400(2)
C252	C253	1.390(3)
C253	C254	1.394(3)
C253	C256	1.507(2)
C254	C255	1.393(2)

Table S25. Bond Angles in ° for **3-Br.**

Atom	Atom	Atom	Angle/°
Br11	In11	In12	113.695(6)
C101	In11	In12	122.45(4)
C101	In11	Br11	100.10(4)
C108	In11	In12	122.99(4)
C108	In11	Br11	99.17(4)
C108	In11	C101	93.62(5)

Atom	Atom	Atom	Angle/°
Br12	In12	In11	114.157(6)
C129	In12	In11	125.16(4)
C129	In12	Br12	97.04(4)
C136	In12	In11	122.72(4)
C136	In12	Br12	98.28(4)
C136	In12	C129	93.57(5)

Atom	Atom	Atom	Angle/°
C102	N101	C101	109.47(13)
C104	N101	C101	130.89(14)
C104	N101	C102	119.61(13)
C103	N102	C101	108.84(13)
B101	N102	C101	128.48(13)
B101	N102	C103	121.54(13)
C109	N103	C108	109.19(12)
C111	N103	C108	128.66(13)
C111	N103	C109	121.89(13)
C110	N104	C108	109.08(13)
B101	N104	C108	130.10(13)
B101	N104	C110	119.81(12)
C130	N105	C129	109.48(13)
C132	N105	C129	130.56(13)
C132	N105	C130	119.96(13)
C131	N106	C129	108.85(12)
B102	N106	C129	129.01(12)
B102	N106	C131	121.24(12)
C137	N107	C136	109.37(12)
C139	N107	C136	128.24(13)
C139	N107	C137	121.87(13)
C138	N108	C136	109.01(12)
B102	N108	C136	130.82(12)
B102	N108	C138	119.83(12)
N101	C101	In11	134.43(11)
N102	C101	In11	118.42(10)
N102	C101	N101	106.91(13)
C103	C102	N101	106.80(14)
C102	C103	N102	107.97(15)
C105	C104	N101	108.33(15)
C106	C104	N101	111.81(13)
C106	C104	C105	110.49(19)
C107	C104	N101	107.69(15)
C107	C104	C105	110.59(17)
C107	C104	C106	107.91(19)
N103	C108	In11	134.79(11)
N104	C108	In11	118.38(10)
N104	C108	N103	106.80(12)
C110	C109	N103	106.68(14)
C109	C110	N104	108.23(14)
C112	C111	N103	109.89(14)
C113	C111	N103	108.36(14)
C113	C111	C112	111.44(16)
C114	C111	N103	109.09(13)
C114	C111	C112	108.79(15)
C114	C111	C113	109.23(16)
C120	C115	C116	116.33(15)
B101	C115	C116	123.71(13)
B101	C115	C120	119.27(14)
C117	C116	C115	121.80(15)
C118	C117	C116	121.13(15)
C119	C118	C117	117.82(15)
C121	C118	C117	120.93(16)
C121	C118	C119	121.24(16)
C120	C119	C118	120.75(15)
C119	C120	C115	122.08(15)
C127	C122	C123	116.19(15)
B101	C122	C123	121.41(15)
B101	C122	C127	122.38(15)
C124	C123	C122	122.37(18)

Atom	Atom	Atom	Angle/°
C125	C124	C123	120.89(19)
C126	C125	C124	117.57(17)
C128	C125	C124	120.6(2)
C128	C125	C126	121.8(2)
C127	C126	C125	121.53(19)
C126	C127	C122	121.30(18)
N105	C129	In12	134.26(11)
N106	C129	In12	117.78(10)
N106	C129	N105	106.77(13)
C131	C130	N105	106.93(13)
C130	C131	N106	107.95(14)
C133	C132	N105	107.73(14)
C134	C132	N105	107.67(14)
C134	C132	C133	110.99(15)
C135	C132	N105	111.46(13)
C135	C132	C133	108.24(15)
C135	C132	C134	110.72(16)
N107	C136	In12	135.16(10)
N108	C136	In12	118.06(10)
N108	C136	N107	106.65(12)
C138	C137	N107	106.93(13)
C137	C138	N108	108.02(13)
C140	C139	N107	108.57(13)
C141	C139	N107	109.41(13)
C141	C139	C140	108.86(14)
C142	C139	N107	109.72(13)
C142	C139	C140	111.46(14)
C142	C139	C141	108.80(14)
C148	C143	C144	115.82(14)
B102	C143	C144	121.31(14)
B102	C143	C148	122.63(13)
C145	C144	C143	122.39(16)
C146	C145	C144	121.03(15)
C147	C146	C145	117.21(15)
C149	C146	C145	121.95(16)
C149	C146	C147	120.84(17)
C148	C147	C146	121.31(16)
C147	C148	C143	122.09(15)
C155	C150	C151	116.17(14)
B102	C150	C151	124.19(14)
B102	C150	C155	119.50(13)
C152	C151	C150	121.60(16)
C153	C152	C151	121.15(15)
C154	C153	C152	118.01(15)
C156	C153	C152	121.82(17)
C156	C153	C154	120.17(17)
C155	C154	C153	120.32(16)
C154	C155	C150	122.73(15)
N104	B101	N102	111.69(12)
C115	B101	N102	109.06(12)
C115	B101	N104	109.21(12)
C122	B101	N102	107.93(12)
C122	B101	N104	108.95(12)
C122	B101	C115	109.99(12)
N108	B102	N106	111.12(11)
C143	B102	N106	109.88(12)
C143	B102	N108	109.34(12)
C150	B102	N106	107.79(12)
C150	B102	N108	105.43(12)
C150	B102	C143	113.22(12)

Atom	Atom	Atom	Angle/°
Br21	In21	In22	115.138(6)
C201	In21	In22	123.55(4)
C201	In21	Br21	99.14(4)
C208	In21	In22	122.93(4)
C208	In21	Br21	97.35(4)
C208	In21	C201	93.05(6)
Br22	In22	In21	112.595(6)
C229	In22	In21	121.71(4)
C229	In22	Br22	101.43(4)
C236	In22	In21	124.43(4)
C236	In22	Br22	99.14(4)
C236	In22	C229	92.94(6)
C202	N201	C201	109.27(13)
C204	N201	C201	128.06(13)
C204	N201	C202	122.23(13)
C203	N202	C201	109.20(13)
B201	N202	C201	129.19(13)
B201	N202	C203	121.17(13)
C210	N203	C208	109.19(13)
B201	N203	C208	127.64(13)
B201	N203	C210	121.95(13)
C209	N204	C208	108.96(14)
C211	N204	C208	130.52(14)
C211	N204	C209	120.48(14)
C230	N205	C229	109.30(13)
C232	N205	C229	127.99(13)
C232	N205	C230	122.25(14)
C231	N206	C229	109.10(13)
B202	N206	C229	132.23(13)
B202	N206	C231	118.54(13)
C238	N207	C236	109.19(14)
B202	N207	C236	130.49(13)
B202	N207	C238	118.99(14)
C237	N208	C236	109.16(14)
C239	N208	C236	130.57(14)
C239	N208	C237	120.19(14)
N201	C201	In21	135.71(11)
N202	C201	In21	117.71(11)
N202	C201	N201	106.53(13)
C203	C202	N201	106.87(14)
C202	C203	N202	108.12(14)
C205	C204	N201	109.79(14)
C206	C204	N201	108.90(14)
C206	C204	C205	109.05(15)
C207	C204	N201	108.37(14)
C207	C204	C205	111.31(15)
C207	C204	C206	109.38(16)
N203	C208	In21	117.67(10)
N204	C208	In21	135.36(12)
N204	C208	N203	106.76(13)
C210	C209	N204	107.48(15)
C209	C210	N203	107.61(15)
C212	C211	N204	111.46(14)
C213	C211	N204	108.00(15)
C213	C211	C212	110.19(17)
C214	C211	N204	108.53(16)
C214	C211	C212	108.36(17)
C214	C211	C213	110.28(17)
C220	C215	C216	116.29(15)
B201	C215	C216	118.33(14)

Atom	Atom	Atom	Angle/°
B201	C215	C220	124.78(14)
C217	C216	C215	121.96(16)
C218	C217	C216	120.91(16)
C219	C218	C217	117.56(16)
C221	C218	C217	121.61(17)
C221	C218	C219	120.81(17)
C220	C219	C218	121.50(16)
C219	C220	C215	121.64(16)
C227	C222	C223	116.28(15)
B201	C222	C223	122.04(14)
B201	C222	C227	121.56(14)
C224	C223	C222	121.84(16)
C225	C224	C223	120.93(16)
C226	C225	C224	117.81(16)
C228	C225	C224	121.02(18)
C228	C225	C226	121.17(18)
C227	C226	C225	120.95(17)
C226	C227	C222	122.16(16)
N205	C229	In22	134.53(11)
N206	C229	In22	118.52(11)
N206	C229	N205	106.69(13)
C231	C230	N205	106.84(14)
C230	C231	N206	108.06(14)
C233	C232	N205	109.82(14)
C234	C232	N205	109.15(13)
C234	C232	C233	109.11(14)
C235	C232	N205	108.56(13)
C235	C232	C233	108.82(15)
C235	C232	C234	111.37(15)
N207	C236	In22	118.70(11)
N208	C236	In22	134.35(12)
N208	C236	N207	106.68(14)
C238	C237	N208	107.03(15)
C237	C238	N207	107.94(16)
C240	C239	N208	107.93(15)
C241	C239	N208	111.74(14)
C241	C239	C240	108.29(16)
C242	C239	N208	107.93(15)
C242	C239	C240	111.14(16)
C242	C239	C241	109.82(17)
C248	C243	C244	115.52(18)
B202	C243	C244	121.08(17)
B202	C243	C248	123.29(17)
C245	C244	C243	122.2(2)
C246	C245	C244	121.3(2)
C247	C246	C245	116.93(19)
C249	C246	C245	121.7(2)
C249	C246	C247	121.4(2)
C248	C247	C246	121.6(2)
C247	C248	C243	122.2(2)
C255	C250	C251	115.90(15)
B202	C250	C251	124.32(15)
B202	C250	C255	119.74(15)
C252	C251	C250	121.97(16)
C253	C252	C251	121.13(16)
C254	C253	C252	117.89(15)
C256	C253	C252	121.40(17)
C256	C253	C254	120.70(17)
C255	C254	C253	120.66(17)
C254	C255	C250	122.42(16)

Atom	Atom	Atom	Angle/°
N203	B201	N202	111.10(12)
C215	B201	N202	110.74(12)
C215	B201	N203	108.75(13)
C222	B201	N202	106.87(12)
C222	B201	N203	108.96(12)
C222	B201	C215	110.40(13)

Atom	Atom	Atom	Angle/°
N207	B202	N206	111.44(13)
C243	B202	N206	109.81(13)
C243	B202	N207	108.43(14)
C250	B202	N206	106.97(13)
C250	B202	N207	106.58(13)
C250	B202	C243	113.60(13)

Table S26. Torsion Angles in ° for **3-Br**.

Atom	Atom	Atom	Atom	Angle/°
In11	C101	N101	C102	-173.97(17)
In11	C101	N101	C104	7.8(2)
In11	C101	N102	C103	175.46(13)
In11	C101	N102	B101	-16.74(16)
In11	C108	N103	C109	-177.62(16)
In11	C108	N103	C111	8.27(19)
In11	C108	N104	C110	177.59(12)
In11	C108	N104	B101	9.36(15)
In12	C129	N105	C130	-166.12(15)
In12	C129	N105	C132	13.6(2)
In12	C129	N106	C131	169.09(11)
In12	C129	N106	B102	-21.88(15)
In12	C136	N107	C137	-174.69(15)
In12	C136	N107	C139	13.53(18)
In12	C136	N108	C138	175.01(11)
In12	C136	N108	B102	1.93(15)
N101	C101	N102	C103	0.29(16)
N101	C101	N102	B101	168.09(11)
N101	C102	C103	N102	0.58(17)
N102	C101	N101	C102	0.08(16)
N102	C101	N101	C104	-178.20(12)
N102	B101	N104	C108	-44.80(17)
N102	B101	N104	C110	148.03(13)
N102	B101	C115	C116	134.09(12)
N102	B101	C115	C120	-55.73(14)
N102	B101	C122	C123	132.06(13)
N102	B101	C122	C127	-46.35(15)
N103	C108	N104	C110	-0.93(15)
N103	C108	N104	B101	-169.15(11)
N103	C109	C110	N104	-0.62(16)
N104	C108	N103	C109	0.54(15)
N104	C108	N103	C111	-173.57(12)
N104	B101	N102	C101	49.17(17)
N104	B101	N102	C103	-144.40(14)
N104	B101	C115	C116	11.79(15)
N104	B101	C115	C120	-178.03(12)
N104	B101	C122	C123	-106.50(14)
N104	B101	C122	C127	75.09(14)
N105	C129	N106	C131	-0.22(14)
N105	C129	N106	B102	168.80(11)
N105	C130	C131	N106	0.66(14)
N106	C129	N105	C130	0.64(15)
N106	C129	N105	C132	-179.62(12)
N106	B102	N108	C136	-39.29(17)
N106	B102	N108	C138	148.26(12)
N106	B102	C143	C144	-51.18(15)
N106	B102	C143	C148	134.67(13)
N106	B102	C150	C151	121.60(13)
N106	B102	C150	C155	-62.85(14)
N107	C136	N108	C138	-1.40(14)

Atom	Atom	Atom	Atom	Angle/°
N107	C136	N108	B102	-174.48(11)
N107	C137	C138	N108	-0.95(14)
N108	C136	N107	C137	0.81(14)
N108	C136	N107	C139	-170.97(11)
N108	B102	N106	C129	51.77(17)
N108	B102	N106	C131	-140.39(13)
N108	B102	C143	C144	-173.39(12)
N108	B102	C143	C148	12.47(16)
N108	B102	C150	C151	-119.65(13)
N108	B102	C150	C155	55.91(14)
C101	N101	C102	C103	-0.41(16)
C101	N101	C104	C105	-117.4(2)
C101	N101	C104	C106	4.6(2)
C101	N101	C104	C107	122.9(2)
C101	N102	C103	C102	-0.55(15)
C101	N102	B101	C115	-71.63(17)
C101	N102	B101	C122	168.90(17)
C102	N101	C104	C105	64.45(18)
C102	N101	C104	C106	-173.6(2)
C102	N101	C104	C107	-55.20(19)
C102	C103	N102	B101	-169.36(14)
C103	N102	B101	C115	94.80(16)
C103	N102	B101	C122	-24.67(16)
C103	C102	N101	C104	178.09(14)
C108	N103	C109	C110	0.05(14)
C108	N103	C111	C112	-35.98(17)
C108	N103	C111	C113	86.01(18)
C108	N103	C111	C114	-155.17(18)
C108	N104	C110	C109	0.98(15)
C108	N104	B101	C115	75.91(17)
C108	N104	B101	C122	-163.93(17)
C109	N103	C111	C112	150.58(17)
C109	N103	C111	C113	-87.44(17)
C109	N103	C111	C114	31.39(17)
C109	C110	N104	B101	170.61(13)
C110	N104	B101	C115	-91.25(15)
C110	N104	B101	C122	28.91(15)
C110	C109	N103	C111	174.64(13)
C115	C116	C117	C118	-0.7(2)
C115	C120	C119	C118	-1.3(2)
C115	B101	C122	C123	13.17(16)
C115	B101	C122	C127	-165.23(13)
C116	C115	C120	C119	3.03(18)
C116	C115	B101	C122	-107.73(16)
C116	C117	C118	C119	2.5(2)
C116	C117	C118	C121	-178.80(17)
C117	C116	C115	C120	-2.03(19)
C117	C116	C115	B101	168.42(15)
C117	C118	C119	C120	-1.50(19)
C119	C120	C115	B101	-167.87(15)
C120	C115	B101	C122	62.46(15)
C120	C119	C118	C121	179.79(18)
C122	C123	C124	C125	1.1(2)
C122	C127	C126	C125	1.7(2)
C123	C122	C127	C126	-3.84(18)
C123	C124	C125	C126	-3.4(2)
C123	C124	C125	C128	175.27(18)
C124	C123	C122	C127	2.5(2)
C124	C123	C122	B101	-176.03(16)
C124	C125	C126	C127	2.0(2)

Atom	Atom	Atom	Atom	Angle/°
C126	C127	C122	B101	174.65(15)
C127	C126	C125	C128	-176.63(18)
C129	N105	C130	C131	-0.82(14)
C129	N105	C132	C133	127.36(18)
C129	N105	C132	C134	-112.87(19)
C129	N105	C132	C135	8.76(19)
C129	N106	C131	C130	-0.28(14)
C129	N106	B102	C143	-69.38(16)
C129	N106	B102	C150	166.82(16)
C130	N105	C132	C133	-52.92(16)
C130	N105	C132	C134	66.85(16)
C130	N105	C132	C135	-171.53(17)
C130	C131	N106	B102	-170.32(12)
C131	N106	B102	C143	98.46(15)
C131	N106	B102	C150	-25.34(14)
C131	C130	N105	C132	179.41(13)
C136	N107	C137	C138	0.09(14)
C136	N107	C139	C140	80.18(17)
C136	N107	C139	C141	-161.14(17)
C136	N107	C139	C142	-41.85(17)
C136	N108	C138	C137	1.48(14)
C136	N108	B102	C143	82.17(16)
C136	N108	B102	C150	-155.80(17)
C137	N107	C139	C140	-90.68(16)
C137	N107	C139	C141	28.00(16)
C137	N107	C139	C142	147.29(16)
C137	C138	N108	B102	175.45(12)
C138	N108	B102	C143	-90.28(14)
C138	N108	B102	C150	31.74(14)
C138	C137	N107	C139	172.49(12)
C143	C144	C145	C146	-0.4(2)
C143	C148	C147	C146	0.1(2)
C143	B102	C150	C151	-0.16(16)
C143	B102	C150	C155	175.39(13)
C144	C143	C148	C147	-3.26(18)
C144	C143	B102	C150	69.39(16)
C144	C145	C146	C147	-2.9(2)
C144	C145	C146	C149	176.21(18)
C145	C144	C143	C148	3.4(2)
C145	C144	C143	B102	-171.11(16)
C145	C146	C147	C148	3.0(2)
C147	C148	C143	B102	171.18(16)
C148	C143	B102	C150	-104.75(16)
C148	C147	C146	C149	-176.07(19)
C150	C151	C152	C153	0.2(2)
C150	C155	C154	C153	-0.27(19)
C151	C150	C155	C154	1.21(18)
C151	C152	C153	C154	0.8(2)
C151	C152	C153	C156	-179.37(18)
C152	C151	C150	C155	-1.1(2)
C152	C151	C150	B102	174.54(15)
C152	C153	C154	C155	-0.8(2)
C154	C155	C150	B102	-174.69(15)
C155	C154	C153	C156	179.42(17)
In21	C201	N201	C202	176.54(16)
In21	C201	N201	C204	-10.99(19)
In21	C201	N202	C203	-176.64(11)
In21	C201	N202	B201	-4.32(15)
In21	C208	N203	C210	-176.10(13)
In21	C208	N203	B201	16.47(15)

Atom	Atom	Atom	Atom	Angle/°
In21	C208	N204	C209	174.32(17)
In21	C208	N204	C211	-3.3(2)
In22	C229	N205	C230	172.96(15)
In22	C229	N205	C232	-14.78(19)
In22	C229	N206	C231	-173.85(12)
In22	C229	N206	B202	1.79(17)
In22	C236	N207	C238	-175.14(14)
In22	C236	N207	B202	18.49(17)
In22	C236	N208	C237	173.59(18)
In22	C236	N208	C239	-3.1(2)
N201	C201	N202	C203	1.10(14)
N201	C201	N202	B201	173.41(11)
N201	C202	C203	N202	0.80(15)
N202	C201	N201	C202	-0.60(14)
N202	C201	N201	C204	171.88(12)
N202	B201	N203	C208	-52.92(16)
N202	B201	N203	C210	141.10(14)
N202	B201	C215	C216	179.89(13)
N202	B201	C215	C220	-9.40(17)
N202	B201	C222	C223	-70.47(14)
N202	B201	C222	C227	105.44(14)
N203	C208	N204	C209	-0.05(16)
N203	C208	N204	C211	-177.65(12)
N203	C210	C209	N204	-0.98(17)
N203	B201	N202	C201	45.37(17)
N203	B201	N202	C203	-143.11(13)
N203	B201	C215	C216	57.54(15)
N203	B201	C215	C220	-131.75(13)
N203	B201	C222	C223	49.67(15)
N203	B201	C222	C227	-134.42(13)
N204	C208	N203	C210	-0.56(16)
N204	C208	N203	B201	-167.99(12)
N205	C229	N206	C231	1.17(15)
N205	C229	N206	B202	176.82(12)
N205	C230	C231	N206	0.42(15)
N206	C229	N205	C230	-0.91(15)
N206	C229	N205	C232	171.35(11)
N206	B202	N207	C236	-45.24(18)
N206	B202	N207	C238	149.51(14)
N206	B202	C243	C244	-46.53(17)
N206	B202	C243	C248	137.39(15)
N206	B202	C250	C251	111.82(14)
N206	B202	C250	C255	-70.22(15)
N207	C236	N208	C237	-0.13(17)
N207	C236	N208	C239	-176.78(13)
N207	C238	C237	N208	-0.62(18)
N207	B202	N206	C229	32.70(19)
N207	B202	N206	C231	-151.99(13)
N207	B202	C243	C244	-168.51(14)
N207	B202	C243	C248	15.41(18)
N207	B202	C250	C251	-128.86(13)
N207	B202	C250	C255	49.10(16)
N208	C236	N207	C238	-0.26(17)
N208	C236	N207	B202	-166.62(13)
C201	N201	C202	C203	-0.14(14)
C201	N201	C204	C205	38.02(17)
C201	N201	C204	C206	157.36(18)
C201	N201	C204	C207	-83.75(18)
C201	N202	C203	C202	-1.21(14)
C201	N202	B201	C215	-75.59(16)

Atom	Atom	Atom	Atom	Angle/˚
C201	N202	B201	C222	164.13(17)
C202	N201	C204	C205	-150.38(16)
C202	N201	C204	C206	-31.04(17)
C202	N201	C204	C207	87.85(17)
C202	C203	N202	B201	-174.25(12)
C203	N202	B201	C215	95.93(15)
C203	N202	B201	C222	-24.36(14)
C203	C202	N201	C204	-173.14(13)
C208	N203	C210	C209	0.97(16)
C208	N203	B201	C215	69.21(16)
C208	N203	B201	C222	-170.41(17)
C208	N204	C209	C210	0.65(16)
C208	N204	C211	C212	-20.9(2)
C208	N204	C211	C213	100.2(2)
C208	N204	C211	C214	-140.2(2)
C209	N204	C211	C212	161.69(18)
C209	N204	C211	C213	-77.13(19)
C209	N204	C211	C214	42.43(19)
C209	C210	N203	B201	169.25(14)
C210	N203	B201	C215	-96.77(16)
C210	N203	B201	C222	23.61(16)
C210	C209	N204	C211	178.53(15)
C215	C216	C217	C218	0.6(2)
C215	C220	C219	C218	0.4(2)
C215	B201	C222	C223	169.03(13)
C215	B201	C222	C227	-15.06(16)
C216	C215	C220	C219	2.78(19)
C216	C215	B201	C222	-61.95(16)
C216	C217	C218	C219	2.6(2)
C216	C217	C218	C221	-175.84(18)
C217	C216	C215	C220	-3.3(2)
C217	C216	C215	B201	168.24(16)
C217	C218	C219	C220	-3.1(2)
C219	C220	C215	B201	-168.10(16)
C220	C215	B201	C222	108.76(16)
C220	C219	C218	C221	175.37(18)
C222	C223	C224	C225	-1.53(19)
C222	C227	C226	C225	-1.3(2)
C223	C222	C227	C226	-0.12(18)
C223	C224	C225	C226	0.08(19)
C223	C224	C225	C228	179.65(17)
C224	C223	C222	C227	1.51(19)
C224	C223	C222	B201	177.62(15)
C224	C225	C226	C227	1.29(19)
C226	C227	C222	B201	-176.25(16)
C227	C226	C225	C228	-178.28(18)
C229	N205	C230	C231	0.30(14)
C229	N205	C232	C233	164.85(17)
C229	N205	C232	C234	45.27(17)
C229	N205	C232	C235	-76.29(18)
C229	N206	C231	C230	-1.01(14)
C229	N206	B202	C243	-87.48(18)
C229	N206	B202	C250	148.83(19)
C230	N205	C232	C233	-23.80(16)
C230	N205	C232	C234	-143.37(16)
C230	N205	C232	C235	95.06(17)
C230	C231	N206	B202	-177.34(13)
C231	N206	B202	C243	87.84(15)
C231	N206	B202	C250	-35.86(15)
C231	C230	N205	C232	-172.49(13)

Atom	Atom	Atom	Atom	Angle/˚
C236	N207	C238	C237	0.56(16)
C236	N207	B202	C243	75.75(18)
C236	N207	B202	C250	-161.60(19)
C236	N208	C237	C238	0.47(17)
C236	N208	C239	C240	-134.6(2)
C236	N208	C239	C241	-15.6(2)
C236	N208	C239	C242	105.2(2)
C237	N208	C239	C240	49.07(19)
C237	N208	C239	C241	168.03(19)
C237	N208	C239	C242	-71.13(19)
C237	C238	N207	B202	168.73(15)
C238	N207	B202	C243	-89.51(16)
C238	N207	B202	C250	33.14(16)
C238	C237	N208	C239	177.53(15)
C243	C244	C245	C246	2.3(2)
C243	C248	C247	C246	2.8(3)
C243	B202	C250	C251	-9.52(18)
C243	B202	C250	C255	168.44(15)
C244	C243	C248	C247	1.4(2)
C244	C243	B202	C250	73.20(18)
C244	C245	C246	C247	1.9(3)
C244	C245	C246	C249	-176.8(2)
C245	C244	C243	C248	-3.8(2)
C245	C244	C243	B202	179.79(18)
C245	C246	C247	C248	-4.3(2)
C247	C248	C243	B202	177.6(2)
C248	C243	B202	C250	-102.88(18)
C248	C247	C246	C249	174.3(2)
C250	C251	C252	C253	0.5(2)
C250	C255	C254	C253	-0.9(2)
C251	C250	C255	C254	2.01(19)
C251	C252	C253	C254	0.7(2)
C251	C252	C253	C256	-178.39(17)
C252	C251	C250	C255	-1.81(19)
C252	C251	C250	B202	176.22(15)
C252	C253	C254	C255	-0.5(2)
C254	C255	C250	B202	-176.11(17)
C255	C254	C253	C256	178.59(18)

Table S27. Hydrogen Fractional Atomic Coordinates (×10[4]) and Equivalent Isotropic Displacement Parameters (Å[2]×10[3]) for **3-Br**. U_{eq} is defined as 1/3 of the trace of the orthogonalised U_{ij}.

Atom	x	y	z	U_{eq}
H102	8277.7(12)	2731.9(11)	-331.1(7)	34.6(4)
H103	10028.2(12)	2494.7(11)	-33.0(7)	34.1(4)
H10a	6943(9)	2158(6)	-25(4)	64.8(8)
H10b	6820(10)	1683(2)	718(4)	64.8(8)
H10c	5885.7(19)	2316(5)	359(7)	64.8(8)
H10d	6676(11)	2436(4)	1449(2)	78.4(10)
H10e	6833(10)	3421(8)	1237(4)	78.4(10)
H10f	5802(2)	3112(11)	1063(3)	78.4(10)
H10g	7099(10)	4052(3)	196(4)	71.1(9)
H10h	7018(11)	3524(3)	-325.1(16)	71.1(9)
H10i	6019.9(17)	3760(6)	86(5)	71.1(9)
H109	11591.6(11)	933.1(10)	2868.3(7)	30.6(4)
H110	11882.3(11)	1560.1(10)	1759.2(7)	30.5(4)
H11a	8685(7)	1526(5)	3828(2)	47.6(6)
H11b	9171(3)	2276(2)	3316(6)	47.6(6)
H11c	8373(4)	1755(6)	3092(4)	47.6(6)
H11d	9095(10)	152(5)	3688(2)	58.7(7)
H11e	8881(8)	485.1(18)	2937(5)	58.7(7)
H11f	9982(3)	7(3)	3173(7)	58.7(7)
H11g	10755(7)	1550(3)	3677(4)	47.0(6)
H11h	10113(2)	864(8)	4147.5(7)	47.0(6)
H11i	10977(6)	572(6)	3668(4)	47.0(6)
H116	10751.6(11)	3113.1(9)	2071.2(7)	27.2(3)
H117	11175.3(11)	4385.3(10)	2032.9(7)	29.7(4)
H119	10698.1(12)	5076.8(10)	218.2(7)	31.3(4)
H120	10229.6(12)	3818.7(9)	263.2(7)	28.8(4)
H12a	12020(2)	5531(2)	1145(7)	57.5(7)
H12b	11087(10)	5973(3)	684(3)	57.5(7)
H12c	10922(9)	5797(4)	1447(4)	57.5(7)
H123	12067.5(11)	3066.6(10)	772.8(8)	33.3(4)
H124	13320.4(13)	2652.6(12)	153.5(8)	41.1(5)
H126	11859.8(15)	817.8(12)	30.2(8)	42.4(5)
H127	10630.2(13)	1197.7(10)	686.6(7)	34.0(4)
H12d	13246(8)	1872(8)	-822.0(17)	77.4(10)
H12e	14065(2)	1605(11)	-263(5)	77.4(10)
H12f	13392(9)	917(3)	-388(6)	77.4(10)
H130	6121.5(11)	3349.9(9)	4616.1(7)	26.7(3)
H131	7388.8(11)	4302.7(9)	4453.6(6)	26.3(3)
H13a	5637(8)	1581(4)	4228(3)	50.7(6)
H13b	6803(3)	1754(6)	4262(4)	50.7(6)
H13c	5907(10)	2215.8(18)	4630.6(9)	50.7(6)
H13d	4494.2(14)	2670(3)	3670(7)	55.2(6)
H13e	4826(4)	3278(8)	4077(3)	55.2(6)
H13f	4909(3)	3550(6)	3309(4)	55.2(6)
H13g	6905(3)	2126(8)	3187(3)	48.1(6)
H13h	5745(7)	1920(6)	3190(3)	48.1(6)
H13i	6056(10)	2824(2)	2794.3(8)	48.1(6)
H137	9024.5(11)	6334.9(9)	1714.5(7)	26.5(3)
H138	9200.6(11)	5846.1(9)	2843.9(7)	26.1(3)
H14a	6589(2)	5484(4)	1493(5)	46.6(6)
H14b	7029(2)	6384(4)	1297(6)	46.6(6)
H14c	6793(4)	5935(8)	752.5(18)	46.6(6)
H14d	8561(8)	6511(3)	901(4)	45.7(5)
H14e	9357.8(16)	5699(4)	869(4)	45.7(5)
H14f	8452(7)	6009(7)	367.2(8)	45.7(5)
H14g	8754(3)	4374(4)	1149(6)	42.0(5)
H14h	7549(7)	4348(4)	1305(4)	42.0(5)

Atom	x	y	z	U_{eq}
H14i	7945(10)	4795.6(11)	597.7(14)	42.0(5)
H144	9035.3(12)	3238.3(10)	4354.0(7)	30.2(4)
H145	10502.8(13)	2419.6(10)	4544.3(7)	32.7(4)
H147	11473.3(12)	3412.3(11)	2830.2(8)	35.6(4)
H148	10003.2(11)	4235.5(10)	2643.5(7)	30.6(4)
H14j	12469(4)	2709(5)	4033(6)	56.5(7)
H14k	11942.3(16)	1858(4)	4115(6)	56.5(7)
H14l	12367(5)	2338(8)	3424.0(12)	56.5(7)
H151	9806.5(11)	4637.3(10)	4193.9(7)	28.8(4)
H152	9825.3(13)	5693.8(10)	4659.6(8)	33.9(4)
H154	7242.7(12)	6734.0(10)	3900.6(7)	29.7(4)
H155	7228.1(11)	5682.6(9)	3441.3(7)	26.7(3)
H15a	7901(5)	7012(6)	4824(6)	59.6(7)
H15b	9123(7)	6818(4)	4879(5)	59.6(7)
H15c	8632(12)	7462(2)	4251.1(11)	59.6(7)
H202	6150.0(11)	5793.4(9)	2420.7(7)	27.7(3)
H203	7295.2(11)	6840.6(9)	2071.9(7)	26.9(3)
H20a	4136(3)	6881(6)	3726(5)	40.6(5)
H20b	3745(6)	6993(5)	3016.4(13)	40.6(5)
H20c	3425(3)	6226.4(13)	3590(6)	40.6(5)
H20d	4351(11)	5912.7(16)	2496(3)	58.3(7)
H20e	5144(4)	5153(6)	2876(6)	58.3(7)
H20f	3981(8)	5267(7)	3137(4)	58.3(7)
H20g	5860(7)	5222(6)	3724.2(9)	54.7(6)
H20h	5493(10)	5957.8(11)	4080(3)	54.7(6)
H20i	4763(4)	5243(7)	4091(3)	54.7(6)
H209	8176.7(13)	9324.5(11)	3812.8(8)	38.7(5)
H210	8508.1(12)	8740.0(11)	2911.6(8)	34.9(4)
H21a	5104.8(14)	9660(7)	3954(3)	48.8(6)
H21b	5144.6(13)	8710.4(19)	4396(7)	48.8(6)
H21c	5106.2(14)	9489(8)	4720(4)	48.8(6)
H21d	6561(9)	8076(3)	4892(4)	59.9(7)
H21e	7545.8(15)	8576(7)	4840(3)	59.9(7)
H21f	6548(9)	8821(5)	5247.6(10)	59.9(7)
H21g	7452(2)	9931(2)	4335(8)	67.8(8)
H21h	6513(11)	10368(3)	3879(3)	67.8(8)
H21i	6362(10)	10209(5)	4641(5)	67.8(8)
H216	7278.7(12)	9663.5(10)	2120.4(8)	31.8(4)
H217	6629.5(12)	10659.1(10)	1302.9(8)	33.0(4)
H219	4647.8(13)	9215.9(10)	1105.4(8)	35.1(4)
H220	5309.8(11)	8208.0(10)	1916.0(7)	29.9(4)
H22a	5762(5)	10618(7)	255(2)	57.7(7)
H22b	4616(6)	10414(5)	476(4)	57.7(7)
H22c	5095(11)	11133(2)	707(2)	57.7(7)
H223	8549.0(11)	7273.3(9)	3040.6(7)	28.2(3)
H224	10067.9(11)	6939.5(10)	2612.0(8)	31.9(4)
H226	9129.4(12)	8200.8(11)	943.0(8)	34.5(4)
H227	7635.9(12)	8578.7(10)	1378.4(7)	31.0(4)
H22d	10540.9(18)	7279(10)	1053(3)	64.4(8)
H22e	11069(4)	7794(5)	1459(7)	64.4(8)
H22f	10955(5)	6809(5)	1753(4)	64.4(8)
H230	3508.4(11)	11269.2(10)	1258.9(7)	29.8(4)
H231	2363.4(11)	10326.9(10)	1108.0(7)	29.8(4)
H23a	4196.6(15)	11910(5)	1677(4)	47.4(6)
H23b	5183(8)	11739(6)	2115.1(10)	47.4(6)
H23c	5082(8)	11161.7(18)	1631(4)	47.4(6)
H23d	5287(7)	9987(5)	2524(2)	43.0(5)
H23e	5411(6)	10681.9(11)	2908(5)	43.0(5)
H23f	4575.1(15)	10030(6)	3147(3)	43.0(5)
H23g	3981(2)	11718(7)	2921(5)	50.4(6)

Atom	x	y	z	U_{eq}
H23h	3054(6)	11750(6)	2462.5(17)	50.4(6)
H23i	3237(8)	10985.4(11)	3092(4)	50.4(6)
H237	172.4(13)	7758.3(12)	3489.0(8)	39.3(5)
H238	359.2(12)	8491.2(11)	2393.4(8)	37.4(4)
H24a	1813(8)	6642(3)	4051(5)	59.6(7)
H24b	1517(11)	6678(3)	4775(2)	59.6(7)
H24c	663(3)	6967.7(12)	4238(7)	59.6(7)
H24d	3091.7(16)	7453(8)	4112(5)	50.4(6)
H24e	2785(4)	8323.2(12)	4318(6)	50.4(6)
H24f	2671(3)	7436(8)	4827(2)	50.4(6)
H24g	1032(8)	8023(5)	4963.4(9)	57.7(7)
H24h	1170(7)	8840(3)	4386(6)	57.7(7)
H24i	279.0(19)	8265(8)	4370(5)	57.7(7)
H244	3260.5(14)	9124.0(13)	1059.4(10)	44.7(5)
H245	4222.1(14)	8112.0(14)	724.9(10)	50.1(6)
H247	3090.8(19)	6440.0(13)	2086.1(11)	61.2(7)
H248	2217.3(16)	7456.3(12)	2465.5(9)	47.3(5)
H24j	4551(13)	6773(5)	749(3)	96.1(13)
H24k	4992(7)	6430(9)	1457(7)	96.1(13)
H24l	4000(6)	6075(5)	1274(9)	96.1(13)
H251	1539.7(11)	8908.2(10)	935.2(7)	28.4(3)
H252	114.2(11)	9443.5(10)	406.2(7)	31.3(4)
H254	-815.0(12)	10607.7(11)	1710.2(8)	36.1(4)
H255	611.1(12)	10079.6(11)	2234.2(8)	33.7(4)
H25a	-1240(4)	10330(9)	262(2)	61.0(7)
H25b	-1888(3)	10044(6)	919(4)	61.0(7)
H25c	-1507(6)	10966(3)	716(6)	61.0(7)

Table S28. Solvent masking (PLATON/SQUEEZE) information for **3-Br.**

No	x	y	z	V	e	Content
1	0.000	0.000	0.500	125.8	0.0	?
2	-0.500	0.500	0.172	847.0	170.4	4Et2O
3	0.279	0.965	0.015	13.1	0.0	?
4	0.444	0.111	0.392	19.6	0.0	?
5	0.556	0.889	0.608	19.6	0.0	?
6	0.721	0.035	-0.015	13.1	0.0	?

7.4 Single crystal structure analysis of 3-H

CCDC Deposition number 2503904

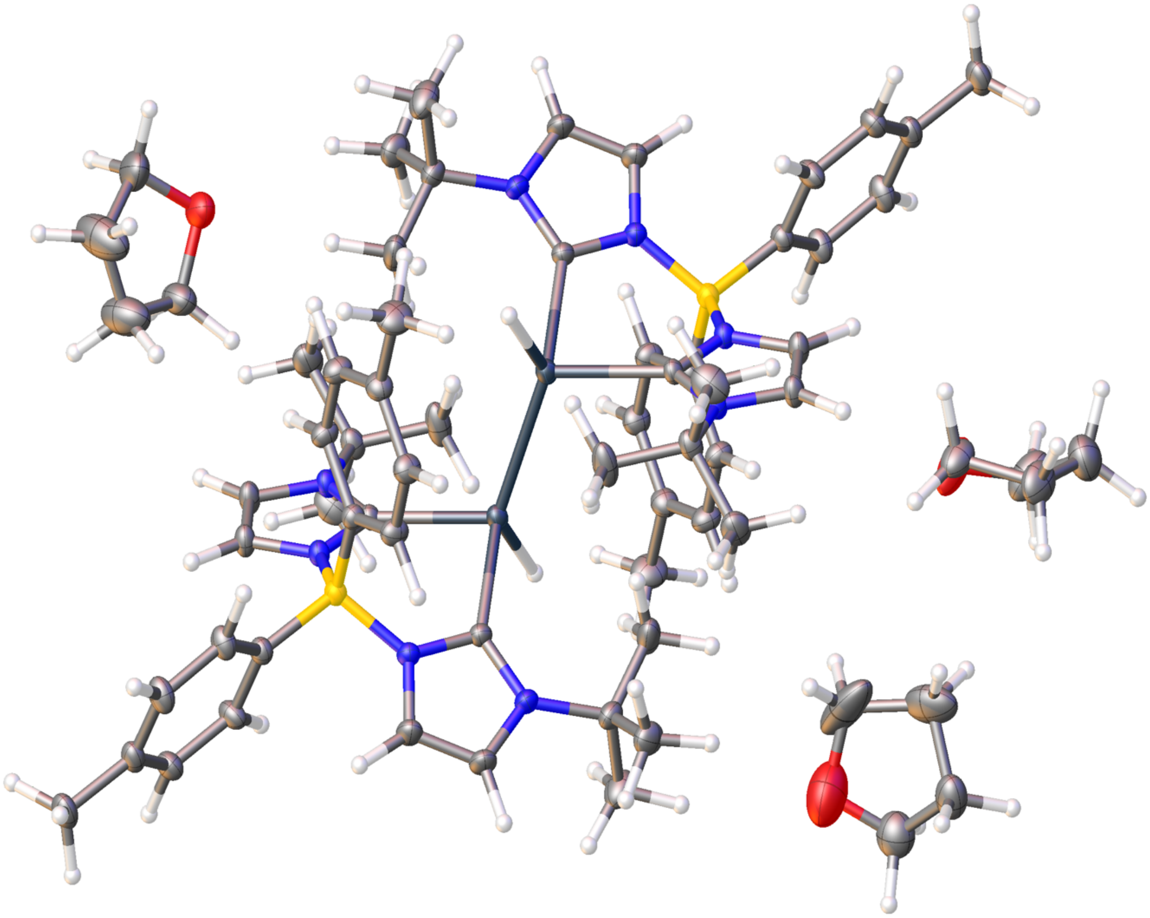

Figure S35. Olex-2 representation of the molecular structure of ligand **3-H**.

7.4.1 Experimental

Single colourless block-shaped crystals of **3-H** recrystallised from THF-d_8 at 238 K. A suitable crystal with dimensions 0.10 × 0.09 × 0.06 mm was selected and mounted on a MITIGEN holder in oil on a Rigaku FRE+ diffractometer with HF Varimax confocal mirrors, a UG2 goniometer and HyPix 6000HE detector. The crystal was kept at a steady T = 100(2) K during data collection. The structure was solved with the ShelXT 2018/2[30] solution program using dual methods and by using Olex2 1.5-dev[31] as the graphical interface. The model was refined with olex2.refine 1.5-dev[32] using full matrix least squares minimisation on $|F|^2$.

7.4.2 Crystal Data

$C_{76}H_{114}B_2In_2N_8O_5$, M_r = 1471.088, triclinic, *P*-1 (No. 2), a = 12.6418(1) Å, b = 12.9930(1) Å, c = 13.6004(1) Å, α = 66.891(1)°, β = 88.240(1)°, γ = 66.063(1)°, *V* = 1855.88(4) Å^3, *T* = 100(2) K, *Z* = 1, *Z'* = 0.5, μ(Mo K_α) = 0.675, 180923 reflections measured, 19455 unique (R_{int} = 0.0444) which were used in all calculations. The final wR_2 was 0.0824 (all data) and R_1 was 0.0322 (I≥2σ(I)).

**Compound**	**3-H**
Formula	$C_{76}H_{114}B_2In_2N_8O_5$
$D_{calc.}$/ g cm^{-3}	1.316
m/mm^{-1}	0.675
Formula Weight	1471.088
Colour	colourless
Shape	block-shaped
Size/mm	0.10×0.09×0.06
T/K	100(2)
Crystal System	triclinic
Space Group	*P*-1
a/Å	12.6418(1)
b/Å	12.9930(1)
c/Å	13.6004(1)
a/°	66.891(1)
b/°	88.240(1)
g/°	66.063(1)
V/Å^3	1855.88(4)
Z	1
Z'	0.5
Wavelength/Å	0.71073
Radiation type	Mo K_a
Q_{min}/°	1.96
Q_{max}/°	37.98
Index range h	-21 ≥ h ≥ 21
Index range k	-22 ≥ k ≥ 22
Index range l	-23 ≥ l ≥ 23
Measured Refl's.	180923
Indep't Refl's	19455
Refl's I≥2*s*(I)	17398
R_{int}	0.0444
Parameters	478
Restraints	46
Largest Peak/eÅ^3	2.3445
Deepest Hole/eÅ^3	-1.1590
GooF	1.0319
R_1 (I≥2*s*(I) / all)	0.0322 / 0.0392
wR_2 (I≥2*s*(I) / all)	0.0791 / 0.0824

7.4.3 Structure Quality Indicators

Reflections:	d min (MoKα) 2Θ=76.0° 0.58	I/σ(I) 40.0	Rint m=9.30 4.44%	Full 50.5° 96% to 76.0° 99.9
Refinement:	Shift -0.001	Max Peak 2.3	Min Peak -1.2	GooF 1.032

A colourless block-shaped crystal with dimensions 0.10 × 0.09 × 0.06 mm was mounted on a MITIGEN holder in oil. Data were collected using a Rigaku FRE+ diffractometer with HF Varimax confocal mirrors, a UG2 goniometer and HyPix 6000HE detector equipped with an Oxford Cryosystems low-temperature device operating at T = 100(2) K.

Data were measured using profile data from ω-scans with Mo K_α radiation. The diffraction pattern was indexed and the total number of runs and images was based on the strategy calculation from the program CrysAlis^{Pro} system (CCD 44.102a 64-bit (release 28-03-2025)). The maximum resolution achieved was Θ = 37.98° (0.58 Å). The unit cell was refined using CrysAlis^{Pro} on 81122 reflections, 45% of the observed reflections.

Data reduction, scaling and absorption corrections were performed using CrysAlis^{Pro}. The final completeness is 99.94 % out to 37.98° in Θ. An analytical absorption correction was performed using CrysAlis^{Pro} 1.171.44.122a (Rigaku Oxford Diffraction, 2025) Analytical numeric absorption correction using a multifaceted crystal model based on expressions derived by R.C. Clark & J.S. Reid.[34] Empirical absorption correction using spherical harmonics, implemented in SCALE3 ABSPACK scaling algorithm. The absorption coefficient μ of this material is 0.675 mm^{-1} at this wavelength (λ = 0.71073Å) and the minimum and maximum transmissions are 0.952 and 0.966. The structure was solved in the space group P-1 (# 2) by ShelXT 2018/2[30] using dual methods. It was refined by full matrix least squares minimisation on $|F|^2$ using version of olex2.refine 1.5-dev.[32] All non-hydrogen atoms were refined anisotropically. Most hydrogen atom positions were calculated geometrically and refined using the riding model, but some hydrogen atoms were refined freely (see below).

_refine_special_details: In refined with a 4th order anharmonic component. Hydride H-atom was located in the difference map of a refinement of low-resolution reflections only (>1.2A) and refined with the riding model. A standard distance restraint (DFIX) for the indium hydride (In1-H) distance of 1.618(2)A derived from the location in the low-resolution difference map was used.

_olex2_refine_details: Refinement using NoSpherA2, an implementation of NOn-SPHERical Atom-form-factors in Olex2.[33] 2021 NoSpherA2 implementation of HAR makes use of tailor-made aspherical atomic form factors calculated on-the-fly from a Hirshfeld-partitioned electron density (ED) - not from spherical-atom form factors. The ED is calculated from a gaussian basis set single determinant SCF wavefunction - either Hartree-Fock or DFT using selected functionals- for a fragment of the crystal. This fragment can be embedded in an electrostatic crystal field by employing cluster charges or modelled using implicit solvation models, depending on the software used. The following options were used: SOFTWARE: ORCA 6.1 ::: PARTITIONING: NoSpherA2 ::: INT ACCURACY: Low ::: METHOD: r2SCAN ::: BASIS SET: x2c-SVP ::: CHARGE: 0 ::: MULTIPLICITY: 2 ::: RELATIVISTIC: ZORA ::: DATE: 2025-10-02_14-08-02

_exptl_absorpt_process_details: CrysAlis^{Pro} 1.171.44.122a (Rigaku Oxford Diffraction, 2025) Analytical numeric absorption correction using a multifaceted crystal model based on expressions derived by R.C. Clark & J.S. Reid.[34] using spherical harmonics, implemented in SCALE3 ABSPACK scaling algorithm.

The value of Z' is 0.5. This means that only half of the formula unit is present in the asymmetric unit, with the other half consisting of symmetry equivalent atoms. The moiety formula is C56 H74 B2 In2 N8, 5(C4 H8 O).

7.4.4 Data Plots: Diffraction Data

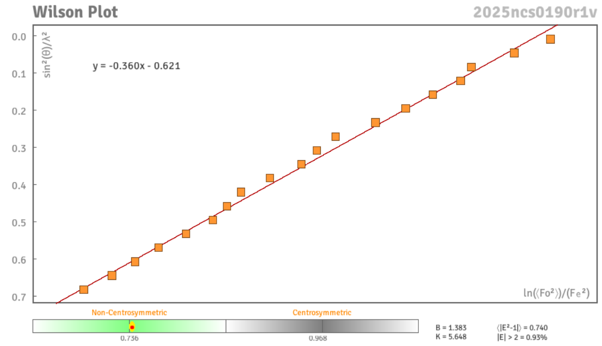

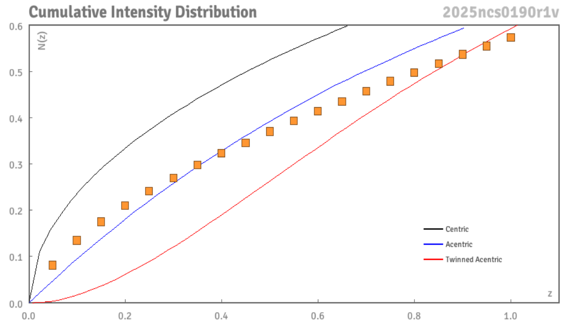

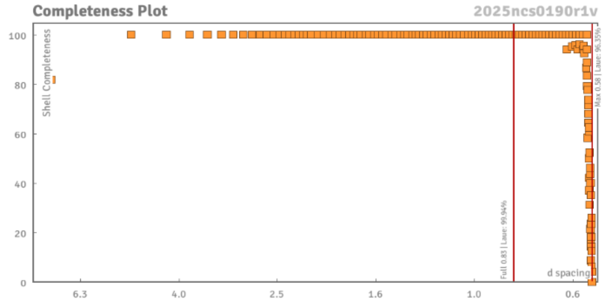

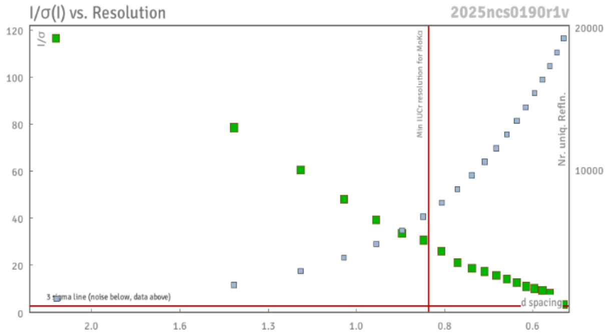

7.4.5 Data Plots: Refinement and Data

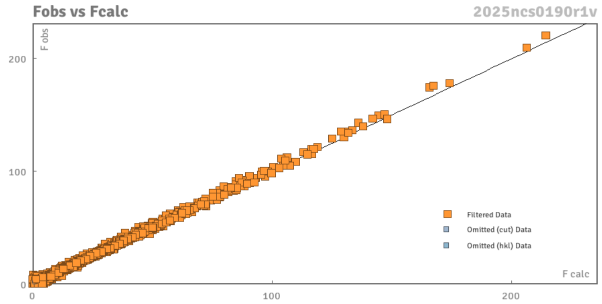

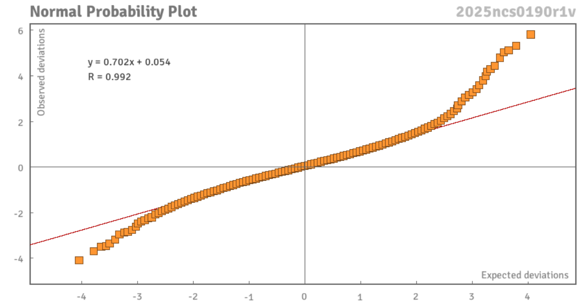

7.4.6 Reflection Statistics

Total reflections (after filtering)	180923	Unique reflections	19455
Completeness	0.963	Mean I/σ	29.91
hkl_{max} collected	(21, 22, 23)	hkl_{min} collected	(-21, -22, -23)
hkl_{max} used	(21, 22, 23)	hkl_{min} used	(-21, -20, 0)
Lim d_{max} collected	100.0	Lim d_{min} collected	0.36
d_{max} used	10.37	d_{min} used	0.58
Friedel pairs	18431	Friedel pairs merged	1
Inconsistent equivalents	0	R_{int}	0.0444
R_{sigma}	0.025	Intensity transformed	0
Omitted reflections	0	Omitted by user (OMIT hkl)	0
Multiplicity	(2773, 6168, 6595, 5839, 4426, 3103, 2398, 1988, 1584, 1127, 761, 482, 237, 152, 90, 45, 37, 18, 27, 8, 8, 3, 7, 4, 1, 2, 3)	Maximum multiplicity	46
Removed systematic absences	0	Filtered off (Shel/OMIT)	0

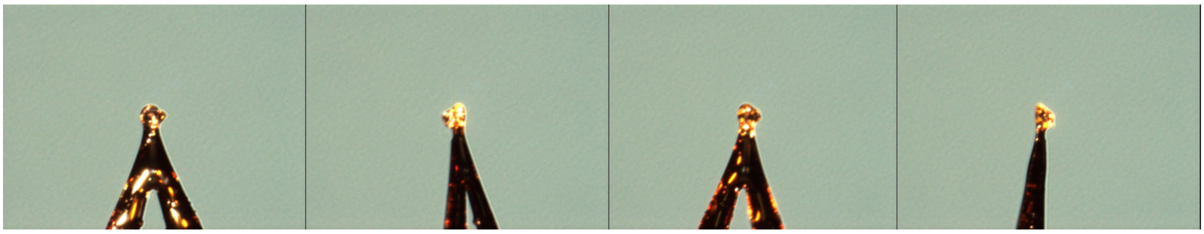

7.4.7 Fractional Atomic Coordinates

Table S29. Fractional Atomic Coordinates (×10^4) and Equivalent Isotropic Displacement Parameters (Å^2×10^3) for **3-H**. U_{eq} is defined as 1/3 of the trace of the orthogonalised U_{ij}.

Atom	x	y	z	U_{eq}
In1	5292.05(15)	4169.68(16)	4526.32(13)	19.03(8)
N1	7923.2(8)	3007.4(8)	4183.0(8)	19.21(15)
N2	6515.5(8)	4878.2(8)	2464.4(7)	17.53(14)
N3	7825.7(9)	1606.9(9)	5683.2(8)	21.80(17)
N4	4673.1(8)	5674.5(9)	1810.5(7)	18.97(15)
C1	7197.5(9)	2775.8(10)	4908.1(8)	18.81(17)
C2	8998.3(11)	1991.7(11)	4507.3(10)	25.8(2)
C3	8940.9(11)	1115.3(11)	5439.7(11)	27.4(2)
C4	5449.1(9)	4985.3(10)	2750.2(8)	16.90(16)
C5	6395.9(10)	5514.2(11)	1357.2(9)	22.59(19)
C6	5252.1(10)	6007.1(11)	945.0(9)	22.72(19)
C7	7459.9(12)	861.9(11)	6656.3(9)	24.5(2)
C8	6268.5(14)	1640.9(14)	6849.4(12)	37.0(3)
C9	8374.0(14)	361.3(14)	7639.2(11)	32.9(3)
C10	7413.0(17)	-212.7(15)	6480.3(13)	38.3(3)
C11	3402.7(10)	5983.2(12)	1623.9(9)	22.84(19)
C12	3265.2(14)	5253.8(18)	1026.9(15)	39.3(3)
C13	2744.3(13)	7373.1(14)	909.2(12)	34.4(3)
C14	2906.5(12)	5676.5(18)	2673.5(11)	37.5(3)
C15	8766.4(9)	4038.3(10)	2513.4(9)	20.09(17)
C16	8867.0(11)	3349.3(12)	1903.5(11)	25.2(2)
C17	9764.5(11)	3097.7(12)	1296.5(10)	25.9(2)
C18	10635.4(10)	3505.8(12)	1282.4(10)	25.4(2)
C19	10561.2(11)	4171.4(14)	1889.3(11)	28.8(2)
C20	9640.6(11)	4439.6(13)	2483.8(10)	25.3(2)
C21	11601.4(12)	3250.6(15)	614.7(12)	33.1(3)
C22	7679.6(9)	5234.5(10)	3757.9(8)	18.60(16)
C23	8412.9(11)	4805.7(11)	4718.2(9)	23.43(19)
C24	8439.8(11)	5597.8(12)	5160.0(10)	24.8(2)
C25	7724.5(11)	6864.9(12)	4661.1(10)	24.4(2)
C26	6993.3(12)	7308.1(11)	3700.4(11)	25.9(2)
C27	6974.7(10)	6510.4(11)	3259.8(9)	22.59(19)
C28	7734.8(16)	7715.2(15)	5151.9(14)	37.8(3)
B1	7712.4(10)	4304.3(11)	3230.0(9)	18.21(18)
O61	4396.4(16)	8000.3(15)	-1595.4(10)	57.4(4)
C61	4957(2)	8638(2)	-2343.5(17)	54.6(5)
C62	4608(2)	8722(2)	-3433.9(17)	54.1(5)
C63	3420.1(19)	8699.0(19)	-3325.3(13)	44.8(4)
C64	3567.3(18)	7865.1(15)	-2149.1(13)	39.4(3)
O71	532(2)	11470(2)	-666.4(17)	91.9(7)
C71	1090(2)	10431(2)	-965(3)	101.2(11)
C72	1194.6(19)	10932(2)	-2162(3)	67.6(7)
C73	450.0(16)	12289(2)	-2524.5(15)	47.3(4)
C74	560(2)	12467(2)	-1502.1(18)	53.1(5)
O51	6077(2)	-190(2)	9435(2)	41.8(5)
C51	5311(6)	854(5)	9648(6)	43.2(10)
C52	4202(5)	680(6)	9874(6)	66.4(16)
C53	4435(8)	-531(8)	10043(9)	78(3)
C54	5822(5)	-1161(4)	10106(4)	55.5(12)

7.4.8 Anisotropic Displacement and Structural Parameters

Table S30. Anisotropic Displacement Parameters (×10^4) for **3-H** The anisotropic displacement factor exponent takes the form: $-2\pi^2[h^2a^{*2} \times U_{11} + ... + 2hka^* \times b^* \times U_{12}]$

Atom	U_{11}	U_{22}	U_{33}	U_{23}	U_{13}	U_{12}
In1	18.35(11)	19.40(12)	15.17(11)	-5.33(8)	3.51(7)	-6.11(8)
N1	17.9(4)	16.1(3)	19.2(4)	-4.8(3)	3.9(3)	-5.8(3)
N2	17.4(3)	19.2(4)	15.1(3)	-7.7(3)	3.4(3)	-6.4(3)
N3	22.7(4)	15.7(4)	19.6(4)	-4.1(3)	3.7(3)	-4.5(3)
N4	18.1(4)	22.6(4)	14.6(3)	-7.6(3)	3.0(3)	-7.3(3)
C1	19.0(4)	15.7(4)	17.4(4)	-4.5(3)	3.3(3)	-5.9(3)
C2	19.9(4)	19.9(4)	26.9(5)	-2.7(4)	5.4(4)	-5.6(4)
C3	23.2(5)	17.8(4)	27.3(5)	-0.8(4)	4.0(4)	-4.1(4)
C4	16.9(4)	19.3(4)	13.2(3)	-6.8(3)	3.4(3)	-6.6(3)
C5	20.3(4)	28.2(5)	15.9(4)	-10.9(4)	4.5(3)	-5.5(4)
C6	20.6(4)	28.8(5)	14.1(4)	-9.5(4)	3.2(3)	-5.7(4)
C7	29.7(5)	18.8(4)	18.9(4)	-8.2(4)	3.1(4)	-4.0(3)
C8	32.3(6)	32.2(6)	25.0(6)	-6.1(5)	9.8(5)	0.4(5)
C9	37.5(7)	29.2(6)	21.4(5)	-8.4(5)	-1.1(5)	-6.1(4)
C10	57.2(10)	31.4(7)	31.0(6)	-26.7(7)	7.2(6)	-9.7(5)
C11	18.0(4)	30.1(5)	18.4(4)	-8.3(4)	2.9(3)	-10.1(4)
C12	32.0(7)	50.6(9)	46.1(8)	-20.0(7)	4.4(6)	-28.1(7)
C13	25.7(6)	34.8(7)	26.7(6)	-2.7(5)	0.9(5)	-7.9(5)
C14	20.8(5)	57.3(9)	23.0(5)	-12.6(6)	5.5(4)	-10.3(6)
C15	18.0(4)	22.2(4)	19.7(4)	-8.8(3)	5.3(3)	-8.5(3)
C16	23.0(5)	28.6(5)	31.0(5)	-12.8(4)	11.8(4)	-17.7(4)
C17	22.5(5)	27.2(5)	27.6(5)	-8.8(4)	9.3(4)	-13.3(4)
C18	19.5(4)	28.7(5)	21.7(5)	-7.3(4)	6.7(4)	-7.9(4)
C19	22.7(5)	41.2(7)	27.8(5)	-17.2(5)	9.9(4)	-16.2(5)
C20	22.0(5)	34.8(6)	24.7(5)	-15.9(4)	7.7(4)	-14.3(4)
C21	23.7(5)	38.9(7)	28.7(6)	-8.9(5)	11.6(5)	-11.5(5)
C22	18.2(4)	18.4(4)	17.4(4)	-6.9(3)	2.2(3)	-6.5(3)
C23	23.7(5)	21.5(5)	20.8(4)	-6.3(4)	-1.5(4)	-7.8(4)
C24	26.8(5)	25.9(5)	21.9(5)	-10.8(4)	0.9(4)	-10.4(4)
C25	27.1(5)	24.7(5)	25.9(5)	-13.0(4)	5.6(4)	-12.9(4)
C26	28.7(5)	20.1(5)	27.4(5)	-9.6(4)	1.7(4)	-9.2(4)
C27	24.1(5)	18.3(4)	21.8(4)	-7.6(4)	-0.2(4)	-6.1(4)
C28	48.4(9)	34.2(7)	39.5(7)	-19.4(6)	3.6(6)	-21.7(6)
B1	16.9(4)	18.9(4)	17.5(4)	-6.9(4)	3.5(3)	-7.0(4)
O61	94.4(12)	59.4(9)	24.5(5)	-52.5(9)	-9.7(6)	-1.6(5)
C61	61.1(12)	47.6(10)	47.3(10)	-31.7(10)	-10.3(9)	-2.4(8)
C62	57.5(12)	61.7(13)	41.2(9)	-24.1(10)	17.7(9)	-21.6(9)
C63	60.0(11)	47.8(9)	25.5(6)	-23.4(8)	-1.4(7)	-13.2(6)
C64	58.6(10)	32.0(7)	30.7(6)	-23.3(7)	1.2(6)	-11.4(5)
O71	118.1(19)	94.2(15)	56.3(11)	-55.9(14)	15.5(11)	-13.6(10)
C71	47.3(13)	46.4(12)	127(2)	-5.0(9)	6.3(14)	28.3(11)
C72	36.2(9)	54.5(12)	111(2)	-14.2(8)	21.0(11)	-40.0(12)
C73	34.6(8)	54.1(10)	38.2(8)	-14.7(7)	8.9(6)	-9.6(7)
C74	48.8(10)	59.1(12)	51.3(11)	-25.8(9)	9.3(8)	-20.1(9)
O51	41.3(12)	34.1(10)	43.1(12)	-16.0(9)	19.0(10)	-10.6(9)
C51	42(2)	33.5(18)	54(2)	-14.7(15)	21.3(17)	-20.7(15)
C52	50(2)	53(3)	104(4)	-22.2(19)	29(2)	-41(3)
C53	66(5)	82(5)	129(8)	-46(3)	54(4)	-71(4)
C54	59(3)	40(2)	52(2)	-19(2)	28(2)	-8.5(18)

Table S31. Bond Lengths in Å for **3-H**.

Atom	Atom	Length/Å
In1	In1[1]	2.7734(3)
In1	C1	2.2695(11)
In1	C4	2.2648(10)
N1	C1	1.3566(14)
N1	C2	1.3812(15)
N1	B1	1.5915(15)
N2	C4	1.3587(13)
N2	C5	1.3817(14)
N2	B1	1.5815(15)
N3	C1	1.3655(14)
N3	C3	1.3827(16)
N3	C7	1.4908(15)
N4	C4	1.3651(13)
N4	C6	1.3829(14)
N4	C11	1.4923(15)
C2	C3	1.3556(17)
C5	C6	1.3535(16)
C7	C8	1.5196(19)
C7	C9	1.5323(19)
C7	C10	1.530(2)
C11	C12	1.528(2)
C11	C13	1.5333(19)
C11	C14	1.5137(17)
C15	C16	1.4105(17)
C15	C20	1.3941(16)
C15	B1	1.6296(16)
C16	C17	1.3889(16)
C17	C18	1.3993(18)
C18	C19	1.388(2)
C18	C21	1.5052(17)
C19	C20	1.4013(17)
C22	C23	1.3999(16)
C22	C27	1.3994(16)
C22	B1	1.6175(16)
C23	C24	1.3915(17)
C24	C25	1.3934(18)
C25	C26	1.3945(18)
C25	C28	1.5025(19)
C26	C27	1.3937(17)
O61	C61	1.413(2)
O61	C64	1.414(2)
C61	C62	1.510(3)
C62	C63	1.516(3)
C63	C64	1.504(2)
O71	C71	1.458(5)
O71	C74	1.360(3)
C71	C72	1.522(5)
C72	C73	1.496(3)
C73	C74	1.515(3)
O51	C51	1.444(7)
O51	C54	1.392(5)
C51	C52	1.513(10)
C52	C53	1.399(10)
C53	C54	1.593(10)

[1]1-x,1-y,1-z

Table S32. Bond Angles in ° for **3-H**.

Atom	Atom	Atom	Angle/°
C4	In1	C1	90.54(4)
C2	N1	C1	109.44(9)
B1	N1	C1	127.08(9)
B1	N1	C2	122.48(9)
C5	N2	C4	109.37(9)
B1	N2	C4	128.15(8)
B1	N2	C5	121.95(9)
C3	N3	C1	109.71(9)
C7	N3	C1	129.65(10)
C7	N3	C3	120.62(10)
C6	N4	C4	109.94(9)
C11	N4	C4	129.51(9)
C11	N4	C6	120.32(9)
N1	C1	In1	118.67(7)
N3	C1	In1	134.57(8)
N3	C1	N1	106.25(9)
C3	C2	N1	107.80(10)
C2	C3	N3	106.80(10)
N2	C4	In1	119.08(7)
N4	C4	In1	134.74(7)
N4	C4	N2	106.06(8)
C6	C5	N2	107.97(9)
C5	C6	N4	106.65(9)
C8	C7	N3	111.41(10)
C9	C7	N3	108.23(11)
C9	C7	C8	109.16(11)
C10	C7	N3	108.25(10)
C10	C7	C8	109.77(13)
C10	C7	C9	110.00(11)
C12	C11	N4	107.94(10)
C13	C11	N4	108.27(10)
C13	C11	C12	109.26(12)
C14	C11	N4	111.88(10)
C14	C11	C12	110.21(13)
C14	C11	C13	109.22(11)
C20	C15	C16	115.65(10)
B1	C15	C16	121.55(10)
B1	C15	C20	122.78(10)
C17	C16	C15	122.65(11)
C18	C17	C16	120.69(12)
C19	C18	C17	117.55(11)
C21	C18	C17	120.94(13)
C21	C18	C19	121.50(12)
C20	C19	C18	121.33(12)

Atom	Atom	Atom	Angle/°
C19	C20	C15	122.11(12)
C27	C22	C23	116.25(10)
B1	C22	C23	121.15(10)
B1	C22	C27	122.50(10)
C24	C23	C22	122.17(11)
C25	C24	C23	120.97(11)
C26	C25	C24	117.58(11)
C28	C25	C24	120.92(12)
C28	C25	C26	121.49(12)
C27	C26	C25	121.14(11)
C26	C27	C22	121.88(11)
N2	B1	N1	110.83(9)
C15	B1	N1	107.04(9)
C15	B1	N2	107.73(8)
C22	B1	N1	108.43(9)
C22	B1	N2	109.50(9)

Atom	Atom	Atom	Angle/°
C22	B1	C15	113.30(9)
C64	O61	C61	110.03(13)
C62	C61	O61	107.03(16)
C63	C62	C61	101.85(16)
C64	C63	C62	102.48(16)
C63	C64	O61	106.61(14)
C74	O71	C71	105.8(2)
C72	C71	O71	108.70(19)
C73	C72	C71	101.8(2)
C74	C73	C72	102.1(2)
C73	C74	O71	106.9(2)
C54	O51	C51	104.6(4)
C52	C51	O51	105.2(5)
C53	C52	C51	108.3(6)
C54	C53	C52	102.9(7)
C53	C54	O51	103.9(5)

Table S33. Torsion Angles in ° for **3-H**.

Atom	Atom	Atom	Atom	Angle/°
In1	C1	N1	C2	-173.28(9)
In1	C1	N1	B1	18.04(11)
In1	C1	N3	C3	171.45(12)
In1	C1	N3	C7	-6.80(14)
In1	C4	N2	C5	-175.64(8)
In1	C4	N2	B1	-3.95(11)
In1	C4	N4	C6	175.12(11)
In1	C4	N4	C11	-10.50(13)
N1	C1	N3	C3	0.12(11)
N1	C1	N3	C7	-178.13(9)
N1	C2	C3	N3	-0.29(12)
N1	B1	N2	C4	46.94(11)
N1	B1	N2	C5	-142.31(9)
N1	B1	C15	C16	66.84(11)
N1	B1	C15	C20	-111.30(10)
N1	B1	C22	C23	36.76(11)
N1	B1	C22	C27	-146.86(9)
N2	C4	N4	C6	-0.70(10)
N2	C4	N4	C11	173.67(8)
N2	C5	C6	N4	0.43(11)
N2	B1	N1	C1	-55.80(11)
N2	B1	N1	C2	136.86(10)
N2	B1	C15	C16	-52.40(11)
N2	B1	C15	C20	129.45(10)
N2	B1	C22	C23	157.79(9)
N2	B1	C22	C27	-25.82(11)
N3	C1	N1	C2	-0.30(11)
N3	C1	N1	B1	-168.99(8)
N4	C4	N2	C5	0.97(10)
N4	C4	N2	B1	172.65(8)
C1	N1	C2	C3	0.38(11)
C1	N1	B1	C15	-173.03(12)
C1	N1	B1	C22	64.41(12)
C1	N3	C3	C2	0.11(11)
C1	N3	C7	C8	-9.78(15)
C1	N3	C7	C9	-129.80(14)
C1	N3	C7	C10	111.01(15)
C2	N1	B1	C15	19.64(12)

Atom	Atom	Atom	Atom	Angle/°
C2	N1	B1	C22	-102.92(11)
C2	C3	N3	C7	178.55(10)
C3	N3	C7	C8	172.13(14)
C3	N3	C7	C9	52.11(13)
C3	N3	C7	C10	-67.08(14)
C3	C2	N1	B1	169.68(10)
C4	N2	C5	C6	-0.89(10)
C4	N2	B1	C15	163.74(12)
C4	N2	B1	C22	-72.63(11)
C4	N4	C6	C5	0.17(10)
C4	N4	C11	C12	-108.53(14)
C4	N4	C11	C13	133.30(13)
C4	N4	C11	C14	12.90(14)
C5	N2	B1	C15	-25.51(11)
C5	N2	B1	C22	98.11(11)
C5	C6	N4	C11	-174.81(10)
C6	N4	C11	C12	65.35(13)
C6	N4	C11	C13	-52.82(12)
C6	N4	C11	C14	-173.23(13)
C6	C5	N2	B1	-173.19(9)
C15	C16	C17	C18	1.28(16)
C15	C20	C19	C18	1.11(15)
C15	B1	C22	C23	-81.92(11)
C15	B1	C22	C27	94.47(10)
C16	C15	C20	C19	-0.21(14)
C16	C15	B1	C22	-173.69(11)
C16	C17	C18	C19	-0.36(15)
C16	C17	C18	C21	-179.19(12)
C17	C16	C15	C20	-0.96(15)
C17	C16	C15	B1	-179.23(12)
C17	C18	C19	C20	-0.80(14)
C19	C20	C15	B1	178.04(12)
C20	C15	B1	C22	8.17(12)
C20	C19	C18	C21	178.03(13)
C22	C23	C24	C25	0.34(14)
C22	C27	C26	C25	0.27(14)
C23	C22	C27	C26	-0.60(13)
C23	C24	C25	C26	-0.67(15)
C23	C24	C25	C28	178.63(13)
C24	C23	C22	C27	0.29(14)
C24	C23	C22	B1	176.90(11)
C24	C25	C26	C27	0.37(14)
C26	C27	C22	B1	-177.15(11)
C27	C26	C25	C28	-178.92(13)
O61	C61	C62	C63	27.8(2)
O61	C64	C63	C62	29.27(18)
C61	O61	C64	C63	-12.3(2)
C61	C62	C63	C64	-33.86(18)
C62	C61	O61	C64	-10.1(2)
O71	C71	C72	C73	-10.9(3)
O71	C74	C73	C72	-39.9(2)
C71	O71	C74	C73	32.9(2)
C71	C72	C73	C74	28.6(2)
C72	C71	O71	C74	-13.7(3)
O51	C51	C52	C53	13.8(5)
O51	C54	C53	C52	-30.5(5)
C51	O51	C54	C53	39.4(7)
C51	C52	C53	C54	9.3(5)
C52	C51	O51	C54	-34.1(6)

Table S34. Hydrogen Fractional Atomic Coordinates (×10^4) and Equivalent Isotropic Displacement Parameters (Å^2×10^3) for **3-H**. U_{eq} is defined as 1/3 of the trace of the orthogonalised U_{ij}.

Atom	x	y	z	U_{eq}
H	4692(16)	3167(16)	4536(15)	22.83(10)
H2	9766.4(11)	1908.5(11)	4083.4(10)	31.0(3)
H3	9647.7(11)	188.5(11)	5912.9(11)	32.9(3)
H5	7107.4(10)	5604.4(11)	891.3(9)	27.1(2)
H6	4858.3(10)	6565.4(11)	85.9(9)	27.3(2)
H8a	6068(6)	1093(5)	7615(6)	55.6(5)
H8b	6280(4)	2464(7)	6888(12)	55.6(5)
H8c	5597(2)	1928(11)	6184(6)	55.6(5)
H9a	8081(6)	-117(11)	8376.1(16)	49.4(4)
H9b	9214(3)	-289(10)	7547(5)	49.4(4)
H9c	8482(9)	1132.5(19)	7698(6)	49.4(4)
H10a	8259(4)	-739(8)	6293(12)	57.5(5)
H10b	7225(13)	-823(8)	7217(4)	57.5(5)
H10c	6721(9)	152.6(16)	5805(8)	57.5(5)
H12a	3673(12)	5444(11)	292(7)	58.9(5)
H12b	3693(11)	4260.3(19)	1554(5)	58.9(5)
H12c	2331.4(15)	5534(10)	807(11)	58.9(5)
H13a	2917(10)	7898.3(15)	1298(5)	51.5(4)
H13b	3043(9)	7585(3)	118(4)	51.5(4)
H13c	1801.2(16)	7631(3)	801(9)	51.5(4)
H14a	3329(9)	4673(2)	3161(5)	56.2(5)
H14b	3071(11)	6150(11)	3123(6)	56.2(5)
H14c	1960(2)	5984(12)	2496.7(12)	56.2(5)
H16	8206.2(11)	2996.0(12)	1908.9(11)	30.3(3)
H17	9791.1(11)	2570.8(12)	820.6(10)	31.0(3)
H19	11240.6(11)	4495.7(14)	1903.8(11)	34.5(3)
H20	9606.7(11)	4985.6(13)	2942.9(10)	30.3(3)
H21a	11296(4)	3958(7)	-220(3)	49.7(4)
H21b	12363(4)	3274(13)	955(7)	49.7(4)
H21c	11850(8)	2334(6)	623(9)	49.7(4)
H23	8988.2(11)	3807.2(11)	5138.5(9)	28.1(2)
H24	9036.2(11)	5216.9(12)	5916.3(10)	29.7(2)
H26	6419.7(12)	8307.4(11)	3282.1(11)	31.0(3)
H27	6387.6(10)	6895.8(11)	2496.6(9)	27.1(2)
H28a	8297(10)	8165(10)	4755(8)	56.7(5)
H28b	6841(2)	8423(8)	5040(10)	56.7(5)
H28c	8074(12)	7179(3)	6019(3)	56.7(5)
H61a	4677(2)	9579(2)	-2373.1(17)	65.5(6)
H61b	5923(2)	8128(2)	-2104.0(17)	65.5(6)
H62a	4542(2)	9595(2)	-4098.9(17)	65.0(6)
H62b	5238(2)	7921(2)	-3578.6(17)	65.0(6)
H63a	2714.4(19)	9640.8(19)	-3526.8(13)	53.8(5)
H63b	3219.2(19)	8311.9(19)	-3854.4(13)	53.8(5)
H64a	3885.4(18)	6887.6(15)	-2035.8(13)	47.3(4)
H64b	2719.2(18)	8137.4(15)	-1838.9(13)	47.3(4)
H71b	1973(2)	9803(2)	-476(3)	121.5(14)
H71a	558(2)	9896(2)	-805(3)	121.5(14)
H72b	2116.9(19)	10739(2)	-2285(3)	81.2(8)
H72a	849.8(19)	10535(2)	-2593(3)	81.2(8)
H73b	789.6(16)	12847(2)	-3185.1(15)	56.7(5)
H73a	-473.7(16)	12541(2)	-2803.1(15)	56.7(5)
H74a	-175(2)	13334(2)	-1550.4(18)	63.8(6)
H74b	1398(2)	12525(2)	-1387.9(18)	63.8(6)
H51a	5714(6)	862(5)	10358(6)	51.8(12)
H51b	5124(6)	1738(5)	8936(6)	51.8(12)
H52a	3913(5)	839(6)	10602(6)	79.7(19)
H52b	3492(5)	1353(6)	9176(6)	79.7(19)
H53a	4160(8)	-1005(8)	10809(9)	94(3)

Atom	x	y	z	U_{eq}
H53b	3985(8)	-542(8)	9362(9)	94(3)
H54a	6093(5)	-1901(4)	9808(4)	66.5(15)
H54b	6269(5)	-1566(4)	10948(4)	66.5(15)

Table S35. Atomic Occupancies for all atoms that are not fully occupied in **3-H**.

Atom	Occupancy
O51	0.5
C51	0.5
H51a	0.5
H51b	0.5
C52	0.5
H52a	0.5
H52b	0.5
C53	0.5
H53a	0.5
H53b	0.5
C54	0.5
H54a	0.5
H54b	0.5

7.5 Single crystal structure analysis of 3-F

CCDC Deposition Number 2503903

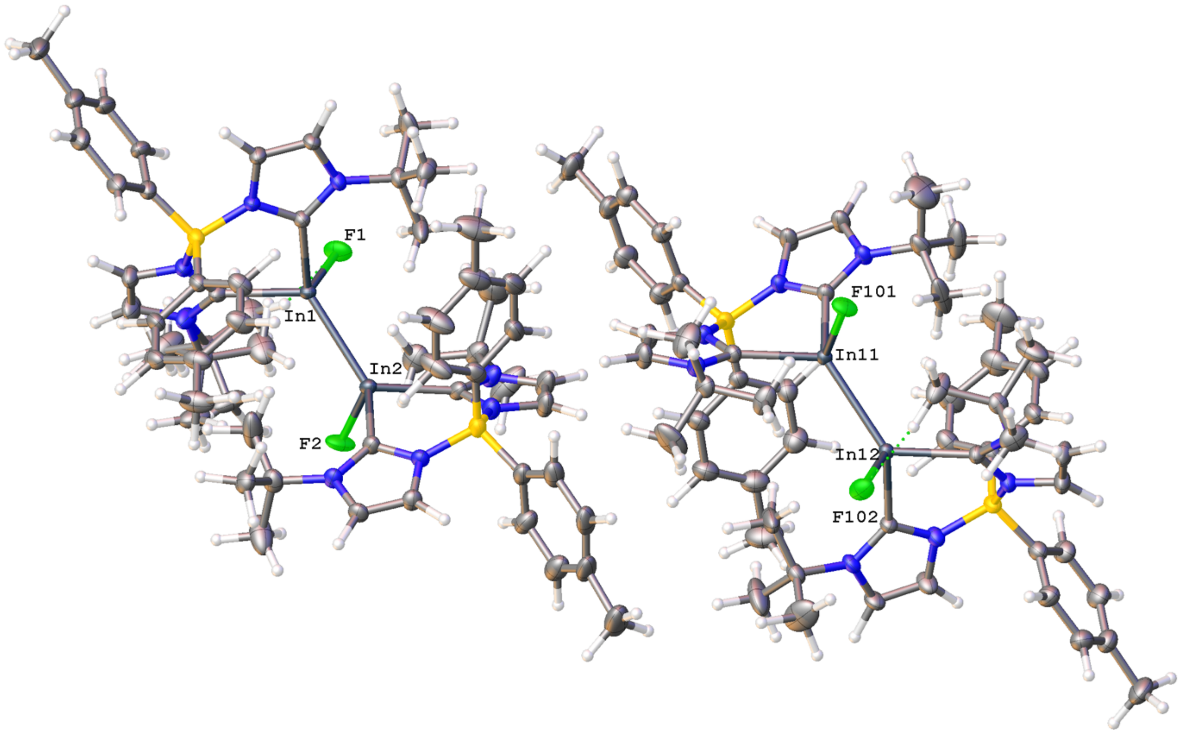

Figure S36. Olex-2 representation of the molecular structure of ligand **3-F**.

7.5.1 Experimental

Single colourless block-shaped crystals of **3-F** were recrystallised by slow diffusion of pentane over a saturated C_6D_6 solution. A suitable crystal with dimensions 0.11 × 0.09 × 0.07 mm was selected and mounted on a MITIGEN holder in oil on a Rigaku FRE+ diffractometer with HF Varimax confocal mirrors, a UG2 goniometer and HyPix 6000HE detector. The crystal was kept at a steady T = 100(2) K during data collection. The structure was solved with the ShelXT[30] solution program using dual methods and by using Olex2 1.5-dev[31] as the graphical interface. The model was refined with olex2.refine 1.5-dev[32] using full matrix least squares minimisation on $|F|^2$.

7.5.2 Crystal Data

$C_{56}H_{72}B_2F_2In_2N_8$, M_r = 1146.531, triclinic, *P*-1 (No. 2), a = 10.7628(1) Å, b = 20.1565(2) Å, c = 27.6818(2) Å, α = 108.512(1)°, β = 96.194(1)°, γ = 97.825(1)°, *V* = 5568.07(10) Å^3, *T* = 100(2) K, *Z* = 4, *Z'* = 2, μ(Mo K_α) = 0.877, 86939 reflections measured, 86939 unique (R_{int} = .) which were used in all calculations. The final wR_2 was 0.1209 (all data) and R_1 was 0.0471 (I≥2σ(I)).

**Compound**	**3-F**
Formula	$C_{56}H_{72}B_2F_2In_2N_8$
$D_{calc.}$/ g cm^{-3}	1.368
μ/mm^{-1}	0.877
Formula Weight	1146.531
Colour	colourless
Shape	block-shaped
Size/mm	0.11×0.09×0.07
T/K	100(2)
Crystal System	triclinic
Space Group	*P*-1
a/Å	10.7628(1)
b/Å	20.1565(2)
c/Å	27.6818(2)
α/°	108.512(1)
β/°	96.194(1)
γ/°	97.825(1)
V/Å^3	5568.07(10)
Z	4
Z'	2
Wavelength/Å	0.71073
Radiation type	Mo K_α
Θ_{min}/°	2.04
Θ_{max}/°	38.15
Index range h	-18 ≥ h ≥ 18
Index range k	-34 ≥ k ≥ 34
Index range l	-47 ≥ l ≥ 47
Measured Refl's.	86939
Indep't Refl's	86939
Refl's I≥2σ(I)	51070
R_{int}	.
Parameters	1334
Restraints	83
Largest Peak/eÅ^3	4.3655
Deepest Hole/eÅ^3	-2.3948
GooF	0.9827
R_1 (I≥2σ(I) / all)	0.0471 / 0.0966
wR_2 (I≥2σ(I) / all)	0.1061 / 0.1209

7.5.3 Structure Quality Indicators

Reflections:	d min (MoKα) 2Θ=76.3° 0.58	I/σ(I) 8.8	Rint m=1.98 n/a	Full 50.5° 96% to 76.3° 100
Refinement:	Shift 0.001	Max Peak 4.4	Min Peak -2.4	GooF 0.983

A colourless block-shaped crystal with dimensions 0.11 × 0.09 × 0.07 mm was mounted on a MITIGEN holder in oil. Data were collected using a Rigaku FRE+ diffractometer with HF Varimax confocal mirrors, a UG2 goniometer and HyPix 6000HE detector equipped with an Oxford Cryosystems low-temperature device operating at T = 100(2) K.

Data were measured using profile data from ω-scans with Mo K_α radiation. The diffraction pattern was indexed and the total number of runs and images was based on the strategy calculation from the program CrysAlis^{Pro} system (CCD 44.127a 64-bit (release 27-10-2025)). The maximum resolution achieved was Θ = 38.15° (0.58 Å).

The unit cell was refined using CrysAlis^{Pro} on 71363 reflections, 82% of the observed reflections.

Data reduction, scaling and absorption corrections were performed using CrysAlis^{Pro}. The final completeness is 99.92 % out to 38.15° in Θ. An analytical absorption correction was performed using CrysAlis^{Pro} 1.171.44.128a (Rigaku Oxford Diffraction, 2025) Analytical numeric absorption correction using a multifaceted crystal model based on expressions derived by R.C. Clark & J.S. Reid.[34] Empirical absorption correction using spherical harmonics, implemented in SCALE3 ABSPACK scaling algorithm. The absorption coefficient μ of this material is 0.877 mm^{-1} at this wavelength (λ = 0.71073Å) and the minimum and maximum transmissions are 0.930 and 0.952.

The structure was solved in the space group P-1 (# 2) by ShelXT using dual methods.[30] It was refined by full matrix least squares minimisation on $|F|^2$ using version of olex2.refine 1.5-dev.[32] All non-hydrogen atoms were refined anisotropically.

Hydrogen atom positions were calculated geometrically and refined using the riding model.

_refine_special_details: Structure refined as a 2-component non-merohedral twin (BASF 0.4964(3)). One tBu group is disordered over two positions (ca.73:27). Standard 1,2 and 1,3 equal distance geometric restraints (SADI) have been applied between all chemically equivalent atom pairs of each disorder component. Standard thermal restraints (RIGU) have been applied to all disordered atoms.

_olex2_refine_details: Refinement using NoSpherA2, an implementation of NOn-SPHERical Atom-form-factors in Olex2.[33] 2021 NoSpherA2 implementation of HAR makes use of tailor-made aspherical atomic form factors calculated on-the-fly from a Hirshfeld-partitioned electron density (ED) - not from spherical-atom form factors. The ED is calculated from a gaussian basis set single determinant SC wavefunction - either Hartree-Fock or DFT using selected functionals- for a fragment of the crystal. This fragment can be embedded in an electrostatic crystal field by employing cluster charges or modelled using implicit solvation models, depending on the software used. The following options were used: SOFTWARE: ORCA 6.1 ::: PARTITIONING: NoSpherA2 ::: INT ACCURACY: Normal ::: METHOD: r2SCAN ::: BASIS SET: jorge-DZP-DKH ::: CHARGE: 0 ::: MULTIPLICITY: 1 ::: RELATIVISTIC: ZORA ::: DATE: 2025-11-12_16-37-15

_exptl_absorpt_process_details: CrysAlis^{Pro} 1.171.44.128a (Rigaku Oxford Diffraction, 2025) Analytical numeric absorption correction using a multifaceted crystal model based on expressions derived by R.C. Clark & J.S. Reid using spherical harmonics, implemented in SCALE3 ABSPACK scaling algorithm.[34]

_twin_special_details: Component 2 rotated by 179.9973° around [-0.30 0.90 -0.30] (reciprocal) or [-0.71 0.71 0.00] (direct).

The value of Z' is 2. This means that there are two independent molecules in the asymmetric unit. The moiety formula is C56 H72 B2 F2 In2 N8.

7.5.4 Data Plots: Diffraction Data

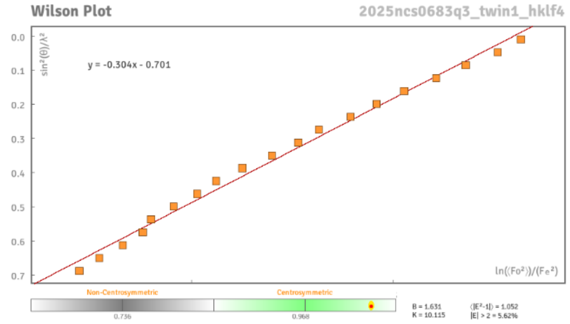

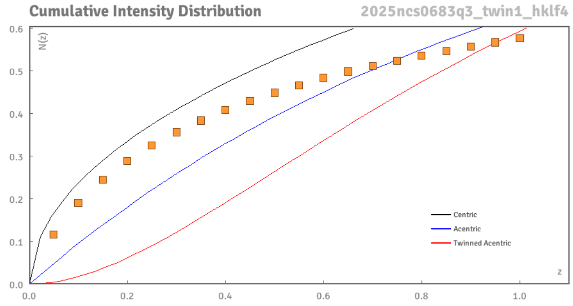

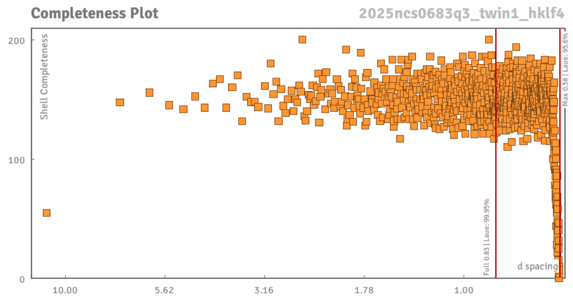

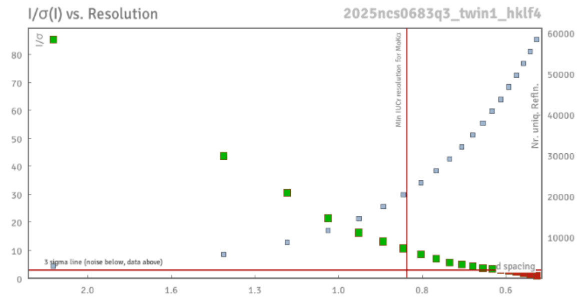

7.5.5 Data Plots: Refinement and Data

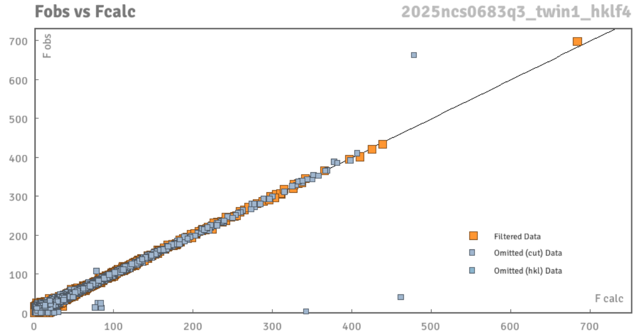

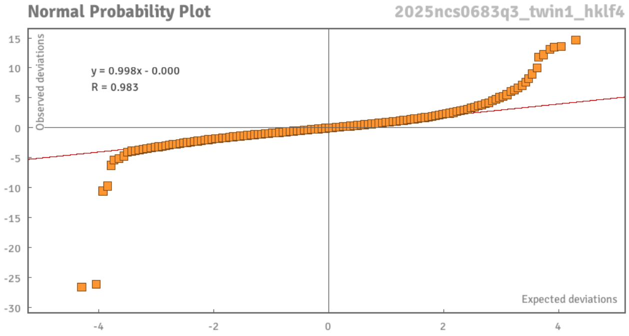

7.5.6 Reflection Statistics

Total reflections (after filtering)	115926	Unique reflections	58549
Completeness	0.956	Mean I/σ	14.72
hkl_{max} collected	(18, 34, 47)	hkl_{min} collected	(-18, -34, -47)
hkl_{max} used	(18, 32, 47)	hkl_{min} used	(-18, -34, 0)
Lim d_{max} collected	100.0	Lim d_{min} collected	0.36
d_{max} used	13.24	d_{min} used	0.58
Friedel pairs	15769	Friedel pairs merged	1
Inconsistent equivalents	0	R_{int}	0.0
R_{sigma}	0.1132	Intensity transformed	0
Omitted reflections	0	Omitted by user (OMIT hkl)	6
Multiplicity	(61703, 12623)	Maximum multiplicity	2
Removed systematic absences	0	Filtered off (Shel/OMIT)	0

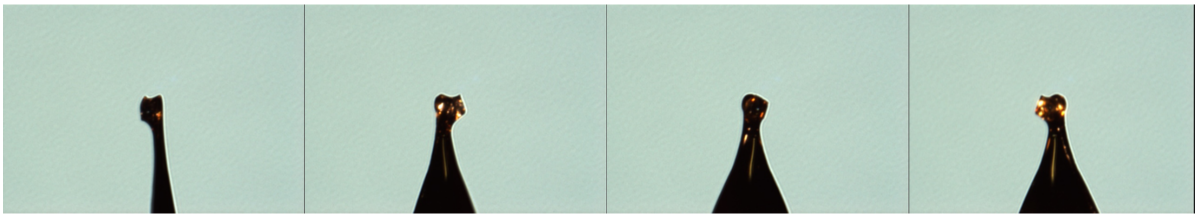

7.5.7 Fractional Atomic Coordinates

Table S36. Fractional Atomic Coordinates (×10^4) and Equivalent Isotropic Displacement Parameters (Å^2×10^3) for **3-F**. U_{eq} is defined as 1/3 of the trace of the orthogonalised U_{ij}.

Atom	x	y	z	U_{eq}
In1	-1109.32(13)	6961.36(7)	2158.93(6)	18.69(3)
In2	948.09(14)	7844.15(7)	2824.58(6)	19.89(3)
F1	-2685.8(13)	7382.7(8)	2373.5(6)	37.5(3)
F2	1337.1(16)	8707.7(8)	2599.0(6)	38.8(4)
N1	-1493.7(18)	6107.8(9)	979.3(7)	25.9(4)
N2	-2004.6(15)	5343.1(8)	1575.0(6)	21.8(3)
N3	-1392(2)	7216.0(10)	1026.2(7)	32.6(5)
N4	-2340.1(19)	5498.0(9)	2360.5(7)	23.2(4)
N5	1825.7(18)	8254.1(10)	4007.2(7)	29.5(4)
N6	3503.8(16)	7766.4(9)	3418.9(6)	23.2(3)
N7	189.8(18)	8770.8(10)	3946.4(7)	31.3(4)
N8	3817.8(16)	7565.9(10)	2624.2(7)	26.2(4)
C1	-1377(2)	6779.2(10)	1313.1(8)	24.2(4)
C2	-1870.2(19)	5841.2(10)	2049.2(8)	21.0(4)
C3	-1571(3)	6128.6(13)	484.1(9)	33.7(5)
C4	-1514(3)	6814.2(13)	509.4(9)	40.5(7)
C5	-2548(2)	4691.6(10)	1594.7(7)	25.3(4)
C6	-2760(2)	4788.5(11)	2085.6(8)	24.1(4)
C11	-2458(3)	5797.3(12)	2918.1(8)	25.8(5)
C12	-1777(4)	6560.4(15)	3163.3(9)	49.7(10)
C13	-1895(3)	5339.1(13)	3196.7(9)	37.3(6)
C14	-3878(3)	5762.1(18)	2945.8(10)	44.8(7)
C15	-2273(2)	4747.9(11)	590.4(7)	28.6(4)
C16	-1851(2)	4137.0(11)	310.3(8)	34.9(5)
C17	-2693(3)	3572.7(13)	-75.0(9)	42.5(6)
C18	-3961(3)	3607.2(14)	-181.2(8)	45.0(7)
C19	-4378(2)	4217.1(14)	90.3(8)	41.6(6)
C20	-3549(2)	4775.6(12)	465.8(7)	33.8(5)
C21	-4875(3)	2981.5(16)	-566.3(9)	69.2(11)
C22	87(2)	5306.3(11)	1171.1(7)	25.3(4)
C23	634(2)	5118.2(12)	1580.4(9)	32.8(5)
C24	1887(2)	5019.5(13)	1633.1(10)	38.9(5)
C25	2657(2)	5094.9(11)	1273.9(10)	35.9(5)
C26	2139(2)	5284.9(12)	864.7(9)	34.2(5)
C27	890(2)	5391.4(12)	817.8(8)	31.6(5)
C28	4028(2)	4988.8(13)	1325.2(12)	49.0(7)
C29	947.7(19)	8359.4(11)	3664.1(8)	26.4(4)
C30	2925.2(19)	7673.4(11)	2940.6(8)	24.4(4)
C31	1594(3)	8579.9(15)	4500.5(9)	40.8(6)
C32	580(2)	8899.8(14)	4463.8(8)	41.5(6)
C33	4762(2)	7711.7(11)	3399.6(8)	27.2(4)
C34	4971(2)	7588.0(12)	2910.2(8)	26.4(4)
C35	-936(2)	9036.7(12)	3766.0(8)	33.4(5)
C36	-825(3)	9818.3(14)	4070.7(11)	66.3(10)
C37	-2097(2)	8596.4(17)	3857.0(11)	54.5(7)
C38	-1023(3)	8948.4(14)	3195.2(9)	43.4(6)
C39	3698(2)	7437.6(13)	2062.0(9)	32.7(5)
C40	4367(4)	8111.3(15)	1992.6(11)	64.3(11)
C41	2327(3)	7231(2)	1807.1(10)	61.9(10)
C42	4359(2)	6815.1(12)	1823.3(8)	34.8(5)
C43	4060(2)	8179.6(13)	4394.9(7)	34.0(5)

Atom	x	y	z	U_{eq}
C44	4593(2)	8887.6(13)	4465.4(8)	41.2(6)
C45	5703(2)	9241.3(16)	4805.8(9)	50.1(7)
C46	6333(3)	8902.2(19)	5100.2(9)	54.2(8)
C47	5777(2)	8223.3(17)	5059.3(8)	49.9(7)
C48	4657(2)	7860.9(15)	4708.6(8)	42.5(6)
C49	7587(3)	9279(2)	5443.2(10)	84.6(13)
C50	2316(2)	6963.7(13)	3861.0(8)	30.4(5)
C51	2650(3)	6373.5(14)	3502.8(11)	51.2(8)
C52	2184(3)	5675.6(15)	3467.0(13)	60.4(9)
C53	1369(3)	5530.7(16)	3790.3(12)	46.5(7)
C54	1012(2)	6108.0(16)	4145.2(9)	43.8(6)
C55	1478(2)	6808.2(15)	4179.9(9)	40.1(6)
C56	841(3)	4778.4(16)	3756.4(13)	68.6(9)
B1	-1391(2)	5386.4(12)	1085.2(8)	23.3(4)
B2	2905(2)	7776.8(14)	3922.2(9)	29.3(5)
C7A	-1110(5)	8002(2)	1199.4(18)	33.0(10)
C8A	-1991(8)	8257(5)	842(3)	62(2)
C9A	264(4)	8276(2)	1197.4(17)	49.0(11)
C10A	-1447(5)	8310.3(19)	1740.3(15)	52.8(14)
C7B	-1502(11)	8013(6)	1226(5)	27.6(19)
C8B	-1880(16)	8228(9)	766(6)	26(2)
C9B	-159(10)	8386(5)	1494(5)	52(3)
C10B	-2418(13)	8197(5)	1598(4)	45(3)
In11	3696.38(13)	7190.23(7)	7153.51(6)	17.58(3)
In12	5719.07(13)	8089.04(7)	7834.08(5)	17.12(3)
F101	3524.0(15)	6257.9(7)	7316.8(6)	31.1(3)
F102	7285.3(13)	7635.5(8)	7667.0(5)	35.6(3)
N101	1084.6(16)	7304.3(9)	6624.5(6)	23.3(3)
N102	2676.3(16)	6892.5(9)	5993.7(6)	24.4(3)
N103	770.3(16)	7281.4(10)	7387.4(7)	23.1(3)
N104	4280.3(17)	6340.5(10)	5978.1(6)	27.3(4)
N105	6181.0(18)	9031.3(9)	8998.3(6)	23.4(4)
N106	6453.7(17)	9727.9(8)	8350.2(6)	25.1(3)
N107	6224(2)	7952.2(9)	9002.5(7)	25.2(4)
N108	7039.6(19)	9500.4(10)	7594.2(7)	24.1(4)
C101	1668.7(19)	7284.5(11)	7075.7(8)	22.0(4)
C102	3601.3(19)	6751.1(10)	6296.7(7)	22.5(4)
C103	-187(2)	7314.7(13)	6654.0(8)	30.3(5)
C104	-392(2)	7302.1(13)	7123.8(8)	29.0(5)
C105	2798(2)	6582.8(12)	5483.2(8)	32.0(5)
C106	3787(2)	6231.4(13)	5471.7(8)	33.4(5)
C107	886(2)	7246.8(15)	7917.8(9)	33.5(5)
C108	251(4)	6515.8(18)	7890.4(14)	76.9(12)
C109	194(3)	7822.6(18)	8232.7(9)	56.7(9)
C110	2257(3)	7441(2)	8175.7(10)	61.1(11)
C111	5371(2)	6005.6(12)	6105.1(8)	30.9(5)
C112	4927(3)	5200.8(14)	5902.6(10)	58.3(8)
C113	5838(2)	6243.5(14)	6680.2(8)	39.6(6)
C114	6453(3)	6233.4(18)	5840.9(11)	63.9(9)
C115	547.9(19)	7226.1(11)	5681.4(7)	24.9(4)
C116	105(2)	7730.3(12)	5492.0(8)	32.0(4)
C117	-980(2)	7551.1(13)	5114.6(8)	37.1(5)
C118	-1663(2)	6860.0(13)	4916.9(8)	34.8(5)
C119	-1233(2)	6348.8(13)	5091.9(8)	32.7(5)
C120	-150(2)	6528.7(12)	5467.0(7)	29.5(4)

Atom	x	y	z	U_{eq}
C121	-2864(2)	6673.1(15)	4523.8(8)	49.4(7)
C122	2488(2)	8232.1(11)	6325.0(8)	25.2(4)
C123	2423(2)	8774.6(12)	6781.0(8)	32.3(5)
C124	3049(2)	9470.8(12)	6896.5(9)	40.2(5)
C125	3780(2)	9662.7(13)	6558.2(10)	41.8(6)
C126	3842(2)	9129.2(14)	6097.8(10)	39.1(6)
C127	3207(2)	8436.1(13)	5986.7(9)	34.9(5)
C128	4472(3)	10415.3(14)	6688.0(12)	62.3(8)
C129	6039(2)	8344.0(10)	8689.8(8)	22.2(4)
C130	6497(2)	9195.1(10)	7910.0(8)	22.1(4)
C131	6443(3)	9068.4(13)	9507.6(9)	33.6(5)
C132	6478(3)	8402.8(13)	9512.8(9)	34.0(6)
C133	6957(2)	10366.7(11)	8304.0(8)	33.8(5)
C134	7336(2)	10228.7(11)	7834.6(9)	30.0(5)
C135	6153(2)	7173.8(11)	8870.2(8)	24.7(4)
C136	7444(2)	7049.7(14)	9069.7(10)	42.4(6)
C137	5841(3)	6786.6(12)	8293.3(9)	43.2(7)
C138	5122(3)	6908.9(13)	9132.7(11)	51.7(7)
C139	7451(3)	9141.1(13)	7092.7(9)	29.4(5)
C140	8830(3)	9073.4(19)	7205.2(13)	70.6(11)
C141	7335(4)	9598.4(16)	6751.9(10)	67.6(12)
C142	6662(4)	8412.2(16)	6813.8(10)	52.5(10)
C143	6610(2)	10415.2(11)	9314.1(7)	28.1(4)
C144	7922(2)	10496.4(12)	9472.5(8)	34.3(5)
C145	8637(2)	11126.1(13)	9825.6(8)	39.2(5)
C146	8060(3)	11704.4(13)	10041.9(8)	36.2(5)
C147	6756(2)	11629.9(12)	9898.3(8)	38.9(5)
C148	6050(2)	10994.6(11)	9544.0(8)	33.1(5)
C149	8862(3)	12394.0(14)	10400.6(9)	54.4(8)
C150	4352(2)	9651.0(11)	8759.3(8)	27.3(4)
C151	3672(2)	9766.6(13)	8342.6(9)	36.5(5)
C152	2373(3)	9774.7(15)	8307.8(10)	45.8(6)
C153	1696(3)	9677.1(14)	8685.4(10)	44.6(6)
C154	2357(3)	9549.3(13)	9097.9(9)	39.4(6)
C155	3648(2)	9527.9(12)	9129.2(9)	32.7(5)
C156	306(3)	9730.1(17)	8666.8(13)	64.9(9)
B101	1712(2)	7423.4(13)	6157.5(8)	22.8(4)
B102	5875(2)	9694.7(12)	8849.8(8)	23.8(4)

7.5.8 Anisotropic Displacement and Structural Parameters

Table S37. Anisotropic Displacement Parameters (×10^4) for **3-F**. The anisotropic displacement factor exponent takes the form: $-2\pi^2[h^2a^{*2} \times U_{11} + ... +2hka^* \times b^* \times U_{12}]$

**Atom**	U_{11}	U_{22}	U_{33}	U_{23}	U_{13}	U_{12}
In1	16.88(7)	18.13(6)	19.30(6)	-0.44(5)	3.35(5)	5.24(5)
In2	16.15(7)	20.60(6)	19.94(6)	0.49(5)	3.69(5)	3.65(5)
F1	23.0(7)	34.3(7)	54.4(8)	8.5(6)	17.6(6)	9.0(6)
F2	27.8(8)	38.4(8)	58.2(9)	-4.6(6)	6.4(7)	31.8(7)
N1	30.5(10)	25.0(8)	20.3(8)	-2.6(7)	3.7(7)	8.3(6)
N2	22.0(8)	21.6(7)	19.5(7)	-2.2(6)	4.8(6)	5.8(6)
N3	42.2(12)	27.7(9)	27.3(9)	-2.6(8)	-2.5(8)	14.6(7)
N4	28.5(10)	22.7(8)	19.5(8)	2.3(7)	7.6(7)	8.3(6)
N5	23.9(9)	37.9(10)	23.0(8)	6.1(7)	6.3(7)	4.0(7)
N6	17.2(8)	27.8(8)	23.2(8)	3.2(6)	5.5(6)	6.4(6)
N7	24.5(9)	34.8(10)	25.0(9)	5.8(8)	4.0(7)	-3.2(7)
N8	17.9(8)	34.3(10)	24.7(8)	7.0(7)	6.0(7)	5.9(7)
C1	26.0(11)	22.6(9)	22.4(9)	-2.6(7)	-0.1(8)	9.7(7)
C2	22.3(10)	20.2(8)	19.5(8)	1.3(7)	5.2(7)	5.5(7)
C3	47.1(15)	30.7(12)	20.6(10)	-2.6(10)	2.8(9)	9.9(9)
C4	58.4(18)	38.1(13)	24.9(11)	-2.8(12)	0.1(10)	17.3(10)
C5	29.8(11)	21.0(9)	21.3(9)	-3.6(7)	3.5(7)	5.3(7)
C6	26.8(11)	22.1(9)	22.1(9)	-2.5(7)	6.0(8)	7.9(7)
C11	31.7(14)	26.2(10)	20.4(9)	4.9(9)	7.7(9)	8.0(8)
C12	89(3)	31.4(13)	20.8(11)	-8.2(15)	16.9(14)	3.7(9)
C13	47.8(16)	35.8(12)	26.4(11)	3.8(11)	-3.7(10)	12.6(9)
C14	38.2(14)	75(2)	30.6(12)	26.0(14)	17.4(10)	20.1(12)
C15	33.9(12)	26.5(10)	20.8(9)	-4.0(8)	6.0(8)	5.2(8)
C16	42.9(14)	26.8(11)	27.4(10)	-4.9(9)	9.3(9)	2.2(8)
C17	54.7(18)	34.1(13)	28.3(11)	-8.2(11)	12.6(11)	1.0(9)
C18	53.5(17)	43.4(14)	25.8(11)	-21.0(12)	1.8(10)	8.4(10)
C19	34.0(13)	53.1(15)	28.5(11)	-17.1(11)	-1.0(9)	13.2(10)
C20	27.1(11)	42.0(13)	24.9(9)	-10.4(9)	-1.2(8)	9.9(9)
C21	81(2)	68(2)	29.7(12)	-44.2(17)	1.5(13)	4.1(12)
C22	26.7(11)	24.5(9)	22.9(9)	-0.1(8)	7.6(8)	6.3(7)
C23	30.2(12)	36.5(11)	40.5(12)	10.0(9)	13.3(9)	20.9(10)
C24	30.4(12)	44.3(13)	53.8(14)	12.0(10)	13.7(11)	28.2(11)
C25	28.2(11)	25.8(10)	55.0(14)	7.4(8)	19.3(10)	10.2(10)
C26	31.5(13)	32.3(11)	32.9(11)	-0.7(9)	16.0(9)	2.0(9)
C27	29.7(12)	36.2(11)	24.9(10)	-3.7(9)	11.1(9)	6.8(8)
C28	33.4(13)	31.9(13)	86(2)	11.4(10)	24.4(14)	19.0(13)
C29	20.1(10)	29.5(10)	23.1(9)	2.2(8)	5.7(8)	0.1(8)
C30	18.8(10)	29.8(10)	21.7(9)	3.7(8)	6.0(8)	4.2(7)
C31	31.7(14)	60.1(17)	21.6(10)	8.5(12)	6.3(10)	0.8(11)
C32	26.8(12)	59.4(16)	26.0(11)	11.7(11)	6.4(9)	-4.6(10)
C33	21.7(11)	33.0(11)	26.3(10)	7.5(8)	3.0(8)	8.4(8)
C34	18.6(10)	33.0(11)	28.1(10)	6.2(8)	7.8(8)	9.0(8)
C35	23.9(11)	33.2(11)	34.7(12)	9.0(9)	4.0(9)	-1.5(9)
C36	50.3(18)	46.7(16)	71.8(19)	27.1(14)	-18.0(15)	-20.0(14)
C37	25.6(13)	77(2)	60.1(17)	8.7(13)	11.6(12)	21.1(15)
C38	37.1(14)	46.5(15)	45.9(14)	21.2(12)	9.9(11)	8.0(11)
C39	27.5(12)	42.2(13)	29.6(11)	12.0(10)	8.9(9)	10.3(10)
C40	127(3)	37.6(16)	39.8(15)	27.9(18)	28.7(18)	17.8(13)
C41	39.2(17)	122(3)	27.7(12)	34.2(18)	7.6(11)	20.7(16)
C42	37.5(13)	32.2(12)	28.2(11)	5.0(9)	8.4(9)	0.6(9)

Atom	U_{11}	U_{22}	U_{33}	U_{23}	U_{13}	U_{12}
C43	23.5(10)	50.0(14)	22.4(10)	2.3(10)	4.0(8)	5.4(9)
C44	28.8(12)	47.5(14)	34.3(11)	3.5(10)	1.8(9)	-1.4(10)
C45	30.3(13)	64.9(18)	39.3(13)	3.7(12)	6.4(10)	-2.3(12)
C46	27.4(13)	92(2)	25.8(12)	-4.0(14)	-1.8(10)	4.0(13)
C47	30.9(13)	92(2)	24.0(11)	10.3(14)	0.3(9)	16.6(13)
C48	27.3(12)	72.1(18)	28.2(11)	12.6(12)	4.7(9)	15.6(11)
C49	43.1(18)	144(3)	37.8(15)	-11(2)	-1.9(13)	4.5(18)
C50	20.0(10)	43.2(13)	32.9(11)	6.4(9)	6.3(8)	18.5(10)
C51	55.8(18)	36.4(14)	78.1(19)	18.0(12)	45.7(16)	27.0(14)
C52	61(2)	40.4(15)	101(2)	20.6(14)	49.7(18)	35.7(16)
C53	29.4(13)	55.7(17)	69.5(18)	8.4(12)	9.4(12)	41.1(15)
C54	29.9(13)	68.4(19)	37.0(13)	-4.3(12)	3.1(10)	29.2(13)
C55	30.3(12)	59.4(17)	27.3(11)	-1.5(11)	8.3(9)	12.8(11)
C56	44.5(18)	70(2)	123(3)	14.7(15)	21.1(18)	71(2)
B1	26.4(12)	20.7(10)	20.9(10)	-0.4(8)	7.0(8)	5.1(8)
B2	24.0(12)	40.3(14)	21.3(11)	3.4(10)	5.0(9)	7.6(10)
C7A	33(2)	29.8(16)	35.6(18)	-4.3(13)	-7.2(14)	17.7(12)
C8A	66(5)	43(3)	68(4)	6(2)	-21(3)	19(2)
C9A	40(2)	36.7(19)	65(3)	-9.0(14)	-5.2(16)	21.2(18)
C10A	83(4)	27.0(17)	56(2)	14.8(18)	22.8(19)	17.9(14)
C7B	34(3)	20(3)	30(4)	-5.1(16)	-7.2(17)	17(2)
C8B	27(5)	19(5)	30(4)	-11(3)	-8(3)	15(3)
C9B	43(5)	29(4)	73(7)	-9(3)	-18(3)	17(4)
C10B	65(6)	28(4)	49(5)	10(3)	12(3)	20(3)
In11	14.53(7)	20.47(6)	17.55(6)	2.08(5)	3.10(5)	6.41(5)
In12	15.18(6)	19.47(6)	16.78(6)	2.47(5)	3.08(5)	6.32(4)
F101	29.6(8)	29.7(7)	43.7(8)	3.7(5)	8.2(6)	25.5(6)
F102	22.0(7)	36.5(7)	40.6(7)	7.2(6)	8.1(6)	0.7(6)
N101	18.2(8)	31.6(9)	21.3(8)	4.6(6)	3.8(6)	10.5(6)
N102	19.4(8)	33.2(9)	19.7(7)	3.5(7)	3.3(6)	8.1(7)
N103	17.5(8)	32.8(9)	22.4(8)	7.7(7)	6.6(6)	11.3(7)
N104	22.1(9)	32.6(9)	23.1(8)	5.7(7)	6.8(7)	2.8(7)
N105	28.8(10)	22.6(8)	19.0(7)	3.3(7)	5.6(6)	7.1(6)
N106	31.5(9)	21.8(8)	23.9(8)	3.9(7)	9.9(7)	8.9(6)
N107	29.9(10)	25.7(8)	21.7(8)	5.2(7)	1.4(7)	11.2(7)
N108	26.1(10)	26.4(8)	23.7(8)	4.0(7)	10.0(7)	12.4(7)
C101	17.5(9)	28.3(9)	20.3(9)	4.6(7)	3.6(7)	7.9(7)
C102	20.8(9)	25.4(9)	20.4(9)	4.3(7)	7.0(7)	5.1(7)
C103	18.0(10)	51.5(13)	24.2(10)	12.0(9)	5.9(8)	13.4(9)
C104	19.3(10)	45.5(13)	25.9(10)	12.5(9)	7.1(8)	13.1(9)
C105	30.1(11)	45.5(13)	19.2(9)	12.2(10)	5.3(8)	6.4(9)
C106	31.1(13)	45.4(13)	21.9(10)	14.5(10)	8.5(9)	4.6(9)
C107	23.3(11)	53.9(16)	32.8(11)	12.4(10)	11.8(9)	23.3(11)
C108	97(3)	68(2)	83(2)	6(2)	14(2)	53(2)
C109	44.9(17)	100(3)	26.3(12)	39.0(17)	8.9(11)	10.6(14)
C110	27.8(14)	140(3)	31.8(13)	35.1(17)	11.1(11)	41.4(17)
C111	30.3(12)	33.1(11)	26.5(10)	8.4(9)	10.5(9)	3.1(8)
C112	80(2)	37.1(14)	49.1(16)	20.1(15)	-0.2(15)	1.6(12)
C113	33.2(13)	46.1(14)	34.5(12)	21.8(11)	0.2(10)	2.4(10)
C114	38.9(16)	103(3)	79(2)	36.0(17)	36.4(15)	52(2)
C115	19.8(9)	34.0(11)	21.7(9)	3.6(8)	4.7(7)	10.6(8)
C116	24.3(10)	38.0(12)	34.1(11)	6.0(9)	-0.8(8)	14.6(9)
C117	28.5(11)	47.0(14)	37.4(11)	6.4(10)	-2.4(9)	19.2(10)
C118	26.6(11)	48.3(14)	24.8(10)	4.7(10)	-0.8(8)	8.2(9)
C119	25.4(11)	36.8(12)	31.5(11)	4.6(9)	5.0(9)	5.8(9)

Atom	U_{11}	U_{22}	U_{33}	U_{23}	U_{13}	U_{12}
C120	24.0(10)	37.0(12)	27.6(10)	3.8(9)	3.0(8)	12.3(9)
C121	37.0(14)	69.4(19)	30.5(11)	4.2(13)	-9.0(10)	8.2(12)
C122	19.5(9)	30.8(11)	24.9(10)	3.4(8)	2.2(7)	9.8(8)
C123	38.4(13)	30.3(11)	29.5(11)	7.7(9)	7.3(9)	10.6(9)
C124	46.6(15)	31.1(12)	40.9(13)	9.1(10)	4.8(11)	9.3(10)
C125	37.2(14)	33.4(13)	56.4(15)	4.4(10)	7.5(12)	17.9(12)
C126	33.0(13)	38.9(14)	48.2(15)	2.4(10)	10.5(11)	18.8(12)
C127	28.4(12)	38.6(13)	37.1(12)	1.6(9)	9.5(9)	12.6(10)
C128	56(2)	33.1(14)	94(2)	2.4(13)	11.2(17)	19.3(15)
C129	25.9(10)	21.6(9)	18.1(8)	2.0(7)	2.4(7)	6.9(7)
C130	24.4(11)	20.7(9)	21.1(9)	0.3(7)	5.1(7)	8.2(7)
C131	46.1(15)	32.3(12)	18.4(9)	0.7(10)	4.6(9)	6.0(8)
C132	49.9(16)	32.4(12)	17.6(9)	2.9(11)	0.5(9)	8.7(9)
C133	50.6(15)	19.0(9)	33.5(11)	1.8(9)	16.7(10)	9.6(8)
C134	37.2(14)	23.5(10)	31.2(11)	-0.8(9)	11.9(10)	12.3(8)
C135	25.4(11)	26.8(10)	26.6(10)	9.7(8)	4.2(8)	13.5(8)
C136	33.8(13)	41.9(14)	51.5(15)	13.5(11)	-2.7(11)	16.0(12)
C137	67(2)	22.7(11)	34.9(12)	11.4(11)	-9.4(12)	7.4(9)
C138	56.9(18)	31.7(13)	75.8(19)	6.9(12)	37.7(15)	22.8(13)
C139	35.4(15)	29.2(11)	26.1(11)	4.7(10)	13.8(10)	10.6(9)
C140	36.1(16)	76(2)	80(2)	22.0(16)	21.0(17)	-9.0(18)
C141	127(4)	48.4(17)	38.1(15)	15.1(19)	34.3(18)	23.0(13)
C142	77(3)	45.0(16)	24.6(12)	-13.7(16)	20.1(14)	2.5(11)
C143	31.1(11)	24.7(10)	26.8(10)	1.5(8)	8.9(8)	6.6(8)
C144	31.7(12)	31.8(12)	33.3(11)	2.0(9)	6.3(9)	4.0(9)
C145	34.9(13)	42.5(14)	36.3(12)	-2.4(10)	3.5(10)	12.4(10)
C146	42.4(15)	31.7(12)	28.0(11)	0.1(10)	3.4(9)	4.5(9)
C147	45.3(15)	29.1(11)	34.8(11)	3.2(10)	8.5(10)	1.1(9)
C148	37.7(13)	24.5(10)	33.9(11)	5.3(9)	9.5(9)	4.2(8)
C149	65(2)	44.3(15)	36.1(13)	-11.1(13)	-4.1(12)	0.9(11)
C150	31.7(12)	24.9(9)	28.7(10)	6.5(8)	12.8(9)	10.3(8)
C151	36.5(13)	44.3(13)	40.1(12)	16.6(11)	16.2(11)	22.7(10)
C152	35.9(14)	61.3(17)	55.5(15)	22.5(12)	16.2(12)	32.4(13)
C153	37.6(14)	45.0(14)	57.1(15)	18.3(11)	21.7(12)	16.5(12)
C154	39.0(15)	40.3(13)	41.3(13)	8.9(11)	19.3(11)	12.3(10)
C155	34.0(14)	34.6(11)	28.8(11)	2.2(9)	12.4(9)	9.1(9)
C156	43.2(17)	75(2)	89(2)	30.6(16)	31.3(17)	29.5(19)
B101	16.5(10)	32.0(11)	20.6(10)	3.3(8)	2.2(7)	10.6(9)
B102	27.7(12)	20.1(10)	22.3(10)	0.9(8)	7.6(9)	5.8(8)

Table S38. Bond Lengths in Å for **3-F**.

Atom	Atom	Length/Å
In1	In2	2.72571(18)
In1	F1	2.0597(13)
In1	C1	2.232(2)
In1	C2	2.2065(19)
In2	F2	2.0354(13)
In2	C29	2.224(2)
In2	C30	2.207(2)
N1	C1	1.355(3)
N1	C3	1.378(3)
N1	B1	1.587(3)

Atom	Atom	Length/Å
N2	C2	1.354(2)
N2	C5	1.383(2)
N2	B1	1.592(2)
N3	C1	1.361(3)
N3	C4	1.384(3)
N3	C7A	1.480(5)
N3	C7B	1.551(12)
N4	C2	1.362(2)
N4	C6	1.374(3)
N4	C11	1.495(3)

Atom	Atom	Length/Å
N5	C29	1.352(3)
N5	C31	1.381(3)
N5	B2	1.597(3)
N6	C30	1.345(3)
N6	C33	1.379(3)
N6	B2	1.592(3)
N7	C29	1.369(3)
N7	C32	1.378(3)
N7	C35	1.490(3)
N8	C30	1.362(2)
N8	C34	1.385(3)
N8	C39	1.482(3)
C3	C4	1.354(3)
C5	C6	1.359(3)
C11	C12	1.512(3)
C11	C13	1.523(3)
C11	C14	1.532(4)
C15	C16	1.395(3)
C15	C20	1.393(3)
C15	B1	1.643(3)
C16	C17	1.412(3)
C17	C18	1.381(4)
C18	C19	1.383(4)
C18	C21	1.509(3)
C19	C20	1.388(3)
C22	C23	1.401(3)
C22	C27	1.408(3)
C22	B1	1.620(3)
C23	C24	1.389(3)
C24	C25	1.392(3)
C25	C26	1.392(4)
C25	C28	1.518(3)
C26	C27	1.388(3)
C31	C32	1.351(4)
C33	C34	1.347(3)
C35	C36	1.514(3)
C35	C37	1.523(3)
C35	C38	1.524(3)
C39	C40	1.526(4)
C39	C41	1.507(4)
C39	C42	1.528(3)
C43	C44	1.407(3)
C43	C48	1.388(3)
C43	B2	1.617(3)
C44	C45	1.386(3)
C45	C46	1.397(4)
C46	C47	1.381(4)
C46	C49	1.512(4)
C47	C48	1.409(3)
C50	C51	1.404(3)
C50	C55	1.400(3)
C50	B2	1.623(3)
C51	C52	1.396(3)
C52	C53	1.386(3)
C53	C54	1.394(4)

Atom	Atom	Length/Å
C53	C56	1.515(4)
C54	C55	1.401(4)
C7A	C8A	1.548(8)
C7A	C9A	1.507(5)
C7A	C10A	1.532(6)
C7B	C8B	1.502(15)
C7B	C9B	1.525(13)
C7B	C10B	1.499(13)
In11	In12	2.72844(18)
In11	F101	2.0573(13)
In11	C101	2.210(2)
In11	C102	2.2381(19)
In12	F102	2.0520(13)
In12	C129	2.2354(19)
In12	C130	2.2070(19)
N101	C101	1.351(3)
N101	C103	1.382(2)
N101	B101	1.589(3)
N102	C102	1.351(3)
N102	C105	1.380(2)
N102	B101	1.589(3)
N103	C101	1.364(2)
N103	C104	1.392(3)
N103	C107	1.485(3)
N104	C102	1.355(2)
N104	C106	1.381(3)
N104	C111	1.495(3)
N105	C129	1.354(2)
N105	C131	1.384(3)
N105	B102	1.583(3)
N106	C130	1.355(3)
N106	C133	1.377(2)
N106	B102	1.593(3)
N107	C129	1.361(3)
N107	C132	1.389(3)
N107	C135	1.483(3)
N108	C130	1.356(2)
N108	C134	1.380(3)
N108	C139	1.493(3)
C103	C104	1.350(3)
C105	C106	1.354(3)
C107	C108	1.512(4)
C107	C109	1.547(4)
C107	C110	1.510(3)
C111	C112	1.526(3)
C111	C113	1.515(3)
C111	C114	1.534(3)
C115	C116	1.397(3)
C115	C120	1.407(3)
C115	B101	1.625(3)
C116	C117	1.403(3)
C117	C118	1.390(3)
C118	C119	1.382(3)
C118	C121	1.516(3)
C119	C120	1.397(3)

Atom	Atom	Length/Å
C122	C123	1.399(3)
C122	C127	1.400(3)
C122	B101	1.623(3)
C123	C124	1.392(3)
C124	C125	1.397(3)
C125	C126	1.398(3)
C125	C128	1.508(3)
C126	C127	1.391(3)
C131	C132	1.352(3)
C133	C134	1.359(3)
C135	C136	1.521(3)
C135	C137	1.514(3)
C135	C138	1.518(3)
C139	C140	1.517(4)
C139	C141	1.521(4)
C139	C142	1.508(3)

Atom	Atom	Length/Å
C143	C144	1.404(3)
C143	C148	1.391(3)
C143	B102	1.634(3)
C144	C145	1.394(3)
C145	C146	1.391(4)
C146	C147	1.391(4)
C146	C149	1.508(3)
C147	C148	1.399(3)
C150	C151	1.399(3)
C150	C155	1.401(3)
C150	B102	1.617(3)
C151	C152	1.394(3)
C152	C153	1.385(3)
C153	C154	1.393(4)
C153	C156	1.510(4)
C154	C155	1.390(3)

Table S39. Bond Angles in ° for **3-F**.

Atom	Atom	Atom	Angle/°
F1	In1	In2	106.91(4)
C1	In1	In2	122.54(5)
C1	In1	F1	102.24(7)
C2	In1	In2	128.98(5)
C2	In1	F1	97.98(7)
C2	In1	C1	93.30(7)
F2	In2	In1	107.28(5)
C29	In2	In1	123.44(5)
C29	In2	F2	101.29(7)
C30	In2	In1	129.17(5)
C30	In2	F2	96.52(7)
C30	In2	C29	93.48(8)
C3	N1	C1	108.81(18)
B1	N1	C1	130.36(17)
B1	N1	C3	120.39(18)
C5	N2	C2	109.13(15)
B1	N2	C2	129.28(15)
B1	N2	C5	120.15(15)
C4	N3	C1	109.26(19)
C7A	N3	C1	129.1(2)
C7A	N3	C4	120.9(2)
C7B	N3	C1	125.9(5)
C7B	N3	C4	124.0(5)
C7B	N3	C7A	16.4(4)
C6	N4	C2	110.19(17)
C11	N4	C2	128.84(17)
C11	N4	C6	120.96(16)
C31	N5	C29	109.1(2)
B2	N5	C29	130.93(17)
B2	N5	C31	119.7(2)
C33	N6	C30	108.43(16)
B2	N6	C30	129.73(17)
B2	N6	C33	120.70(18)
C32	N7	C29	109.3(2)

Atom	Atom	Atom	Angle/°
C35	N7	C29	129.43(18)
C35	N7	C32	121.25(18)
C34	N8	C30	109.26(18)
C39	N8	C30	129.90(19)
C39	N8	C34	120.84(17)
N1	C1	In1	118.94(14)
N3	C1	In1	133.96(15)
N3	C1	N1	107.00(18)
N2	C2	In1	119.44(13)
N4	C2	In1	134.14(15)
N4	C2	N2	106.31(16)
C4	C3	N1	108.2(2)
C3	C4	N3	106.7(2)
C6	C5	N2	107.84(17)
C5	C6	N4	106.52(17)
C12	C11	N4	112.18(17)
C13	C11	N4	108.1(2)
C13	C11	C12	109.6(2)
C14	C11	N4	107.0(2)
C14	C11	C12	109.3(3)
C14	C11	C13	110.7(2)
C20	C15	C16	116.9(2)
B1	C15	C16	123.7(2)
B1	C15	C20	119.2(2)
C17	C16	C15	121.0(2)
C18	C17	C16	120.9(2)
C19	C18	C17	118.2(2)
C21	C18	C17	120.6(3)
C21	C18	C19	121.1(3)
C20	C19	C18	121.0(3)
C19	C20	C15	122.0(2)
C27	C22	C23	115.7(2)
B1	C22	C23	123.09(17)
B1	C22	C27	121.14(19)

Atom	Atom	Atom	Angle/°
C24	C23	C22	122.1(2)
C25	C24	C23	121.1(2)
C26	C25	C24	118.0(2)
C28	C25	C24	121.8(2)
C28	C25	C26	120.2(2)
C27	C26	C25	120.7(2)
C26	C27	C22	122.4(2)
N5	C29	In2	119.02(14)
N7	C29	In2	134.33(16)
N7	C29	N5	106.59(17)
N6	C30	In2	119.48(14)
N8	C30	In2	132.72(16)
N8	C30	N6	107.26(18)
C32	C31	N5	108.0(2)
C31	C32	N7	107.1(2)
C34	C33	N6	108.89(19)
C33	C34	N8	106.17(18)
C36	C35	N7	109.50(18)
C37	C35	N7	106.4(2)
C37	C35	C36	111.1(2)
C38	C35	N7	111.00(18)
C38	C35	C36	108.6(2)
C38	C35	C37	110.3(2)
C40	C39	N8	107.4(2)
C41	C39	N8	111.43(19)
C41	C39	C40	112.4(3)
C42	C39	N8	107.99(19)
C42	C39	C40	110.0(2)
C42	C39	C41	107.5(2)
C48	C43	C44	116.6(2)
B2	C43	C44	118.1(2)
B2	C43	C48	125.0(2)
C45	C44	C43	122.4(3)
C46	C45	C44	120.5(3)
C47	C46	C45	117.7(2)
C49	C46	C45	119.7(3)
C49	C46	C47	122.6(3)
C48	C47	C46	121.7(3)
C47	C48	C43	121.0(3)
C55	C50	C51	115.7(2)
B2	C50	C51	122.53(19)
B2	C50	C55	121.7(2)
C52	C51	C50	122.3(2)
C53	C52	C51	121.2(3)
C54	C53	C52	117.4(3)
C56	C53	C52	122.3(3)
C56	C53	C54	120.2(2)
C55	C54	C53	121.2(2)
C54	C55	C50	122.0(2)
N2	B1	N1	112.36(16)
C15	B1	N1	106.26(17)
C15	B1	N2	105.26(15)
C22	B1	N1	109.74(16)
C22	B1	N2	109.20(16)
C22	B1	C15	113.99(17)

Atom	Atom	Atom	Angle/°
N6	B2	N5	111.89(18)
C43	B2	N5	106.12(18)
C43	B2	N6	104.46(17)
C50	B2	N5	110.00(18)
C50	B2	N6	108.17(17)
C50	B2	C43	116.1(2)
C8A	C7A	N3	108.7(4)
C9A	C7A	N3	111.6(4)
C9A	C7A	C8A	110.4(5)
C10A	C7A	N3	109.7(3)
C10A	C7A	C8A	105.9(5)
C10A	C7A	C9A	110.3(4)
C8B	C7B	N3	107.9(10)
C9B	C7B	N3	103.6(8)
C9B	C7B	C8B	110.9(10)
C10B	C7B	N3	115.0(8)
C10B	C7B	C8B	109.6(11)
C10B	C7B	C9B	109.6(10)
F101	In11	In12	106.12(4)
C101	In11	In12	129.39(5)
C101	In11	F101	98.31(7)
C102	In11	In12	125.38(5)
C102	In11	F101	99.61(7)
C102	In11	C101	92.00(7)
F102	In12	In11	106.35(4)
C129	In12	In11	126.28(5)
C129	In12	F102	98.92(7)
C130	In12	In11	128.10(5)
C130	In12	F102	99.41(7)
C130	In12	C129	91.91(7)
C103	N101	C101	108.63(16)
B101	N101	C101	128.31(16)
B101	N101	C103	122.37(17)
C105	N102	C102	109.07(18)
B101	N102	C102	128.70(16)
B101	N102	C105	121.51(17)
C104	N103	C101	108.81(17)
C107	N103	C101	130.00(18)
C107	N103	C104	121.18(17)
C106	N104	C102	109.40(19)
C111	N104	C102	129.67(17)
C111	N104	C106	120.89(18)
C131	N105	C129	108.86(17)
B102	N105	C129	128.72(17)
B102	N105	C131	121.57(17)
C133	N106	C130	108.90(16)
B102	N106	C130	129.60(16)
B102	N106	C133	121.43(16)
C132	N107	C129	108.92(18)
C135	N107	C129	129.96(18)
C135	N107	C132	121.10(18)
C134	N108	C130	109.68(18)
C139	N108	C130	128.01(18)
C139	N108	C134	121.81(17)
N101	C101	In11	118.96(13)

Atom	Atom	Atom	Angle/°
N103	C101	In11	133.49(15)
N103	C101	N101	107.37(17)
N102	C102	In11	118.40(13)
N104	C102	In11	134.65(15)
N104	C102	N102	106.94(17)
C104	C103	N101	108.44(19)
C103	C104	N103	106.74(18)
C106	C105	N102	107.7(2)
C105	C106	N104	106.88(19)
C108	C107	N103	108.7(2)
C109	C107	N103	106.8(2)
C109	C107	C108	110.4(2)
C110	C107	N103	110.96(18)
C110	C107	C108	113.1(3)
C110	C107	C109	106.6(2)
C112	C111	N104	107.9(2)
C113	C111	N104	112.41(17)
C113	C111	C112	109.0(2)
C114	C111	N104	107.1(2)
C114	C111	C112	111.7(2)
C114	C111	C113	108.7(2)
C120	C115	C116	116.02(19)
B101	C115	C116	123.48(19)
B101	C115	C120	120.26(18)
C117	C116	C115	121.8(2)
C118	C117	C116	120.8(2)
C119	C118	C117	118.5(2)
C121	C118	C117	120.8(2)
C121	C118	C119	120.7(2)
C120	C119	C118	120.5(2)
C119	C120	C115	122.3(2)
C127	C122	C123	115.7(2)
B101	C122	C123	124.17(18)
B101	C122	C127	119.93(19)
C124	C123	C122	122.3(2)
C125	C124	C123	121.2(2)
C126	C125	C124	117.2(2)
C128	C125	C124	121.0(2)
C128	C125	C126	121.7(2)
C127	C126	C125	120.9(2)
C126	C127	C122	122.6(2)
N105	C129	In12	118.81(13)
N107	C129	In12	133.80(14)
N107	C129	N105	107.20(17)
N106	C130	In12	119.24(13)
N108	C130	In12	133.86(15)
N108	C130	N106	106.90(17)
C132	C131	N105	107.9(2)

Atom	Atom	Atom	Angle/°
C131	C132	N107	107.1(2)
C134	C133	N106	108.03(18)
C133	C134	N108	106.48(17)
C136	C135	N107	107.65(19)
C137	C135	N107	112.03(17)
C137	C135	C136	108.9(2)
C138	C135	N107	107.47(18)
C138	C135	C136	111.2(2)
C138	C135	C137	109.5(2)
C140	C139	N108	107.9(2)
C141	C139	N108	108.9(2)
C141	C139	C140	109.5(2)
C142	C139	N108	112.13(19)
C142	C139	C140	109.3(3)
C142	C139	C141	109.1(2)
C148	C143	C144	116.0(2)
B102	C143	C144	119.21(19)
B102	C143	C148	124.6(2)
C145	C144	C143	122.4(2)
C146	C145	C144	120.5(2)
C147	C146	C145	118.0(2)
C149	C146	C145	119.7(3)
C149	C146	C147	122.3(2)
C148	C147	C146	120.9(2)
C147	C148	C143	122.1(2)
C155	C150	C151	116.2(2)
B102	C150	C151	124.31(18)
B102	C150	C155	119.4(2)
C152	C151	C150	121.4(2)
C153	C152	C151	121.8(2)
C154	C153	C152	117.3(2)
C156	C153	C152	122.1(3)
C156	C153	C154	120.6(2)
C155	C154	C153	121.0(2)
C154	C155	C150	122.2(2)
N102	B101	N101	110.10(16)
C115	B101	N101	105.68(15)
C115	B101	N102	108.58(16)
C122	B101	N101	110.30(16)
C122	B101	N102	108.43(16)
C122	B101	C115	113.71(17)
N106	B102	N105	111.32(16)
C143	B102	N105	108.00(17)
C143	B102	N106	105.04(15)
C150	B102	N105	108.99(16)
C150	B102	N106	110.89(17)
C150	B102	C143	112.55(18)

Table S40. Torsion Angles in ° for **3-F**.

Atom	Atom	Atom	Atom	Angle/°
In1	C1	N1	C3	-176.38(18)
In1	C1	N1	B1	-4.1(2)
In1	C1	N3	C4	175.9(2)

Atom	Atom	Atom	Atom	Angle/°
In1	C1	N3	C7A	6.1(4)
In1	C1	N3	C7B	-14.2(6)
In1	C2	N2	C5	-177.27(16)
In1	C2	N2	B1	16.7(2)
In1	C2	N4	C6	176.5(2)
In1	C2	N4	C11	-2.1(3)
In2	C29	N5	C31	-175.87(17)
In2	C29	N5	B2	-2.7(2)
In2	C29	N7	C32	175.3(2)
In2	C29	N7	C35	-1.9(3)
In2	C30	N6	C33	-173.03(15)
In2	C30	N6	B2	19.4(2)
In2	C30	N8	C34	171.6(2)
In2	C30	N8	C39	-8.6(3)
N1	C1	N3	C4	-0.2(2)
N1	C1	N3	C7A	-170.0(3)
N1	C1	N3	C7B	169.6(5)
N1	C3	C4	N3	0.4(2)
N1	B1	N2	C2	-45.9(2)
N1	B1	N2	C5	149.37(17)
N1	B1	C15	C16	129.68(16)
N1	B1	C15	C20	-56.43(18)
N1	B1	C22	C23	132.57(17)
N1	B1	C22	C27	-50.5(2)
N2	C2	N4	C6	0.4(2)
N2	C2	N4	C11	-178.18(18)
N2	C5	C6	N4	-0.2(2)
N2	B1	N1	C1	38.0(2)
N2	B1	N1	C3	-150.49(19)
N2	B1	C15	C16	-110.95(17)
N2	B1	C15	C20	62.94(18)
N2	B1	C22	C23	9.0(2)
N2	B1	C22	C27	-174.05(16)
N3	C1	N1	C3	0.5(2)
N3	C1	N1	B1	172.71(16)
N4	C2	N2	C5	-0.5(2)
N4	C2	N2	B1	-166.52(16)
N5	C29	N7	C32	-1.8(2)
N5	C29	N7	C35	-178.97(16)
N5	C31	C32	N7	-0.1(2)
N5	B2	N6	C30	-46.1(2)
N5	B2	N6	C33	147.64(17)
N5	B2	C43	C44	-56.90(19)
N5	B2	C43	C48	129.86(18)
N5	B2	C50	C51	140.0(2)
N5	B2	C50	C55	-42.8(2)
N6	C30	N8	C34	0.4(2)
N6	C30	N8	C39	-179.89(16)
N6	C33	C34	N8	-0.05(19)
N6	B2	N5	C29	35.7(2)
N6	B2	N5	C31	-151.79(19)
N6	B2	C43	C44	61.5(2)
N6	B2	C43	C48	-111.76(19)
N6	B2	C50	C51	17.5(3)
N6	B2	C50	C55	-165.33(18)

Atom	Atom	Atom	Atom	Angle/°
N7	C29	N5	C31	1.8(2)
N7	C29	N5	B2	174.93(16)
N8	C30	N6	C33	-0.4(2)
N8	C30	N6	B2	-168.01(17)
C1	N1	C3	C4	-0.5(2)
C1	N1	B1	C15	152.6(2)
C1	N1	B1	C22	-83.7(2)
C1	N3	C4	C3	-0.1(2)
C1	N3	C7A	C8A	-144.8(5)
C1	N3	C7A	C9A	93.1(4)
C1	N3	C7A	C10A	-29.4(4)
C1	N3	C7B	C8B	-163.0(9)
C1	N3	C7B	C9B	79.3(7)
C1	N3	C7B	C10B	-40.3(7)
C2	N2	C5	C6	0.40(19)
C2	N2	B1	C15	-161.1(2)
C2	N2	B1	C22	76.1(2)
C2	N4	C6	C5	-0.1(2)
C2	N4	C11	C12	-9.5(3)
C2	N4	C11	C13	-130.4(3)
C2	N4	C11	C14	110.3(3)
C3	N1	B1	C15	-35.9(2)
C3	N1	B1	C22	87.8(2)
C3	C4	N3	C7A	170.7(3)
C3	C4	N3	C7B	-170.2(5)
C4	N3	C7A	C8A	46.4(4)
C4	N3	C7A	C9A	-75.7(3)
C4	N3	C7A	C10A	161.8(4)
C4	N3	C7B	C8B	5.4(7)
C4	N3	C7B	C9B	-112.3(7)
C4	N3	C7B	C10B	128.1(7)
C4	C3	N1	B1	-173.7(2)
C5	N2	B1	C15	34.15(19)
C5	N2	B1	C22	-88.63(19)
C5	C6	N4	C11	178.55(18)
C6	N4	C11	C12	172.1(3)
C6	N4	C11	C13	51.2(2)
C6	N4	C11	C14	-68.1(2)
C6	C5	N2	B1	167.93(17)
C15	C16	C17	C18	-0.7(2)
C15	C20	C19	C18	-0.6(2)
C15	B1	C22	C23	-108.40(18)
C15	B1	C22	C27	68.6(2)
C16	C15	C20	C19	1.6(2)
C16	C15	B1	C22	8.7(2)
C16	C17	C18	C19	1.7(3)
C16	C17	C18	C21	-175.6(2)
C17	C16	C15	C20	-0.9(2)
C17	C16	C15	B1	173.10(18)
C17	C18	C19	C20	-1.1(3)
C19	C20	C15	B1	-172.72(19)
C20	C15	B1	C22	-177.42(18)
C20	C19	C18	C21	176.2(2)
C22	C23	C24	C25	-0.9(3)
C22	C27	C26	C25	-0.8(3)

Atom	Atom	Atom	Atom	Angle/°
C23	C22	C27	C26	1.1(2)
C23	C24	C25	C26	1.1(3)
C23	C24	C25	C28	-180.0(2)
C24	C23	C22	C27	-0.2(3)
C24	C23	C22	B1	176.9(2)
C24	C25	C26	C27	-0.3(3)
C26	C27	C22	B1	-176.1(2)
C27	C26	C25	C28	-179.2(2)
C29	N5	C31	C32	-1.1(2)
C29	N5	B2	C43	149.0(2)
C29	N5	B2	C50	-84.6(2)
C29	N7	C32	C31	1.2(2)
C29	N7	C35	C36	-133.7(3)
C29	N7	C35	C37	106.2(2)
C29	N7	C35	C38	-13.8(2)
C30	N6	C33	C34	0.28(19)
C30	N6	B2	C43	-160.4(3)
C30	N6	B2	C50	75.3(2)
C30	N8	C34	C33	-0.20(19)
C30	N8	C39	C40	107.7(3)
C30	N8	C39	C41	-15.8(3)
C30	N8	C39	C42	-133.7(3)
C31	N5	B2	C43	-38.4(2)
C31	N5	B2	C50	87.9(2)
C31	C32	N7	C35	178.61(19)
C32	N7	C35	C36	49.5(3)
C32	N7	C35	C37	-70.6(2)
C32	N7	C35	C38	169.3(2)
C32	C31	N5	B2	-175.1(2)
C33	N6	B2	C43	33.27(19)
C33	N6	B2	C50	-91.0(2)
C33	C34	N8	C39	-179.96(18)
C34	N8	C39	C40	-72.6(2)
C34	N8	C39	C41	163.9(3)
C34	N8	C39	C42	46.0(2)
C34	C33	N6	B2	169.22(18)
C43	C44	C45	C46	-0.7(3)
C43	C48	C47	C46	-0.9(3)
C43	B2	C50	C51	-99.5(2)
C43	B2	C50	C55	77.7(2)
C44	C43	C48	C47	-3.1(2)
C44	C43	B2	C50	-179.5(2)
C44	C45	C46	C47	-3.4(3)
C44	C45	C46	C49	176.0(2)
C45	C44	C43	C48	3.9(3)
C45	C44	C43	B2	-169.9(2)
C45	C46	C47	C48	4.2(3)
C47	C48	C43	B2	170.2(2)
C48	C43	B2	C50	7.3(2)
C48	C47	C46	C49	-175.1(2)
C50	C51	C52	C53	-0.6(4)
C50	C55	C54	C53	0.3(3)
C51	C50	C55	C54	0.4(3)
C51	C52	C53	C54	1.3(4)
C51	C52	C53	C56	179.5(3)

Atom	Atom	Atom	Atom	Angle/°
C52	C51	C50	C55	-0.2(4)
C52	C51	C50	B2	177.1(3)
C52	C53	C54	C55	-1.2(3)
C54	C55	C50	B2	-177.0(2)
C55	C54	C53	C56	-179.4(3)
C7A	N3	C7B	C8B	90(2)
C7A	N3	C7B	C9B	-28(2)
C7A	N3	C7B	C10B	-147.6(18)
C8A	C7A	N3	C7B	-59.6(14)
C9A	C7A	N3	C7B	178.3(12)
C10A	C7A	N3	C7B	55.8(13)
In11	C101	N101	C103	175.93(16)
In11	C101	N101	B101	-13.5(2)
In11	C101	N103	C104	-175.2(2)
In11	C101	N103	C107	3.5(3)
In11	C102	N102	C105	179.22(15)
In11	C102	N102	B101	8.90(19)
In11	C102	N104	C106	179.9(2)
In11	C102	N104	C111	2.1(2)
In12	C129	N105	C131	-176.38(17)
In12	C129	N105	B102	14.2(2)
In12	C129	N107	C132	175.2(2)
In12	C129	N107	C135	-6.8(3)
In12	C130	N106	C133	-178.38(17)
In12	C130	N106	B102	-1.6(2)
In12	C130	N108	C134	178.7(2)
In12	C130	N108	C139	-9.4(3)
N101	C101	N103	C104	-0.2(2)
N101	C101	N103	C107	178.51(18)
N101	C103	C104	N103	-0.2(2)
N101	B101	N102	C102	-48.1(2)
N101	B101	N102	C105	142.67(17)
N101	B101	C115	C116	110.34(17)
N101	B101	C115	C120	-63.80(18)
N101	B101	C122	C123	-10.3(2)
N101	B101	C122	C127	174.53(18)
N102	C102	N104	C106	0.77(19)
N102	C102	N104	C111	-177.03(15)
N102	C105	C106	N104	-1.1(2)
N102	B101	N101	C101	51.1(2)
N102	B101	N101	C103	-139.44(17)
N102	B101	C115	C116	-131.57(16)
N102	B101	C115	C120	54.29(18)
N102	B101	C122	C123	-130.98(17)
N102	B101	C122	C127	53.9(2)
N103	C101	N101	C103	0.1(2)
N103	C101	N101	B101	170.68(15)
N104	C102	N102	C105	-1.46(19)
N104	C102	N102	B101	-171.78(14)
N105	C129	N107	C132	0.4(2)
N105	C129	N107	C135	178.48(16)
N105	C131	C132	N107	-0.5(2)
N105	B102	N106	C130	40.6(2)
N105	B102	N106	C133	-142.96(18)
N105	B102	C143	C144	53.48(19)

Atom	Atom	Atom	Atom	Angle/°
N105	B102	C143	C148	-131.98(17)
N105	B102	C150	C151	-134.47(17)
N105	B102	C150	C155	49.1(2)
N106	C130	N108	C134	-0.3(2)
N106	C130	N108	C139	171.64(18)
N106	C133	C134	N108	0.8(2)
N106	B102	N105	C129	-48.2(2)
N106	B102	N105	C131	143.60(19)
N106	B102	C143	C144	-65.41(19)
N106	B102	C143	C148	109.13(18)
N106	B102	C150	C151	-11.6(2)
N106	B102	C150	C155	172.03(17)
N107	C129	N105	C131	-0.7(2)
N107	C129	N105	B102	-170.12(16)
N108	C130	N106	C133	0.8(2)
N108	C130	N106	B102	177.55(16)
C101	N101	C103	C104	0.07(19)
C101	N101	B101	C115	168.2(2)
C101	N101	B101	C122	-68.5(2)
C101	N103	C104	C103	0.2(2)
C101	N103	C107	C108	-107.2(3)
C101	N103	C107	C109	133.6(3)
C101	N103	C107	C110	17.8(3)
C102	N102	C105	C106	1.62(19)
C102	N102	B101	C115	-163.3(2)
C102	N102	B101	C122	72.7(2)
C102	N104	C106	C105	0.22(19)
C102	N104	C111	C112	112.8(2)
C102	N104	C111	C113	-7.4(2)
C102	N104	C111	C114	-126.8(3)
C103	N101	B101	C115	-22.4(2)
C103	N101	B101	C122	100.9(2)
C103	C104	N103	C107	-178.60(19)
C104	N103	C107	C108	71.3(3)
C104	N103	C107	C109	-47.8(2)
C104	N103	C107	C110	-163.6(3)
C104	C103	N101	B101	-171.20(18)
C105	N102	B101	C115	27.42(19)
C105	N102	B101	C122	-96.58(19)
C105	C106	N104	C111	178.25(18)
C106	N104	C111	C112	-64.8(2)
C106	N104	C111	C113	175.0(2)
C106	N104	C111	C114	55.6(2)
C106	C105	N102	B101	172.76(17)
C115	C116	C117	C118	0.5(2)
C115	C120	C119	C118	-0.1(2)
C115	B101	C122	C123	108.15(19)
C115	B101	C122	C127	-67.0(2)
C116	C115	C120	C119	-0.9(2)
C116	C115	B101	C122	-10.8(2)
C116	C117	C118	C119	-1.5(3)
C116	C117	C118	C121	177.7(2)
C117	C116	C115	C120	0.7(2)
C117	C116	C115	B101	-173.67(19)
C117	C118	C119	C120	1.3(2)

Atom	Atom	Atom	Atom	Angle/°
C119	C120	C115	B101	173.69(19)
C120	C115	B101	C122	175.08(19)
C120	C119	C118	C121	-177.8(2)
C122	C123	C124	C125	0.1(3)
C122	C127	C126	C125	-0.6(3)
C123	C122	C127	C126	1.3(3)
C123	C124	C125	C126	0.7(3)
C123	C124	C125	C128	-179.2(3)
C124	C123	C122	C127	-1.1(3)
C124	C123	C122	B101	-176.4(2)
C124	C125	C126	C127	-0.4(3)
C126	C127	C122	B101	176.8(2)
C127	C126	C125	C128	179.4(3)
C129	N105	C131	C132	0.7(2)
C129	N105	B102	C143	-163.0(2)
C129	N105	B102	C150	74.5(2)
C129	N107	C132	C131	0.1(2)
C129	N107	C135	C136	120.7(3)
C129	N107	C135	C137	0.9(3)
C129	N107	C135	C138	-119.4(3)
C130	N106	C133	C134	-1.0(2)
C130	N106	B102	C143	157.3(2)
C130	N106	B102	C150	-80.9(2)
C130	N108	C134	C133	-0.4(2)
C130	N108	C139	C140	-91.1(3)
C130	N108	C139	C141	150.1(3)
C130	N108	C139	C142	29.4(3)
C131	N105	B102	C143	28.8(2)
C131	N105	B102	C150	-93.8(2)
C131	C132	N107	C135	-178.2(2)
C132	N107	C135	C136	-61.4(2)
C132	N107	C135	C137	178.8(2)
C132	N107	C135	C138	58.5(3)
C132	C131	N105	B102	171.1(2)
C133	N106	B102	C143	-26.3(2)
C133	N106	B102	C150	95.5(2)
C133	C134	N108	C139	-172.9(2)
C134	N108	C139	C140	79.9(3)
C134	N108	C139	C141	-38.8(3)
C134	N108	C139	C142	-159.6(3)
C134	C133	N106	B102	-178.11(18)
C143	C144	C145	C146	0.7(3)
C143	C148	C147	C146	-0.9(2)
C143	B102	C150	C151	105.74(19)
C143	B102	C150	C155	-70.6(2)
C144	C143	C148	C147	2.2(2)
C144	C143	B102	C150	173.84(19)
C144	C145	C146	C147	0.6(3)
C144	C145	C146	C149	-176.6(2)
C145	C144	C143	C148	-2.1(3)
C145	C144	C143	B102	172.9(2)
C145	C146	C147	C148	-0.5(3)
C147	C148	C143	B102	-172.54(19)
C148	C143	B102	C150	-11.6(2)
C148	C147	C146	C149	176.6(2)

Atom	Atom	Atom	Atom	Angle/°
C150	C151	C152	C153	0.7(3)
C150	C155	C154	C153	1.7(3)
C151	C150	C155	C154	-2.7(2)
C151	C152	C153	C154	-1.8(3)
C151	C152	C153	C156	176.2(3)
C152	C151	C150	C155	1.5(3)
C152	C151	C150	B102	-175.0(2)
C152	C153	C154	C155	0.6(3)
C154	C155	C150	B102	174.0(2)
C155	C154	C153	C156	-177.4(3)

Table S41. Hydrogen Fractional Atomic Coordinates (×10^4) and Equivalent Isotropic Displacement Parameters (Å^2×10^3) for **3-F**. U_{eq} is defined as 1/3 of the trace of the orthogonalised U_{ij}.

Atom	x	y	z	U_{eq}
H3	-1664(3)	5666.6(13)	128.8(9)	40.4(6)
H4	-1556(3)	7013.9(13)	181.4(9)	48.6(8)
H5	-2770(2)	4183.7(10)	1269.7(7)	30.4(5)
H6	-3185(2)	4378.0(11)	2234.1(8)	28.9(5)
H12a	-2234(12)	6897(2)	2983(5)	74.5(15)
H12b	-1825(17)	6742(4)	3577.7(18)	74.5(15)
H12c	-780(5)	6596(2)	3107(7)	74.5(15)
H13a	-1959(14)	5552(5)	3608.6(14)	55.9(9)
H13b	-2425(9)	4792(2)	3033(4)	55.9(9)
H13c	-895(5)	5343(7)	3150(5)	55.9(9)
H14a	-4007(3)	5991(8)	3349.2(12)	67.2(10)
H14b	-4277(4)	6064(7)	2719(5)	67.2(10)
H14c	-4365(4)	5205.7(19)	2789(6)	67.2(10)
H16	-844(2)	4094.2(11)	391.2(8)	41.8(6)
H17	-2332(3)	3098.0(13)	-294.1(9)	51.0(8)
H19	-5383(2)	4260.2(14)	7.1(8)	50.0(7)
H20	-3913(2)	5256.0(12)	671.8(7)	40.5(6)
H21a	-4343(4)	2582(5)	-770(6)	103.7(16)
H21b	-5532(14)	2747(8)	-363.2(13)	103.7(16)
H21c	-5416(15)	3161(2)	-845(5)	103.7(16)
H23	50(2)	5046.2(12)	1870.2(9)	39.4(6)
H24	2279(2)	4879.1(13)	1965.0(10)	46.7(6)
H26	2727(2)	5351.4(12)	574.8(9)	41.1(6)
H27	514(2)	5548.6(12)	491.7(8)	37.9(6)
H28a	4275(5)	4871(9)	1681(4)	73.4(10)
H28b	4146(4)	4544(6)	992(3)	73.4(10)
H28c	4654(3)	5475(3)	1340(7)	73.4(10)
H31	2140(3)	8579.2(15)	4861.1(9)	48.9(7)
H32	147(2)	9206.3(14)	4786.9(8)	49.8(7)
H33	5480(2)	7761.1(11)	3730.9(8)	32.7(5)
H34	5877(2)	7518.3(12)	2766.1(8)	31.7(5)
H36a	-890(20)	9883.9(17)	4476(2)	99.5(15)
H36b	92(9)	10110(2)	4047(7)	99.5(15)
H36c	-1598(12)	10028(3)	3910(5)	99.5(15)
H37a	-2229(11)	8047(3)	3592(5)	81.8(11)
H37b	-1957(8)	8602(8)	4257(2)	81.8(11)
H37c	-2941(4)	8823(6)	3788(7)	81.8(11)
H38a	-102(5)	9170(9)	3121.9(16)	65.1(9)
H38b	-1277(17)	8381.5(16)	2963.7(10)	65.1(9)

Atom	x	y	z	U_{eq}
H38c	-1752(12)	9231(8)	3088.9(18)	65.1(9)
H40a	3873(12)	8552(3)	2158(8)	96.5(16)
H40b	5354(7)	8243(7)	2190(7)	96.5(16)
H40c	4355(19)	8023(4)	1580.0(11)	96.5(16)
H41a	1845(6)	6808(7)	1927(6)	92.8(15)
H41b	1859(5)	7695(3)	1922(6)	92.8(15)
H41c	2277(3)	7039(10)	1386.4(10)	92.8(15)
H42a	5389(3)	6980(3)	1949(5)	52.3(8)
H42b	4016(11)	6368(3)	1948(5)	52.3(8)
H42c	4148(12)	6650(5)	1401.2(8)	52.3(8)
H44	4111(2)	9171.4(13)	4241.6(8)	49.4(7)
H45	6091(2)	9794.4(16)	4844.6(9)	60.1(8)
H47	6220(2)	7958.2(17)	5307.5(8)	59.9(9)
H48	4250(2)	7316.0(15)	4683.0(8)	51.0(7)
H49a	7949(12)	8929(5)	5636(7)	126.9(19)
H49b	8274(8)	9413(12)	5208.1(17)	126.9(19)
H49c	7449(6)	9768(7)	5734(6)	126.9(19)
H51	3305(3)	6464.5(14)	3240.6(11)	61.4(9)
H52	2469(3)	5231.0(15)	3175.9(13)	72.5(10)
H54	350(2)	6011.8(16)	4403.4(9)	52.6(8)
H55	1177(2)	7250.3(15)	4467.3(9)	48.1(7)
H56a	1305(15)	4666(4)	4087(5)	102.9(14)
H56b	-186(4)	4722(3)	3760(8)	102.9(14)
H56c	1021(18)	4402.3(17)	3397(4)	102.9(14)
H8Aa	-1702(18)	8120(15)	459(5)	93(4)
H8Ab	-2976(9)	7996(12)	806(9)	93(4)
H8Ac	-1920(20)	8836(5)	1006(7)	93(4)
H9Aa	872(4)	8124(11)	1474(7)	73.6(17)
H9Ab	495(7)	8045(10)	810(3)	73.6(17)
H9Ac	425(6)	8857(2)	1307(9)	73.6(17)
H10a	-2388(12)	8036(10)	1754(3)	79(2)
H10b	-737(15)	8235(13)	2024.7(19)	79(2)
H10c	-1460(30)	8880(4)	1829(5)	79(2)
H8Ba	-1150(30)	8150(30)	515(13)	39(4)
H8Bb	-2790(30)	7900(20)	551(13)	39(4)
H8Bc	-1970(50)	8791(12)	897(7)	39(4)
H9Ba	150(30)	8160(30)	1790(20)	78(5)
H9Bb	491(16)	8310(30)	1210(7)	78(5)
H9Bc	-146(18)	8957(8)	1670(30)	78(5)
H10d	-3391(13)	7960(30)	1397(7)	68(4)
H10e	-2210(40)	7990(30)	1912(14)	68(4)
H10f	-2330(50)	8777(5)	1760(20)	68(4)
H103	-911(2)	7330.5(13)	6345.8(8)	36.3(6)
H104	-1305(2)	7307.2(13)	7270.4(8)	34.8(5)
H105	2197(2)	6615.5(12)	5145.4(8)	38.4(6)
H106	4132(2)	5918.1(13)	5124.4(8)	40.0(6)
H10g	-745(7)	6413(5)	7708(8)	115.3(17)
H10h	744(14)	6114(2)	7662(8)	115.3(17)
H10i	290(20)	6490(4)	8281.6(15)	115.3(17)
H10j	543(13)	8333(3)	8187(6)	85.0(13)
H10k	-832(3)	7668(5)	8092(5)	85.0(13)
H10l	379(15)	7875(8)	8641.8(14)	85.0(13)
H11a	2780(5)	7032(6)	7978(5)	91.7(16)
H11b	2681(6)	7956(5)	8156(7)	91.7(16)
H11c	2306(3)	7475(11)	8581(2)	91.7(16)

Atom	x	y	z	U_{eq}
H11d	4117(12)	5062.8(16)	6083(6)	87.5(12)
H11e	4638(19)	5019.7(19)	5482.6(14)	87.5(12)
H11f	5707(6)	4941.4(15)	5996(7)	87.5(12)
H11g	6100(16)	6825.4(15)	6834.0(15)	59.4(9)
H11h	5082(6)	6069(8)	6872.4(12)	59.4(9)
H11i	6671(10)	6008(8)	6750.7(9)	59.4(9)
H11j	6179(9)	5995(9)	5420.7(12)	95.9(13)
H11k	6643(14)	6815(2)	5950(7)	95.9(13)
H11l	7313(6)	6053(10)	5963(7)	95.9(13)
H116	622(2)	8284.6(12)	5643.4(8)	38.3(5)
H117	-1295(2)	7964.1(13)	4973.0(8)	44.5(6)
H119	-1748(2)	5794.2(13)	4934.3(8)	39.2(6)
H120	169(2)	6109.1(12)	5599.5(7)	35.3(5)
H12d	-3697(2)	6644(9)	4717.3(14)	74.1(10)
H12e	-2878(8)	7083(5)	4342(5)	74.1(10)
H12f	-2885(8)	6157(4)	4228(4)	74.1(10)
H123	1855(2)	8646.0(12)	7058.7(8)	38.7(6)
H124	2968(2)	9878.4(12)	7261.9(9)	48.2(6)
H126	4403(2)	9259.4(14)	5818.7(10)	47.0(7)
H127	3273(2)	8031.3(13)	5617.6(9)	41.8(6)
H12g	3901(9)	10787.4(14)	6898(7)	93.4(12)
H12h	5391(8)	10489(3)	6930(7)	93.4(12)
H12i	4626(18)	10517(4)	6330.7(13)	93.4(12)
H131	6597(3)	9555.1(13)	9849.1(9)	40.3(7)
H132	6671(3)	8247.0(13)	9857.3(9)	40.8(7)
H133	7039(2)	10897.2(11)	8596.7(8)	40.6(6)
H134	7790(2)	10621.3(11)	7676.8(9)	36.0(6)
H13d	8159(4)	7220(8)	8858(4)	63.6(9)
H13e	7708(7)	7358(7)	9482.4(17)	63.6(9)
H13f	7397(5)	6482.0(19)	9012(6)	63.6(9)
H13g	4905(8)	6867(8)	8146.9(14)	64.7(10)
H13h	6563(10)	6996(7)	8102.2(11)	64.7(10)
H13i	5829(18)	6216.5(18)	8213.2(10)	64.7(10)
H13j	5352(10)	7187(7)	9550.8(13)	77.5(11)
H13k	4207(4)	7013(9)	8985(5)	77.5(11)
H13l	5061(13)	6335(2)	9052(6)	77.5(11)
H14d	9416(4)	9605.1(19)	7385(7)	106.0(17)
H14e	8921(4)	8769(9)	7468(6)	106.0(17)
H14f	9150(7)	8798(10)	6843.6(16)	106.0(17)
H14g	7941(16)	10122(4)	6945(4)	101.4(17)
H14h	7640(20)	9340(6)	6382(4)	101.4(17)
H14i	6344(5)	9663(10)	6682(7)	101.4(17)
H14j	6899(15)	8204(5)	6425(3)	78.7(15)
H14k	6861(15)	8054(3)	7028(4)	78.7(15)
H14l	5650(4)	8449(2)	6786(7)	78.7(15)
H144	8405(2)	10045.3(12)	9311.5(8)	41.1(6)
H145	9668(2)	11166.3(13)	9934.5(8)	47.1(6)
H147	6273(2)	12078.1(12)	10066.2(8)	46.7(6)
H148	5015(2)	10951.7(11)	9443.5(8)	39.8(6)
H14m	9641(10)	12280.1(14)	10634(5)	81.6(11)
H14n	9255(15)	12705(5)	10173.4(9)	81.6(11)
H14o	8272(5)	12698(5)	10655(5)	81.6(11)
H151	4174(2)	9853.2(13)	8034.9(9)	43.8(6)
H152	1870(3)	9861.3(15)	7970.3(10)	54.9(7)
H154	1850(3)	9463.7(13)	9404.6(9)	47.2(7)

Atom	x	y	z	U_{eq}
H155	4134(2)	9410.3(12)	9456.1(9)	39.2(6)
H15a	-249(3)	9215(3)	8635(8)	97.4(13)
H15b	177(4)	10138(7)	9021(4)	97.4(13)
H15c	-20(5)	9876(10)	8331(4)	97.4(13)

Table S42. Atomic Occupancies for all atoms that are not fully occupied in **3-F**.

**Atom**	**Occupancy**
C7A	0.736(7)
C8A	0.736(7)
H8Aa	0.736(7)
H8Ab	0.736(7)
H8Ac	0.736(7)
C9A	0.736(7)
H9Aa	0.736(7)
H9Ab	0.736(7)
H9Ac	0.736(7)
C10A	0.736(7)
H10a	0.736(7)
H10b	0.736(7)
H10c	0.736(7)
C7B	0.264(7)
C8B	0.264(7)
H8Ba	0.264(7)
H8Bb	0.264(7)
H8Bc	0.264(7)
C9B	0.264(7)
H9Ba	0.264(7)
H9Bb	0.264(7)
H9Bc	0.264(7)
C10B	0.264(7)
H10d	0.264(7)
H10e	0.264(7)
H10f	0.264(7)

8 NMR Characterisation Data

8.1 NMR characterisation of In(III) complexes 2-X

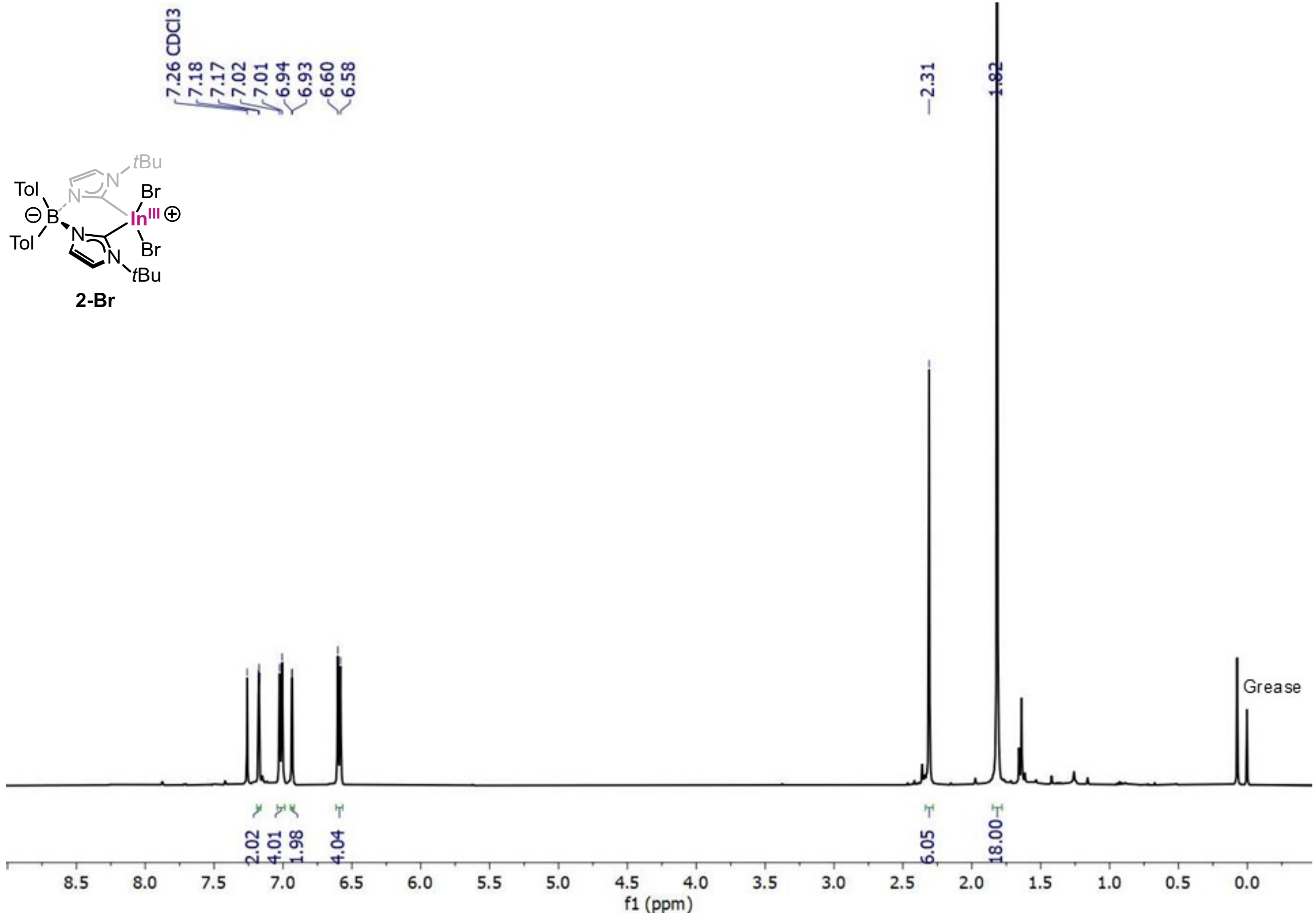

Figure S37. ^{1}H NMR ($CDCl_3$, 400 MHz) of **2-Br.**

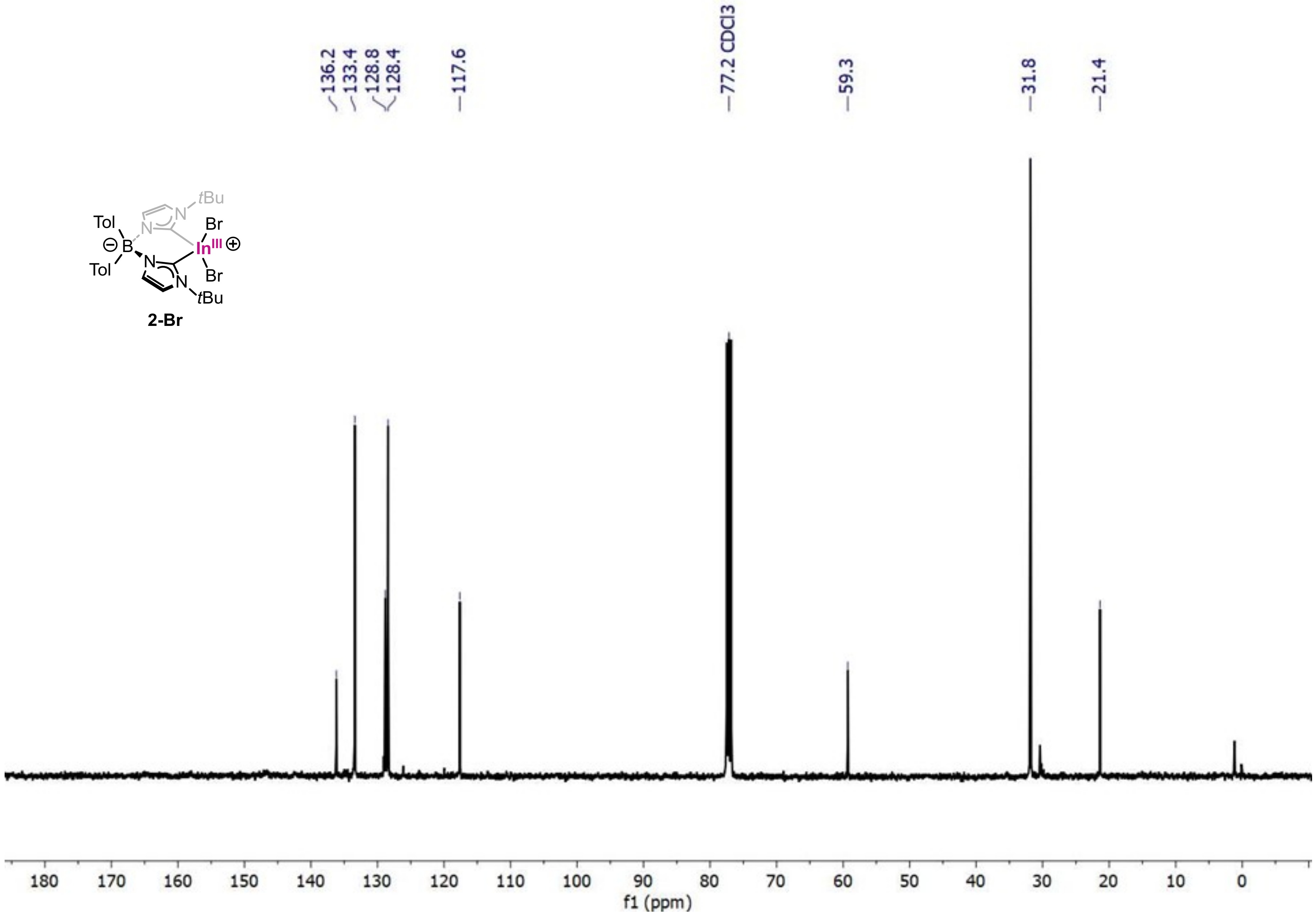

Figure S38. ^{13}C NMR ($CDCl_3$, 101 MHz) of **2-Br**.

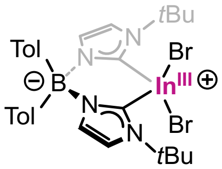

2-Br

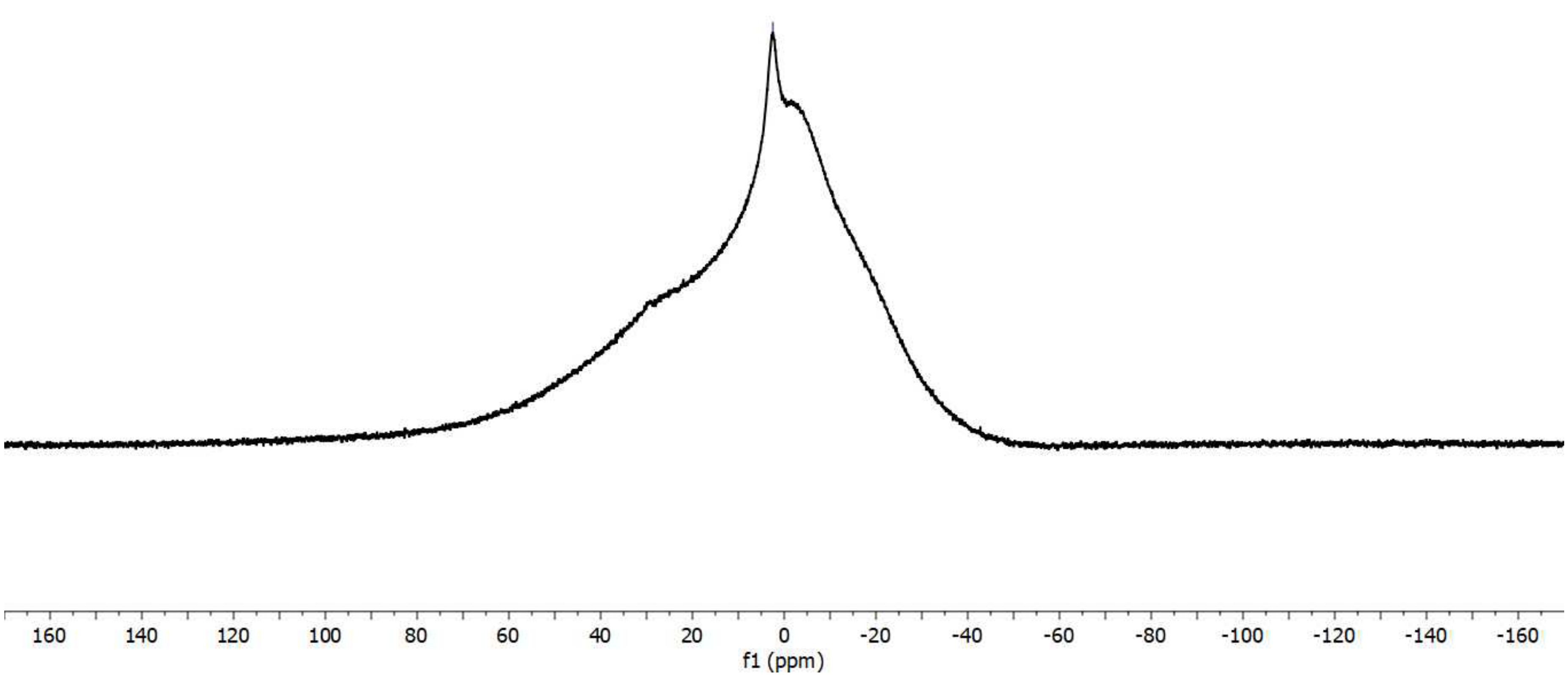

Figure S39. ^{11}B NMR ($CDCl_3$, 128 MHz) of **2-Br**.

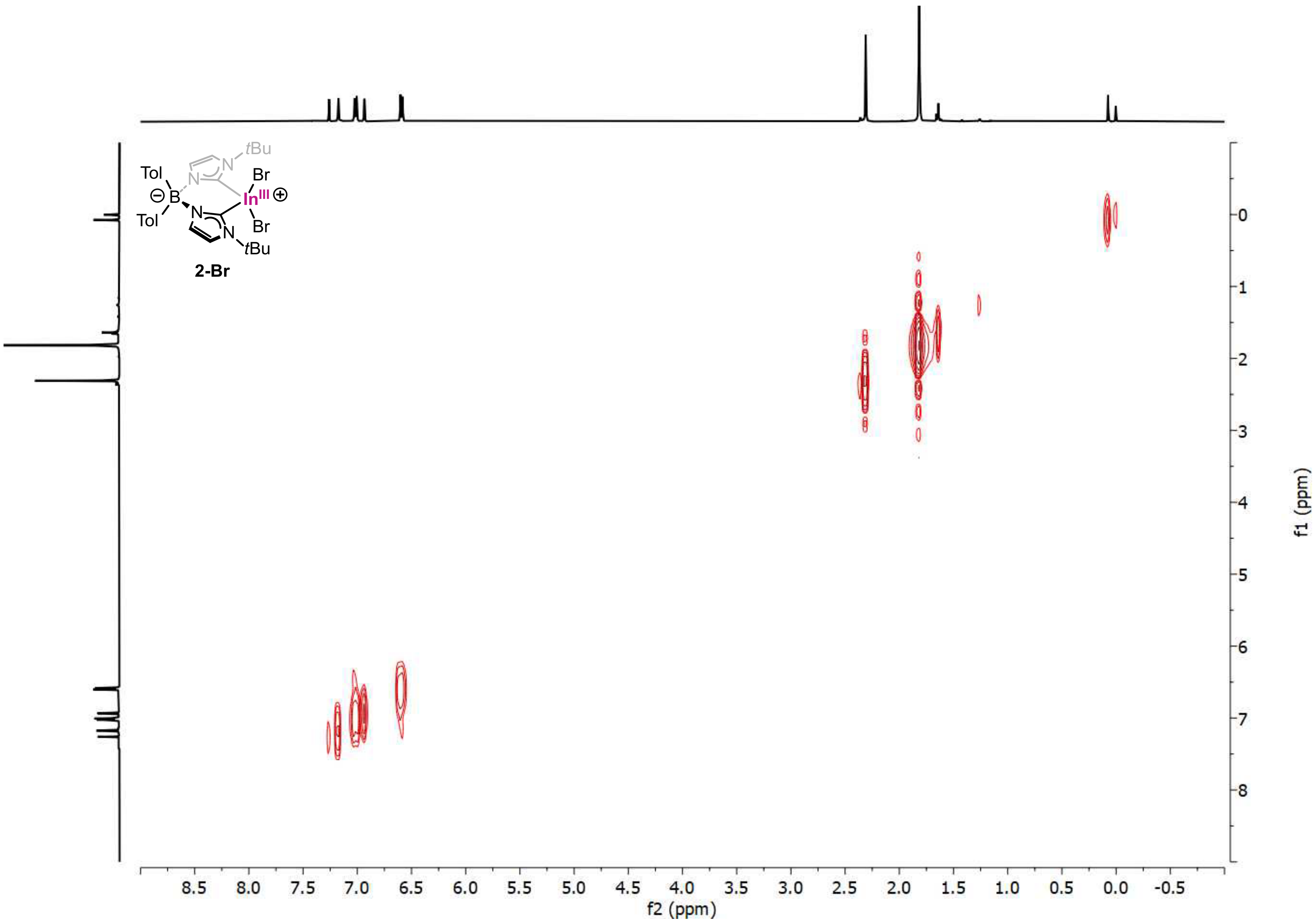

Figure S40. ^{1}H-^{1}H COSY NMR ($CDCl_3$, 400 MHz) of **2-Br**.

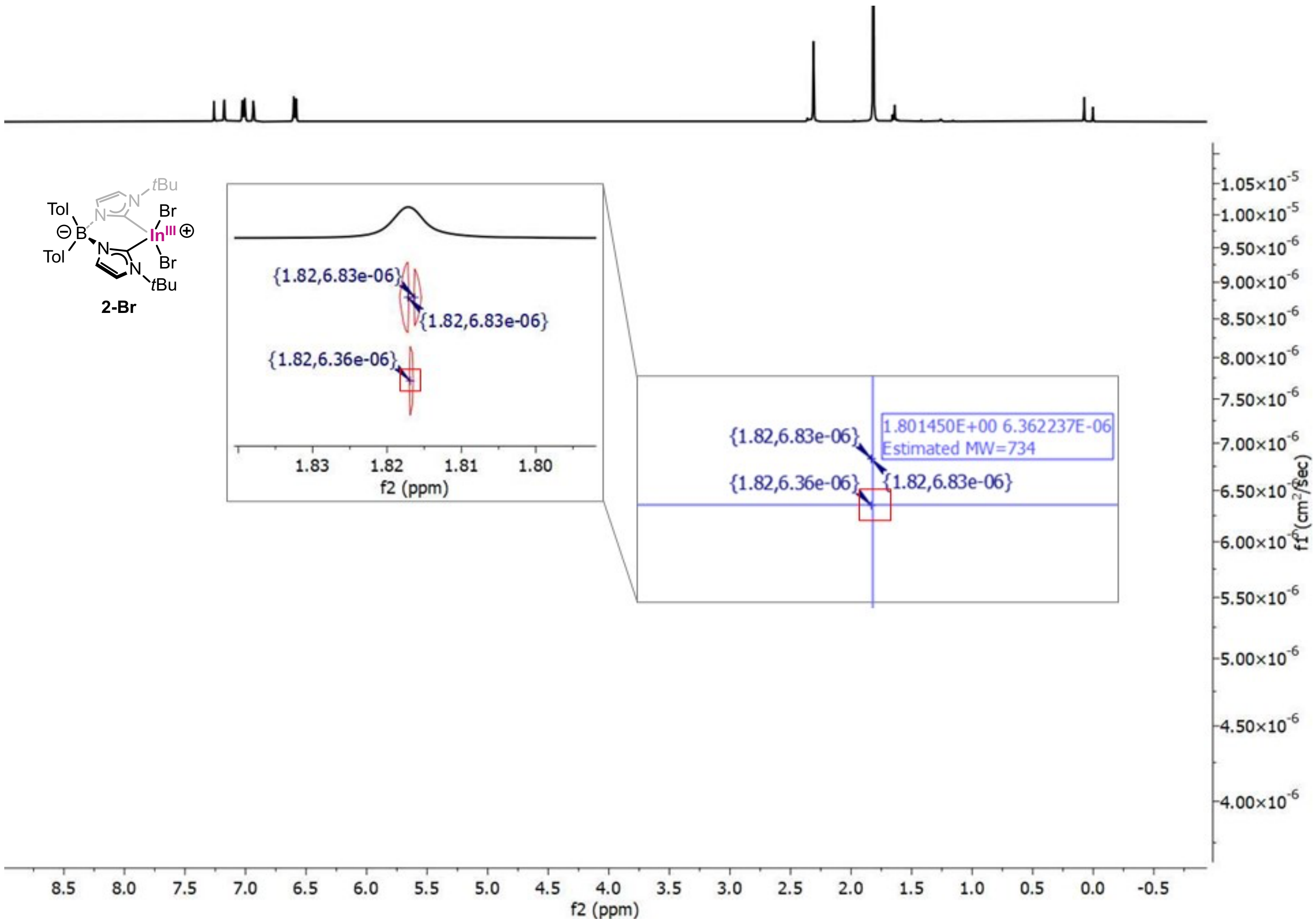

Figure S41. ^{1}H-^{1}H DOSY NMR ($CDCl_3$, 400 MHz) of **2-Br**.

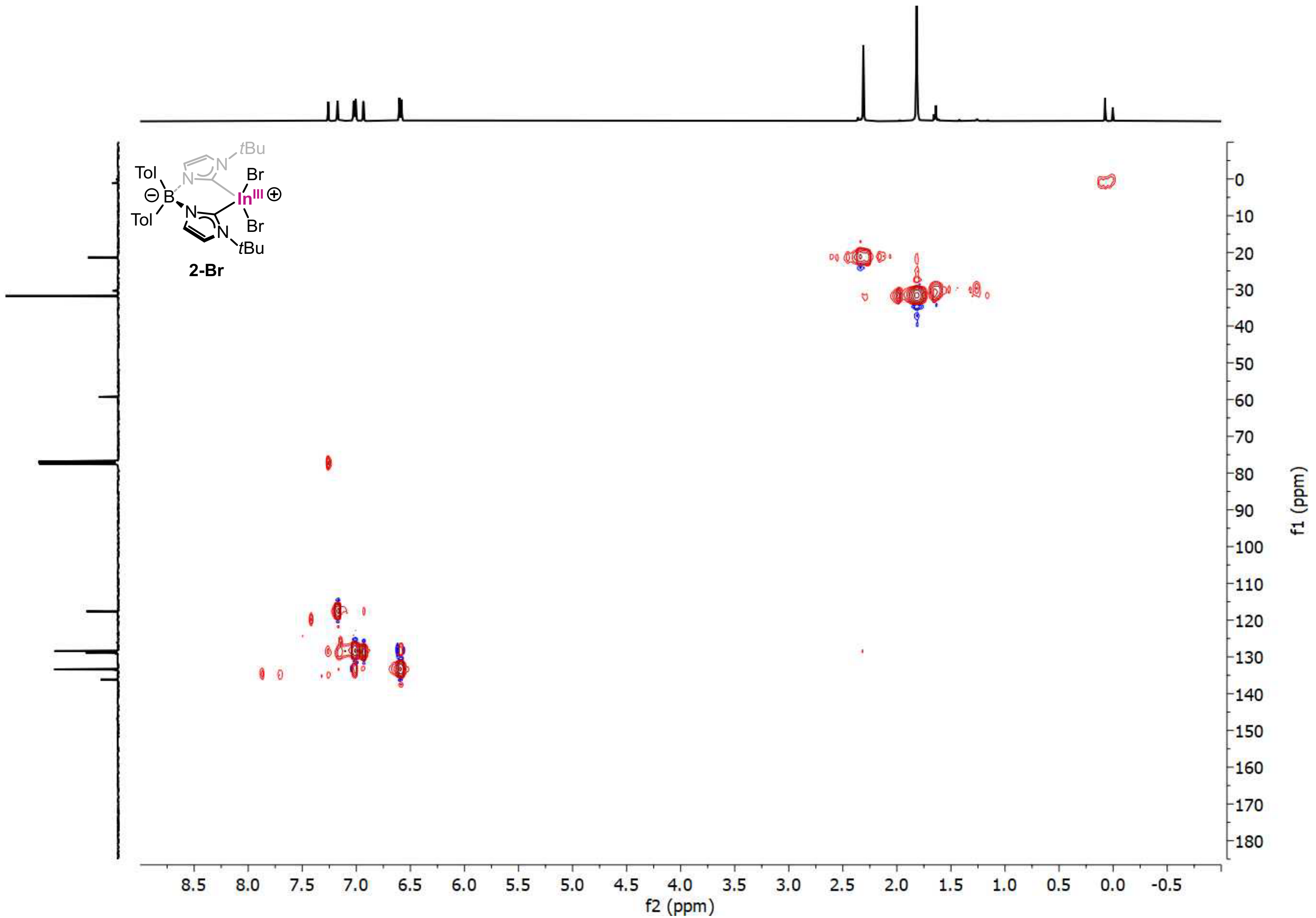

Figure S42. ^{1}H-^{13}C HSQC NMR ($CDCl_3$, 400 MHz) of **2-Br**.

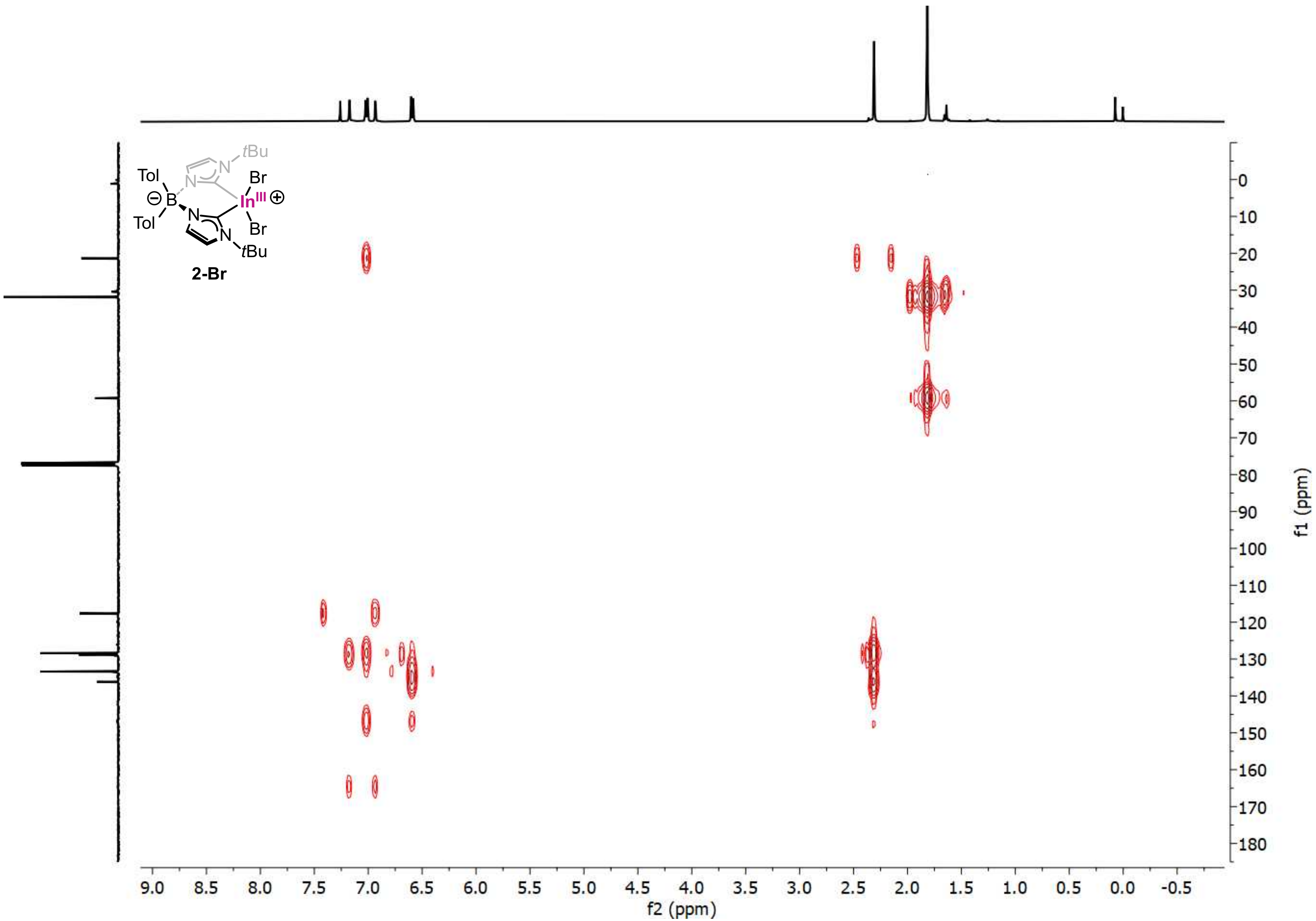

Figure S43. ^{1}H-^{13}C HMBC NMR ($CDCl_3$, 400 MHz) of **2-Br**.

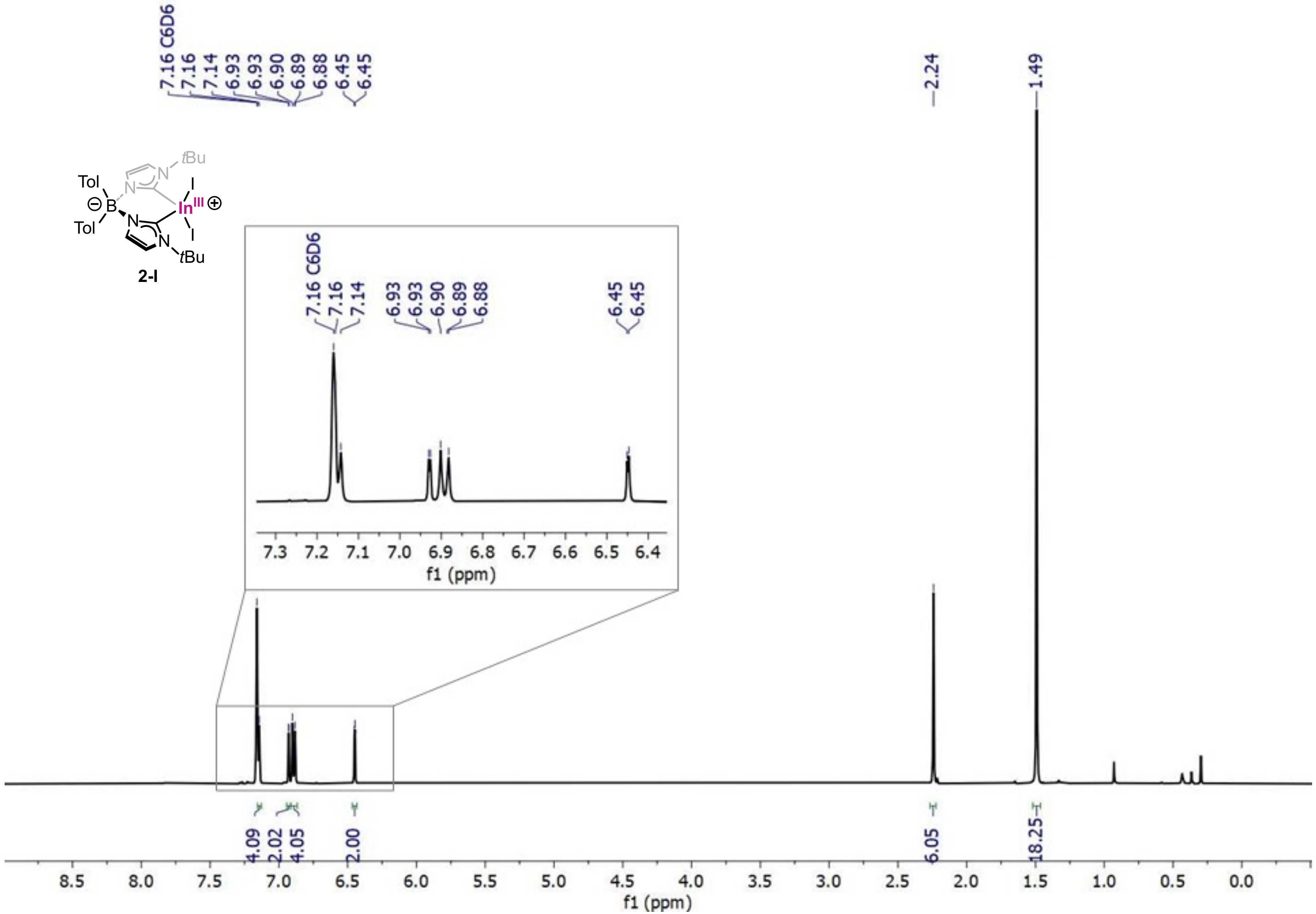

Figure S44. ^{1}H NMR (C_6D_6, 400 MHz, 298 K) of **2-I**.

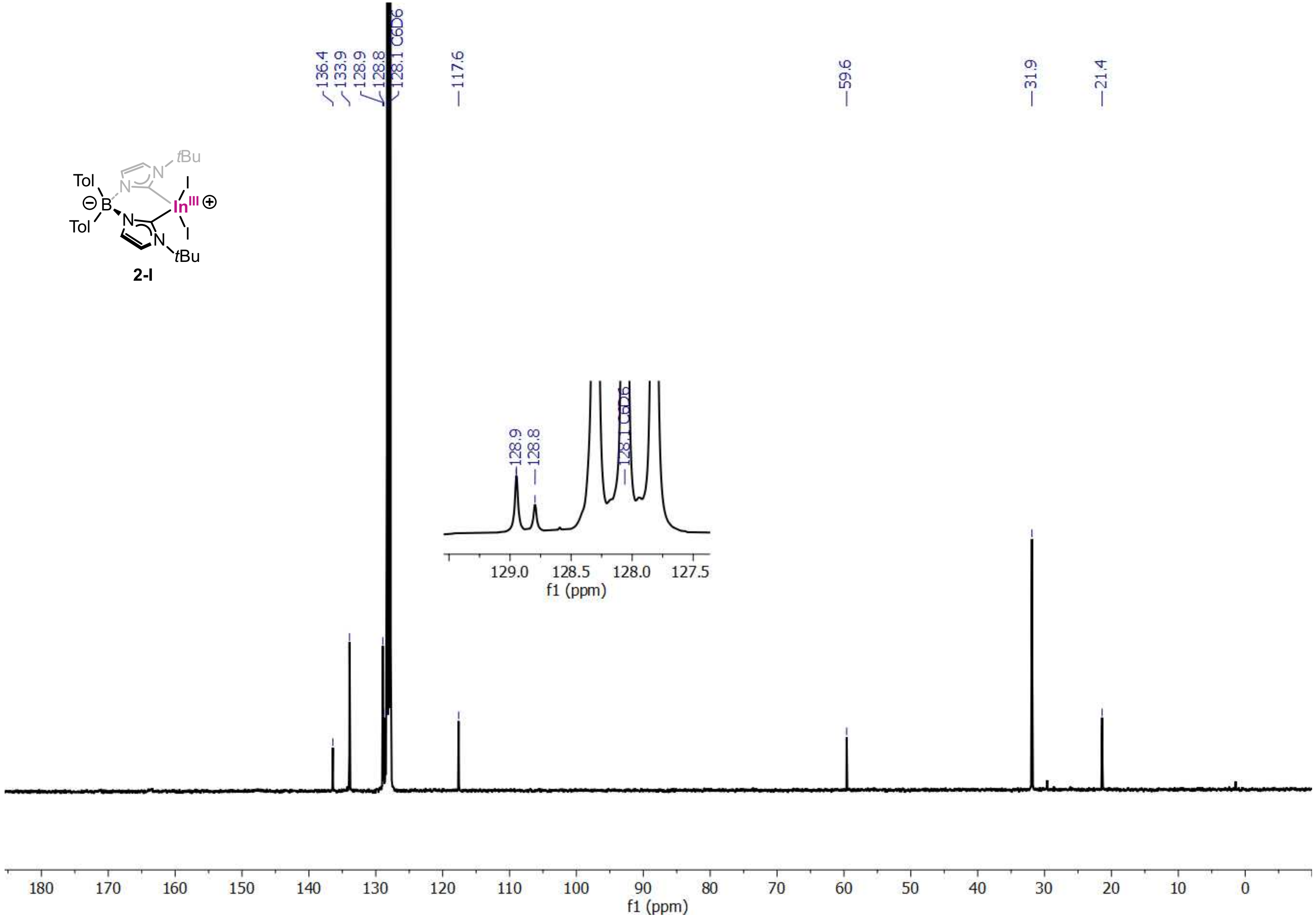

Figure S45. ^{13}C NMR (C_6D_6, 101 MHz, 298 K) of **2-I**.

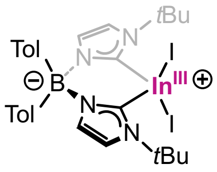

2-I

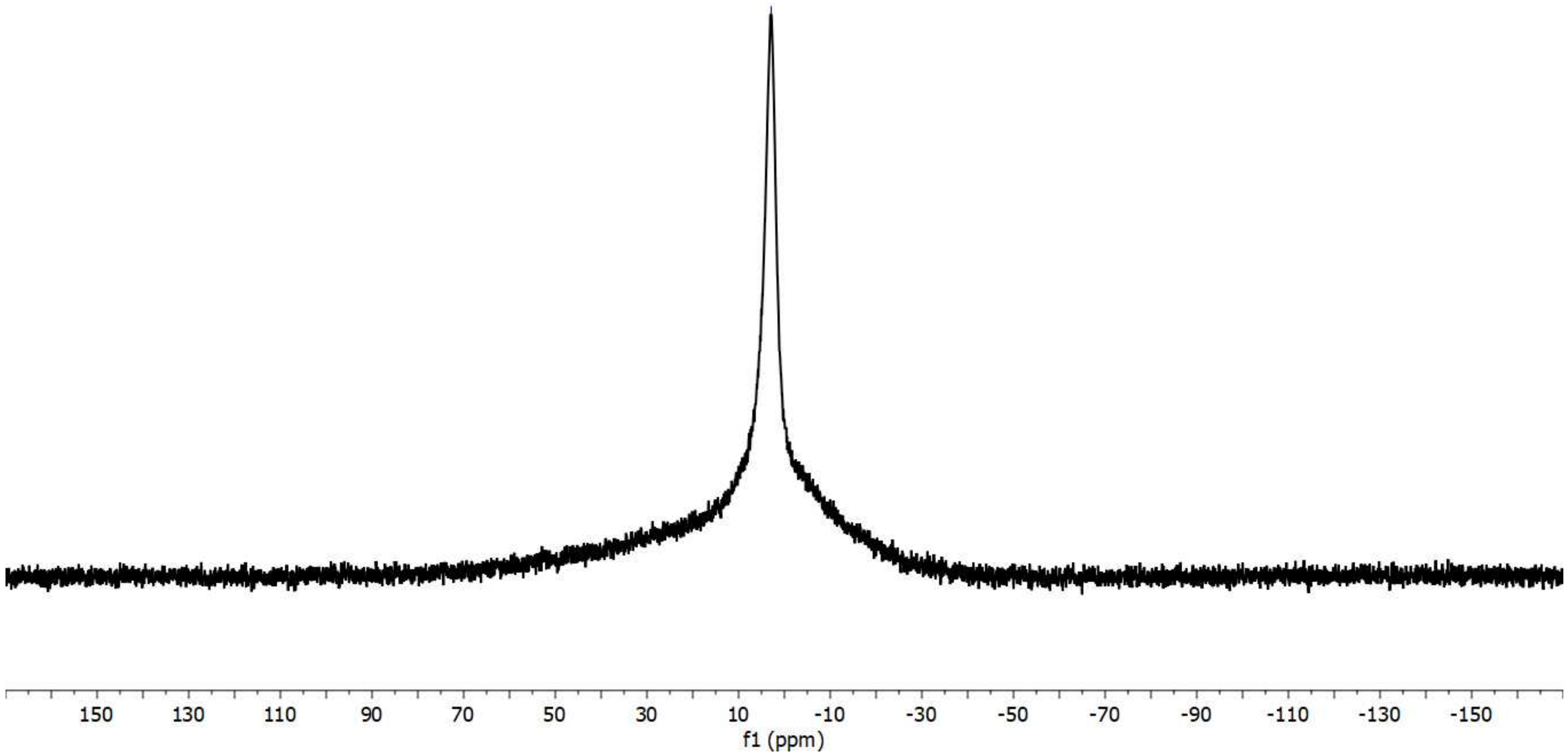

Figure S46. ^{11}B NMR (C_6D_6, 128 MHz, 298 K) of **2-I**.

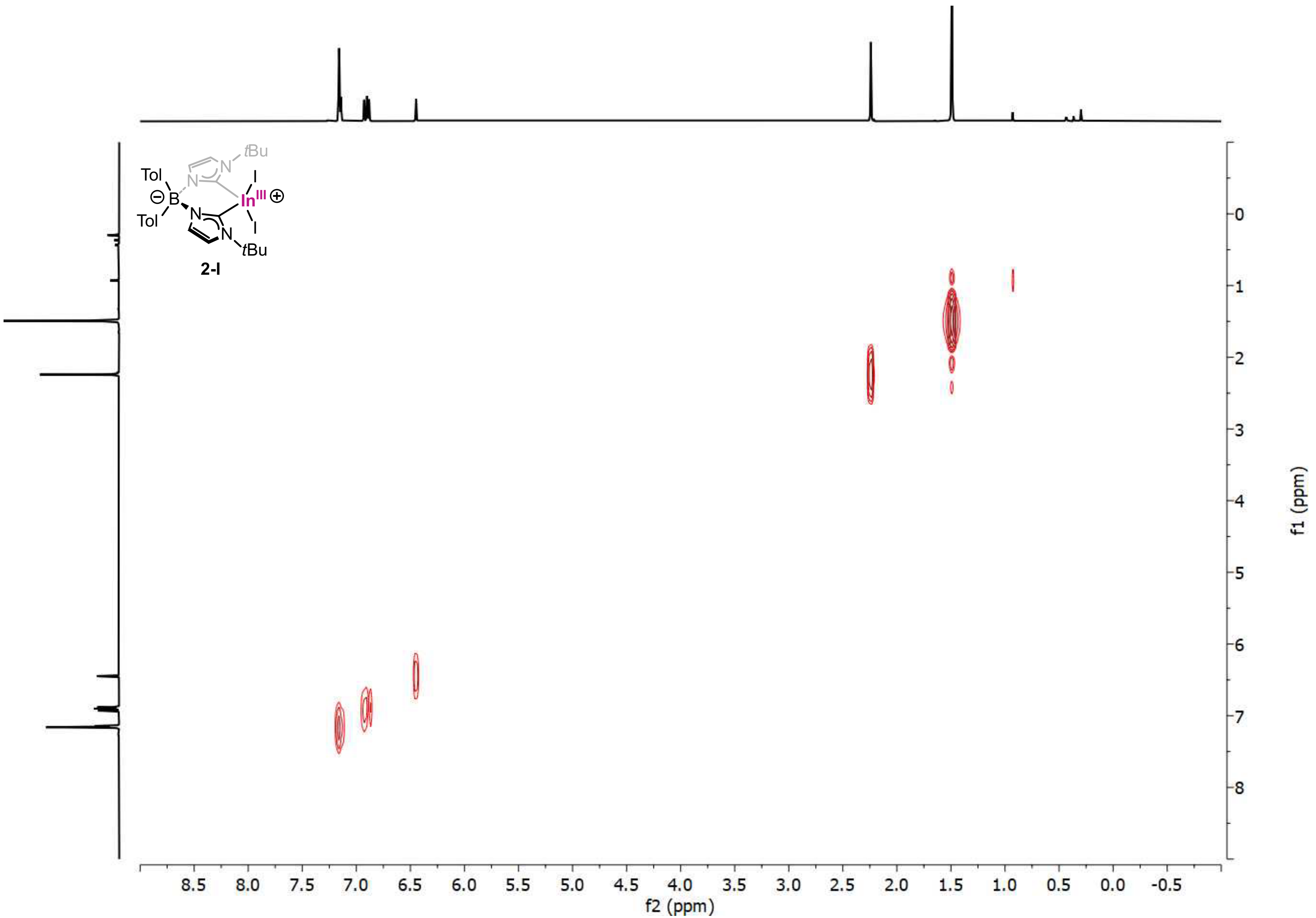

Figure S47. ^1H-^1H COSY NMR (C_6D_6, 400 MHz, 298 K) of **2-I**.

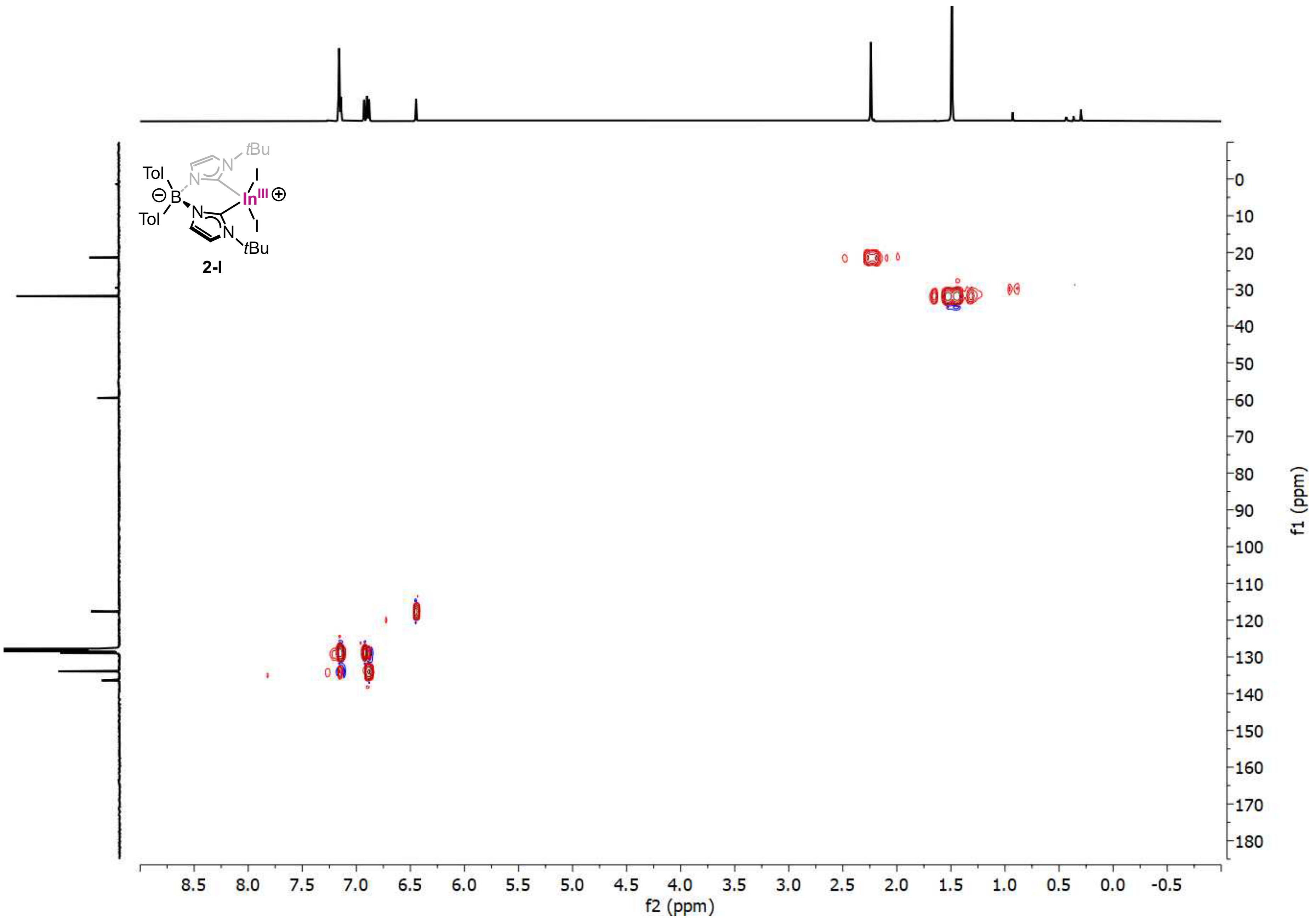

Figure S48. ^{1}H-^{13}C HSQC NMR (C_6D_6, 400 MHz, 298 K) of **2-I**.

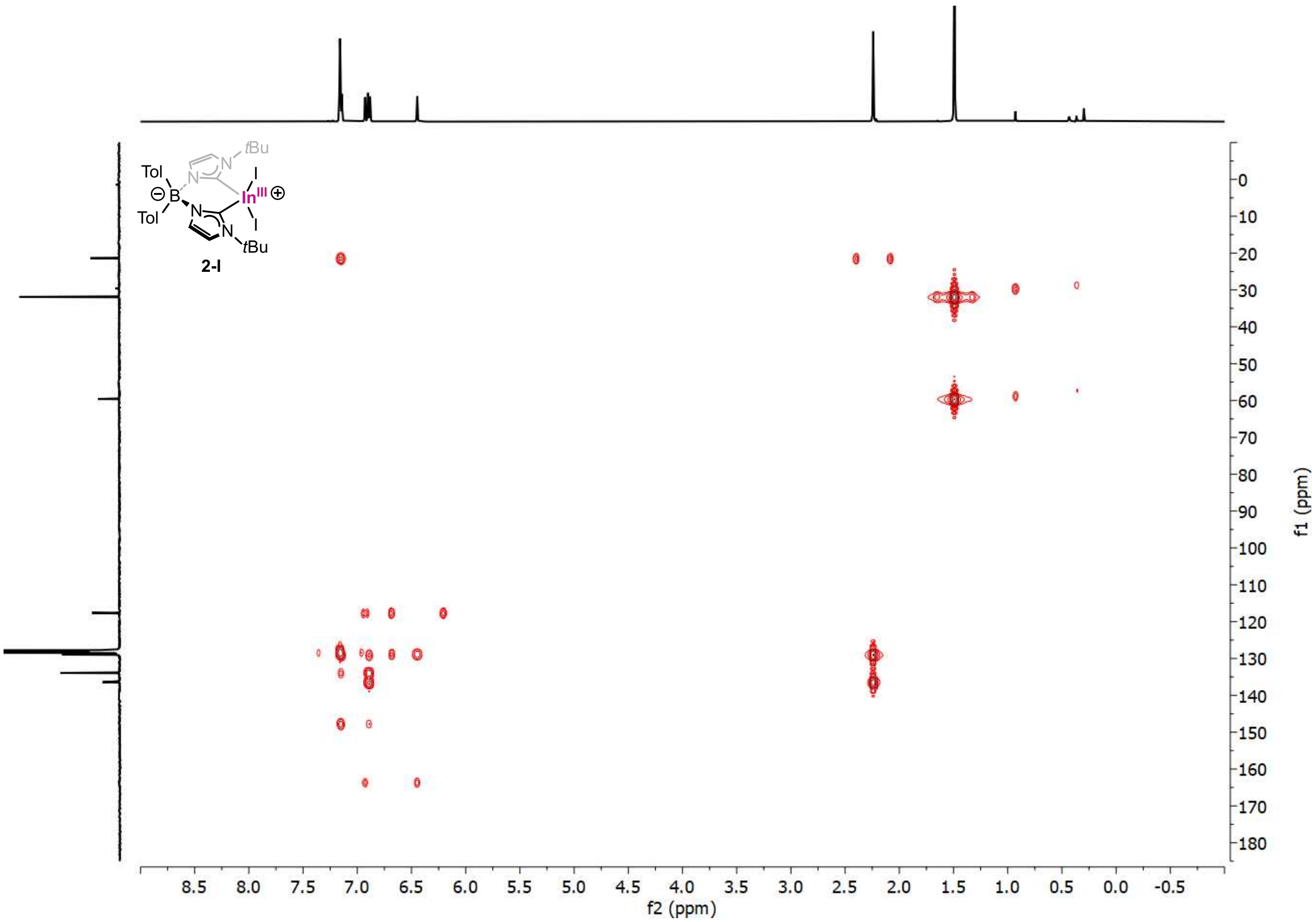

Figure S49. ^{1}H-^{13}C HMBC NMR (C_6D_6, 400 MHz, 298 K) of **2-I**.

8.2 NMR characterisation of diindium complexes 3-X

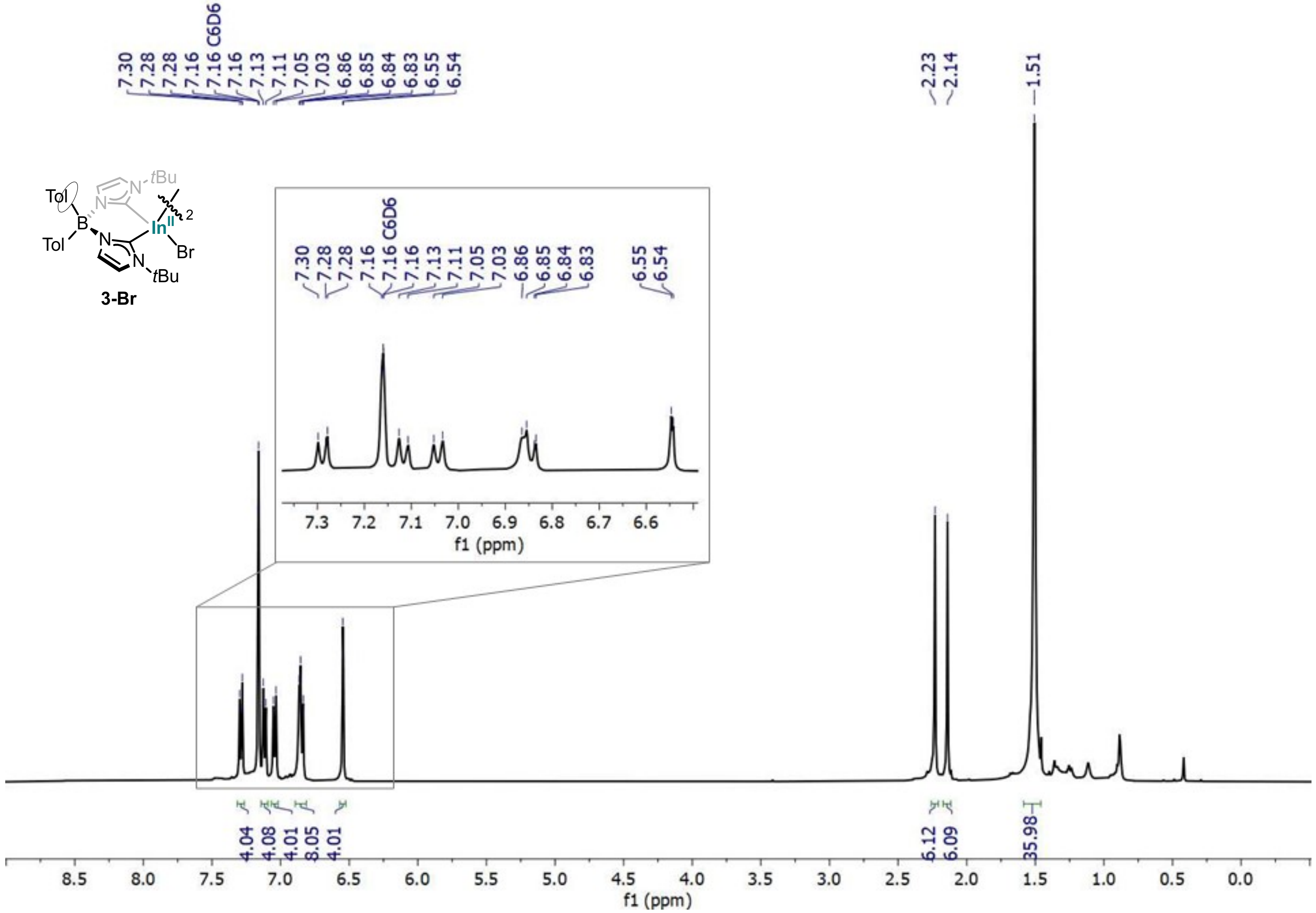

Figure S50. ^{1}H NMR (C_6D_6, 400 MHz) of **3-Br**.

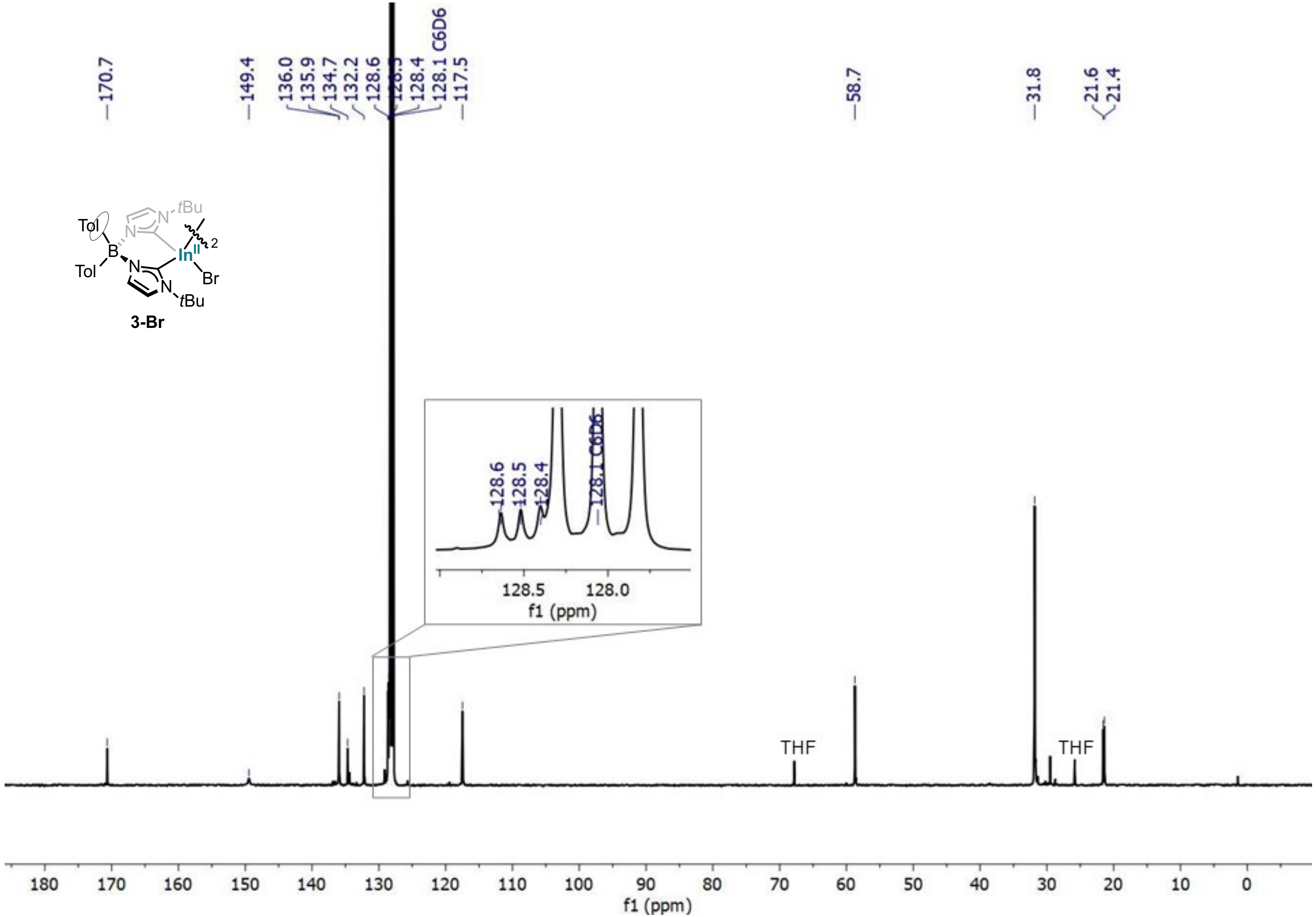

Figure S51. ^{13}C NMR (C_6D_6, 400 MHz) of **3-Br**.

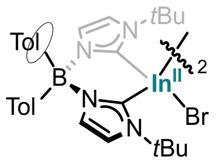

3-Br

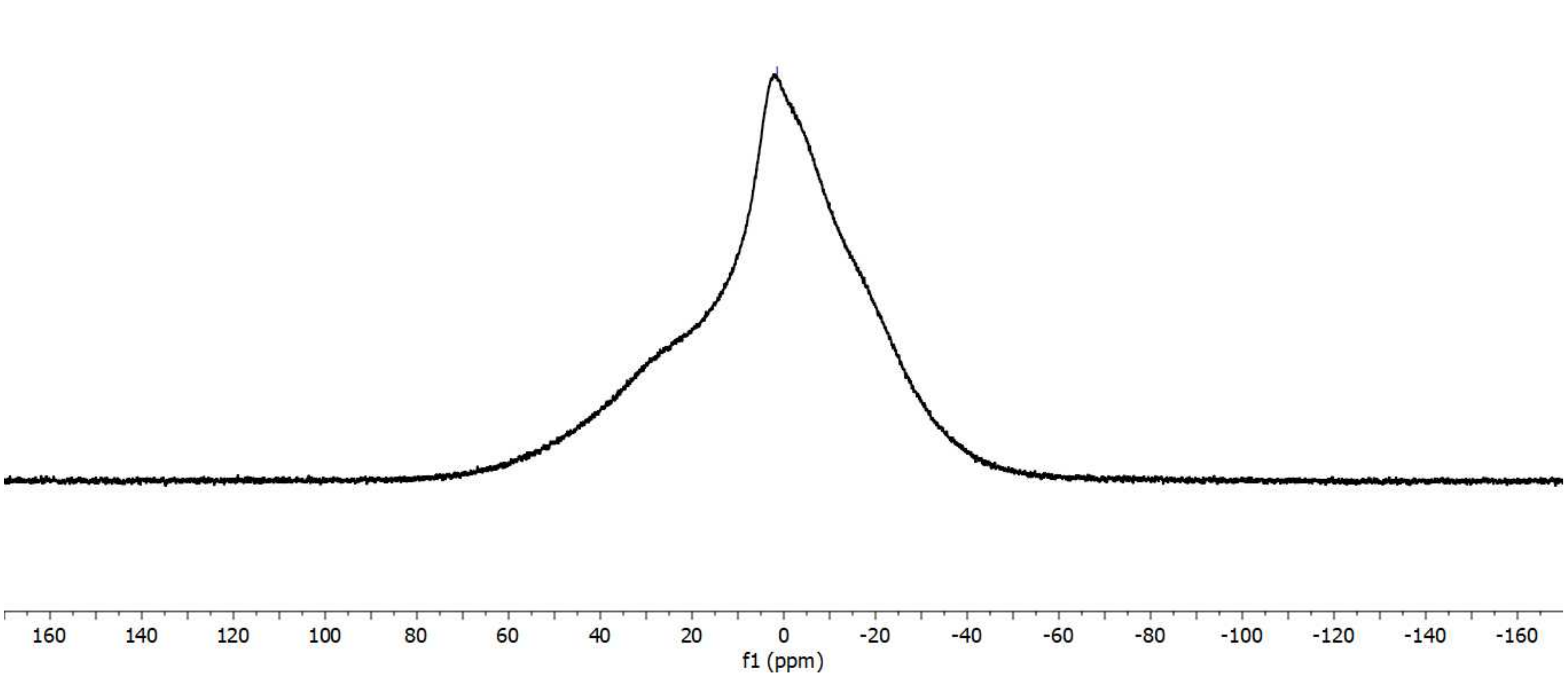

Figure S52. ^{11}B NMR (C_6D_6, 128 MHz) of **3-Br**.

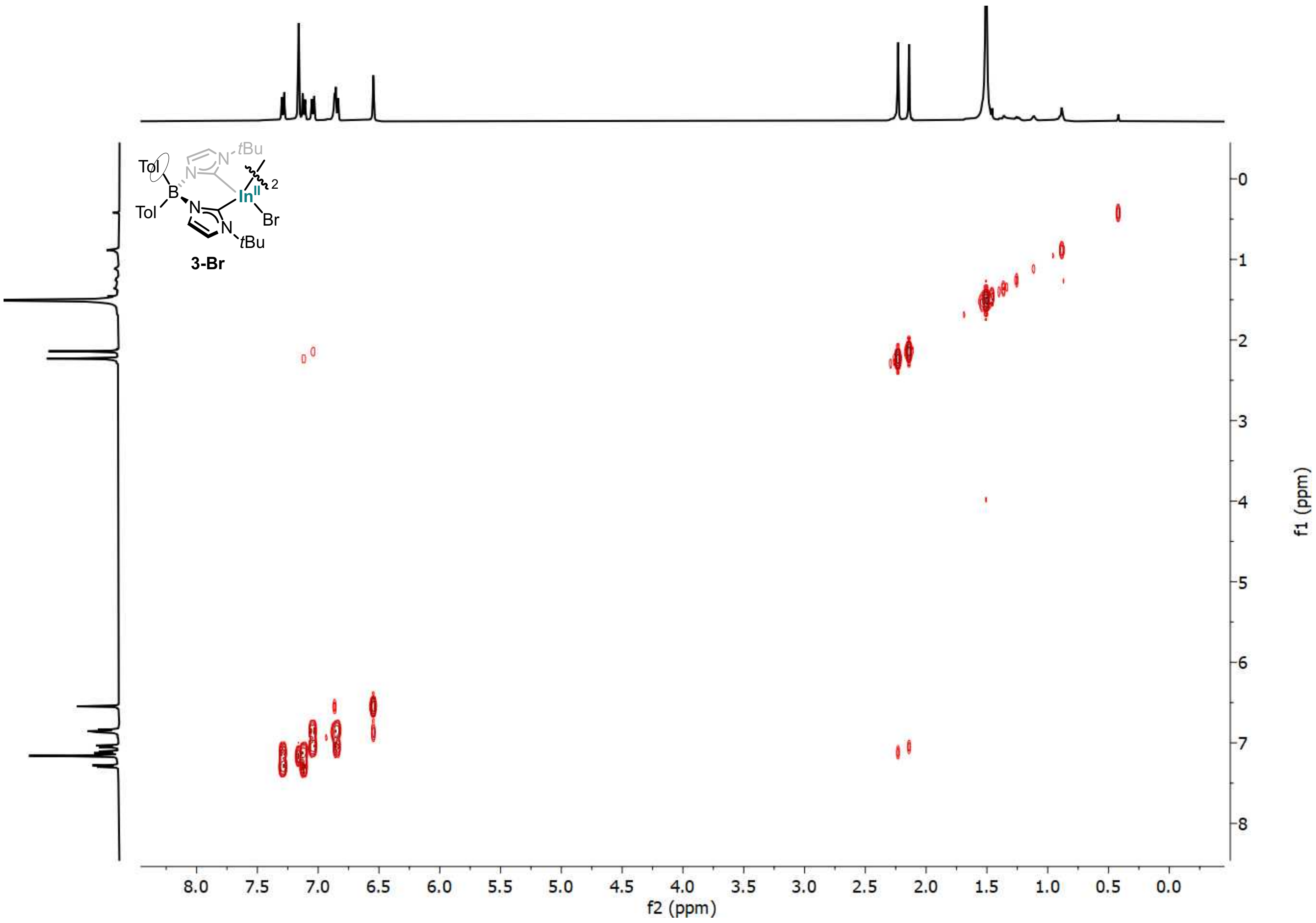

Figure S53. ^1H-^1H COSY NMR (C_6D_6, 400 MHz) of **3-Br**.

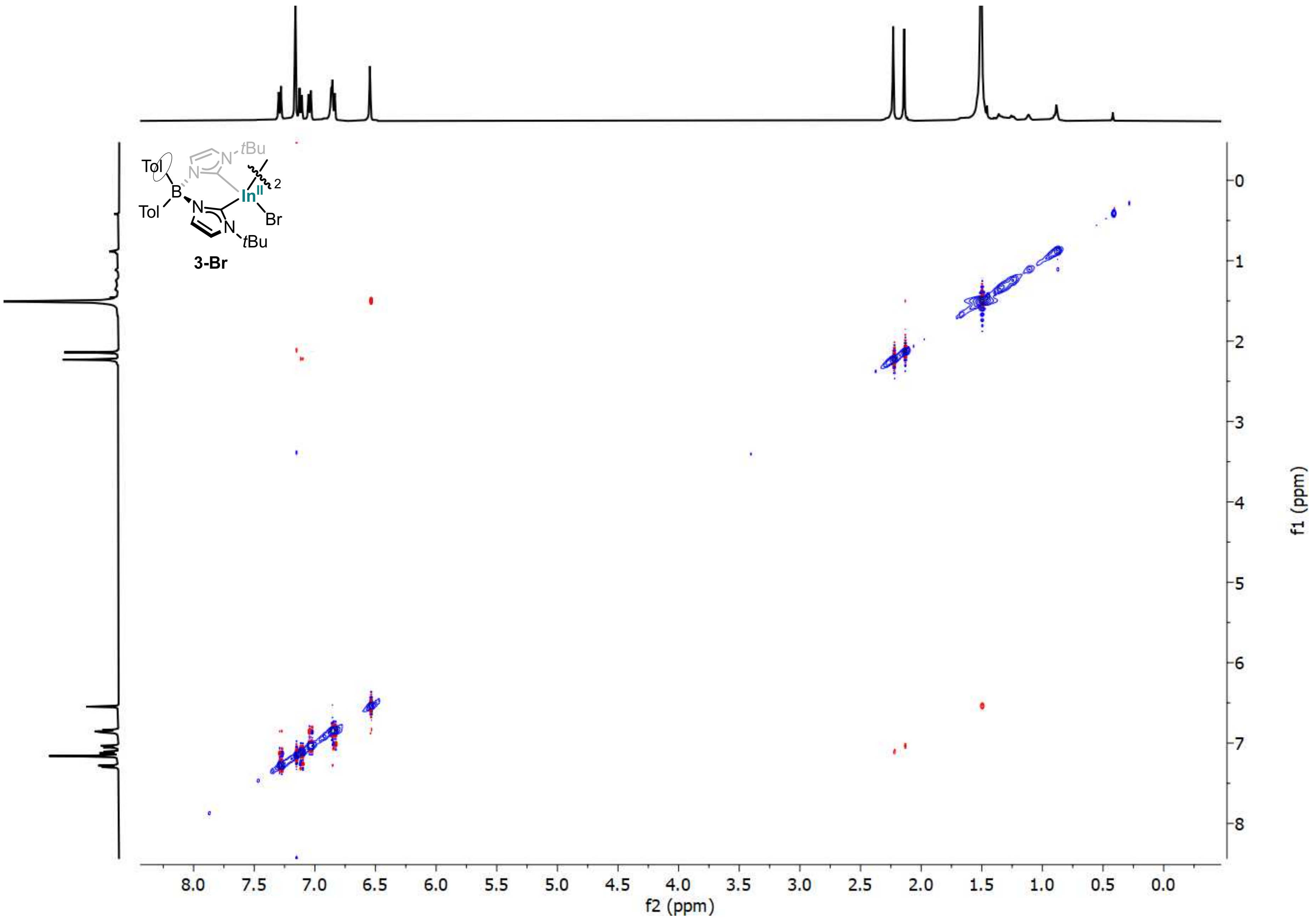

Figure S54. ^{1}H-^{1}H NOESY NMR (C_6D_6, 400 MHz, 298 K) of **3-Br**.

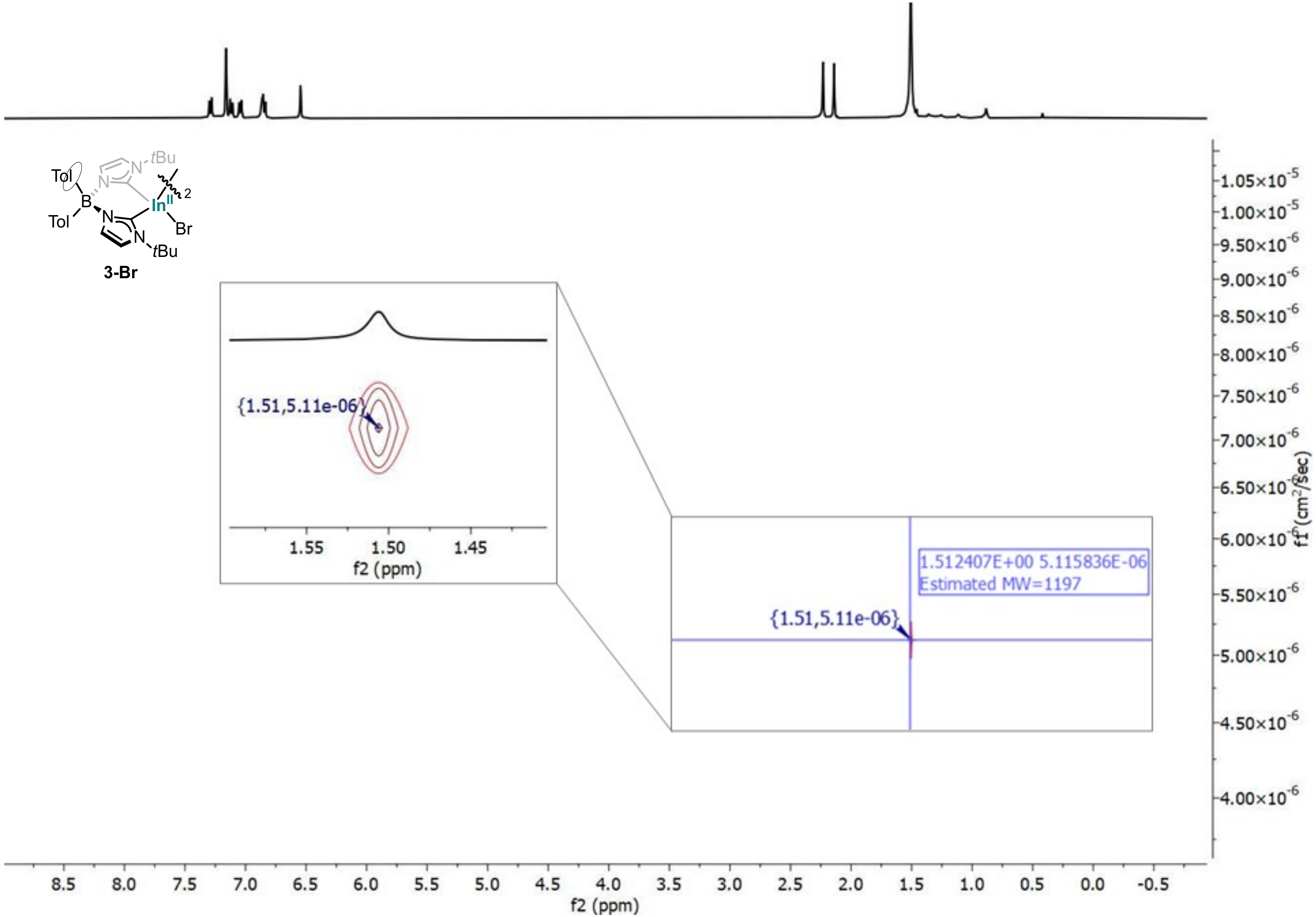

Figure S55. ^{1}H DOSY NMR (C_6D_6, 400 MHz) of **3-Br**.

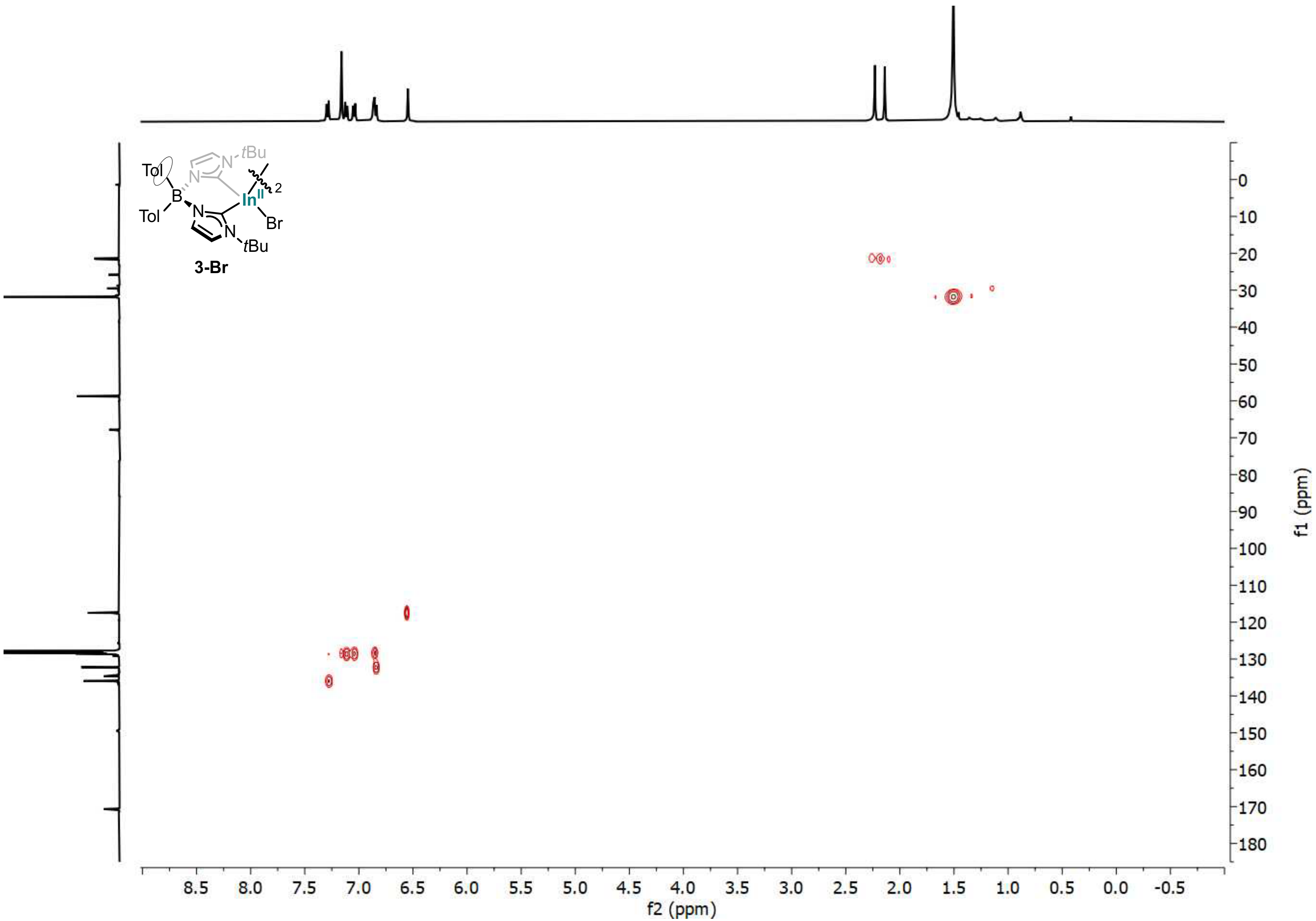

Figure S56. ^{1}H-^{13}C HSQC NMR (C_6D_6, 400 MHz) of **3-Br**.

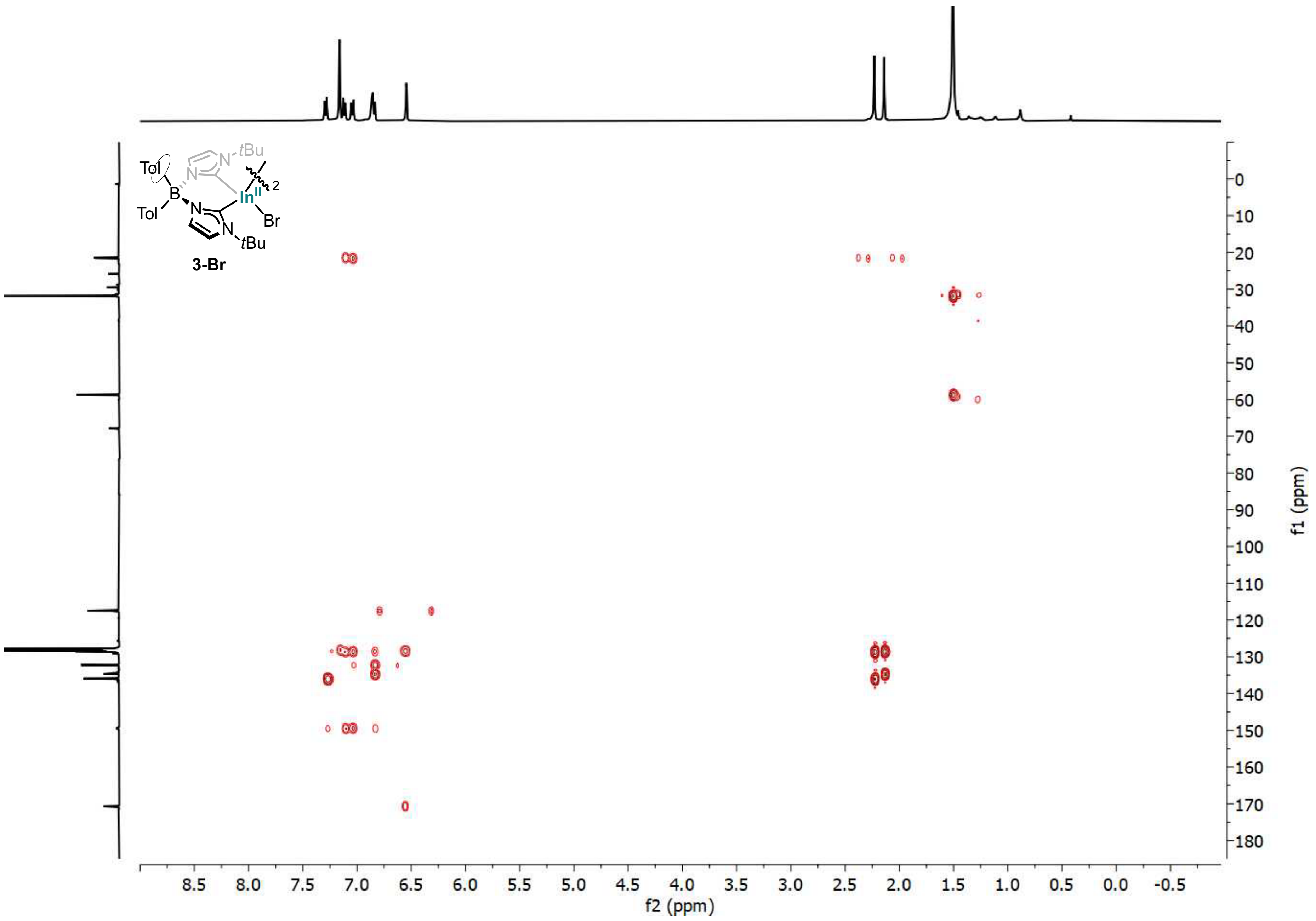

Figure S57. ^{1}H-^{13}C HMBC NMR (C_6D_6, 400 MHz) of **3-Br**.

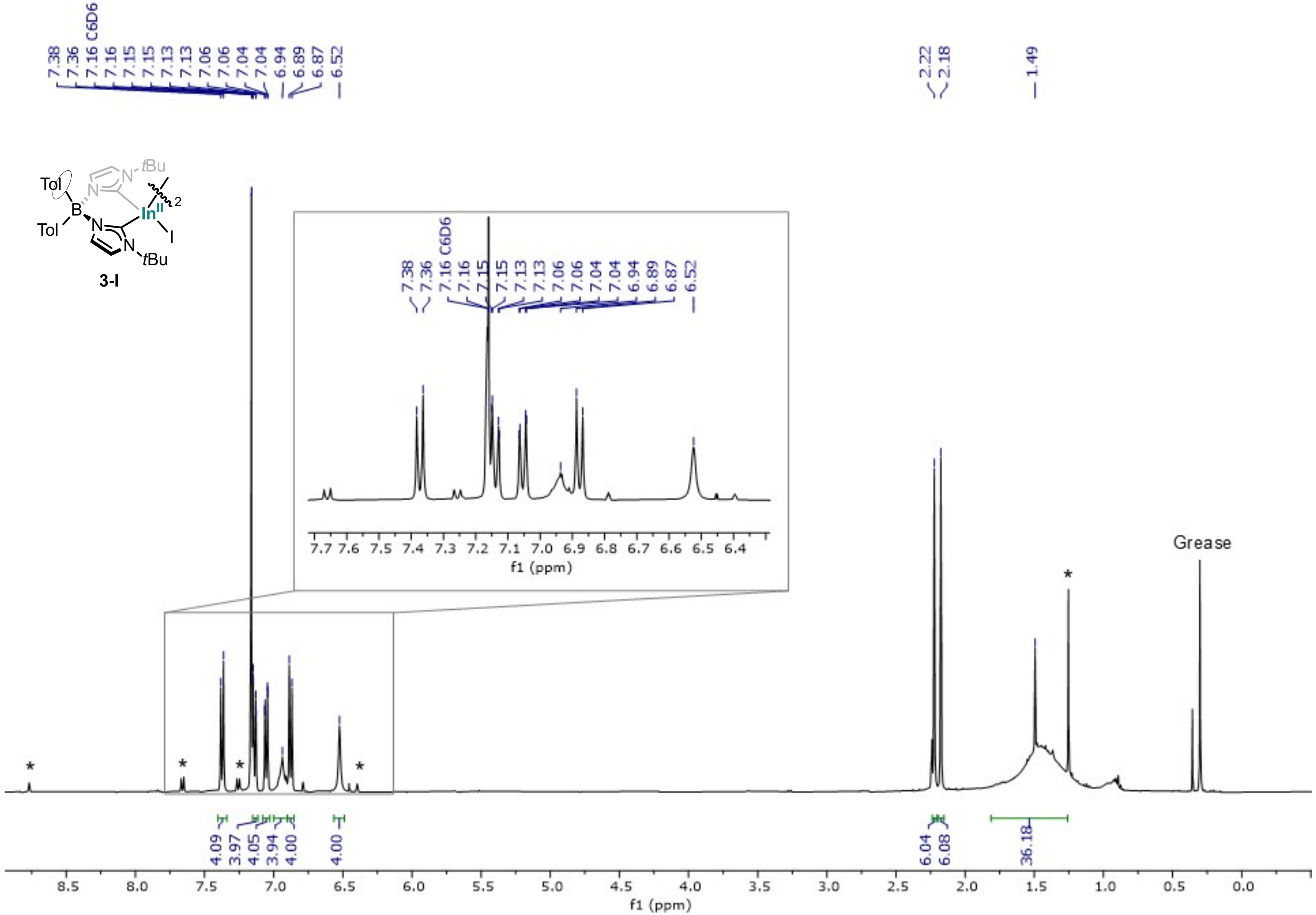

Figure S58. ^1H NMR (C_6D_6, 400 MHz) of **3-I**. Ligand impurity indicated by (*).

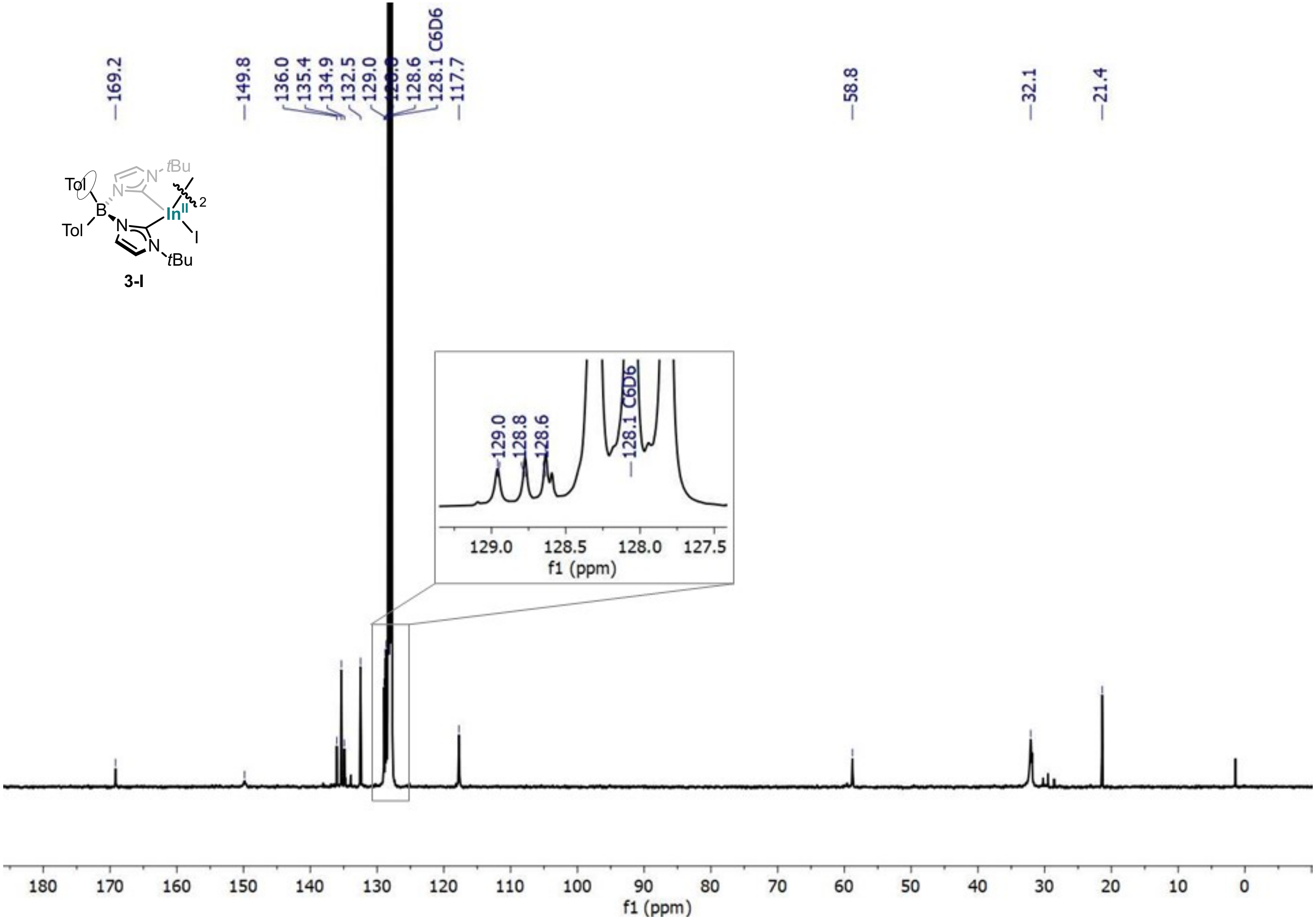

Figure S59. ^{13}C NMR (C_6D_6, 101 MHz) of **3-I**.

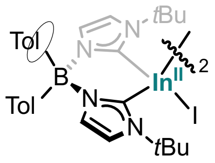

3-I

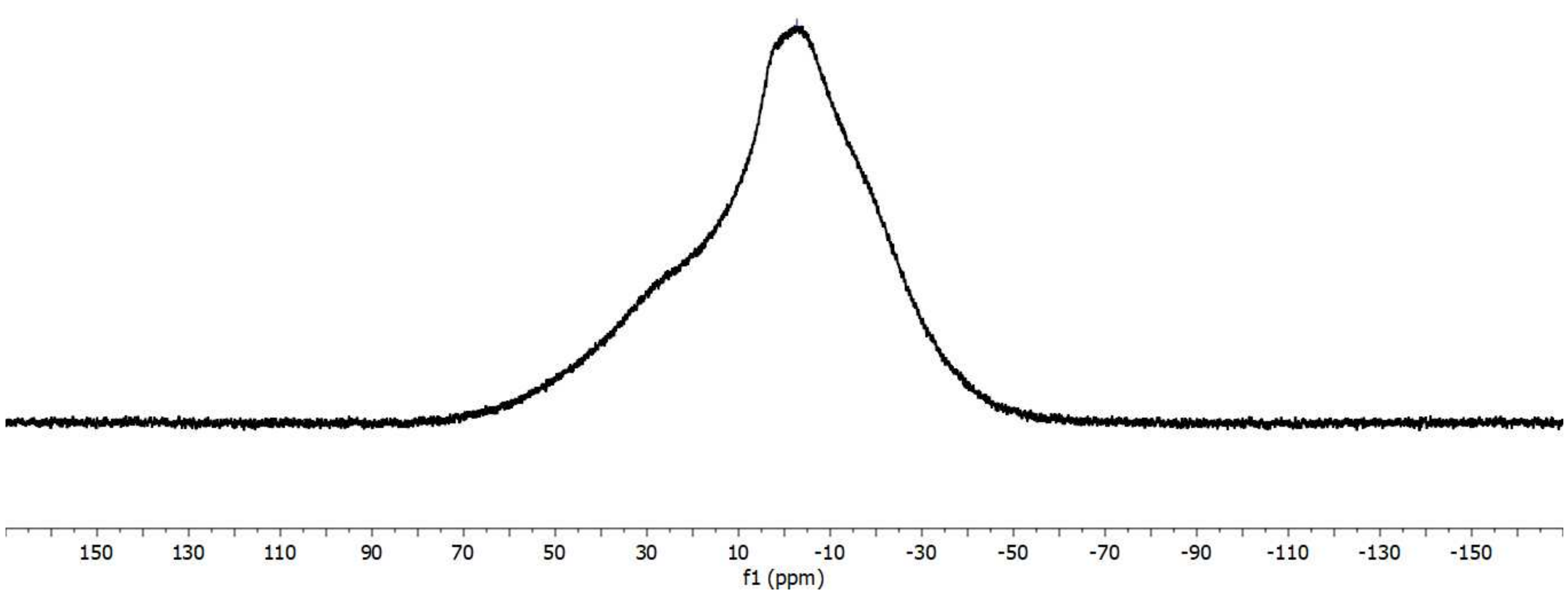

Figure S60. ^{11}B NMR (C_6D_6, 128 MHz) of **3-I**.

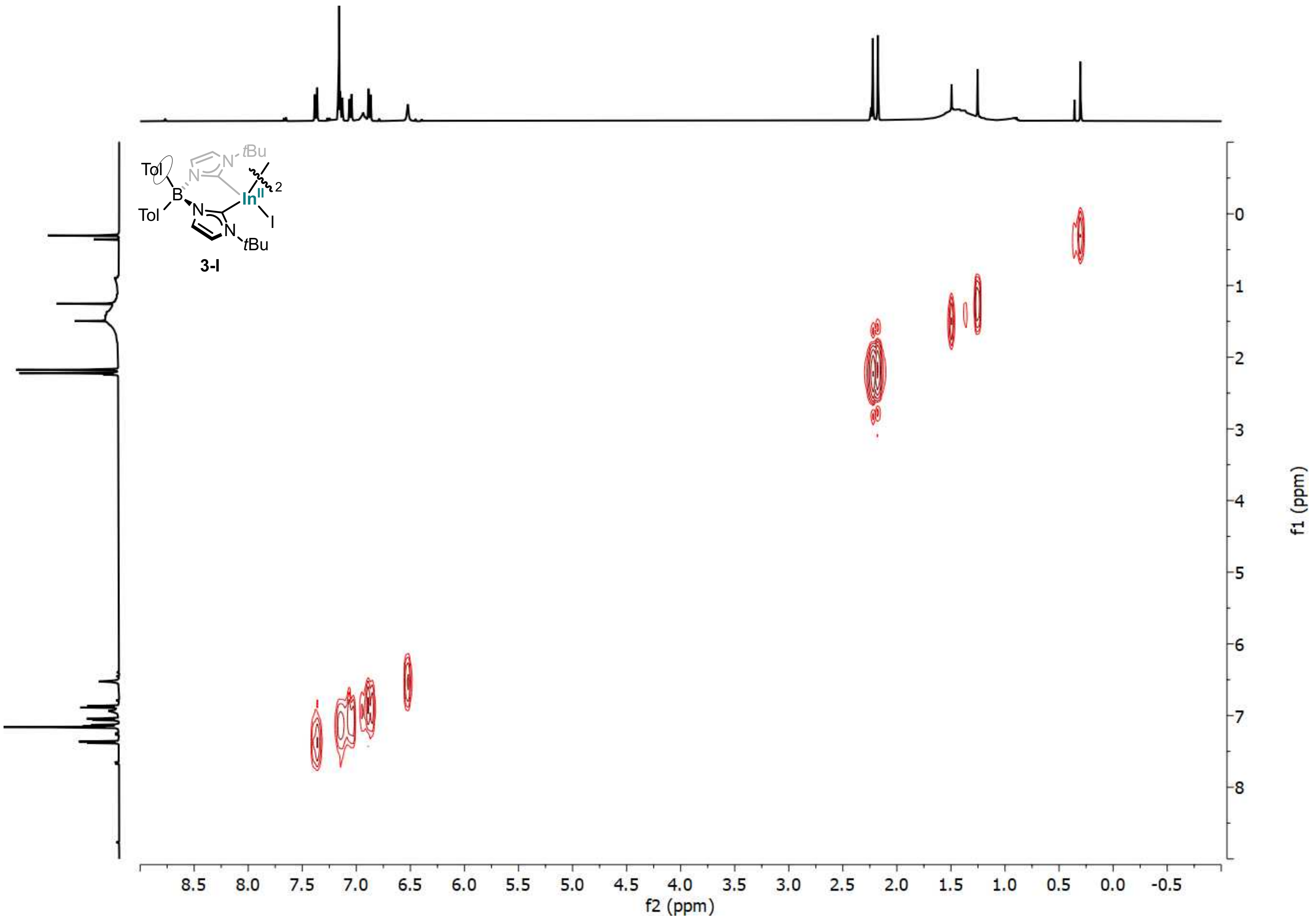

Figure S61. ^{1}H-^{1}H COSY NMR (C_6D_6, 400 MHz) of **3-I**.

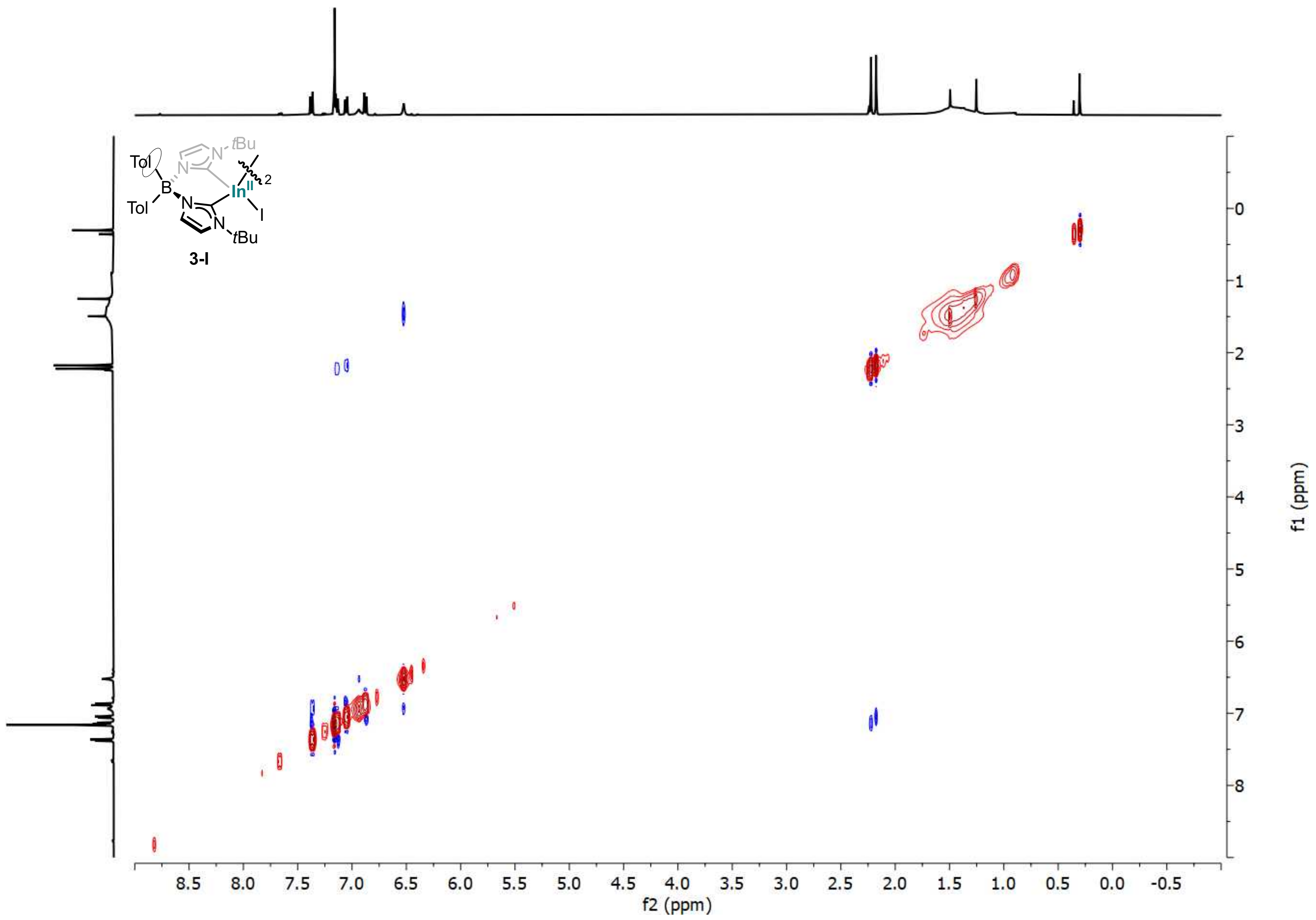

Figure S62. ^{1}H-^{1}H NOESY NMR (C_6D_6, 400 MHz, 298 K) of **3-I**.

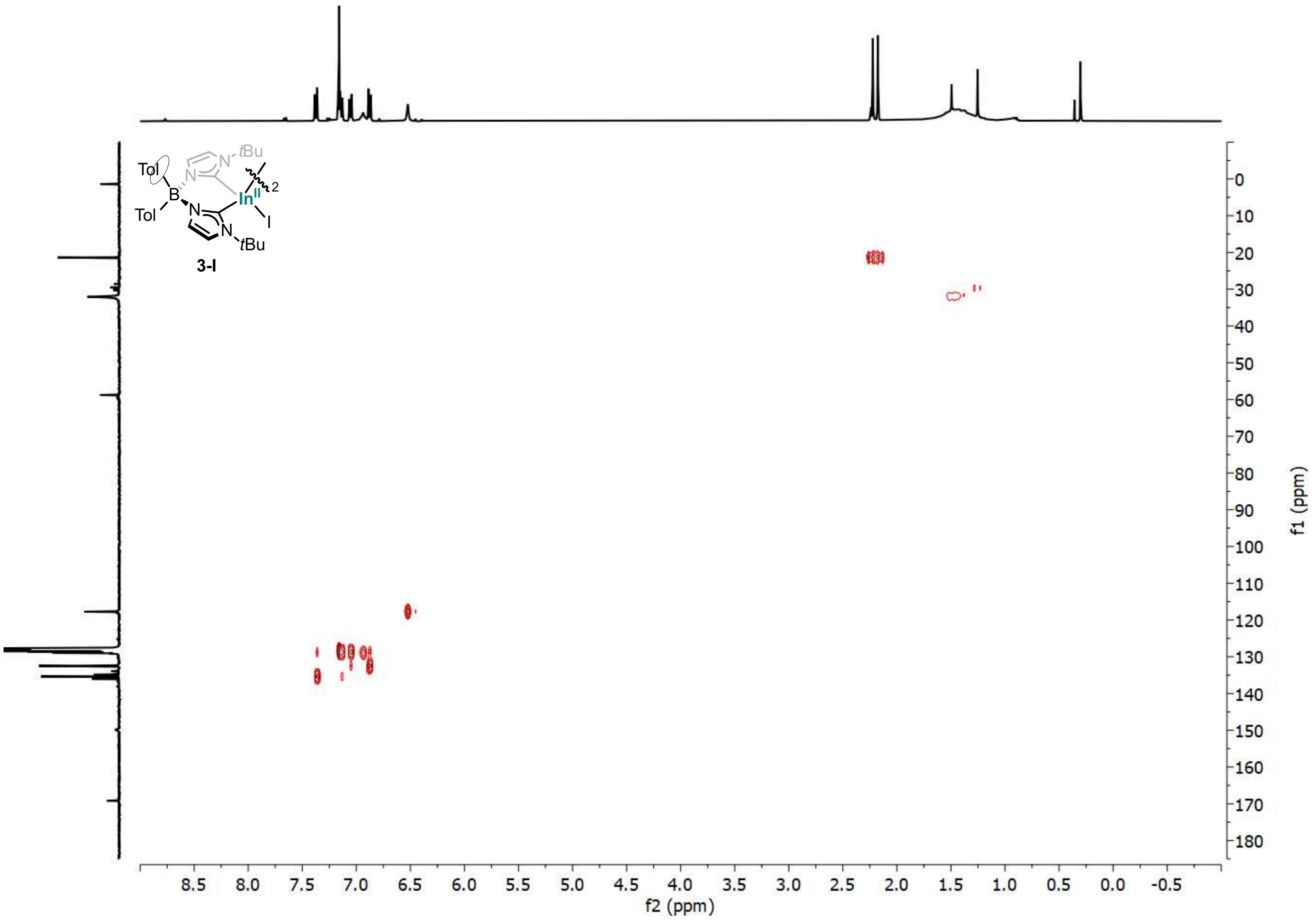

Figure S63. ^{1}H-^{13}C HSQC NMR (C_6D_6, 400 MHz) of **3-I**.

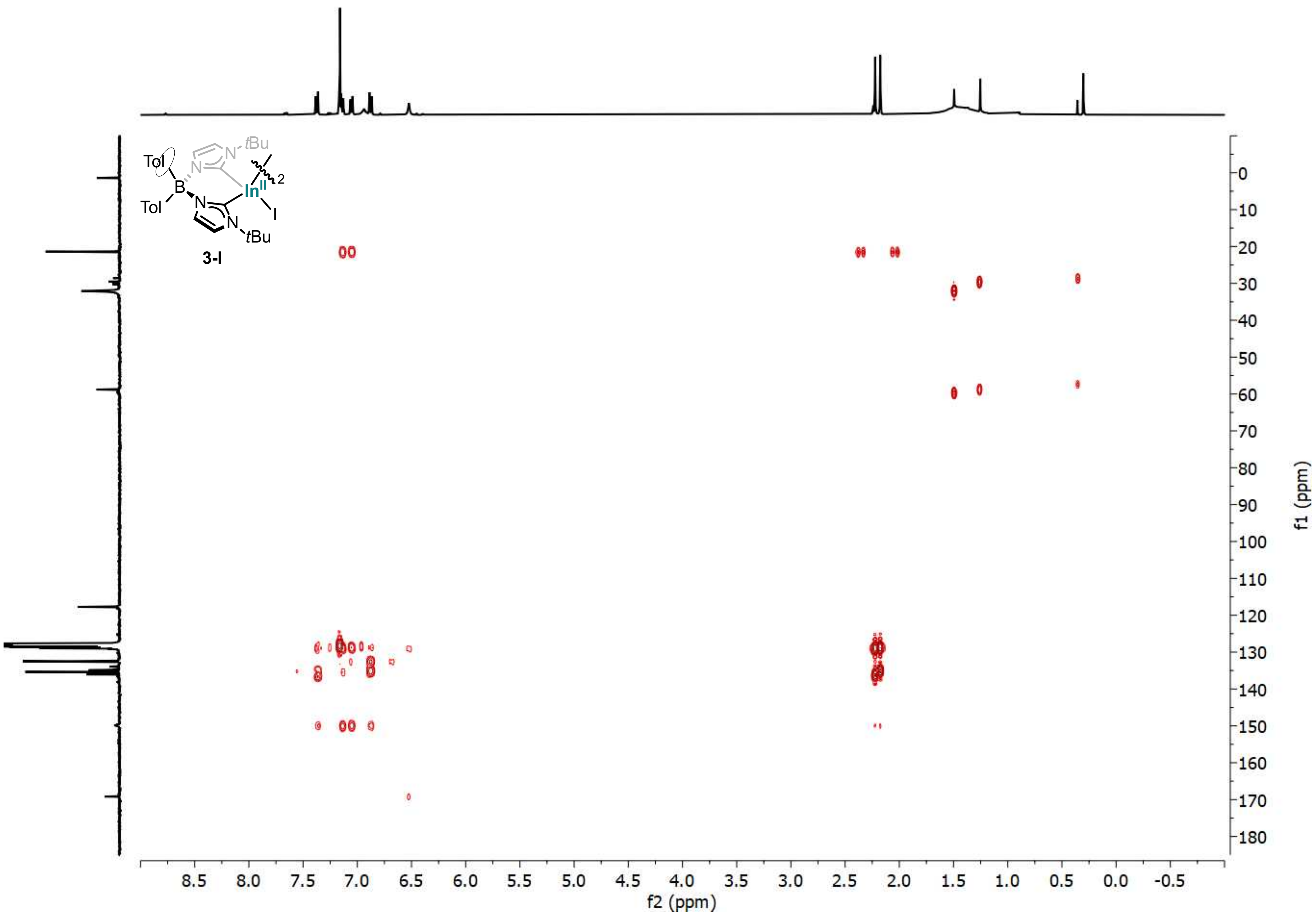

Figure S64. ^{1}H-^{13}C HMBC NMR (C_6D_6, 400 MHz) of **3-I**.

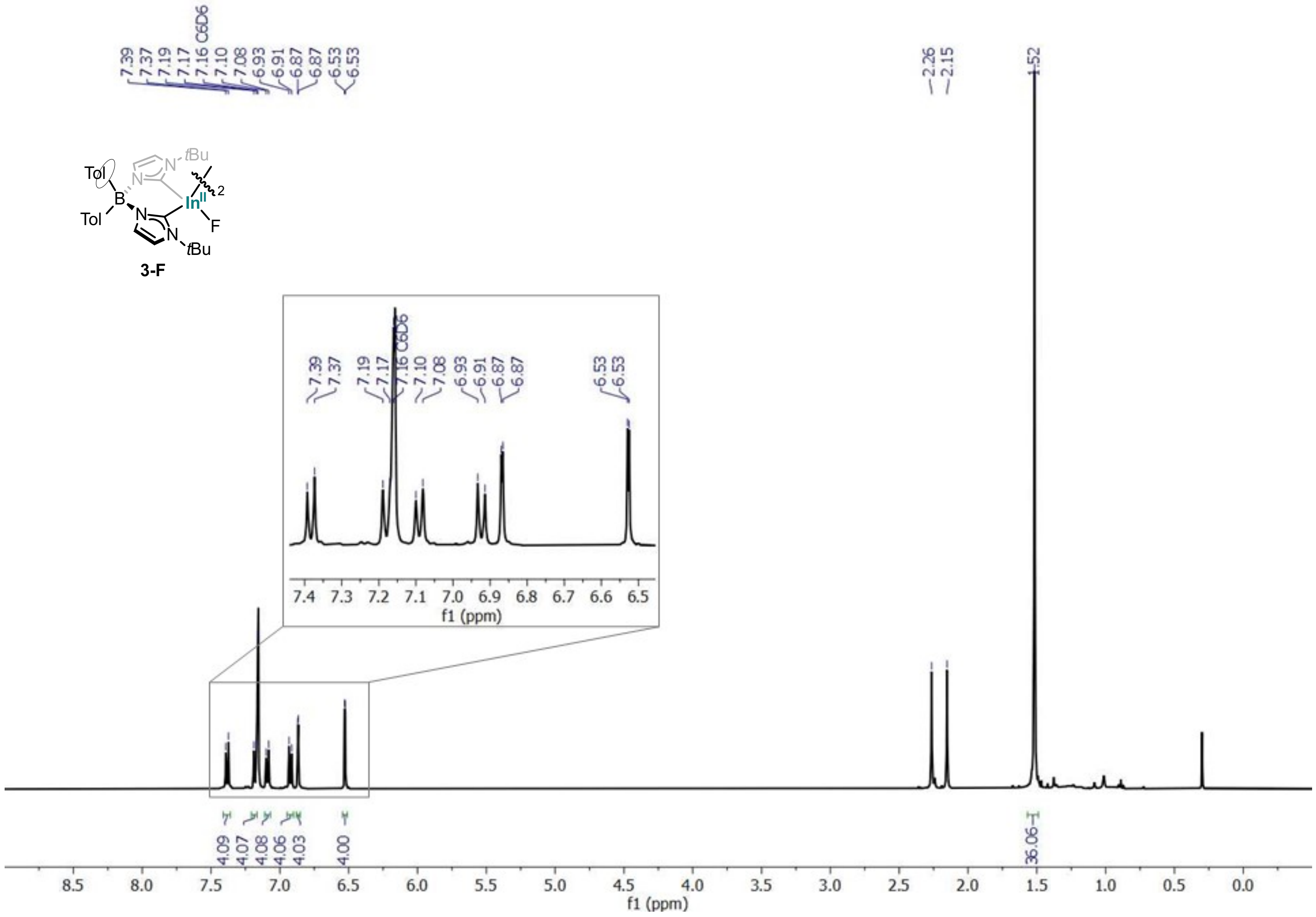

Figure S65. ^{1}H NMR (C_6D_6, 400 MHz) of **3-F**.

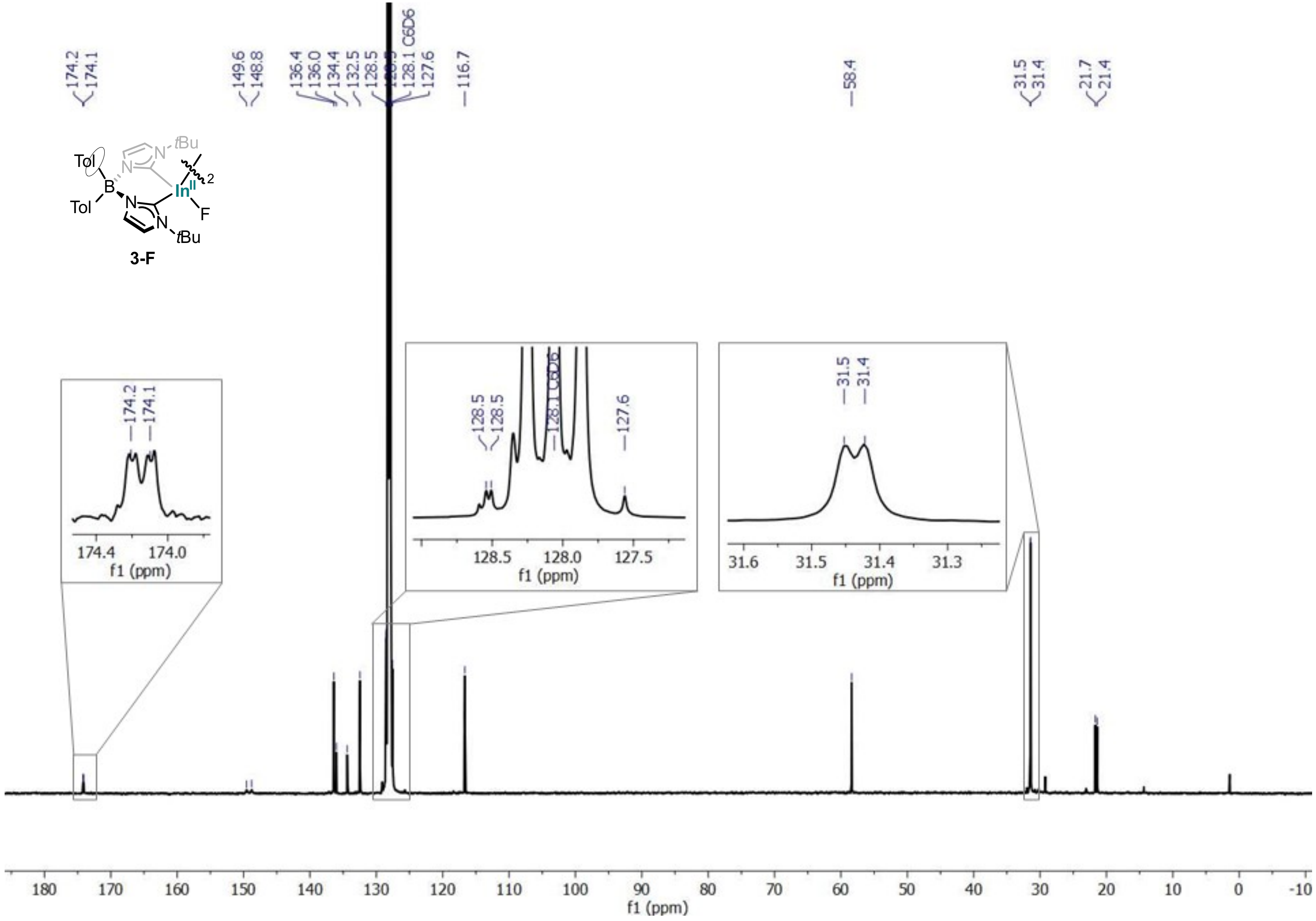

Figure S66. ^{13}C NMR (C_6D_6, 101 MHz) of **3-F**.

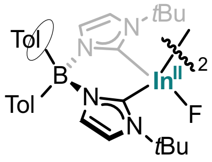

3-F

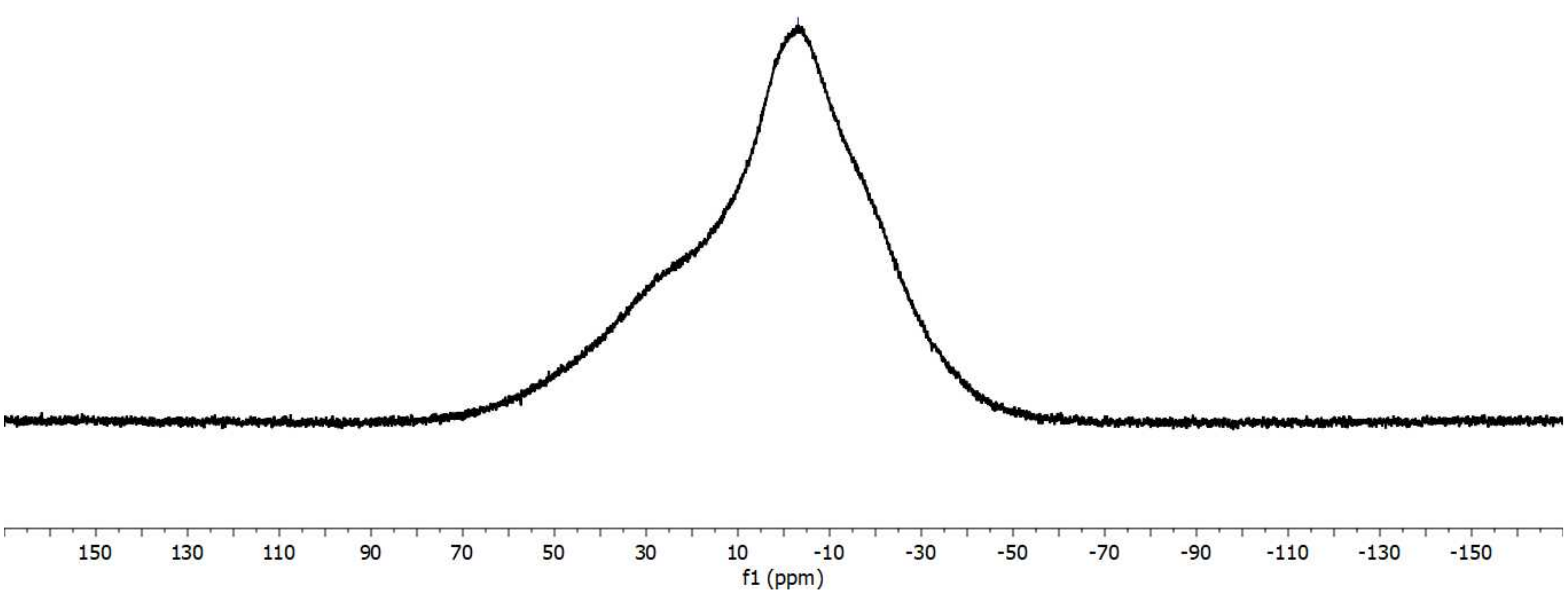

Figure S67. ^{11}B NMR (C_6D_6, 128 MHz) of **3-F**.

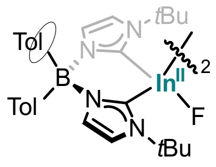

3-F

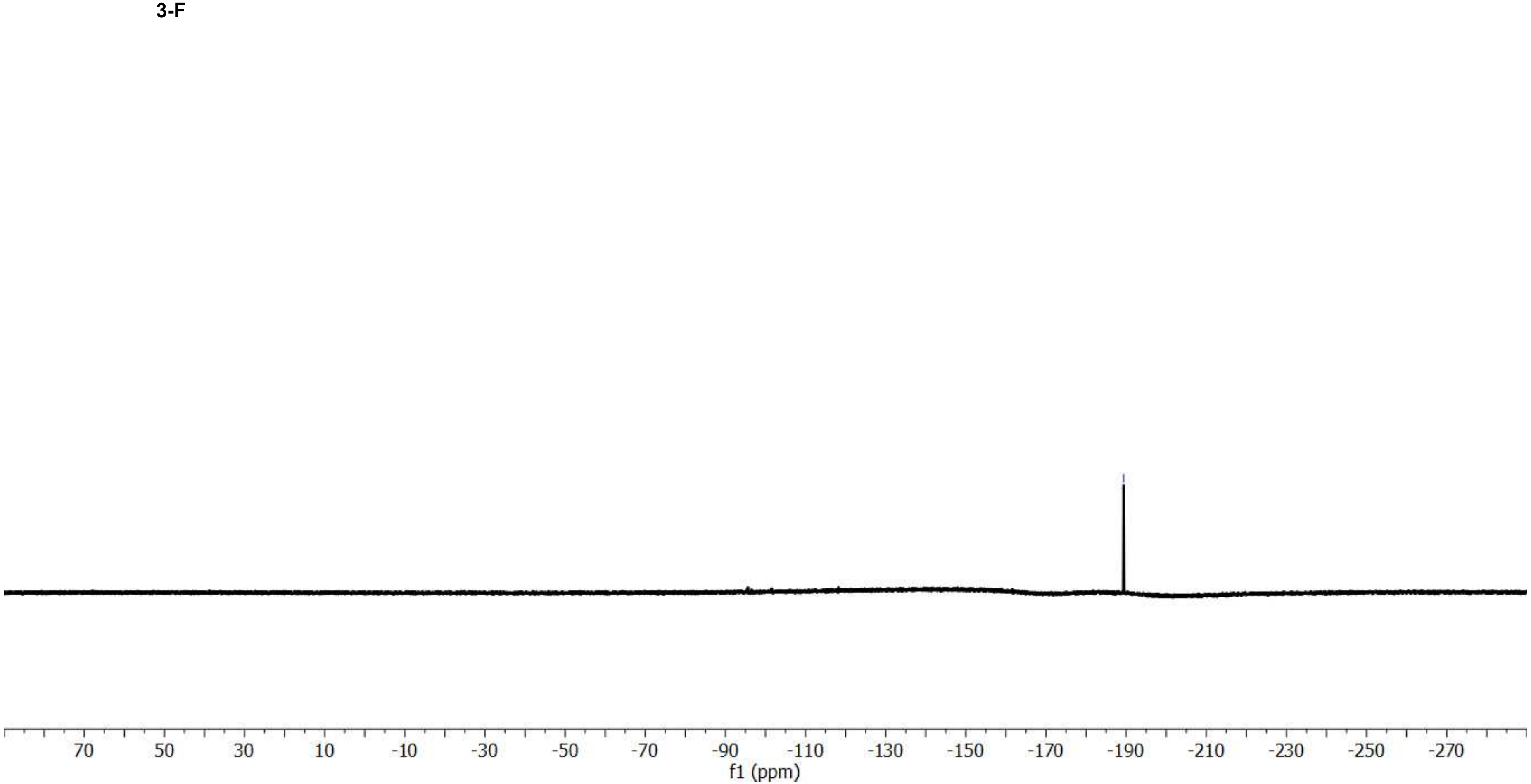

Figure S68. ^{19}F NMR (C_6D_6, 471 MHz) of **3-F**.

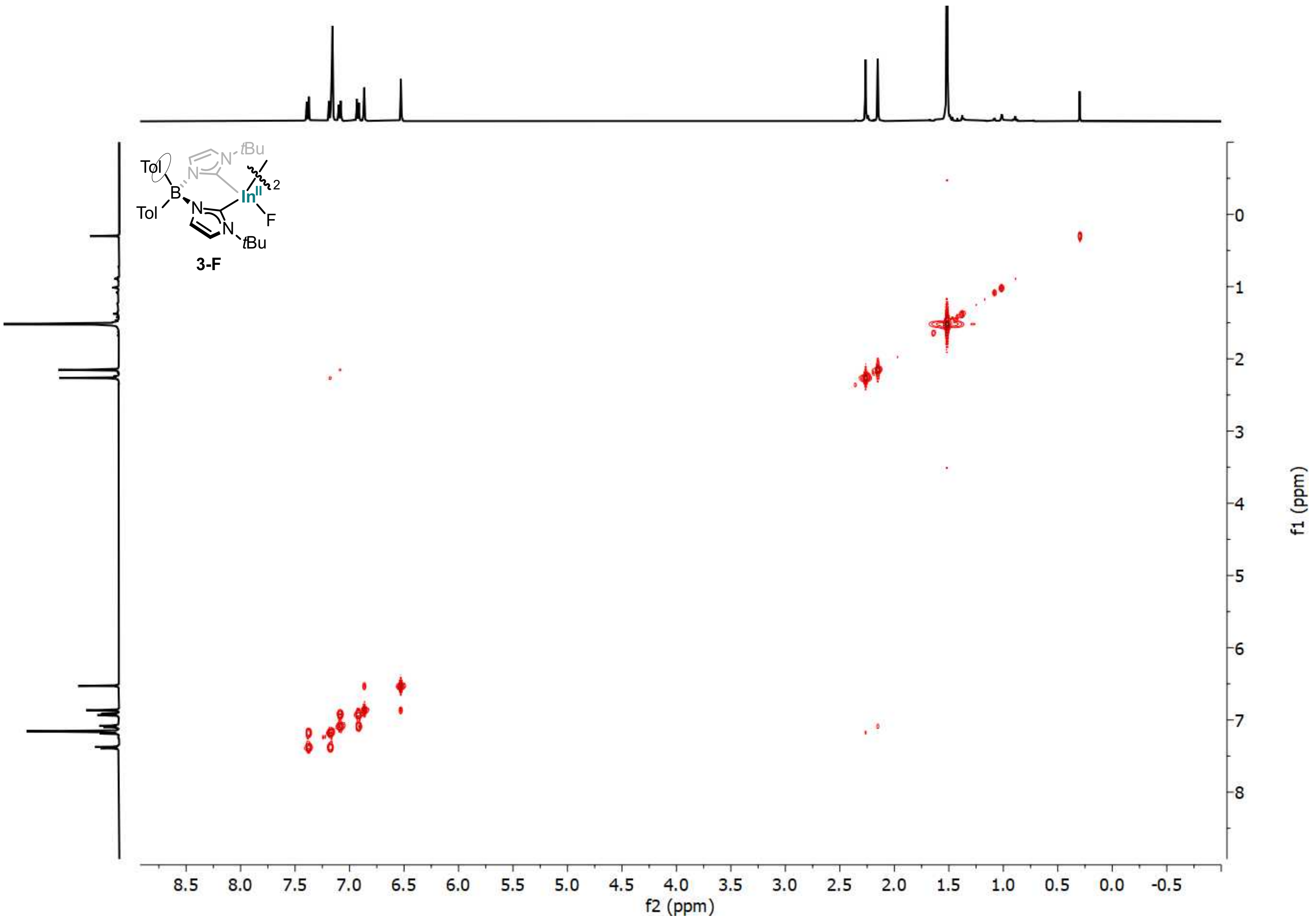

Figure S69. ^{1}H-^{1}H COSY NMR (C_6D_6, 400 MHz) of **3-F**.

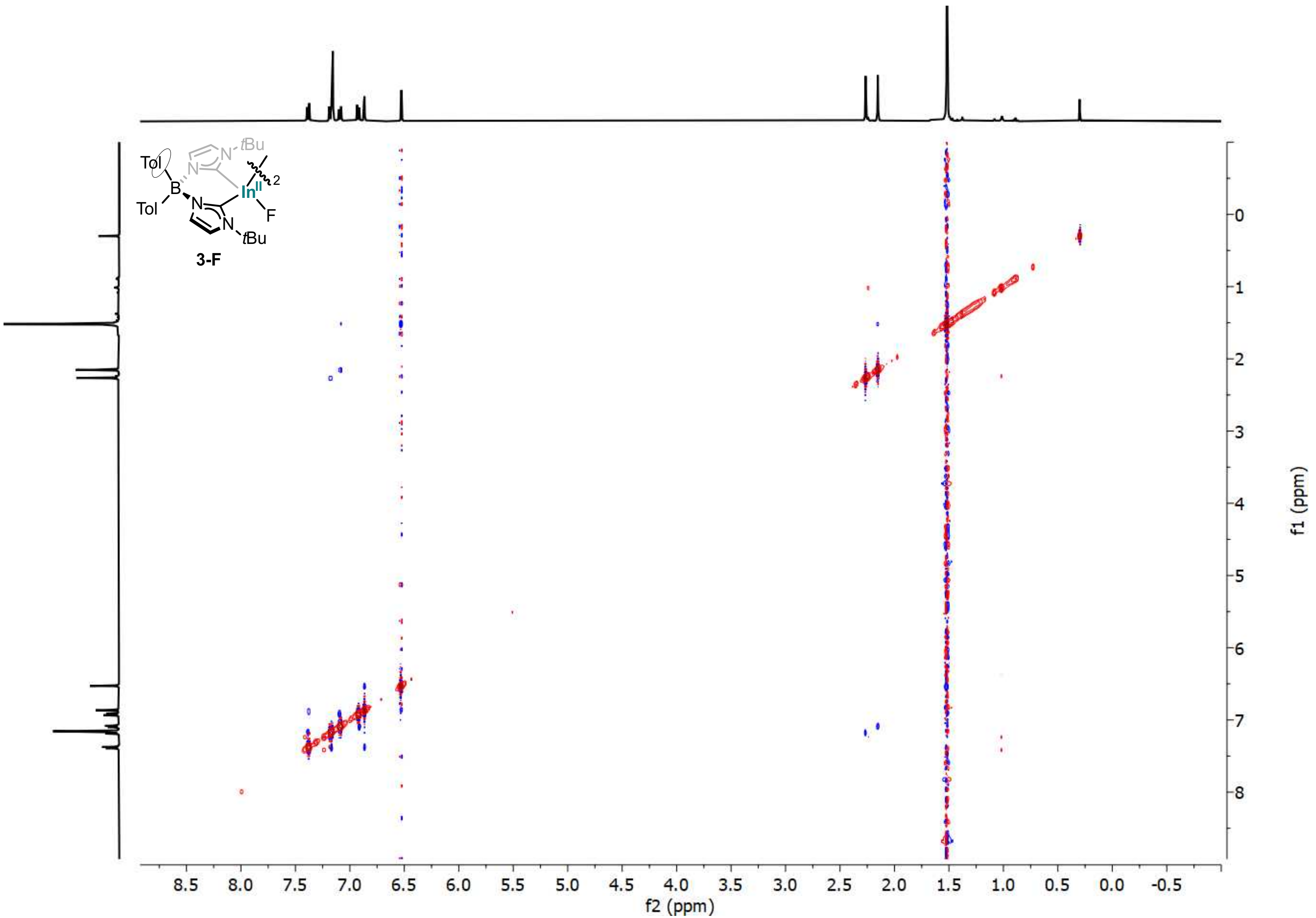

Figure S70. ^{1}H-^{1}H NOESY NMR (C_6D_6, 400 MHz, 298 K) of **3-F**.

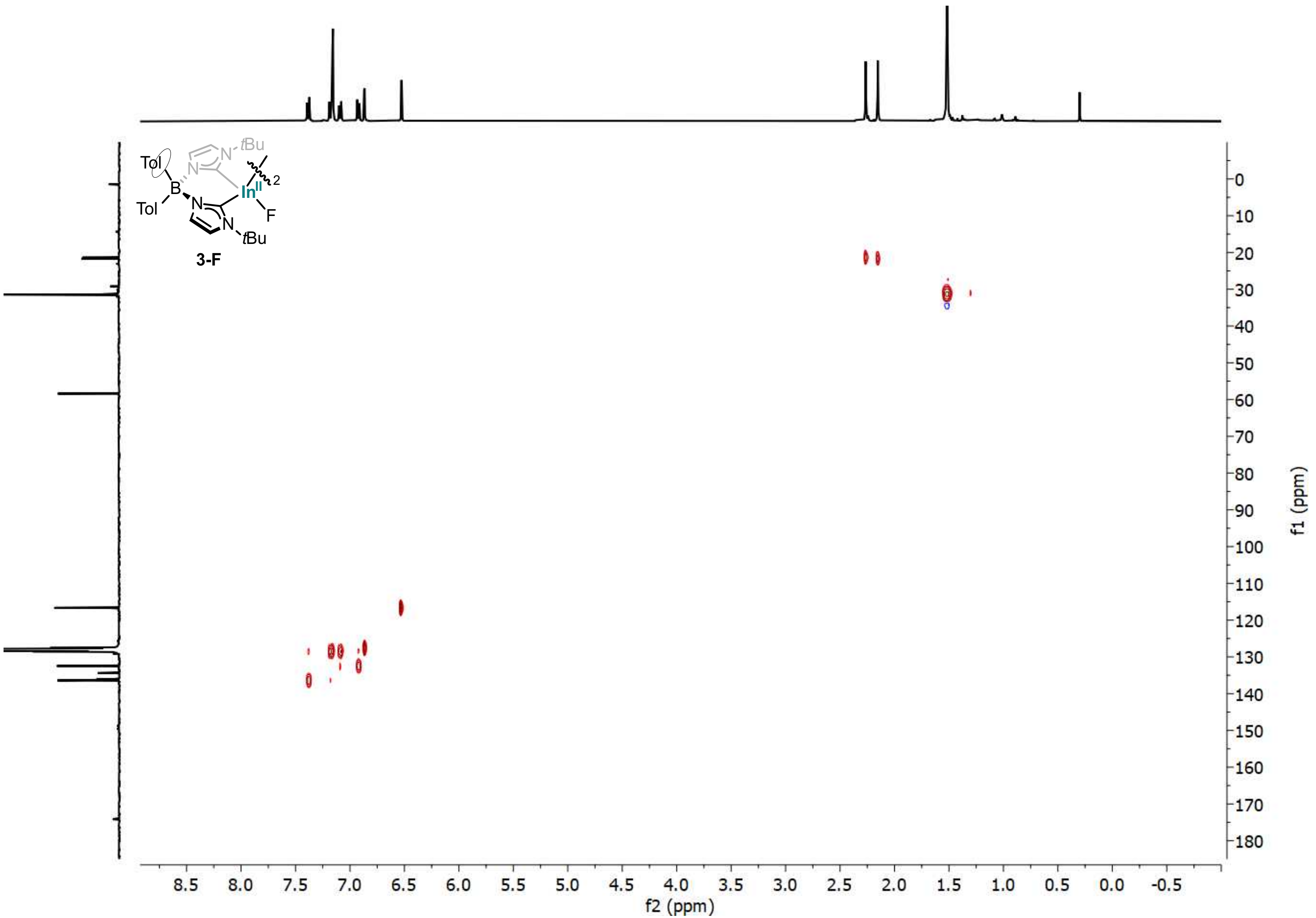

Figure S71. ^{1}H-^{13}C HSQC NMR (C_6D_6, 400 MHz) of **3-F**.

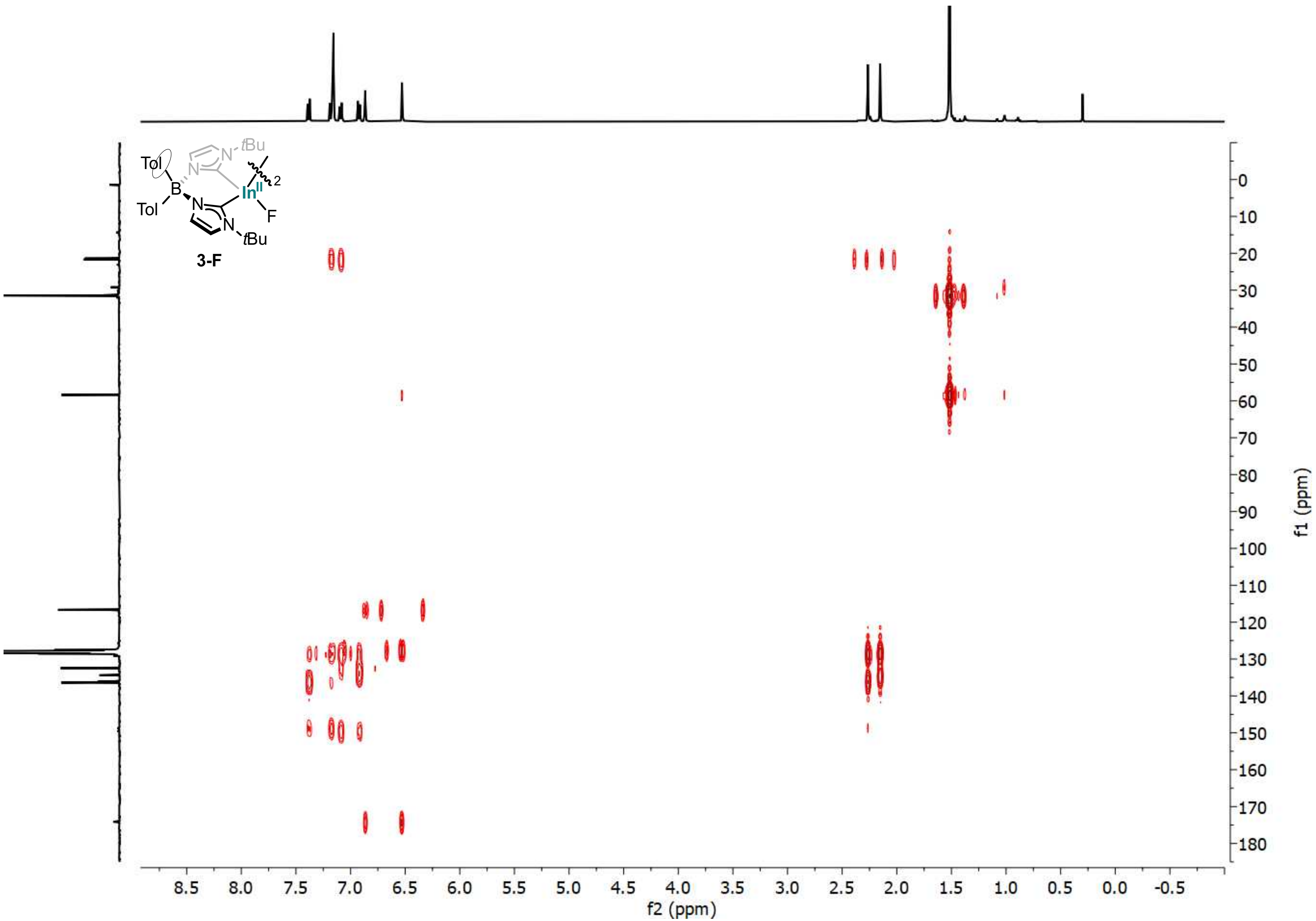

Figure S72. ^{1}H-^{13}C HMBC NMR (C_6D_6, 400 MHz) of **3-F**.

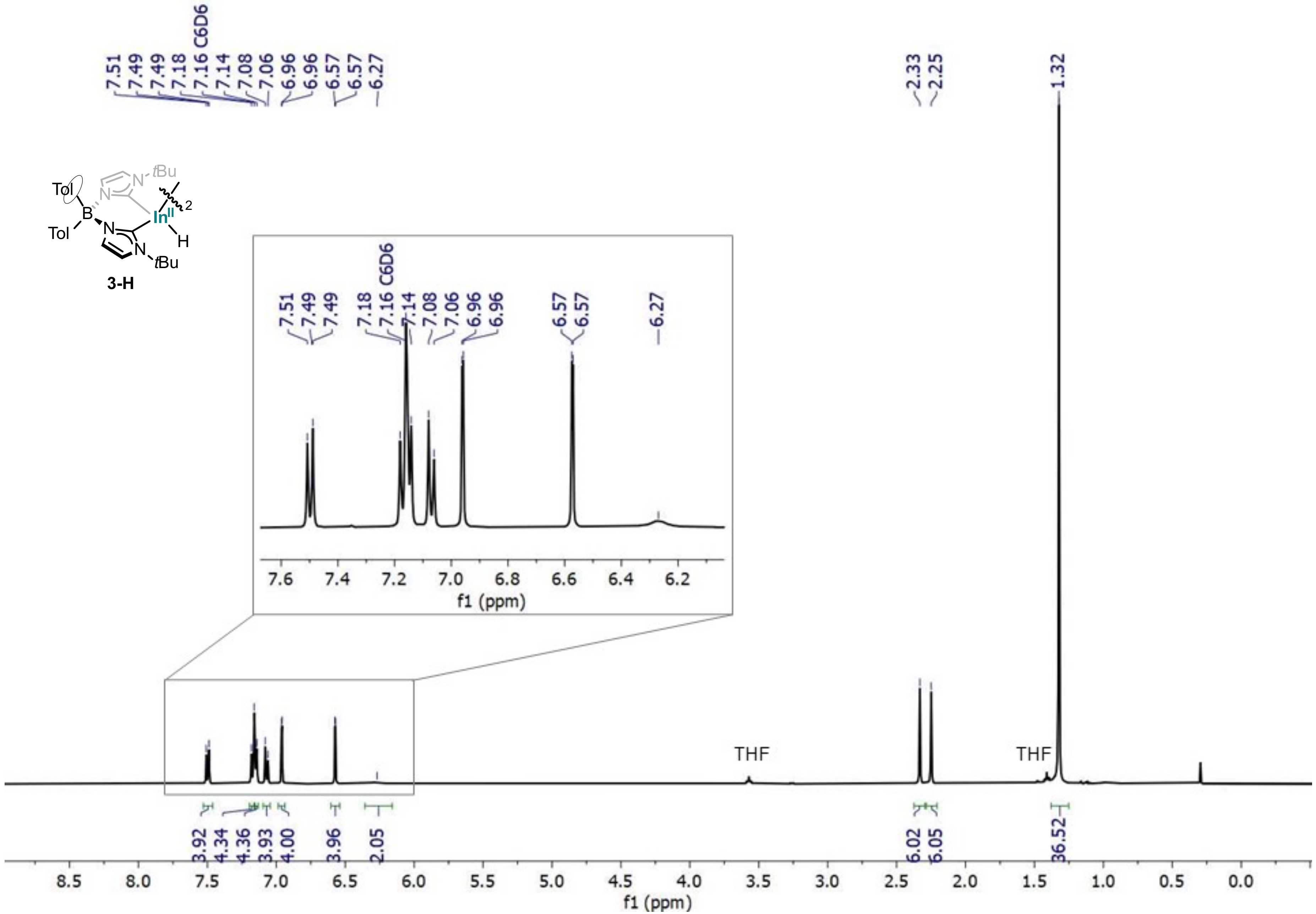

Figure S73. ^{1}H NMR (C_6D_6, 400 MHz) of **3-H**.

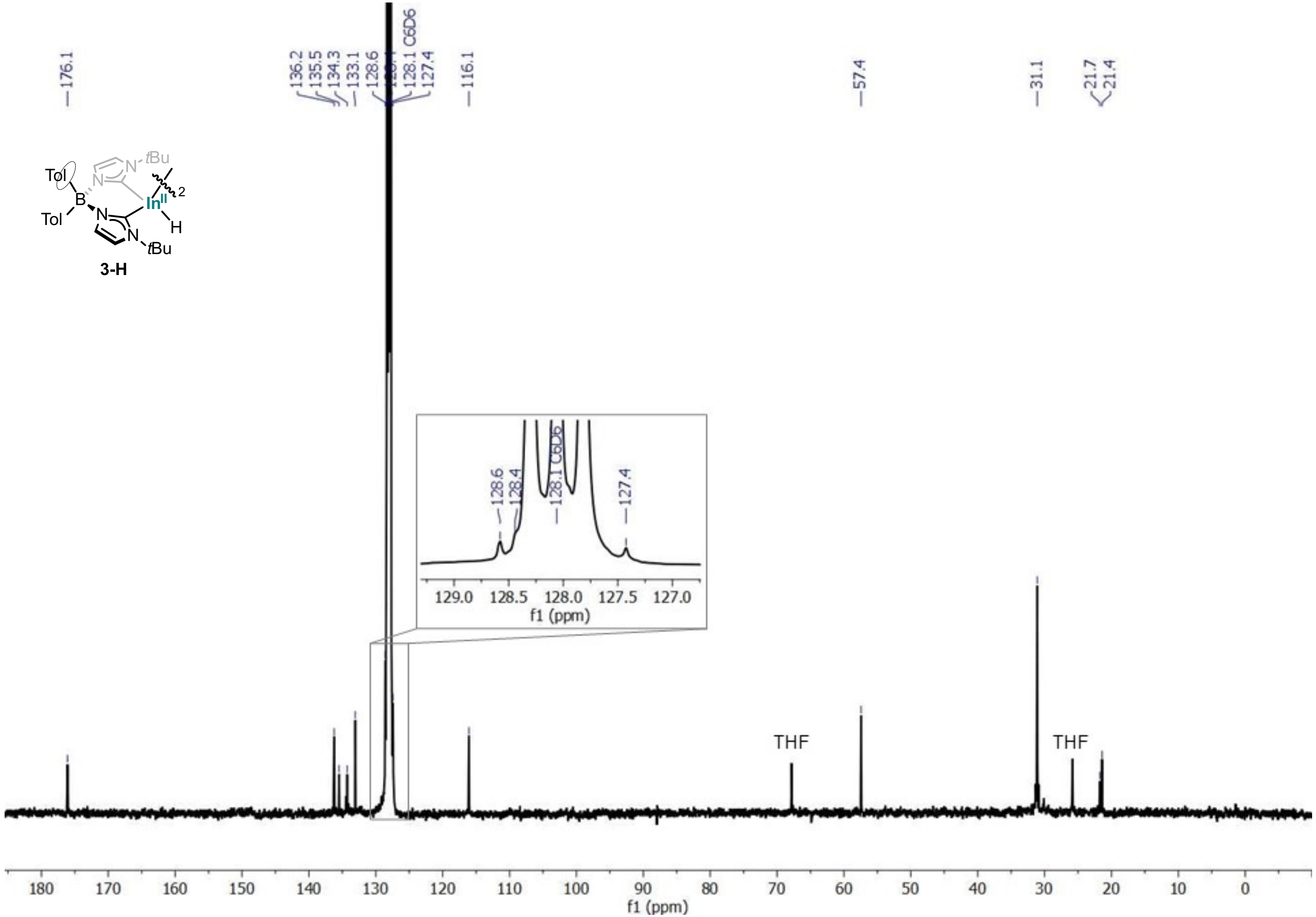

Figure S74. ^{13}C NMR (C_6D_6, 400 MHz) of **3-H**.

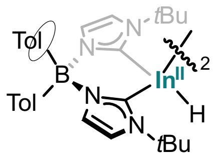

3-H

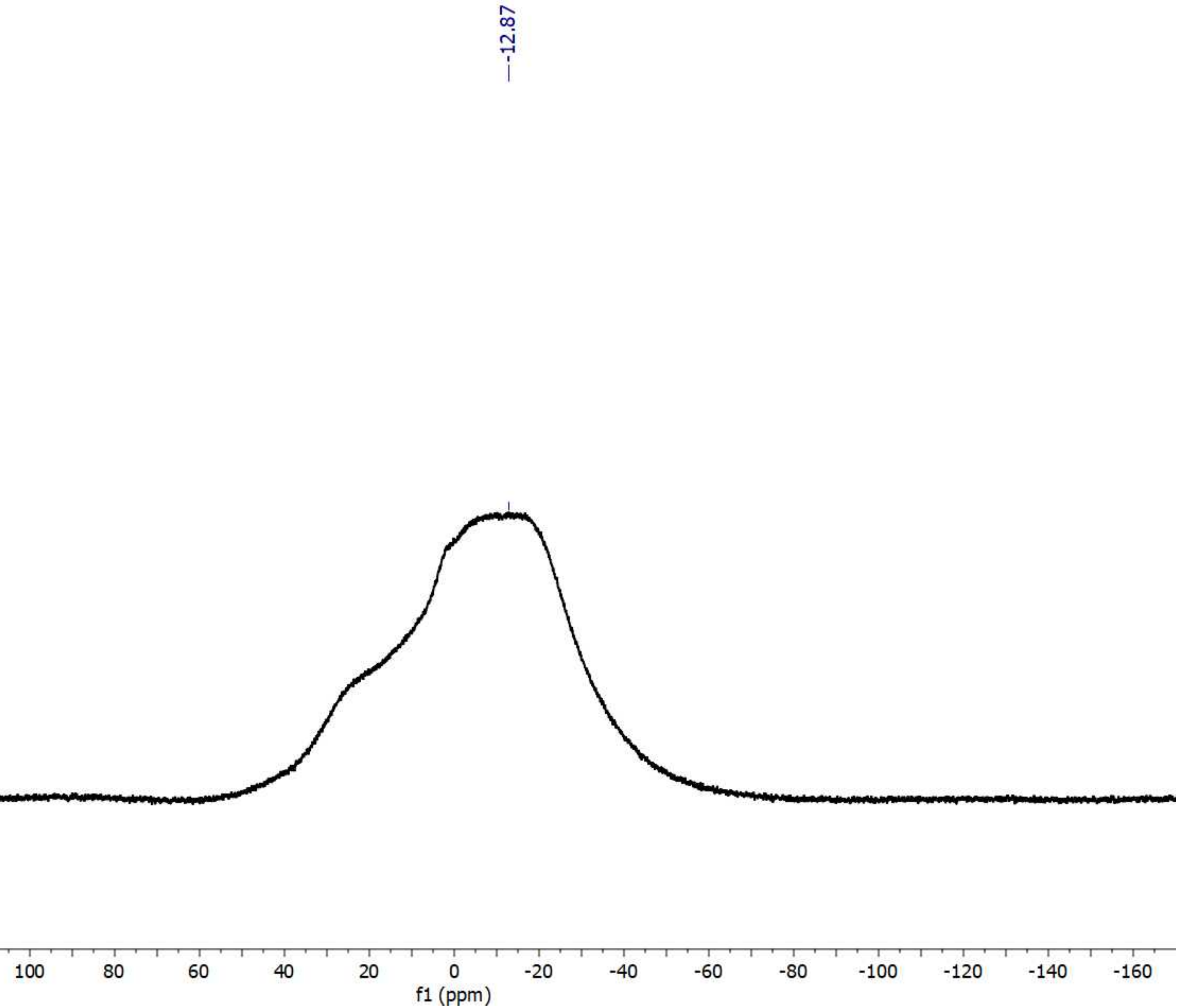

Figure S75. ^{11}B NMR (C_6D_6, 128 MHz) of **3-H**.

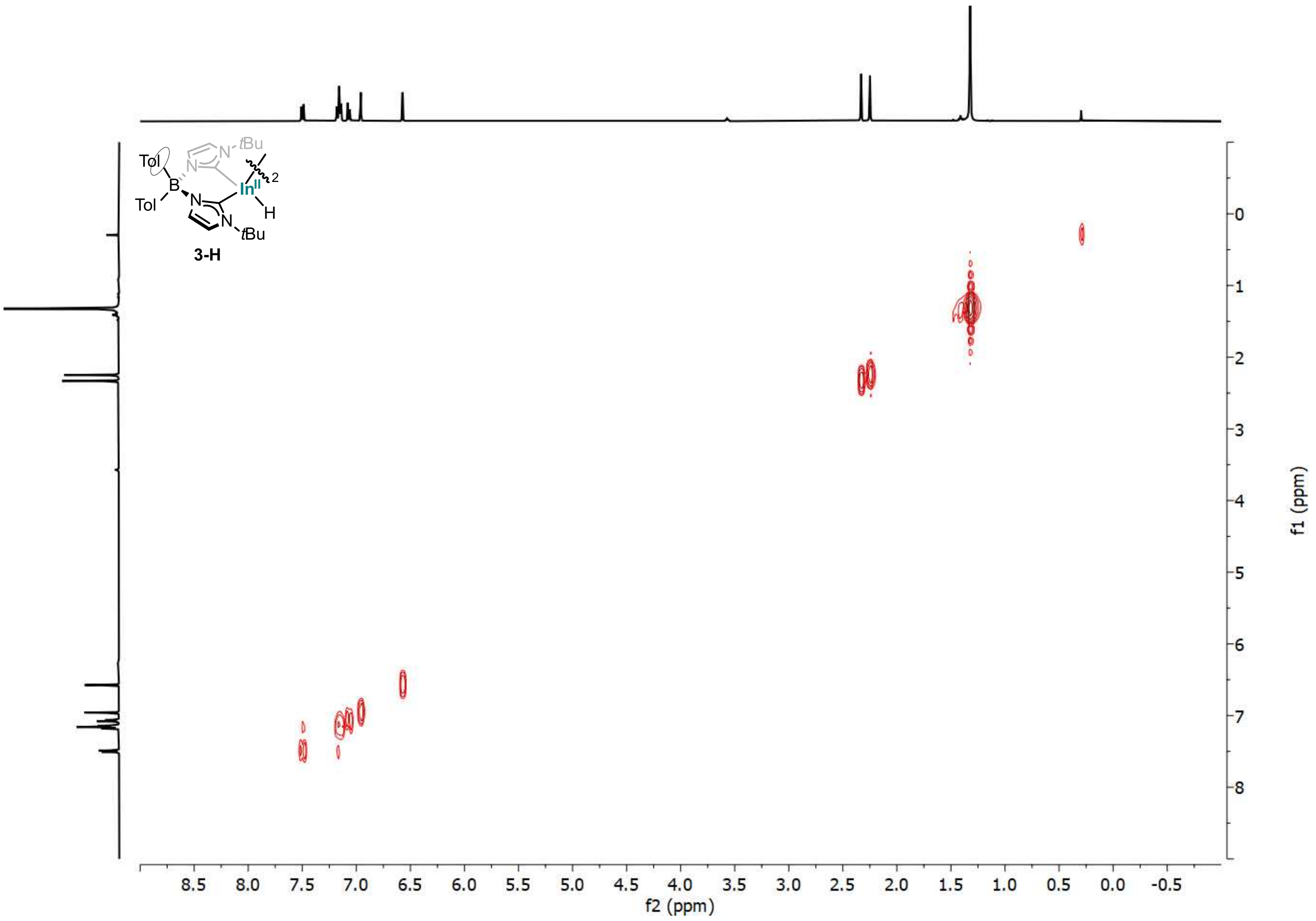

Figure S76. ^1H-^1H COSY NMR (C_6D_6, 400 MHz) of **3-H**.

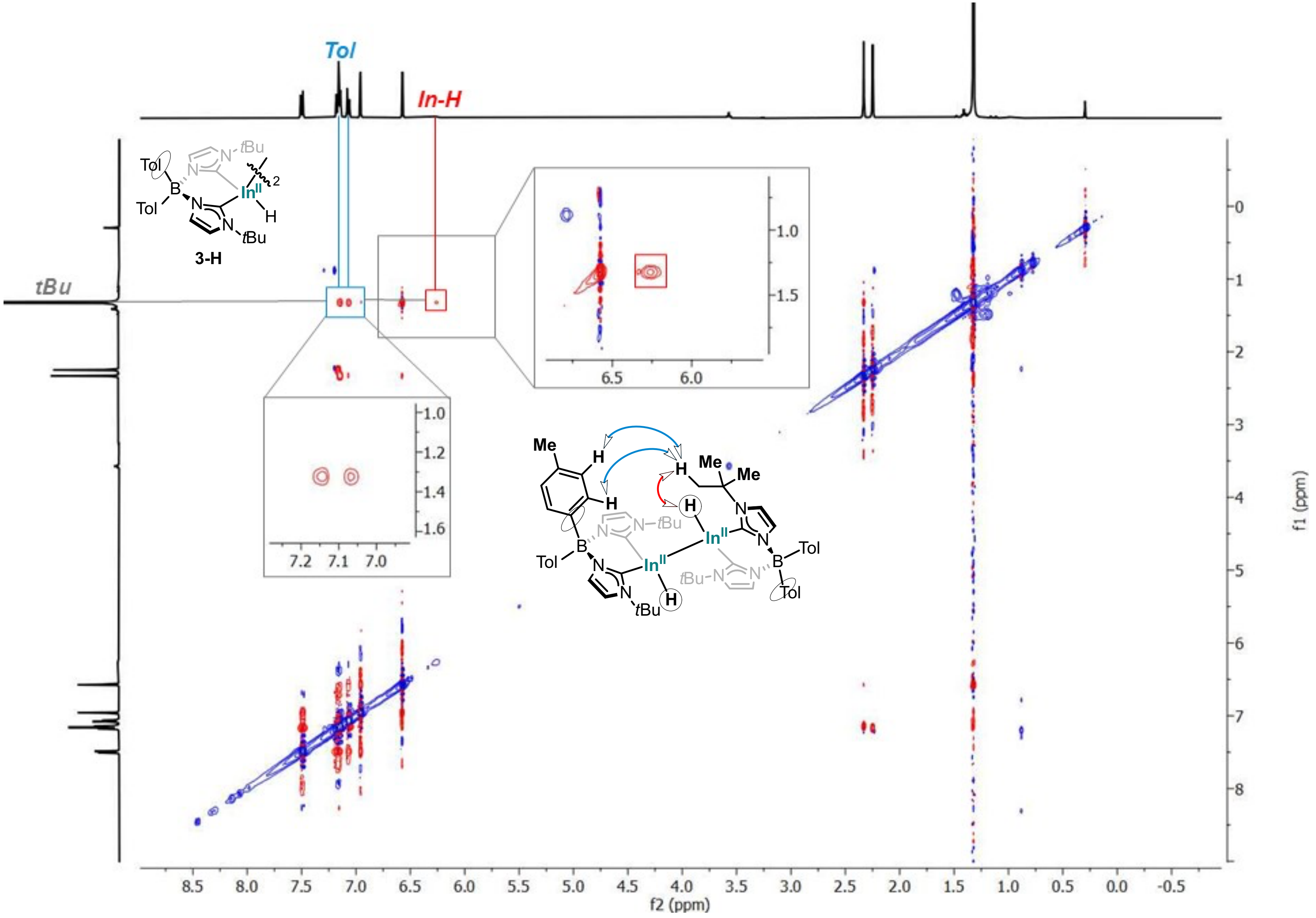

Figure S77. ^{1}H-^{1}H NOESY NMR (C_6D_6, 400 MHz, 298 K) of **3-H**.

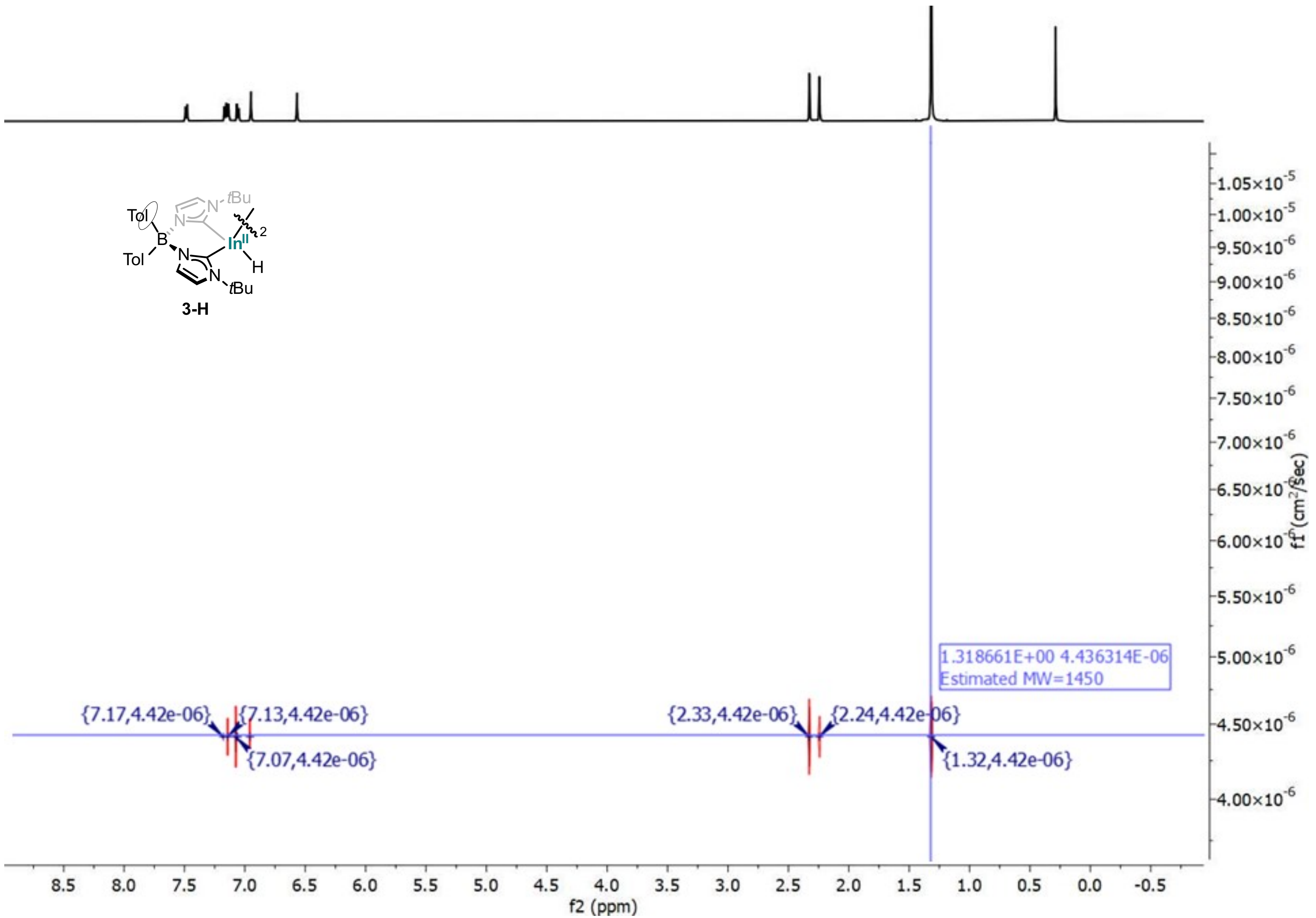

Figure S78. ^{1}H-^{1}H DOSY NMR (C_6D_6, 400 MHz) of **3-H**.

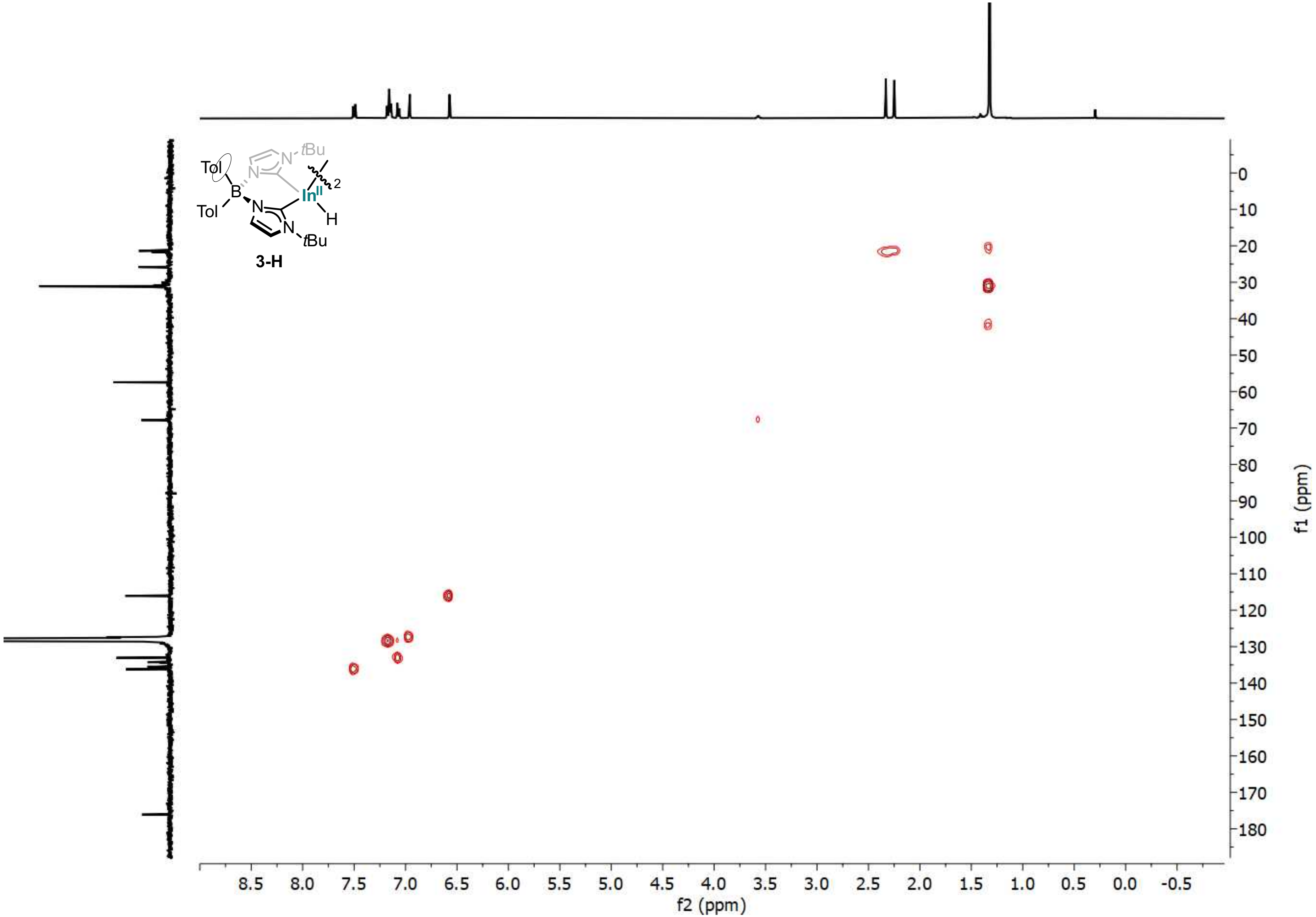

Figure S79. ^{1}H-^{13}C HSQC NMR (C_6D_6, 400 MHz) of **3-H**.

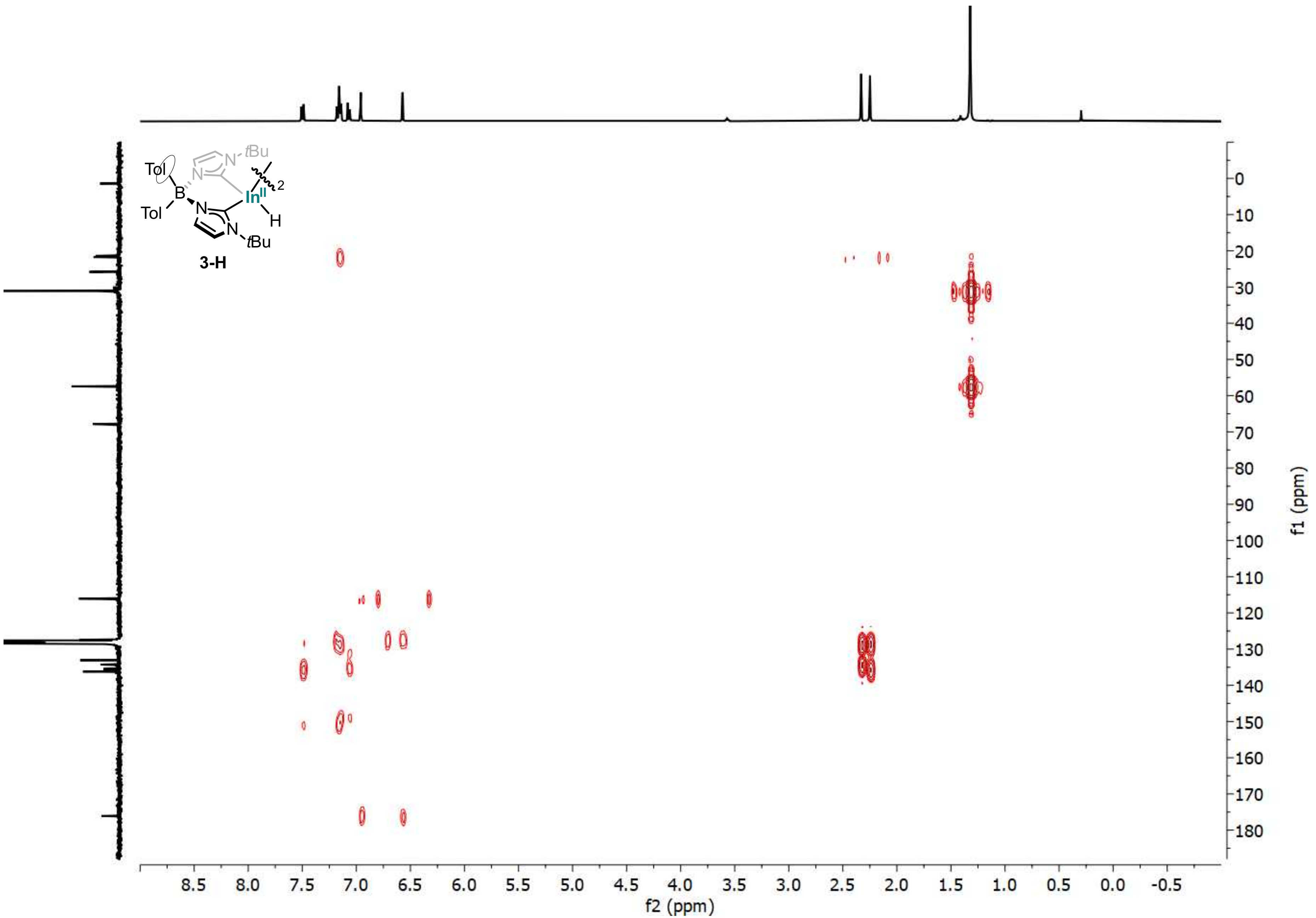

Figure S80. ^{1}H-^{13}C HMBC NMR (C_6D_6, 400 MHz) of **3-H**.

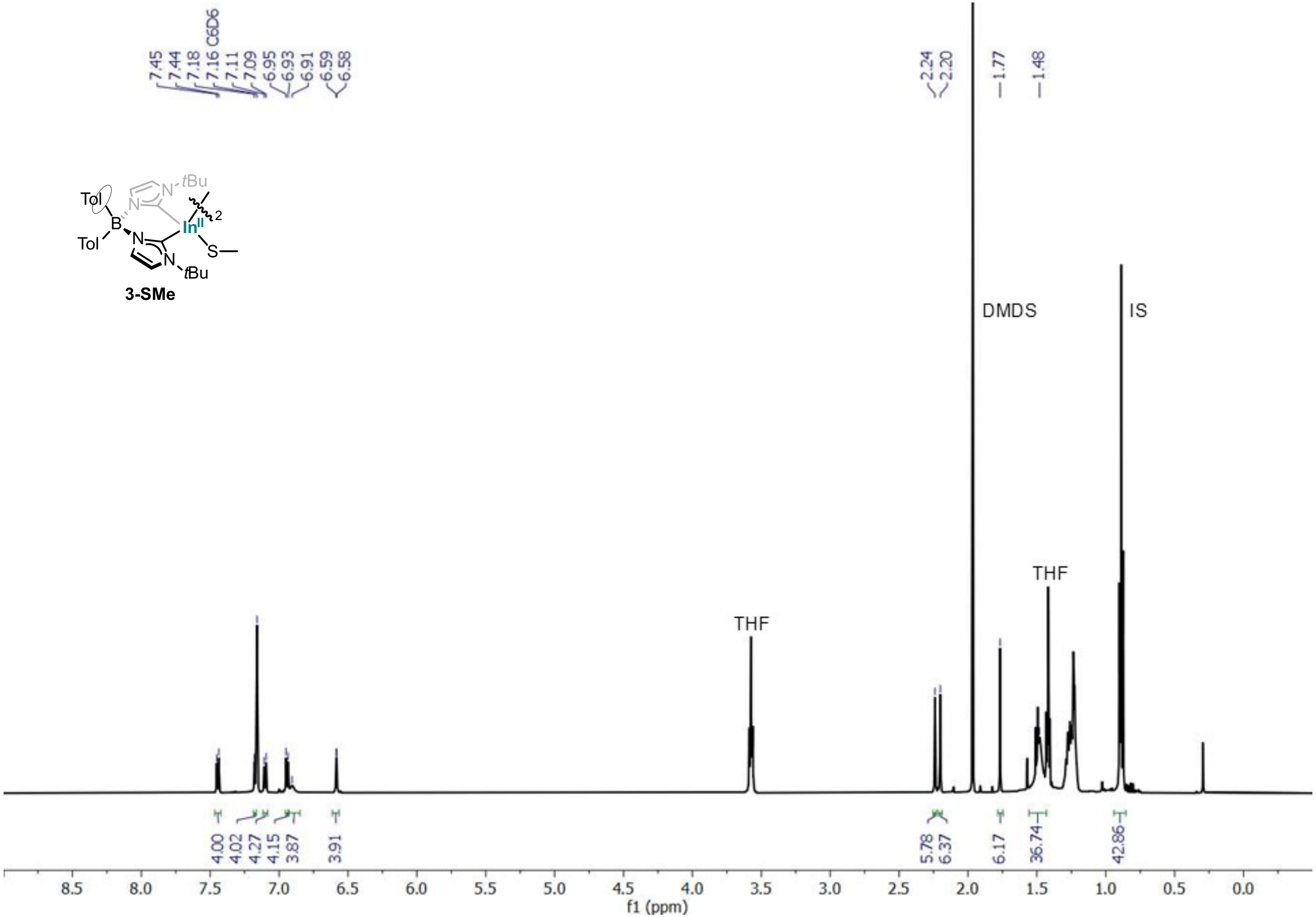

Figure S81. ^{1}H NMR (C_6D_6, 500 MHz, 298 K) of **3-SMe**.

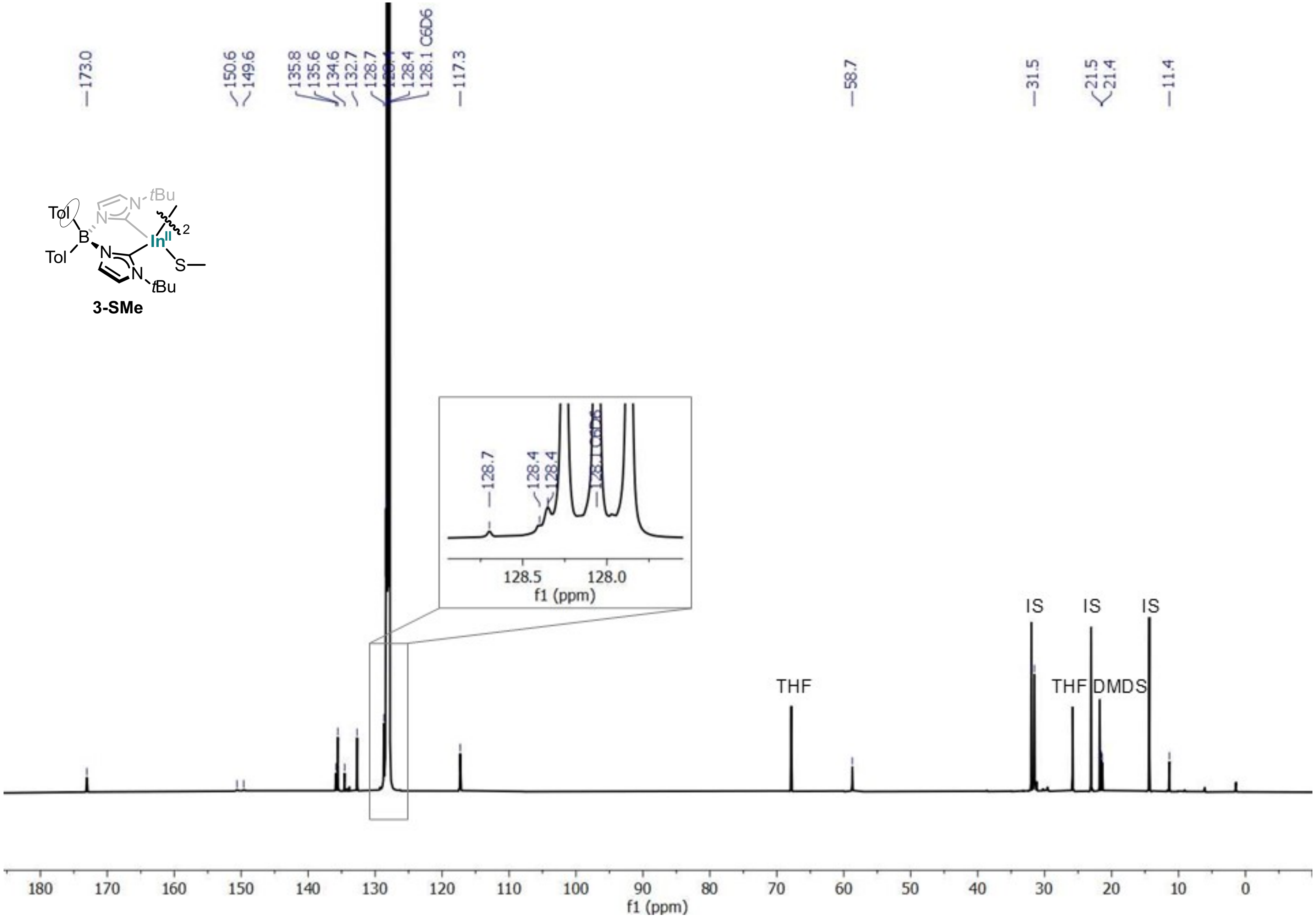

Figure S82. ^{13}C NMR (C_6D_6, 101 MHz, 298 K) of **3-SMe**.

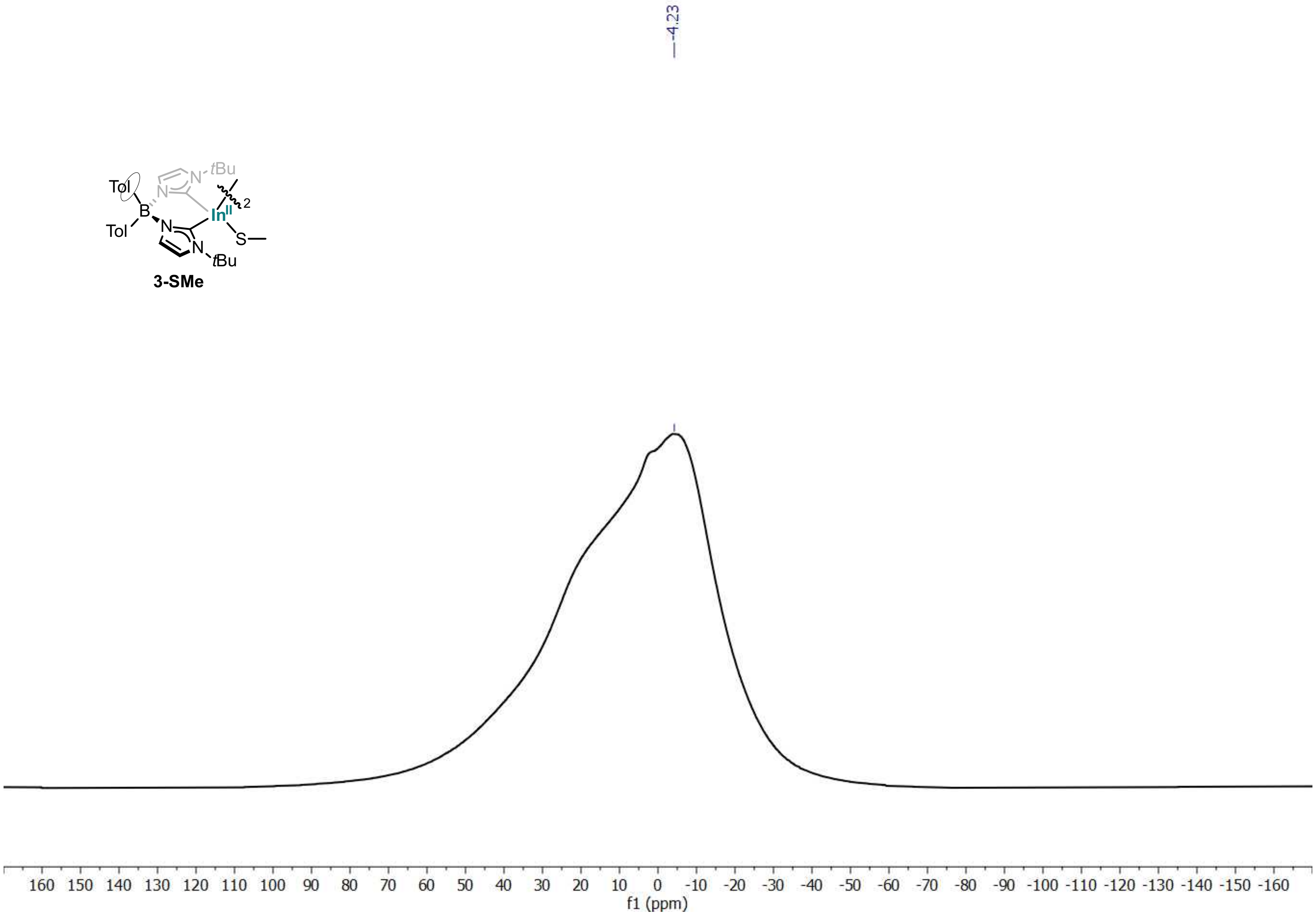

Figure S83. ^{13}C NMR (C_6D_6, 128 MHz, 298 K) of **3-SMe**.

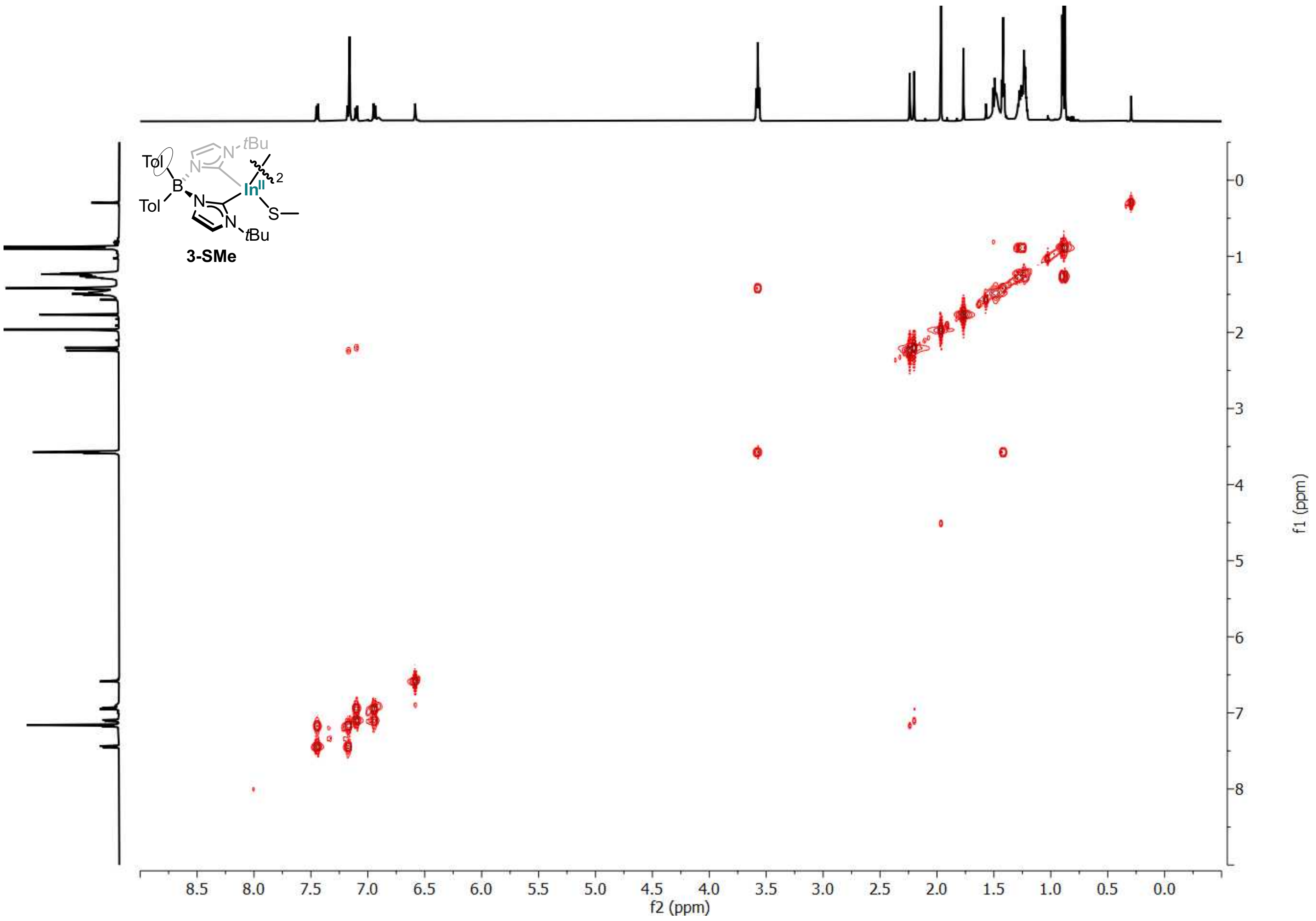

Figure S84. ^{1}H-^{1}H COSY NMR (C_6D_6, 500 MHz, 298 K) of **3-SMe**.

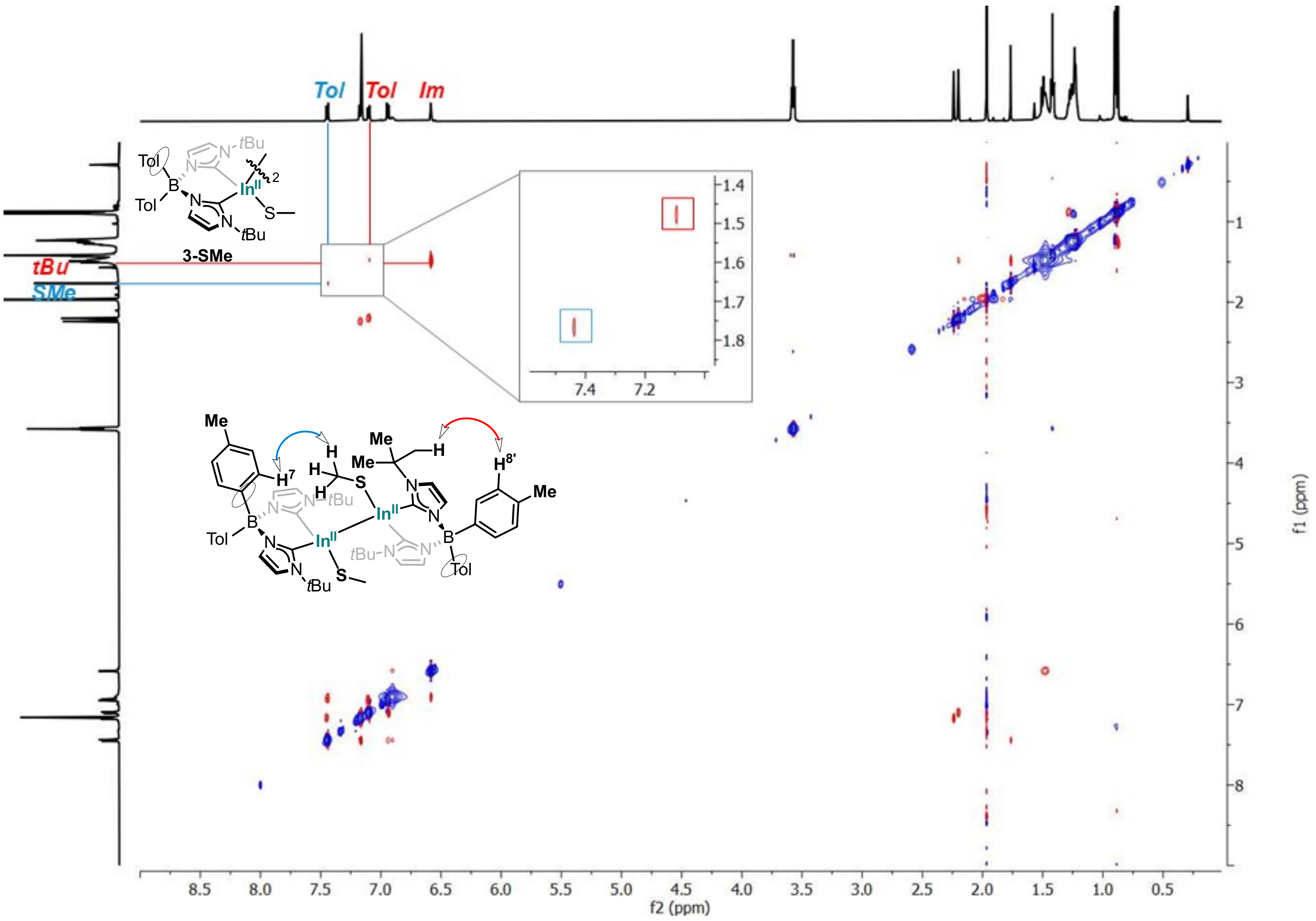

Figure S85. ^{1}H-^{1}H NOESY NMR (C_6D_6, 500 MHz, 298 K) of **3-SMe**.

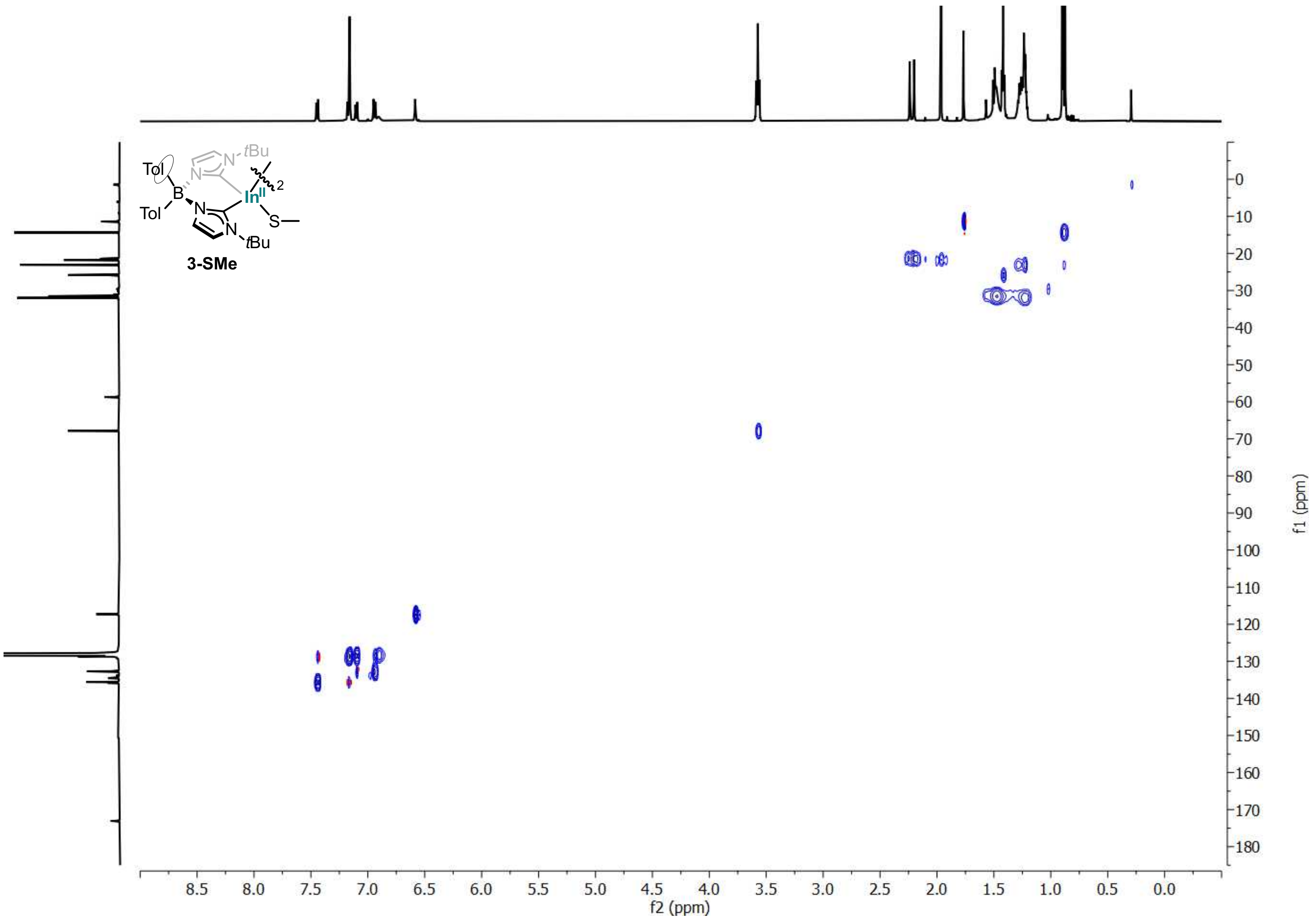

Figure S86. ^{1}H-^{13}C HSQC NMR (C_6D_6, 500 MHz, 298 K) of **3-SMe**.

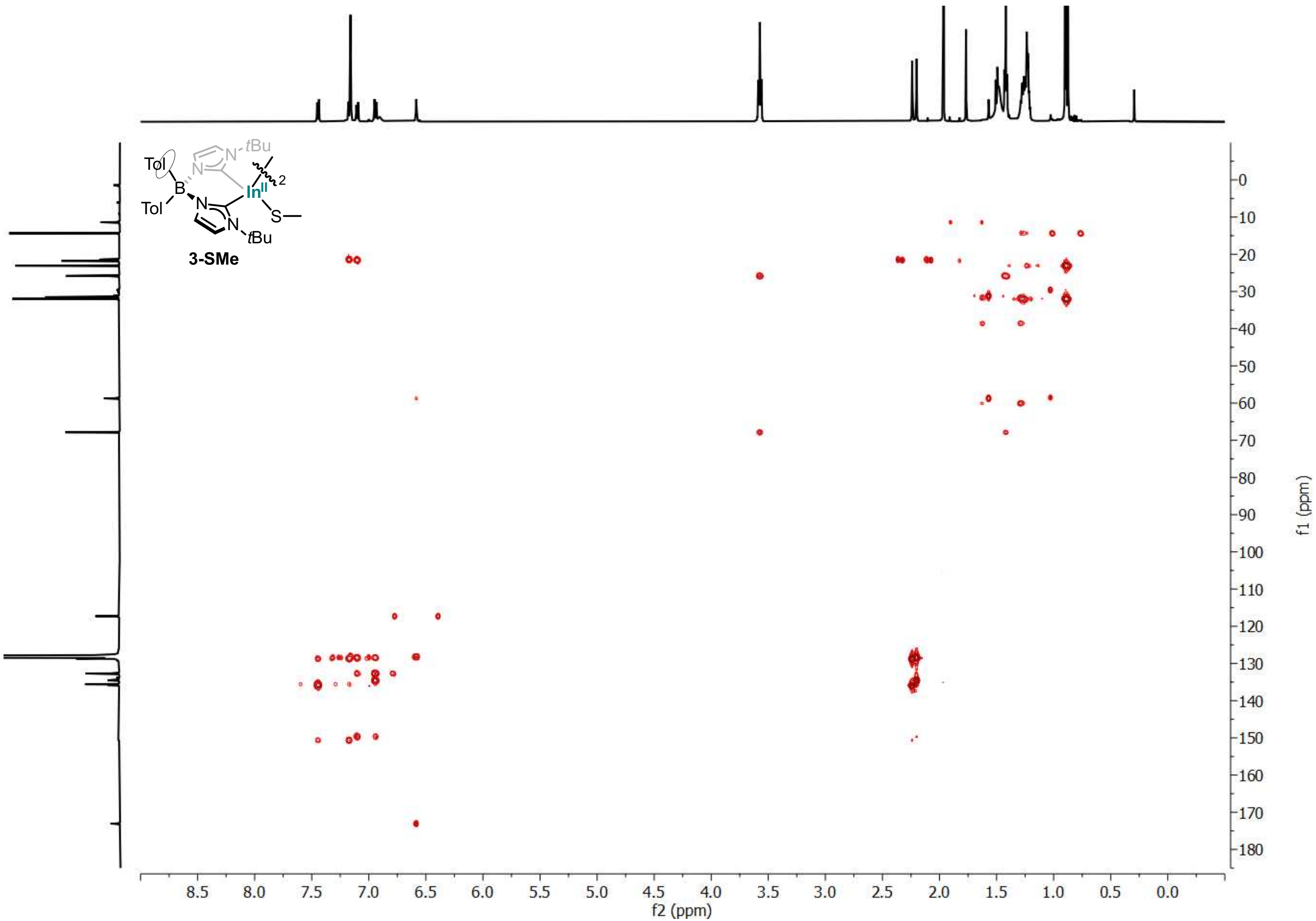

Figure S87. ^{1}H-^{13}C HMBC NMR (C_6D_6, 500 MHz, 298 K) of **3-SMe**.

9 References

(1) Fernando, S.; Chan, Y. C.; Fernandez, S.; Sabater, E.; Tizzard, G.; Coles, S. J.; Andrada, D. M. Planas, O. Zwitterionic Heavier Pnictinidenes in Redox Catalysis. *Angew. Chem. Int. Ed.* **2025**, *64,* e202505697.
https://onlinelibrary.wiley.com/doi/10.1002/anie.202505697.

(2) Vránová, I.; Alonso, M.; Lo, R.; Sedlák, R.; Jambor, R.; Růžička, A.; Proft, F. D.; Hobza, P.; Dostál, L. From Dibismuthenes to Three- and Two-Coordinated Bismuthinidenes by Fine Ligand Tuning: Evidence for Aromatic BiC3N Rings through a Combined Experimental and Theoretical Study. *Chem. Eur. J.* **2015**, *21*, 16917–16928. https://doi.org/10.1002/chem.201502724.

(3) Neese, F. Software Update: The ORCA Program System—Version 6.0. WIREs *Comput. Mol. Sci.* **2025**, *15*, e70019.
https://wires.onlinelibrary.wiley.com/doi/full/10.1002/wcms.70019

(4) Ernzerhof, M.; Scuseria, G. E. Assessment of the Perdew–Burke–Ernzerhof Exchange-Correlation Functional. *J. Chem. Phys.* **1999**, *110*, 5029–5036. https://doi.org/10.1063/1.478401.

(5) Adamo, C.; Barone, V. Toward Reliable Density Functional Methods without Adjustable Parameters: The PBE0 Model. *J. Chem. Phys.* **1999**, *110*, 6158–6170. https://doi.org/10.1063/1.478522.

(6) Grimme, S.; Antony, J.; Ehrlich, S.; Krieg, H. A Consistent and Accurate Ab Initio Parametrization of Density Functional Dispersion Correction (DFT-D) for the 94 Elements H-Pu. *J. Chem. Phys.* **2010**, *132*, 154104. https://doi.org/10.1063/1.3382344.

(7) Grimme, S.; Ehrlich, S.; Goerigk, L. Effect of the Damping Function in Dispersion Corrected Density Functional Theory. *J. Comput. Chem.* **2011**, *32*, 1456–1465. https://doi.org/10.1002/jcc.21759.

(8) Caldeweyher, E.; Bannwarth, C.; Grimme, S. Extension of the D3 Dispersion Coefficient Model. *J. Chem. Phys.* **2017**, *147*, 034112. https://doi.org/10.1063/1.4993215.

(9) Caldeweyher, E.; Ehlert, S.; Hansen, A.; Neugebauer, H.; Spicher, S.; Bannwarth, C.; Grimme, S. A Generally Applicable Atomic-Charge Dependent London Dispersion Correction. *J. Chem. Phys.* **2019**, *150*, 154122. https://doi.org/10.1063/1.5090222.

(10) Weigend, F.; Ahlrichs, R. Balanced Basis Sets of Split Valence, Triple Zeta Valence and Quadruple Zeta Valence Quality for H to Rn: Design and Assessment of Accuracy. *Phys. Chem. Chem. Phys.* **2005**, *7*, 3297–3305. https://doi.org/10.1039/B508541A.

(11) McIver, J. W. Jr.; Komornicki, A. Structure of Transition States in Organic Reactions. General Theory and an Application to the Cyclobutene-Butadiene Isomerization Using a

Semiempirical Molecular Orbital Method. *J. Am. Chem. Soc.* **1972**, *94*, 2625–2633. https://doi.org/10.1021/ja00763a011.

(12) Reed, A. E.; Curtiss, L. A.; Weinhold, F. Intermolecular Interactions from a Natural Bond Orbital, Donor-Acceptor Viewpoint. *Chem. Rev.* **1988**, *88*, 899–926. https://doi.org/10.1021/cr00088a005.

(13) Reed, A. E.; Weinstock, R. B.; Weinhold, F. Natural Population Analysis. *J. Chem. Phys.* **1985**, *83*, 735–746. https://doi.org/10.1063/1.449486.

(14) Glendening, E. D.; Badenhoop, J. K.; Reed, A. E.; Carpenter, J. E.; Bohmann, J. A.; Morales, C. M.; Karafiloglou, P.; Landis, C. R.; Weinhold, F., NBO 7.0. Theoretical Chemistry Institute, University of Wisconsin, Madison, **2018**.

(15) Bader, R. F. W.; Bader, R. F. Atoms in Molecules: A Quantum Theory; International series of monographs on chemistry; Clarendon Press, **1990**.

(16) AIMAll (Version 19.10.12), Todd A. Keith, TK Gristmill Software, Overland Park KS, USA, **2019**.

(17) Morokuma, K. Molecular Orbital Studies of Hydrogen Bonds. III. C=O···H–O Hydrogen Bond in H2CO···H2O and H2CO···2H2O. *J. Chem. Phys.* **1971**, *55*, 1236–1244. https://doi.org/10.1063/1.1676210.

(18) Ziegler, T.; Rauk, A. Carbon Monoxide, Carbon Monosulfide, Molecular Nitrogen, Phosphorus Trifluoride, and Methyl Isocyanide as .Sigma. Donors and .Pi. Acceptors. A Theoretical Study by the Hartree-Fock-Slater Transition-State Method. *Inorg. Chem.* **1979**, *18*, 1755–1759. https://doi.org/10.1021/ic50197a006.

(19) Ziegler, T.; Rauk, A. A Theoretical Study of the Ethylene-Metal Bond in Complexes between Copper(1+), Silver(1+), Gold(1+), Platinum(0) or Platinum(2+) and Ethylene, Based on the Hartree-Fock-Slater Transition-State Method. *Inorg. Chem.* **1979**, *18*, 1558–1565. https://doi.org/10.1021/ic50196a034.

(20) Bickelhaupt, F. M.; Nibbering, N. M. M.; Van Wezenbeek, E. M.; Baerends, E. J. Central Bond in the Three CN.Cntdot.Dimers NC-CN, CN-CN and CN-NC: Electron Pair Bonding and Pauli Repulsion Effects. *J. Phys. Chem.* **1992**, *96*, 4864–4873. https://doi.org/10.1021/j100191a027.

(21) Zhao, L.; von Hopffgarten, M.; Andrada, D. M.; Frenking, G. Energy Decomposition Analysis. WIREs Comput. Mol. Sci. **2018**, 8, e1345. https://doi.org/10.1002/wcms.1345.

(22) Michalak, A.; Mitoraj, M.; Ziegler, T. Bond Orbitals from Chemical Valence Theory. *J. Phys. Chem. A* **2008**, *112*, 1933–1939. https://doi.org/10.1021/jp075460u.

(23) Mitoraj, M.; Michalak, A. Applications of Natural Orbitals for Chemical Valence in a Description of Bonding in Conjugated Molecules. *J. Mol. Model.* **2008**, *14*, 681–687. https://doi.org/10.1007/s00894-008-0276-1.

(24) Mitoraj, M. P.; Michalak, A.; Ziegler, T. A Combined Charge and Energy Decomposition Scheme for Bond Analysis. *J. Chem. Theory Comput.* **2009**, *5*, 962–975. https://doi.org/10.1021/ct800503d.

(25) te Velde, G.; Bickelhaupt, F. M.; Baerends, E. J.; Fonseca Guerra, C.; van Gisbergen, J. A.; Snijders, J. G.; Ziegler, T. Chemistry with ADF. *J. Comput. Chem.* **2001**, *22*, 931–967. https://onlinelibrary.wiley.com/doi/10.1002/jcc.1056.

(26) Becke, A. D. Density-Functional Exchange-Energy Approximation with Correct Asymptotic Behavior. *Phys. Rev. A* **1988**, *38*, 3098–3100. https://doi.org/10.1103/PhysRevA.38.3098.

(27) Perdew, J. P. Density-Functional Approximation for the Correlation Energy of the Inhomogeneous Electron Gas. *Phys. Rev. B* **1986**, *33*, 8822–8824. https://doi.org/10.1103/PhysRevB.33.8822.

(28) Lenthe, E. van; Baerends, E. J.; Snijders, J. G. Relativistic Regular Two-component Hamiltonians. *J. Chem. Phys.* **1993**, *99*, 4597–4610. https://doi.org/10.1063/1.466059.

(29) Lu, T. A Comprehensive Electron Wavefunction Analysis Toolbox for Chemists, Multiwfn. *J. Chem. Phys.* **2024**, *161*, 082503. https://doi.org/10.1063/5.0216272.

(30) Sheldrick, G. M. SHELXT – Integrated Space-Group and Crystal-Structure Determination. *Acta Cryst A* **2015**, *71*, 3–8. https://doi.org/10.1107/S2053273314026370.

(31) Dolomanov, O. V.; Bourhis, L. J.; Gildea, R. J.; Howard, J. a. K.; Puschmann, H. OLEX2: A Complete Structure Solution, Refinement and Analysis Program. *J. Appl. Cryst.* **2009**, *42*, 339–341. https://doi.org/10.1107/S0021889808042726.

(32) Bourhis, L. J.; Dolomanov, O. V.; Gildea, R. J.; Howard, J. A. K.; Puschmann, H. The Anatomy of a Comprehensive Constrained, Restrained Refinement Program for the Modern Computing Environment – It Olex2 Dissected. *Act. Cryst. A* **2015**, *71*, 59–75. https://doi.org/10.1107/S2053273314022207.

(33) Kleemiss, F.; Dolomanov, O. V.; Bodensteiner, M.; Peyerimhoff, N.; Midgley, L.; Bourhis, L. J.; Genoni, A.; Malaspina, L. A.; Jayatilaka, D.; Spencer, J. L.; White, F.; Grundkötter-Stock, B.; Steinhauer, S.; Lentz, D.; Puschmann, H.; Grabowsky, S. Accurate Crystal Structures and Chemical Properties from NoSpherA2. *Chem. Sci.* **2021**, *12*, 1675–1692. https://doi.org/10.1039/D0SC05526C.

(34) Clark, R. C.; Reid, J. S. The Analytical Calculation of Absorption in Multifaceted Crystals. *Act. Cryst A* **1995**, *51*, 887–897. https://doi.org/10.1107/S0108767395007367.
